# Facile Access to Dative, Single, and Double Silicon−Metal Bonds Through M−Cl Insertion Reactions of Base‐Stabilized Si^II^ Cations

**DOI:** 10.1002/chem.202000866

**Published:** 2020-04-28

**Authors:** Philipp Frisch, Tibor Szilvási, Shigeyoshi Inoue

**Affiliations:** ^1^ Department of Chemistry WACKER-Institute of Silicon Chemistry and Catalysis Research Center Technische Universität München Lichtenbergstraße 4 85748 Garching bei München Germany; ^2^ Department of Chemical and Biological Engineering University of Wisconsin-Madison 1415 Engineering Drive Madison Wisconsin 53706-1607 USA

**Keywords:** carbene ligands, insertion, reduction, silylene, transition metals

## Abstract

Silicon(II) cations can offer fascinating reactivity patterns due to their unique electronic structure: a lone pair of electrons, two empty p orbitals and a positive charge combined on a single silicon center. We now report the facile insertion of N‐heterocyclic carbene (NHC)‐stabilized silyliumylidene ions into M−Cl bonds (M=Ru, Rh), forming a series of novel chlorosilylene transition‐metal complexes. Theoretical investigations revealed a reaction mechanism in which the insertion into the M−Cl bond with concomitant 1,2‐migration of a silicon‐bound NHC to the transition metal takes place after formation of an initial silyliumylidene transition‐metal complex. The mechanism could be verified experimentally through characterization of the intermediate complexes. Furthermore, the obtained chlorosilylene complexes can be conveniently utilized as synthons to access Si−M and Si=M bonding motifs bonds through reductive dehalogenation.

## Graphical abstract

**A facile access route** to Si→M, Si−M, and Si=M (M=Ru, Rh) moieties through the insertion of NHC‐stabilized silyliumylidene ions into metal−chloride bonds and subsequent reductive dehalogenation is reported. The coordination–insertion–migration mechanism was investigated theoretically and verified experimentally through NMR and XRD characterization of the intermediate silyliumylidene transition‐metal coordination complexes.

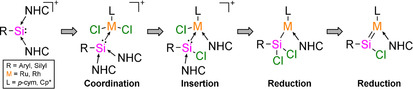

## Introduction

The presence of a lone pair of electrons, two vacant orbitals and a positive charge on the silicon center makes silyliumylidene ions an incredibly versatile and promising class of low‐valent silicon compounds.^1^ They offer a large, yet untapped synthetic potential in organosilicon chemistry with the possibility to form up to three new bonds in a single reaction.^2^ They are promising candidates for the activation of small molecules, transition metal free catalysis^3^ and can act as synthons for novel (low‐valent) silicon compounds. Further, with the presence of a stereochemically active lone pair, they can also function as ligands in transition metal complexes. So far, no one‐coordinate Si^II^ cation has been isolated^4^ and most reported examples are three‐coordinate and utilize two Lewis bases for their stabilization (e.g. NHCs).^5^ This brings the drawback of a generally reduced reactivity by blocking the empty *p*‐orbitals.

Hence, both amount and diversity of reported reactivities lag behind those of silylenes, where common reactivity patterns include insertion reactions into various types of (strong) bonds. A staggering number of examples for the insertion into E−H (E=H, N, O, S, C, B, …) and E−Halogen bonds have been reported in recent years.^6^ Similarly, the coordination chemistry of silylenes with transition metals is a continuously expanding research field with various catalytic applications.6d, 7

In contrast, even as the number of isolable base‐stabilized silyliumylidenes continues to grow,4a, 8 reported reactivities remain scarce.^5^, ^9^ Only few reactivity studies with small molecules^10^ have been found and E−H bond activation reactions are limited to S−H, O−H and acidic C−H bonds.8g, 11

The chemistry of Si^II^ cations as transition metal ligands has seen some progress in recent years.^12^ Reported examples include complexes with coinage metals12d and group 6 and 8 metal carbonyls,12b, 12e but no further reactivity of these complexes has been reported to date. Importantly, the synthesis of new types of complexes with silicon‐based ligands and substituents is of high interest for the development of improved catalysts.7a, 7b, 7c, 13 With their intriguing synthetic potential, silyliumylidenes are uniquely suited for the facile synthesis of various types of Si−M (multiple) bonds (e.g. through salt metathesis or formation of coordination complexes followed by abstraction/migration of stabilizing Lewis bases). This was elegantly demonstrated by Filippou et al. with the direct synthesis of a molybdenum silylidyne complex.12c

**Scheme 1:**
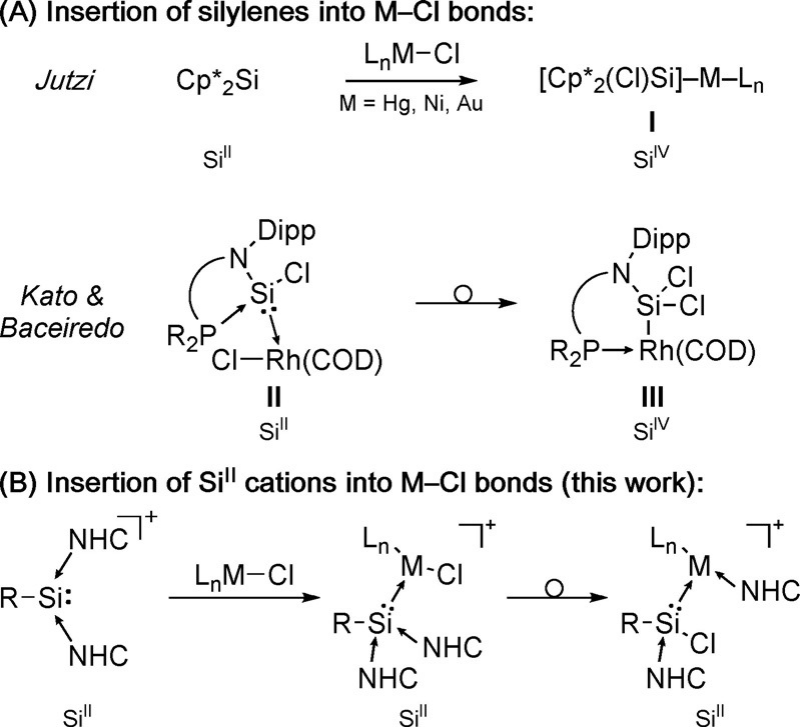
(A) Examples for silylene insertion reactions into M−Cl bonds. (B) Formation of chlorosilylenes via insertion of Si^II^ cations into M−Cl bonds.

For silylene complexes, a variety of follow‐up chemistry is known.6d, 7 For instance, multiple insertion reactions into metal‐chloride bonds of a coordinated transition metal fragment have been reported. For example, Jutzi and co‐workers disclosed the insertion of Decamethylsilicocene into a Hg−X bond, furnishing silyl‐substituted Hg compounds (**I**, Scheme 1 A).^14^ The group further reported analogous insertion reactions into Ni−Cl and Au−Cl bonds^15^ and related reactivities with Pt−Cl bonds were also reported by Lappert et al.^16^ Recently, Kato, Baceiredo, and co‐workers reported the insertion of a chlorosilylene ligand into the Rh−Cl bond of a coordinated [RhCl(COD)] fragment (**II**), forming the corresponding RSiCl_2_−Rh(COD) compound **III**.^17^ For silyliumylidene ions and their transition‐metal complexes, no analogous reactivity has been observed so far. In fact, insertion reactions into E−Halogen bonds have not been reported at all.

Herein, we now report the first reactivity studies regarding insertion reactions of a Si^II^ cation into transition metal‐chloride bonds. Reactions of NHC‐stabilized silyliumylidene ions with dimeric, chloro‐bridged transition‐metal precursors lead to coordination of the Si^II^ cation to the metal fragment, followed by insertion of the silyliumylidene ligand into the M−Cl (M=Ru, Rh) bond, furnishing NHC‐stabilized transition metal silylene complexes (Scheme 1 B). The complexes have been fully characterized by multinuclear NMR spectroscopy and SC‐XRD (single crystal X‐ray diffraction) and the insertion mechanism has been investigated theoretically and verified experimentally. Furthermore, we present a facile access route to Si−M and Si=M bonds through stepwise reduction of the isolated complexes with KC_8_, initially furnishing silyl‐substituted complexes, followed by the formation of the corresponding Si=Ru double bond through additional reductive dehalogenation. Importantly, while these types of insertion reactions are generally accompanied by an increase of the silicon oxidation state from II to IV, no such change occurs for silyliumylidene ions (cf. Scheme 1).

## Results and Discussion

### Insertion of a Si^II^ cation into a Ru−Cl Bond

**Scheme 2:**
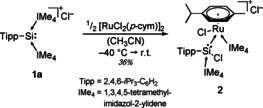
Synthesis of chlorosilylene ruthenium complex **2**.

While exploring the coordination chemistry of NHC‐stabilized Si^II^ cations, we investigated the reaction of the Tipp‐substituted silyliumylidene ion **1 a** 8g with the transition metal precursor [RuCl_2_(*p*‐cym)]_2_ (Scheme 2, *p*‐cym=1‐Me‐4‐*i*Pr‐benzene). Addition of cold acetonitrile to a mixture of **1 a** and the precursor at −40 °C led to an immediate color change of the solution to deep red. At about −20 to −15 °C, the color of the solution rapidly changed to orange. Even at −40 °C, a color change to orange can be observed within 2 hours. The ^29^Si NMR of the orange solution displays one resonance with an expected downfield shift at 17.6 ppm (from −69.5 ppm (**1 a**)8g), indicating the formation of a single coordination product. Interestingly, the corresponding ^1^H NMR (cf. Supporting Information, Figure S8) showed a highly asymmetric species with four separate septets and eight doublets (corresponding to four chemically unique *iso*‐propyl groups) and two distinct signal sets for the NHCs. The ^13^C NMR showed two resonances for the carbene carbon atoms at 169.9 and 154.8 ppm, indicating the possible migration of one NHC to the transition metal.

**Figure 1:**
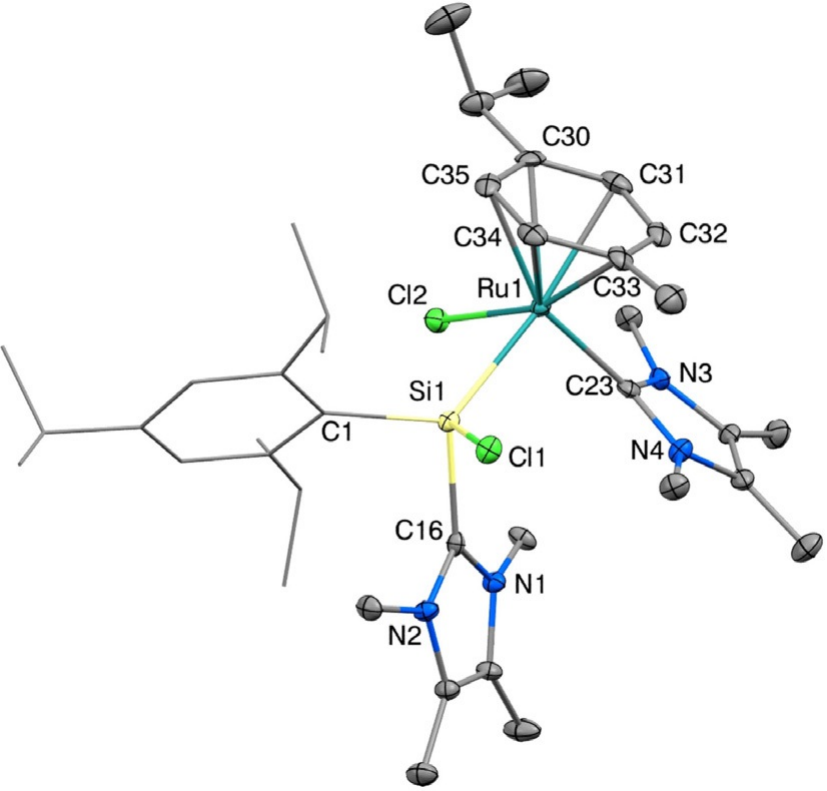
Ellipsoid plot (50 %) of the molecular structure of **2**. Hydrogen atoms and the anion are omitted. The Tipp substituent is simplified as a wireframe for clarity. Selected bond lengths [Å] and angles [°]: Si1−Ru1 2.409(1), Si1−Cl1 2.167(1), Si1−C1 1.941(4), Si1−C16 1.970(4), Ru1−Cl2 2.404(1), Ru1−C23 2.077(4), Ru1−*p*‐cym_⊥_ 1.770(1), C1−Si1−Ru1 123.3(1), Si1−Ru1−*p*‐cym_⊥_ 131.1(1), Cl1−Si1−Ru1−Cl2 −173.1(1), C16−Si1−Ru1−C23 −11.1(2).

The complex rapidly decomposes at room temperature in solution to a mixture of products, making further investigation and functionalization difficult. Nevertheless, crystals suitable for SC‐XRD analysis could be obtained by storing a concentrated solution of **2** in MeCN at −35 °C. Figure 1 shows the solid‐state structure of **2**, unambiguously confirming the asymmetric nature of the complex and the shift of one NHC to the metal. The half‐sandwich complex with a piano‐stool configuration features an NHC‐ stabilized aryl‐chlorosilylene ligand with a tetrahedral coordination sphere around the silicon center and a Si1−Ru1 bond length (2.409(1) Å) typical for Si−Ru bonds.^18^ The Si−C_NHC_ (1.970(4) Å) and Ru−C_NHC_ (2.077(4) Å) bond lengths are in the typical range for Si−C_NHC_ and Ru−C_NHC_ bonds.

It is worth noting that attempts to stabilize the complex by employing the significantly bulkier *m*‐terphenyl (2,6‐(2,4,6‐Me_3_‐C_6_H_2_)_2_‐C_6_H_3_) substituent were unsuccessful and no reaction could be observed, presumably due to its large steric hindrance. Similarly, we envisioned the introduction of a Cp* ligand (Cp*=1,2,3,4,5‐pentamethyl‐cyclopentadienyl) on the metal. The *p*‐cymene ligand is often a weak spot in such complexes, as it can be relatively easily cleaved from the metal. Unfortunately, no reaction of the related precursor [RhCl_2_(Cp*)]_2_ with **1 a** could be observed, most likely due to the increased steric demand of the Cp* substituent.

### Formation of complex 2—mechanistic insights

**Figure 2:**
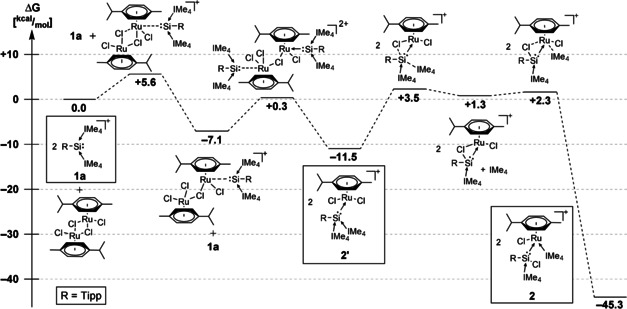
DFT‐derived reaction mechanism and energy profile for the formation of **2** from **1 a** via **2′**.

To elucidate the mechanism of formation of chlorosilylene complex **2**, we performed DFT calculations at the B97‐D/def2‐SVP level of theory (Figure 2). In a first step, the coordination of a silyliumylidene moiety to each transition metal center leads to the splitting of the dimer, forming the silyliumylidene complex **2′**. This also indicates why no reaction could be observed at all for the significantly bulkier *m*‐terphenyl and Cp*: the initial coordination step is blocked due to their large steric hindrance, which completely stops any product formation. After the coordination, the insertion reaction of the low‐valent silicon into the Ru−Cl bond occurs with concomitant 1,2‐migration of one NHC moiety to the transition metal. We have previously observed a related NHC migration reaction involving NHC‐stabilized silyliumylidene ions with the formation of a [(IMe_4_)_2_Au]Cl complex from a silyliumylidene gold complex.12d This migration/insertion reaction is similar to the mentioned insertion reaction of a chlorosilylene ligand into a Rh−Cl bond (**II**→**III**, Scheme 1).^17^ However, a key distinction to the insertion reactions of silylenes is that in the case of the Si^II^ cation, the formal oxidation state of the silicon center does not change: here, the insertion reaction leads from [R−Si^II^]^+^ to [R−Si^II^−Cl], whereas silylenes [R_2_Si^II^] yield silyl‐substituted complexes [R_2_ClSi^IV^−M] (cf. Scheme 1).

Based on the calculated reaction profile we presumed that the deep red species observed at low temperatures during the synthesis should be the silyliumylidene complex **2′**. Indeed, low‐temperature ^29^Si NMR analysis (−30 °C) showed a weak resonance at considerably higher field (−21.1 ppm vs. +17.6 ppm for **2**) that immediately vanished upon warming and even disappeared at low temperatures within 2 hours. This upfield shifted resonance is expected for a Si^II^ center with two coordinated NHC moieties and is in line with our previously reported group 6 silyliumylidene complexes (Cr: +6.3 ppm; Mo: −17.3 ppm; W: −30.5 ppm and the related iron complex (+5.4 ppm)).12e To further reinforce the suggestion that **2′** is in fact the intermediate observed at low temperatures, we calculated the ^29^Si NMR shifts for **2** and **2′**: we find that the calculated chemical shifts (19.8 ppm for **2** and −23.4 ppm for **2′** (HCTH407/def2‐SVP//B97‐D/def2‐SVP)) are in good agreement with the experimentally observed values.

**Scheme 3:**
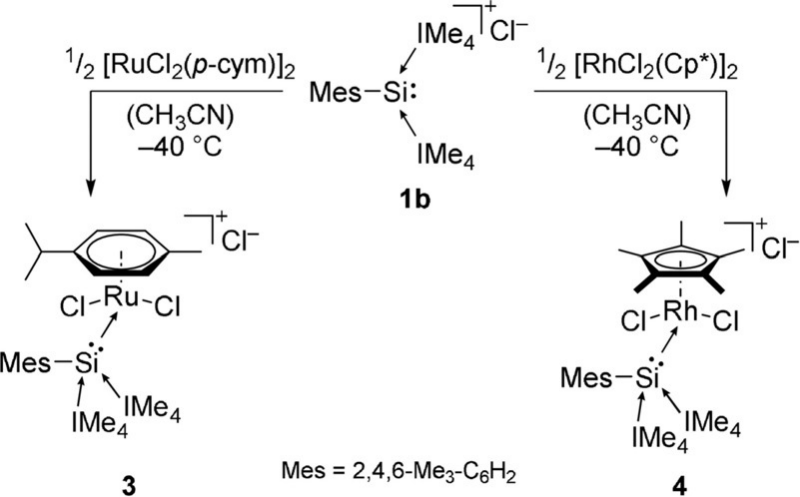
Synthesis of silyliumylidene complexes **3** and **4**.

**Figure 3:**
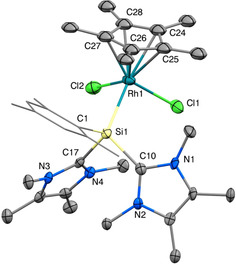
Ellipsoid plot (50 %) of the molecular structure of **4**. Hydrogen atoms and the anion are omitted. The mesityl substituent is simplified as a wireframe for clarity. Selected bond lengths [Å] and angles [°]: Si1−Rh1 2.426(2), Si1−C1 1.899(7), Si1−C10 1.958(7), Si1−C17 1.944(7), Rh1−Cl1 2.420(2), Rh1−Cl2 2.404(2), Rh1−Cp*_⊥_ 1.857(1), C1−Si1−Rh1 112.7(2), C10−Si1−C17 93.9(3), Si1−Rh1−Cp*_⊥_ 132.3(1).

Due to the relatively rapid insertion reaction occurring even at low temperatures, we were unable to structurally characterize **2′**. However, based on these results we hypothesized, that the insertion/migration reaction from **2′** to **2** occurs so rapidly to reduce the considerable steric congestion at the silicon center and that reducing the size of the aryl substituent could enable us to isolate the intermediate silyliumylidene complex. Consequently, we utilized **1 b** 8k and performed the same reaction (Scheme 3). Indeed, ^29^Si NMR analysis of the resulting red–orange solution showed a resonance at −20.5 ppm, considerably upfield shifted compared to **2** (17.6 ppm) and very close to the −21.1 ppm for **2′**. However, **3** decomposes incredibly quickly at room temperature (even faster than **2**) and slowly at −35 °C, preventing further characterization and analysis (especially through SC‐XRD). Hence, to stabilize the desired complex, we also attempted the reaction with [RhCl_2_(Cp*)]_2_, which proceeds instantly even at −40 °C. ^29^Si NMR analysis of the deep red solution showed a resonance at −24.2 ppm (d, ^1^ *J*_Si−Rh_=66.9 Hz), indicating the formation of the desired complex **4**. While **4** is somewhat more stable in solution than **3**, it still decomposes rapidly (for details concerning the decomposition, see Supporting Information). Still, we were able to obtain single crystals of **4** through quick diffusion of Et_2_O into a MeCN solution at −35 °C. The solid‐state structure (Figure 3) revealed a silyliumylidene complex with a geometry comparable to the chlorosilylene complex **3**, except that in **4** both NHCs are still located on the silicon center and both chlorides are still bound to the metal. The compound features a long Rh1−Si1 bond length of 2.426(2) Å with typical Si−C_NHC_ bond lengths (1.958(7) and 1.944(7) Å). The angle between the coordinated NHCs (93.9(3)°) is comparable to uncoordinated silyliumylidene ions (e.g. **1 a**: 94.3(1)°).12e

Attempts to convert **4** into the chlorosilylene complex analogous to **2** through prolonged stirring failed due to the low stability of **4** in solution. No conversion could be detected after 12 hours at −35 °C and at higher temperatures only decomposition products were observed.

### Reactivity of silyl‐substituted silyliumylidene ions

**Scheme 4:**
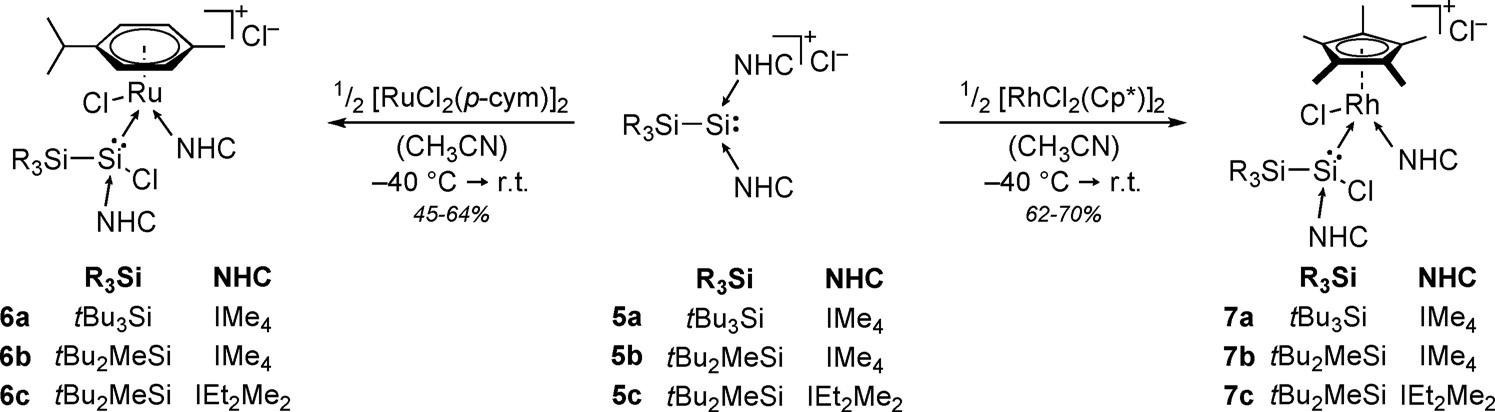
Synthesis of **6** and **7** from **5** (IEt_2_Me_2_=1,3‐diethyl‐4,5‐dimethylimidazol‐2‐ylidene).

Silyl groups have proven to be excellent substituents for the stabilization of elusive main group species because of their tuneable steric demand as well as their strong σ‐electron‐donating properties.^19^ Consequently, we attempted the same conversions with our recently reported silyl‐substituted silyliumylidenes8k **5** in the hope of furnishing analogous silyliumylidene or chlorosilylene complexes with increased stability in solution to allow further functionalization. Reaction of **5** with [RuCl_2_(*p*‐cym)]_2_ and [RhCp*Cl_2_]_2_ (Scheme 4) furnished the orange to red chlorosilylene complexes **6** and **7**, respectively. Only **5 c** did not react in a clean fashion with [RhCp*Cl_2_]_2_, giving a mixture of products containing the desired complex with less than 40 % (cf. Supporting Information Figure S56). Purification attempts were not successful. This can presumably be attributed to the increased steric demand of the bulkier NHCs together with the Cp* ligand, thus favouring side reactions. ^29^Si NMR analysis of **6**–**7** (see Table 1) revealed resonances close to **2**, clearly indicating the formation of the analogous chlorosilylene complexes. Furthermore, ^1^H and ^13^C NMR spectra show formation of asymmetric species with clear signal sets for NHCs bound to both silicon and the metal. Generally, reactions with the rhodium precursor give higher yields than the analogous ruthenium reactions due to higher stability of the Rh complexes in solution. While complexes **6** still slowly decompose at room temperature in solution (**6 a** being the most stable of all ruthenium complexes with full decomposition after roughly 12 hours), **7 a** and **7 b** are stable for at least two weeks.

**Table 1:** Comparison of ^29^Si NMR shifts (CD_3_CN, Central Silicon) and XRD data of Si^II^ cations **1** and **5** and complexes **2**–**4** and **6**–**10**.^[a]^

	R	M	^29^Si NMR [ppm]	Si−M [Å]	M−aryl_⊥_ [Å]
**1 a**	Tipp		−69.58g		
**1 b**	Mes		−71.28k		
**5 a**	*t*Bu_3_Si		−82.08k		
**5 b**	*t*Bu_2_MeSi		−90.78k		
**5 c**	*t*Bu_2_MeSi^[b]^		−86.28k		
**3**	Mes	Ru	−20.5		
**4**	Mes	Rh	−24.2	2.426(2)	1.857(1)
**2**	Tipp	Ru	+17.6	2.409(1)	1.770(1)
**6 a**	*t*Bu_3_Si	Ru	+29.4	2.499(1)	1.767(1)
**6 b**	*t*Bu_2_MeSi	Ru	+29.4		
**6 c**	*t*Bu_2_MeSi^[b]^	Ru	+23.5		
**7 a**	*t*Bu_3_Si	Rh	+23.5	2.423(2)	1.896(1)
**7 b**	*t*Bu_2_MeSi	Rh	+23.9	2.384(1)	1.890(1)
**7 c**	*t*Bu_2_MeSi^[b]^	Rh	+18.6		
**8**	*t*Bu_3_Si	Ru		2.374(1)	1.756(1)
**9**	*t*Bu_3_Si	Rh		2.328(1)/2.331(1)	1.911(1)/1.909(1)
**10**	*t*Bu_3_Si	Ru	+240.6^[c]^	2.236(1)	1.751(1)

[a] Ordered according to structural relationship. [b] NHC=IEt_2_Me_2_. [c] C_6_D_6_.

To further elucidate and strengthen our proposed reaction mechanism, we used the *t*Bu_3_Si‐substituted silyliumylidene triflate **5 a‐OTf** (instead of chloride) and carried out the same reaction: the corresponding complex **7 a‐OTf** could be obtained (cf. Supporting Inforamtion, Figures S46–S48), excluding any relevant involvement of the anion in the reaction mechanism. This reactivity also further underscores the hypothesis that the Si^II^ cation indeed inserts into the M−Cl bond.

SC‐XRD analysis of complexes **6 a**, **7 a** and **7 b** (for details, see Supporting Information Figure S82‐S84) revealed the same general structural motif present in **2**. The Si−M bonds (**2**: 2.409(1) Å, **6 a**: 2.499(1) Å, **7 a**: 2.423(2) Å, **7 b**: 2.384(1) Å) in all complexes are quite long. Interestingly, **2** exhibits a shorter bond length (*Δ*=0.09 Å, 3.6 %) than **6 a**. This trend in bond lengths can also be observed in the related silyl‐ and aryl‐substituted hydrosilylene iron complexes (e.g. Si‐Fe distance in *Aryl*(H)Si(NHC)→Fe(CO)_4_ (2.3268(6) Å^20^) is shorter than in *Silyl*(H)Si(NHC)→Fe(CO)_4_ (2.3717(16) Å^21^)). The distance between the metal and the centroid of the aryl ligand M‐aryl_⊥_ are statistically identical in **2** and **6 a** (1.770(1) Å vs. 1.767(1) Å).

Si1−Cl1 bonds are essentially identical in all complexes, with Si1−C_NHC_ bonds being slightly longer for the *t*Bu_3_Si substituted complexes. The two chloride substituents are oriented almost completely opposite to each other in **2** (dihedral angle Cl−Si−M−Cl: −173.1(1)°, while they exhibit a slightly more staggered position in **6 a** and **7 a** (−156.1(1)° and −154.8(1)°, respectively). A similar trend can be observed for the dihedral angle between the two NHC ligands: **2** shows a C_NHC_−Si−M−C_NHC_ dihedral angle of −11.1(2)°, whereas narrowing of this angle can be observed for **6 a** and **7 a** (−0.2(2)° and −4.2(3), respectively). The angle between the calculated planes of the two NHC scaffolds in the *t*Bu_3_Si‐substituted complexes (**6 a**: 25.0°, **7 a**: 32.5°) are significantly smaller compared to the Tipp‐substituted complex (**2**: 45.8°), meaning they are oriented in a more parallel fashion.

### Access to Si−M single and Si=M double bonds

**Scheme 5:**
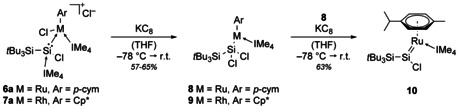
Reduction of **6 a** and **7 a** with KC_8_ to silyl complexes **8** and **9** and to silylene complex **10**.

**Figure 4:**
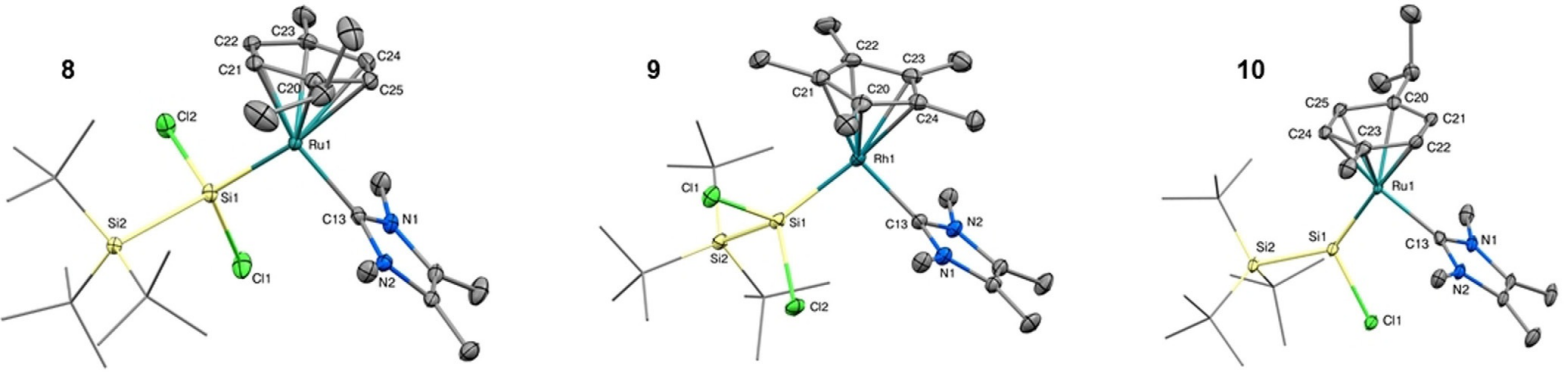
Ellipsoid plot (50 %) of the molecular structures of **8** (left), **9** (middle, one out of two independent molecules in the asymmetric unit shown) and **10** (right). Hydrogen atoms are omitted and the *t*Bu substituents are simplified as wireframes for clarity. Selected bond lengths [Å] and angles [°]: **8**: Si1−Ru1 2.374(1), Si1−Cl1 2.161(1), Si1−Cl2 2.160(1), Si1−Si2 2.424(1), Ru1−C13 2.064(2), Ru1−*p*‐cym_⊥_ 1.756(1), Si2−Si1−Ru1 128.0(1), Si1−Ru1−*p*‐cym_⊥_ 134.0(1); **9**: Si1−Rh1 2.328(1)/2.331(1), Si1−Cl1 2.145(1)/2.146(1), Si1−Cl2 2.170(1)/2.171(1), Si1−Si2 2.430(1)/2.429(1), Rh1−C13 2.033(3)/2.018(3), Rh1−Cp*_⊥_ 1.911(1)/1.909(1), Si2−Si1−Rh1 126.6(1)/126.0(4), Si1−Rh1−Cp*_⊥_ 135.7(1)/136.1(1); **10**: Si1−Ru1 2.236(1), Si1−Cl1 2.169(1), Si1−Si2 2.416(1), Ru1−C13 2.055(4), Ru1−*p*‐cym_⊥_ 1.751(1), Si2−Si1−Ru1 143.8(1), Si1−Ru1−*p*‐cym_⊥_ 147.7(1).

As complexes **2**, **6** and **7** exhibit a halide counterion and one halide bound to the silicon and transition metal each, we thought them to be ideal precursors for the synthesis of Si−Ru and Si−Rh multiple bonds through reductive dehalogenation. We utilized the *t*Bu_3_Si‐substituted complexes **6 a** and **7 a** for further investigations due to their significantly increased stability in solution. After treatment of **6 a** and **7 a** with one equivalent of potassium graphite (Scheme 5), we were able to isolate the unexpected paramagnetic silyl‐substituted complexes **8** (bright green) and **9** (grey‐black) in moderate and good yield, respectively. EPR analysis of **8** and **9** revealed only a single band in both cases (cf. Supporting Information, Figures S64 and S67). No hyperfine coupling to α‐ or β‐silicon could be observed. The *g*‐values (**8**: *g=*2.1062, **9**: *g=*2.1003) are in line with other paramagnetic ruthenium and rhodium complexes.^22^ We successfully confirmed the composition of **8** and **9** through SC‐XRD analysis (Figure 4, left and center). Formation of these complexes most likely takes place through 1,2‐migration of the metal‐bound chloride to silicon under dissociation of the silicon‐bound NHC. As expected, the chloride counterion was the first halide to be removed through reductive dehalogenation.

The Si1−Ru1 bond length in **8** (2.374(1) Å) is shortened significantly (*Δ*=0.125 Å, 5.0 %) in comparison to **6 a**, which is consistent with a reduction of the complex and an increase in the bond order of the Si−Ru bond. Similarly, the Si1−Rh1 bond length in **9** (2.328(1)/2.331(1) Å) is also reduced (*Δ*=0.094 Å (average), 3.9 %) in comparison to **7 a**. Interestingly, the Ru−*p*‐cym_⊥_ distance in **8** (1.756(1) Å) is slightly shorter than in **6 a** (1.767(1) Å), whereas the Rh−Cp*_⊥_ distance is slightly increased from 1.895(1) Å in **7 a** to 1.911(1)/1.909(1) Å in **9**.

We further attempted the reaction of **6 a** with two equivalents of KC_8_ in the hopes of furnishing a Si=Ru bond. Indeed, two‐electron reduction of **6 a** or additional reduction of **8** with 1 KC_8_ yielded the ruthenium silylene complex **10** (Scheme 5, 63 % from **8**, 41 % from **6 a**). During the reaction, an intense color change from bright green (**8**) to deep red (**10**) can be easily observed. We also attempted the reduction of complex **7 a** (or **9**; color change from black to purple) to a similar Si=Rh species. While ^29^Si NMR and mass spectrometry analysis (for details, see Supporting Information) suggest that formation of the analogous complex takes place (albeit in a significantly less clean fashion), we have been unable to obtain satisfactory analytical data so far.

With the additional reductive step, **10** is no longer paramagnetic. The ^29^Si NMR exhibits a significantly downfield shifted resonance at 240.6 ppm, which falls in the expected range of Si=M bonds with a three coordinate Si center^23^ and indicates the multiple‐bond character of the Si=Ru bond. The observed resonance is even more downfield shifted than the previously reported structurally related aryl‐chlorosilylene complexes Cp*(R_3_P)(H)Ru=SiCl(aryl) (aryl=Tipp (221.7 ppm), *m*‐terphenyl (205.0 ppm)).23a No signal splitting analogous to complexes **6** and **7** could be observed in the ^1^H/^13^C NMR spectra. The carbene carbon atom of the metal‐bound NHC also exhibits a significantly more downfield shifted resonance at 188.5 ppm compared to the 172.1 ppm observed for **6 a**.

SC‐XRD analysis of **10** (Figure 4, right) revealed the expected structure with only one chloride atom bound to the silicon center. The silylene silicon adopts a trigonal planar coordination sphere (sum of angles around Si1: 359.4°). Again, a significant shortening (*Δ*=0.138 Å, 5.8 %) of the Si−Ru bond takes place from 2.374(1) Å (**8**) to 2.236(1) Å (**10**) (cf. *Δ*=0.263 Å, 10.5 % from **6 a**), further indicating double‐bond character. In fact, the Si=Ru bond length is easily in the range of other Si=Ru double bonds (2.1823a–2.34 Å^18^, ^24^).

### Computational studies

**Figure 5:**
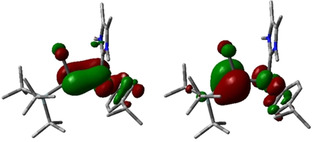
Calculated frontier orbitals of Si=Ru complex **10**: HOMO (left, −3.52 eV) and LUMO (right, −1.43 eV).

To better understand the bonding situation and the electronic structure of the isolated complexes, we also carried out DFT calculations (for details, see Supporting Information). The calculated metric parameters (Table 2, Supporting Information Table S10) show good agreement with the experimentally observed values, indicating the validity of the computational method. Analysis of the Natural Bond Orbitals (NBO, Supporting Information Table S3–S9) revealed that the Si−M bond polarity can change in different complexes: for example, the Si−Ru bond is polarized towards the metal center in complexes **2**, **8**, and **10**. In contrast, the bond is polarized towards the Si atom in **6 a**. Combined with the very long Si‐Ru bond distance in **6 a** (2.499(1) Å), we conclude that the Si−Ru bond in **6 a** is more dative in nature while it exhibits an increased covalent character in the other complexes. Natural Population Analysis (NPA, Table 2 and Supporting Information Table S10) shows that for the aryl‐substituted complexes **2** and **4** the central Si atom bears a more positive charge than in the silyl‐substituted complexes **6 a**, **7 a** and **8**–**10**. This can presumably be attributed to the stronger σ‐donating properties of the silyl moieties compared to aryl groups. In general, the ruthenium center in complexes **2**, **6 a** and **8** exhibits a more negative charge than the Rh atom in **4**, **7 a** and **9**. The Ru center in complex **10** exhibits the highest negative charge (−0.73) out of all complexes. This increased negative charge is most likely the consequence of the double bond character of the Si=Ru bond in **10** suggested by the NBOs (cf. Supporting Information, Table S9). The Wiberg Bond Index (WBI) and Mayer Bond Order (MBO) also support the double bond character, as both WBI and MBO for complex **10** are significantly higher than in the other complexes. These results agree well with the experimentally determined Si−M bond lengths. The calculated frontier orbitals also confirm the validity of the Si=Ru bond in **10** (Figure 5), where the HOMO (highest occupied molecular orbital) and LUMO (lowest unoccupied molecular orbital) correspond to the bonding and anti‐bonding orbital of the Si−Ru π‐bond. Additionally, we were unable to find similar orbitals for the other investigated complexes (cf. Supporting Information, Figure S88–S93), in which the HOMO and LUMO are associated with the metal d orbitals and the π‐system of the Cp* or *p*‐cymene ligands.

**Table 2:** Summary of the calculated Si−M bond lengths, NPA atomic charges and Wiberg bond index (WBI)/Mayer bond order (MBO) of the investigated complexes.

	M	Theor.	NPA atomic charge		WBI	MBO
		Si−M [Å]	Si	M	Si−M	Si−M
**2**	Ru	2.392	+1.31	−0.56	0.73	0.83
**4**	Rh	2.365	+1.23	−0.25	0.64	0.81
**6 a**	Ru	2.481	+0.81	−0.53	0.73	0.72
**7 a**	Rh	2.428	+0.74	−0.21	0.64	0.70
**8**	Ru	2.371	+0.74	−0.46	0.60	0.86
**9**	Rh	2.324	+0.68	−0.21	0.68	0.91
**10**	Ru	2.225	+0.62	−0.73	1.35	1.52

## Conclusions

In summary, we have used NHC‐stabilized Si^II^ cations as a convenient entry point for the isolation of Si→M, Si−M and Si=M moieties via the insertion of silyliumylidenes into M−Cl (M=Ru, Rh) bonds with simultaneous silicon‐to‐metal NHC‐migration, followed by reductive dehalogenation. This work significantly expands the still‐young field of silyliumylidene transition metal coordination chemistry and showcases the ease with which relatively bulky aryl‐ and silyl‐substituted silyliumylidenes insert into M−Cl bonds, forming chlorosilylene transition metal complexes. This is an important distinction to previously reported M−Cl insertion reactions of low‐valent silicon compounds, where the insertion leads to Si^IV^ compounds. The mechanism of formation was investigated theoretically and predicted to include an initially formed silyliumylidene transition metal complex followed by insertion of the Si^II^ cation into the M−Cl bond with concomitant 1,2‐migration of a silicon‐bound NHC moiety to the metal. This could be verified experimentally through NMR and XRD characterization of the silyliumylidene complexes.

The presence of multiple halides on the isolated chlorosilylene complexes gives a simple access route to Si−M single and Si=M double bonds through successive reductive dechlorination. The possible utilization of this synthetic approach to access various transition metal silylidene and silylidyne complexes is currently under investigation in our laboratory.

## Conflict of interest

The authors declare no conflict of interest.

## Supporting information

As a service to our authors and readers, this journal provides supporting information supplied by the authors. Such materials are peer reviewed and may be re‐organized for online delivery, but are not copy‐edited or typeset. Technical support issues arising from supporting information (other than missing files) should be addressed to the authors.

Supplementary
