# Supplementary material for: Facile Access to Dative, Single, and Double Silicon−Metal Bonds Through M−Cl Insertion Reactions of Base‐Stabilized SiII Cations
Source: Chemistry. 2020 Apr 28;26(28):6271–8. doi: 10.1002/chem.202000866 (PMC7318344; doi:10.1002/chem.202000866)
Supplement: Supplementary file 1 — Supplementary [file CHEM-26-6271-s001.pdf]

Chemistry–A European Journal

Supporting Information

Facile Access to Dative, Single, and Double Silicon–Metal Bonds Through M–Cl Insertion Reactions of Base-Stabilized Si^{II} Cations

Philipp Frisch,[a] Tibor Szilvási,[b] and Shigeyoshi Inoue*[a]

Supporting Information

Facile Access to Dative, Single and Double Silicon–Metal Bonds Through M–Cl Insertion Reactions of Base-stabilized Si(II) cations

*Philipp Frisch, Tibor Szilvási and Shigeyoshi Inoue**

Table of Contents

1. Experimental Section S3

1.1 General Methods and Instrumentation S3

1.2 Synthesis of [^{t}Bu$_3$Si–Si(IMe$_4$)$_2$]OTf (**5a-OTf**) S5

1.3 General Synthetic Procedure for [R–Si(NHC)Cl→RuCl(NHC)(*p*-cym)]Cl S9

*1.3.1 [Tipp–Si(IMe$_4$)Cl→RuCl(IMe$_4$)(p-cymene)]Cl (**2**) S10*

*1.3.2 [^{t}Bu$_3$Si–Si(IMe$_4$)Cl→RuCl(IMe$_4$)(p-cymene)]Cl (**6a**) S17*

*1.3.3 [^{t}Bu$_2$MeSi–Si(IMe$_4$)Cl→RuCl(IMe$_4$)(p-cymene)]Cl (**6b**) S22*

*1.3.4 [^{t}Bu$_2$MeSi–Si(IEt$_2$Me$_2$)Cl→RuCl(IEt$_2$Me$_2$)(p-cymene)]Cl (**6c**) S27*

1.4 General Synthetic Procedure for [R–Si(NHC)Cl→RhCl(NHC)(Cp*)]Cl S33

1.4.1 [^{t}Bu$_3$Si–Si(IMe$_4$)Cl→RhCl(IMe$_4$)(Cp)]X (**7a**, X = Cl, OTf) S34*

1.4.2 [^{t}Bu$_2$MeSi–Si(IMe$_4$)Cl→RhCl(IMe$_4$)(Cp)]Cl (**7b**) S42*

1.4.3 [^{t}Bu$_2$MeSi–Si(IEt$_2$Me$_2$)Cl→RhCl(IEt$_2$Me$_2$)(Cp)]Cl (**7c**) S47*

1.5 General Synthetic Procedure for [R–Si(NHC)$_2$→MCl$_2$(Ar)]Cl S49

*1.5.1 [Mes–Si(IMe$_4$)$_2$→RuCl$_2$(p-cymene)]Cl (**3**) S50*

1.5.2 [Mes–Si(IMe$_4$)$_2$→RhCl$_2$(Cp)]X (**4**, X = Cl, [RhCl$_3$Cp*]) S51*

1.6 General Synthetic Procedure for [^{t}Bu$_3$Si–SiCl$_2$–M(IMe$_4$)(Ar)] S56

*1.6.1 [^{t}Bu$_3$Si–SiCl$_2$–Ru(IMe$_4$)(p-cymene)] (**8**) S57*

1.6.2 [^{t}Bu$_3$Si–SiCl$_2$–Rh(IMe$_4$)(Cp)] (**9**) S60*

1.7 Synthesis of [^{t}Bu$_3$Si–Si(Cl)=Ru(IMe$_4$)(*p*-cymene)] (**10**) S62

2. X-ray Crystallographic Data S69

2.1 General Information S69

2.2 SC-XRD structure of **2** (CCDC-1976774) S70

2.3 SC-XRD structure of **4-RhCl$_3$Cp*** (CCDC-1976772) S71

2.4 SC-XRD structure of **6a** (CCDC-1976773) S72

2.5 SC-XRD structure of **7a** (CCDC-1976776) S73

2.6 SC-XRD structure of **7b** (CCDC-1976775) S74

2.7 SC-XRD structure of **8** (CCDC-1976777) S75

2.8 SC-XRD structure of **9** (CCDC-1976778) S76

2.9 SC-XRD structure of **10** (CCDC-1976779) S77

2.10 Crystal data and structural refinement parameters S78

3. DFT Calculations S80

3.1 NBO Analysis of Complexes **2**, **4**, **6a**, **7a**, **8**, **9** and **10** S80

3.2 HOMOs and LUMOs of Complexes **2**, **4**, **6a**, **7a**, **8**, **9** and **10** S83

3.3 Calculated Mechanism for the Formation of **2** *via* **2’** S87

3.4 Cartesian Coordinates of the Calculated Structures S88

4. References S110

1. Experimental Section

1.1 General Methods and Instrumentation

All reactions were carried out under exclusion of water and oxygen in an atmosphere of argon 4.6 (≥99.996%) using standard Schlenk techniques or in a Labstar glovebox from *MBraun* with H_2O and O_2 levels below 0.5 ppm. Glassware was heat dried under vacuum prior to use. Acetonitrile and Acetonitrile-d_3 were refluxed over CaH_2, distilled under argon, deoxygenated by three freeze-pump-thaw cycles and stored over 3 Å molecular sieve in a glovebox. Diethylether, benzene, toluene, THF and *n*-hexane were refluxed over sodium/benzophenone, distilled under argon, deoxygenated by three freeze-pump-thaw cycles and stored over 3 Å molecular sieve in a glovebox. C_6D_6 and THF-d_8 were stirred over Na/K alloy, distilled under argon, deoxygenated by three freeze-pump-thaw cycles and stored over 3 Å molecular sieve in a glovebox. All NMR samples were prepared under argon in *J. Young* PTFE valve NMR tubes. NMR spectra at ambient temperature (300 K) were recorded on a *Bruker* AV500C (^{1}H: 500.36 MHz, ^{13}C: 125.83 MHz, ^{29}Si: 99.41 MHz) spectrometer. Low temperature NMR spectra were recorded on a *Bruker* DRX400 (^{1}H: 400.13 MHz, ^{13}C: 100.62 MHz, ^{29}Si: 79.49 MHz) spectrometer. The ^{1}H, ^{13}C and ^{29}Si NMR spectroscopic chemical shifts δ are reported in ppm relative to tetramethylsilane. ^{1}H and ^{13}C{^{1}H} NMR spectra are calibrated against the residual proton and natural abundance carbon resonances of the respective deuterated solvent as internal standard (CD_3CN: δ(^{1}H) = 1.94 ppm and δ(^{13}C) = 118.3 ppm; THF-d_8: δ(^{1}H) = 1.73 ppm and δ(^{13}C) = 25.4 ppm; C_6D_6: δ(^{1}H) = 7.16 ppm and δ(^{13}C) = 128.1 ppm). ^{29}Si NMR spectra are referenced to the resonance of tetramethylsilane (δ = 0 ppm) as external standard. ^{19}F NMR spectra are referenced to the resonance of $CFCl_3$ (δ = 0 ppm) as external standard. Individual peaks were assigned on the basis of 2D (^{1}H/^{1}H COSY, ^{1}H/^{13}C HSQC, ^{1}H/^{13}C HMBC, ^{1}H/^{29}Si HMBC) NMR experiments. The following abbreviations are used to describe signal multiplicities: s = singlet, d = doublet, t = triplet, q = quartet, sept = septet, m = multiplet. NMR spectra were visualized using MestReNova 12. Spectra recorded in C_6D_6 include a resonance for silicone grease (C_6D_6: δ(^{1}H) = 0.29 ppm, δ(^{13}C) = 1.4 ppm and δ(^{29}Si) = –21.8 ppm) derived from *B. Braun AG Sterican*® cannulas, which is marked with § in the corresponding spectra. Quantitative elemental analyses (EA) were carried out using a *HEKAtech* EURO EA instrument equipped with a CHNS combustion analyzer. LIFDI-MS (Liquid Injection Field Desorption Ionization Mass Spectrometry) spectra were recorded on a *Waters* Micromass LCT TOF mass spectrometer equipped with a LIFDI-ion

source (LIFDI 700) from *Linden CMS GmbH*. UV-Vis spectra were recorded on a *Varian, Inc.* Cary 50 spectrophotometer with a Schlenk quartz cuvette. EPR spectra were recorded on a *Jeol* jes-Fa200 ESR spectrometer with a spectrometer frequency of 9.267 GHz (X-band). Generally, samples were prepared in a glovebox and spectra were visualized using OriginPro 2018. Melting Points (M.P.) were determined in sealed glass capillaries under inert gas by a *Büchi* M-565 melting point apparatus. Unless otherwise stated, all commercially available chemicals were purchased from *abcr* or *Sigma-Aldrich* and used without further purification. The compounds [Tipp–Si(IMe_4)$_2$]Cl (**1a**)[1], [Mes–Si(IMe_4)$_2$]Cl (**1b**)[2], [^{t}Bu$_3$Si–Si(IMe_4)$_2$]Cl (**5a**)[2], [^{t}Bu$_2$MeSi–Si(IMe_4)$_2$]Cl (**5b**)[2], [^{t}Bu$_2$MeSi–Si(IEt_2Me_2)$_2$]Cl (**5c**)[2], [$RuCl_2$(*p*-cymene)]$_2$[3], [$RhCl_2$(C_5Me_5)]$_2$[4] and KC_8[5] were prepared as described in the literature. The compound [^{t}Bu$_3$Si–Si(IMe_4)$_2$]OTf (**5a-OTf**) was synthesized similarly to the previously published procedure for the anion exchange of NHC-stabilized silyliumylidene ions.[6]

1.2 Synthesis of [^{t}Bu$_3$Si–Si(IMe$_4$)$_2$]OTf (5a-OTf)

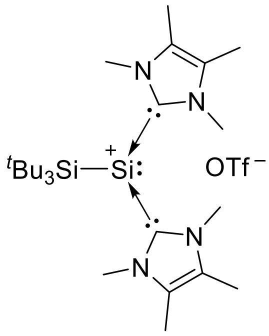

5a-OTf

$C_{27}H_{51}F_3N_4O_3SSi_2$

624.96 g/mol

[^{t}Bu$_3$Si–Si(IMe$_4$)$_2$]Cl (**5a**) (300.0 mg, 586.7 µmol, 1.0 eq) and KOTf (110.4 mg, 586.7 µmol, 1.0 eq) was suspended in 7 mL THF and stirred at room temperature until the starting material dissolved (5-10 minutes). The cloudy, bright orange suspension was concentrated under reduced pressure to 4-5 mL, filtered and the clear solution was further concentrated under reduced pressure to about 2-3 mL where precipitation began. *n*-Hexane (12 mL) was added to precipitate an orange solid which was collected by filtration, washed with *n*-hexane (3 mL) and after drying under vacuum the product **5a-OTf** (352.3 mg, 563.7 µmol, 96%) was obtained as an orange air- and moisture-sensitive solid.

^{1}H NMR (500 MHz, CD$_3$CN, 300 K): δ [ppm] = 3.68 (s, 12H, N$_{NHC}$CH_3), 2.15 (s, 12H, C$_{NHC}$CH_3), 1.22 (s, 27H, C(CH_3)$_3$).

^{1}H NMR (500 MHz, THF-d_8, 300 K): δ [ppm] = 3.85 (s, 12H, N$_{NHC}$CH_3), 2.21 (s, 12H, C$_{NHC}$CH_3), 1.25 (s, 27H, C(CH_3)$_3$).

^{13}C{^{1}H} NMR (126 MHz, CD$_3$CN, 300 K): δ [ppm] = 164.1 (NC_{NHC}N), 128.7 (C_{NHC}CH$_3$), 122.0 (q, $^1J_{C–F}$ = 320.8 Hz, SO$_3$$CF_3$), 36.6 (N$_{NHC}$$CH_3$), 33.1 (C($CH_3$)$_3$), 26.3 ($C$(CH$_3$)$_3$), 9.2 (C$_{NHC}$$CH_3$).

Note: *The resonance for the SO$_3$CF$_3$ group (quartet) partially overlaps with the resonance for CD$_3$CN.*

^{13}C{^{1}H} NMR (126 MHz, THF-d_8, 300 K): δ [ppm] = 162.8 (NC_{NHC}N), 129.0 (C_{NHC}CH$_3$), 122.4 (q, $^1J_{C–F}$ = 322.8 Hz, SO$_3$$CF_3$), 36.6 (N$_{NHC}$$CH_3$), 33.2 (C($CH_3$)$_3$), 26.1 ($C$(CH$_3$)$_3$), 9.4 (C$_{NHC}$$CH_3$).

^{29}Si{^{1}H} NMR (99 MHz, CD$_3$CN, 300 K): δ [ppm] = 21.7 (Si*Si*^{t}Bu$_3$), –82.1 (*Si*Si^{t}Bu$_3$).

^{29}Si{^{1}H} NMR (99 MHz, THF-d_8, 300 K): δ [ppm] = 20.2 (Si*Si*^{t}Bu$_3$), –84.4 (*Si*Si^{t}Bu$_3$).

^{19}F{^{1}H} NMR (471 MHz, CD$_3$CN, 300 K): δ [ppm] = –79.3 (SO$_3$CF_3).

EA: $C_{27}H_{51}N_4Si_2SO_3F_3$ calculated [%]: C (51.89), H (8.23), N (8.97), S (5.13).
measured [%]: C (52.17), H (8.46), N (9.24), S (4.96).

M.P.: 157-158 °C (decomposition, color change to black).

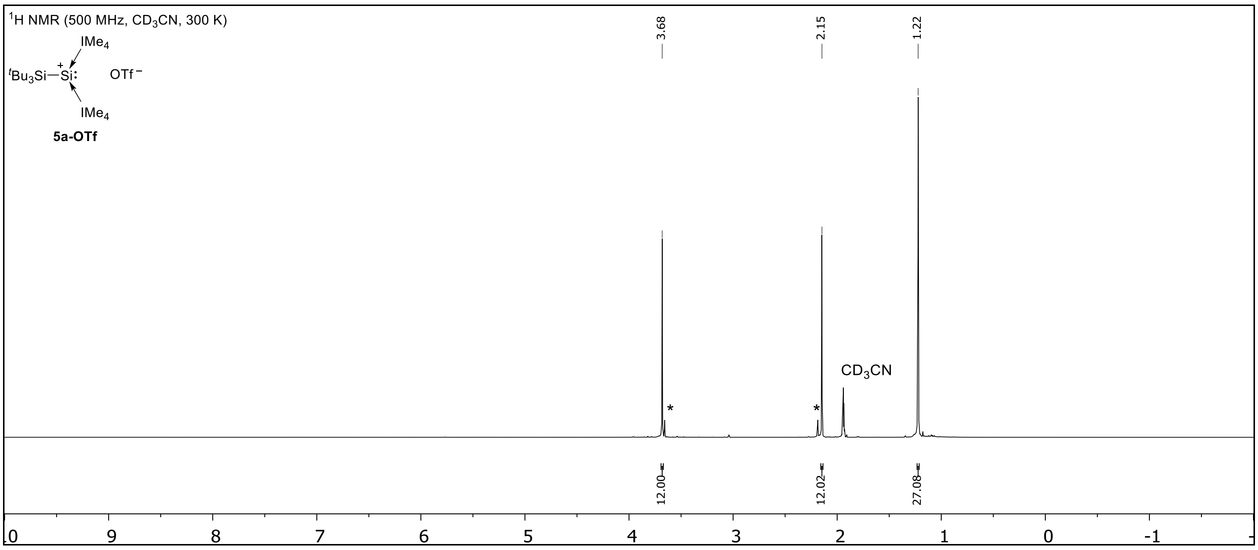

Figure S1 ^{1}H NMR spectrum of [^{t}Bu$_3$Si–Si(IMe$_4$)$_2$]OTf (**5a-OTf**) in CD_3CN at 300 K. Residual imidazolium triflate [IMe$_4$·HOTf] from the precursor synthesis is marked with *.

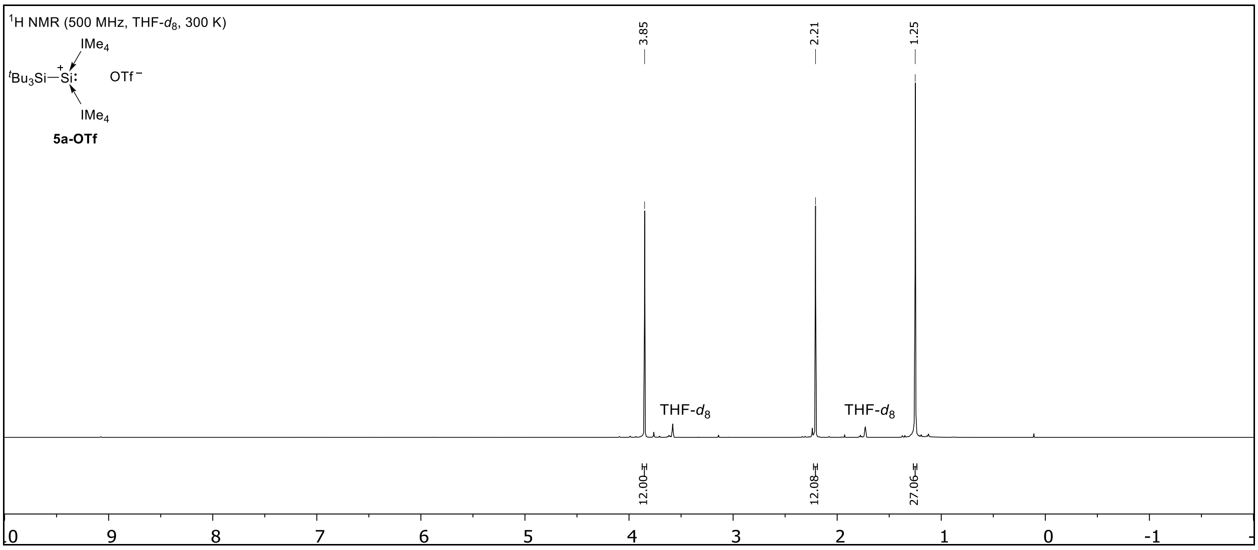

Figure S2 ^{1}H NMR spectrum of [^{t}Bu$_3$Si–Si(IMe$_4$)$_2$]OTf (**5a-OTf**) in THF-d_8 at 300 K.

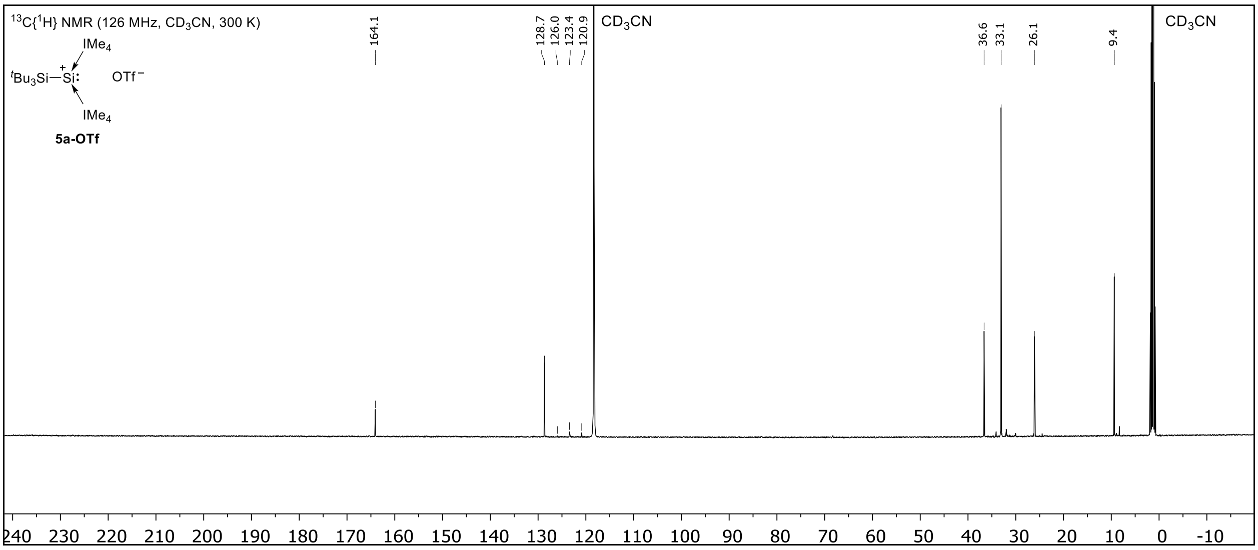

Figure S3 $^{13}C\{^1H\}$ NMR spectrum of [^{t}Bu$_3$Si–Si(IMe$_4$)$_2$]OTf (**5a-OTf**) in CD_3CN at 300 K.

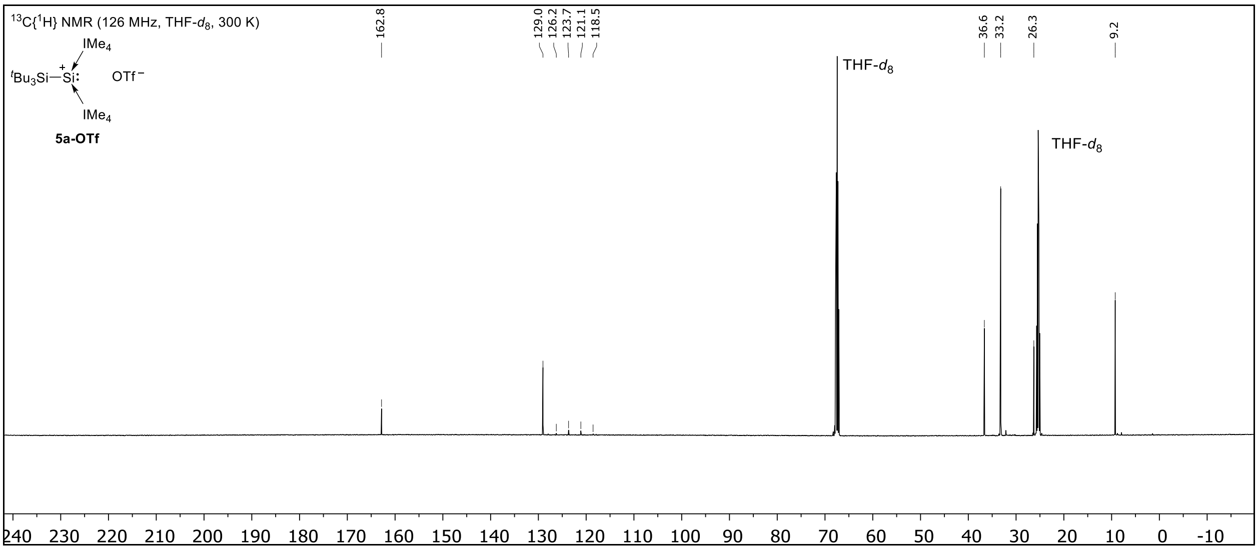

Figure S4 $^{13}C\{^1H\}$ NMR spectrum of [^{t}Bu$_3$Si–Si(IMe$_4$)$_2$]OTf (**5a-OTf**) in THF-d_8 at 300 K.

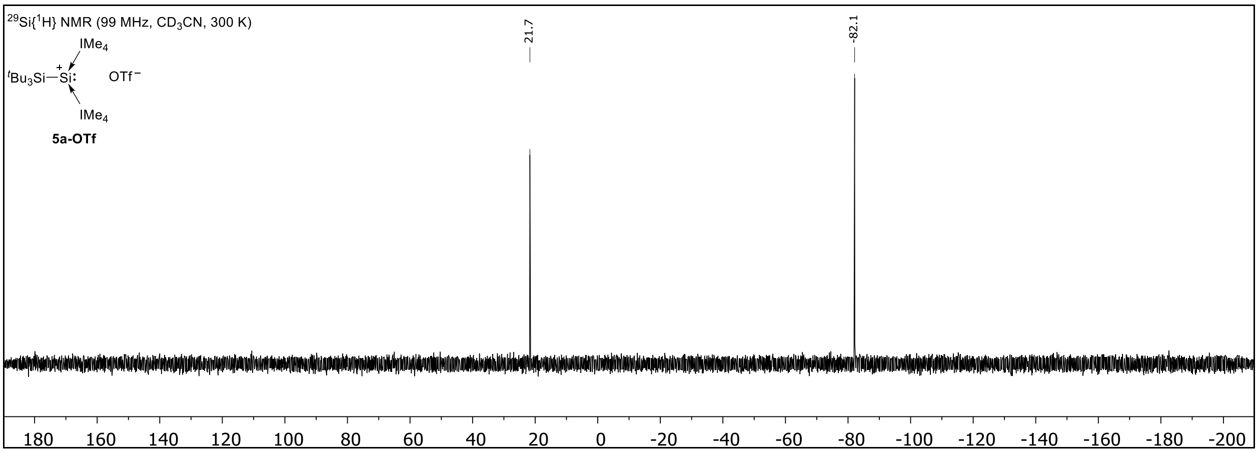

Figure S5 ^{29}Si{^{1}H} NMR spectrum of [^{t}Bu$_3$Si–Si(IMe$_4$)$_2$]OTf (**5a-OTf**) in CD$_3$CN at 300 K.

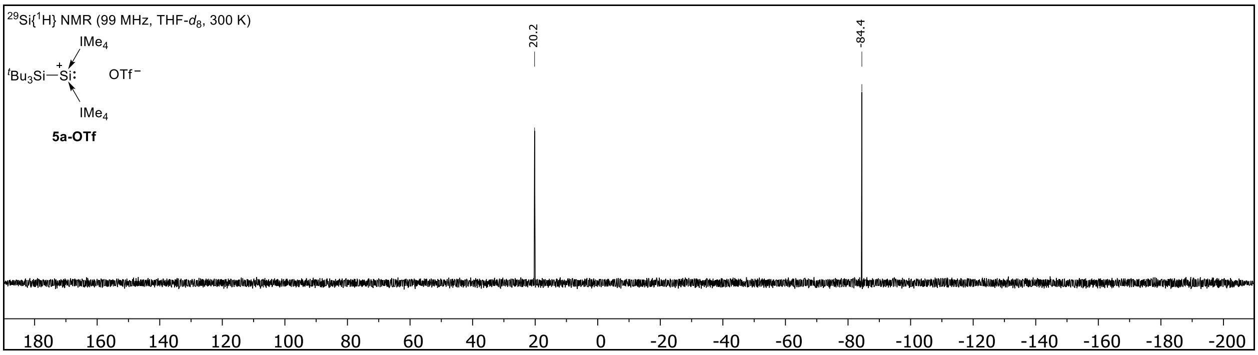

Figure S6 ^{29}Si{^{1}H} NMR spectrum of [^{t}Bu$_3$Si–Si(IMe$_4$)$_2$]OTf (**5a-OTf**) in THF-d_8 at 300 K.

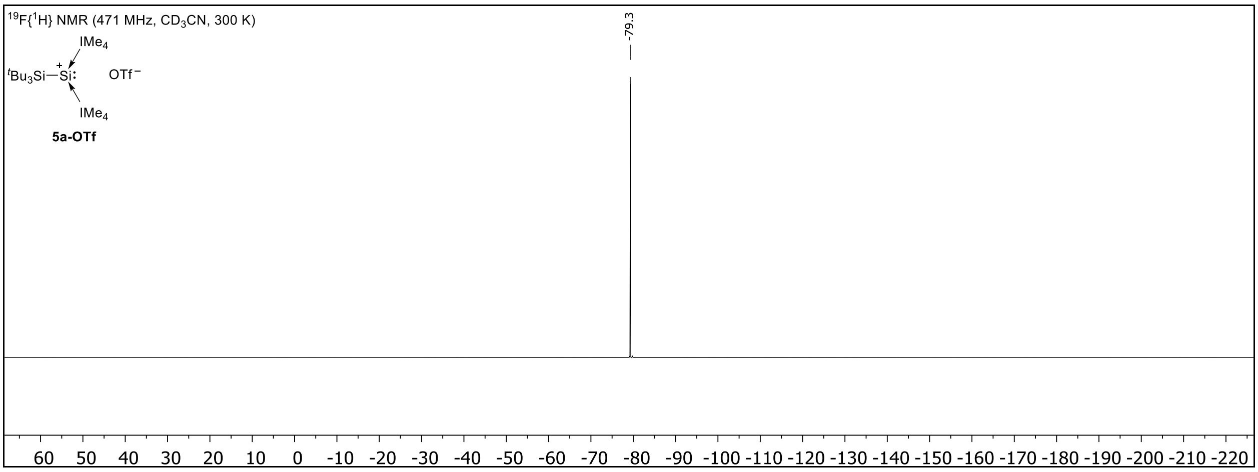

Figure S7 ^{19}F{^{1}H} NMR spectrum of [^{t}Bu$_3$Si–Si(IMe$_4$)$_2$]OTf (**5a-OTf**) in CD$_3$CN at 300 K.

1.3 General Synthetic Procedure for [R–Si(NHC)Cl→RuCl(NHC)(*p*-cym)]Cl

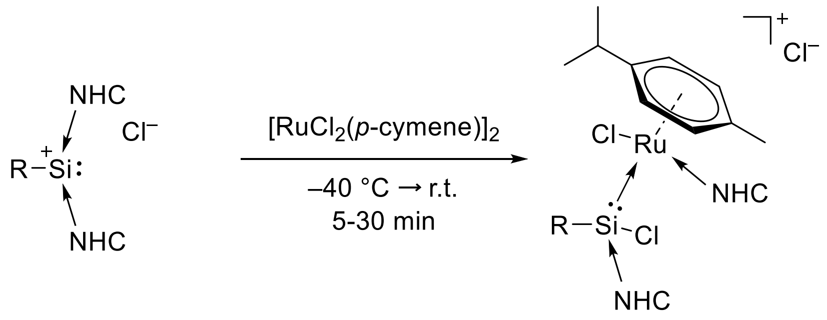

	R	NHC
**1a**	Tipp	IMe_4
**5a**	^{t}Bu$_3$Si	IMe_4
**5b**	^{t}Bu$_2$MeSi	IMe_4
**5c**	^{t}Bu$_2$MeSi	IEt_2Me_2

	R	NHC
**2**	Tipp	IMe_4
**6a**	^{t}Bu$_3$Si	IMe_4
**6b**	^{t}Bu$_2$MeSi	IMe_4
**6c**	^{t}Bu$_2$MeSi	IEt_2Me_2

Silyliumylidene chloride [R–Si(NHC)$_2$]Cl (**1a** or **5a-c**) (1.0 eq) and [RuCl$_2$(*p*-cymene)]$_2$ (0.5 eq) were mixed, cooled to –40 °C and pre-cooled (–40 °C) MeCN (3-10 mL) was added. The reaction mixtures were stirred at –40 °C until all starting material had dissolved (5-30 minutes) and then warmed to room temperature. The solutions were quickly concentrated under reduced pressure to about 1-3 mL. A mixture of toluene and Et_2O (1:1, 5-15 mL) was added and the orange-red solutions were stored at –35 °C for 3-10 days. The formed (microcrystalline) precipitate was collected by filtration, washed with benzene or toluene (2 mL) and Et_2O (2×2 mL) and after drying under vacuum the complexes [R–Si(NHC)Cl→RuCl(NHC)(*p*-cymene)]Cl (**2** and **6a-c**) were isolated as orange air- and moisture-sensitive solids.

1.3.1 [Tipp–Si(IMe$_4$)Cl→RuCl(IMe$_4$)(*p*-cymene)]Cl (2)

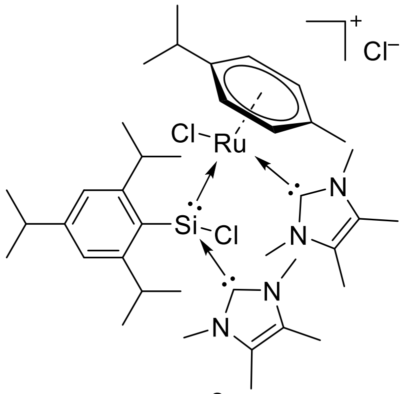

2

$C_{39}H_{61}Cl_3N_4RuSi$

821.45 g/mol

Note:* (a)** *Upon addition of cold MeCN to the starting materials the solution turns deep red. Warming of this solution leads to an almost instantaneous color change to orange at temperatures higher than –20 to –15 °C. We presume that the deep red species (**2’**) is in fact the silyliumylidene transition metal complex before insertion into the M–Cl bond and migration of one coordinated NHC moiety, whereas the orange species is complex **2**. Low-temperature ^{29}Si NMR at –35 °C of **2’** revealed a resonance at –21.1 ppm (cf. Figure S16), which is in line with the –20.5 ppm observed for the silyliumylidene ruthenium complex **3** and corresponds well with the calculated NMR shift for **2’** (–23.4 ppm). Isolation of the silyliumylidene complex in a clean fashion was not possible. The insertion reaction occurs too fast, preventing any kind of work up. Utilization of solvents with a significantly lower melting point did not give the desired product (for example, carrying out the reaction in THF led to complete polymerization of the solvent);* ***(b) *Full decomposition of **2** occurs in solution within roughly 4 hours (cf. Figure S15) at room temperature. No silicon containing species could be identified. **2** is stable for at least 3 days at –35 °C in MeCN solution.*

2' at –40 °C

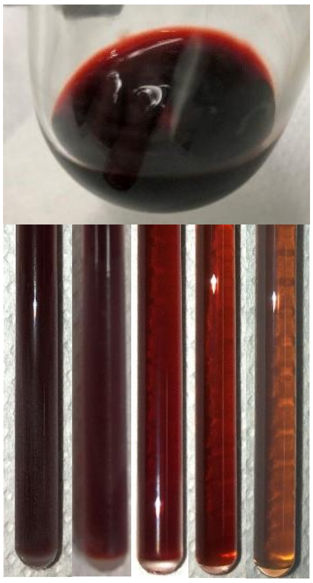

–40 °C ⟶ –15 °C ⟶ r.t.
Color change in <60 sec

Batch size: **1a**: 100.0 mg, 194.1 µmol, 1.0 eq.
[RuCl$_2$(*p*-cymene)]$_2$: 59.4 mg, 97.0 µmol, 0.5 eq.

Yield: 56.6 mg (68.9 µmol, 36%) as an orange solid.

SC-XRD: Suitable crystals were obtained by cooling a concentrated solution of **2** in MeCN to –35 °C.

^{1}H NMR (500 MHz, CD_3CN, 300 K): δ [ppm] = 7.23 (s, 1H, $\mathrm{C_{Tipp}}H_{\mathrm{ar}}$), 6.88 (s, 1H, $\mathrm{C_{Tipp}}H_{\mathrm{ar}}$), 5.84 (d, $^{3}J_{\mathrm{H–H}}$ = 6.1 Hz, 1H, $\mathrm{C}_{p\text{-cym}}H_{\mathrm{ar}}$), 5.77 (d, $^{3}J_{\mathrm{H–H}}$ = 5.9 Hz, 1H, $\mathrm{C}_{p\text{-cym}}H_{\mathrm{ar}}$), 5.69 (m, 2H, $\mathrm{C}_{p\text{-cym}}H_{\mathrm{ar}}$), 4.14 (sept, $^{3}J_{\mathrm{H–H}}$ = 5.9 Hz, 1H, $\mathrm{C_{Tipp}}H\mathrm{(CH_3)_2}$), 4.07 (s, 3H, $\mathrm{N_{NHC\rightarrow Ru}C}H_3$), 3.27 (s, 3H, $\mathrm{N_{NHC\rightarrow Si}C}H_3$), 3.04 (s, 3H, $\mathrm{N_{NHC\rightarrow Si}C}H_3$), 2.89 (s, 3H, $\mathrm{N_{NHC\rightarrow Si}C}H_3$), 2.84 (sept, $^{3}J_{\mathrm{H–H}}$ = 6.9 Hz, 1H, $\mathrm{C}_{p\text{-cym}}H\mathrm{(CH_3)_2}$), 2.60 (sept, $^{3}J_{\mathrm{H–H}}$ = 6.3 Hz, 1H, $\mathrm{C_{Tipp}}H\mathrm{(CH_3)_2}$), 2.47 (sept, $^{3}J_{\mathrm{H–H}}$ = 6.4 Hz, 1H, $\mathrm{C_{Tipp}}H\mathrm{(CH_3)_2}$), 2.24 (s, 3H, $\mathrm{C_{NHC\rightarrow Ru}C}H_3$), 2.12 (s, 3H, $\mathrm{C_{NHC\rightarrow Ru}C}H_3$), 2.02 (s, 3H, $\mathrm{C_{NHC\rightarrow Si}C}H_3$), 2.00 (s, 3H, $\mathrm{C_{NHC\rightarrow Si}C}H_3$), 1.75 (s, 3H, $\mathrm{C}_{p\text{-cym}}\mathrm{C}H_3$), 1.58 (d, $^{3}J_{\mathrm{H–H}}$ = 5.9 Hz, 3H, $\mathrm{C_{Tipp}H(C}H_3\mathrm{)_2}$), 1.49 (d, $^{3}J_{\mathrm{H–H}}$ = 5.9 Hz, 3H, $\mathrm{C_{Tipp}H(C}H_3\mathrm{)_2}$), 1.21 (d, $^{3}J_{\mathrm{H–H}}$ = 6.4 Hz, 3H, $\mathrm{C_{Tipp}H(C}H_3\mathrm{)_2}$), 1.21 – 1.19 (m, 6H, $\mathrm{C}_{p\text{-cym}}\mathrm{H(C}H_3\mathrm{)_2}$), 0.93 (d, $^{3}J_{\mathrm{H–H}}$ = 6.3 Hz, 3H, $\mathrm{C_{Tipp}H(C}H_3\mathrm{)_2}$), 0.74 (d, $^{3}J_{\mathrm{H–H}}$ = 6.4 Hz, 3H, $\mathrm{C_{Tipp}H(C}H_3\mathrm{)_2}$), 0.05 (d, $^{3}J_{\mathrm{H–H}}$ = 6.3 Hz, 3H, $\mathrm{C_{Tipp}H(C}H_3\mathrm{)_2}$).

^{13}C{^{1}H} NMR (126 MHz, CD_3CN, 300 K): δ [ppm] = 169.9 ($\mathrm{N}C_{\mathrm{NHC\rightarrow Ru}}\mathrm{N}$), 155.9 ($C_{\mathrm{Tipp}}$–^{i}Pr), 155.7 ($C_{\mathrm{Tipp}}$–^{i}Pr), 154.8 ($\mathrm{N}C_{\mathrm{NHC\rightarrow Si}}\mathrm{N}$), 151.2 ($C_{\mathrm{Tipp}}$–^{i}Pr), 137.9 ($C_{\mathrm{Tipp}}$–Si), 130.0 ($C_{\mathrm{NHC\rightarrow Ru}}\mathrm{CH_3}$), 129.8 ($C_{\mathrm{NHC\rightarrow Si}}\mathrm{CH_3}$), 128.9 ($C_{\mathrm{NHC\rightarrow Si}}\mathrm{CH_3}$), 127.5 ($C_{\mathrm{NHC\rightarrow Ru}}\mathrm{CH_3}$), 123.9 ($C_{\mathrm{Tipp}}\mathrm{H_{ar}}$), 122.6 ($C_{\mathrm{Tipp}}\mathrm{H_{ar}}$), 96.5 ($C_{p\text{-cym}}\mathrm{H_{ar}}$), 94.6 ($C_{p\text{-cym}}\mathrm{H_{ar}}$), 93.7 ($C_{p\text{-cym}}\mathrm{H_{ar}}$), 85.0 ($C_{p\text{-cym}}\mathrm{H_{ar}}$), 37.4 ($\mathrm{N_{NHC\rightarrow Ru}}C\mathrm{H_3}$), 37.0 ($\mathrm{N_{NHC\rightarrow Ru}}C\mathrm{H_3}$), 36.7 ($C_{\mathrm{Tipp}}\mathrm{H(CH_3)_2}$), 35.7 ($\mathrm{N_{NHC\rightarrow Si}}C\mathrm{H_3}$), 34.8 ($C_{\mathrm{Tipp}}\mathrm{H(CH_3)_2}$), 34.7 ($C_{p\text{-cym}}\mathrm{H(CH_3)_2}$), 32.9 ($\mathrm{N_{NHC\rightarrow Si}}C\mathrm{H_3}$), 31.1 ($C_{\mathrm{Tipp}}\mathrm{H(CH_3)_2}$), 28.8 ($\mathrm{C_{Tipp}H(}C\mathrm{H_3)_2}$), 27.0 ($\mathrm{C_{Tipp}H(}C\mathrm{H_3)_2}$), 26.1 ($\mathrm{C_{Tipp}H(}C\mathrm{H_3)_2}$), 24.1 ($\mathrm{C_{Tipp}H(}C\mathrm{H_3)_2}$), 24.1 ($\mathrm{C}_{p\text{-cym}}\mathrm{H(C}H_3\mathrm{)_2}$), 23.9 ($\mathrm{C}_{p\text{-cym}}\mathrm{H(}C\mathrm{H_3)_2}$), 22.1 ($\mathrm{C_{Tipp}H(}C\mathrm{H_3)_2}$), 20.1 ($\mathrm{C_{Tipp}H(}C\mathrm{H_3)_2}$), 18.4 ($\mathrm{C}_{p\text{-cym}}C\mathrm{H_3}$), 10.1 ($\mathrm{C_{NHC\rightarrow Ru}}C\mathrm{H_3}$), 9.7 ($\mathrm{C_{NHC\rightarrow Ru}}C\mathrm{H_3}$), 9.1 ($\mathrm{C_{NHC\rightarrow Si}}C\mathrm{H_3}$), 8.9 ($\mathrm{C_{NHC\rightarrow Si}}C\mathrm{H_3}$).

^{29}Si{^{1}H} NMR (99 MHz, CD_3CN, 300 K): δ [ppm] = 17.8 (Tipp*Si*).

EA: $C_{39}H_{61}Cl_3N_4RuSi$ calculated [%]: C (57.02), H (7.49), N (6.82).
measured [%]: C (56.79), H (7.71), N (7.10).

M.P.: 124-125 °C (decomposition, color change to black).

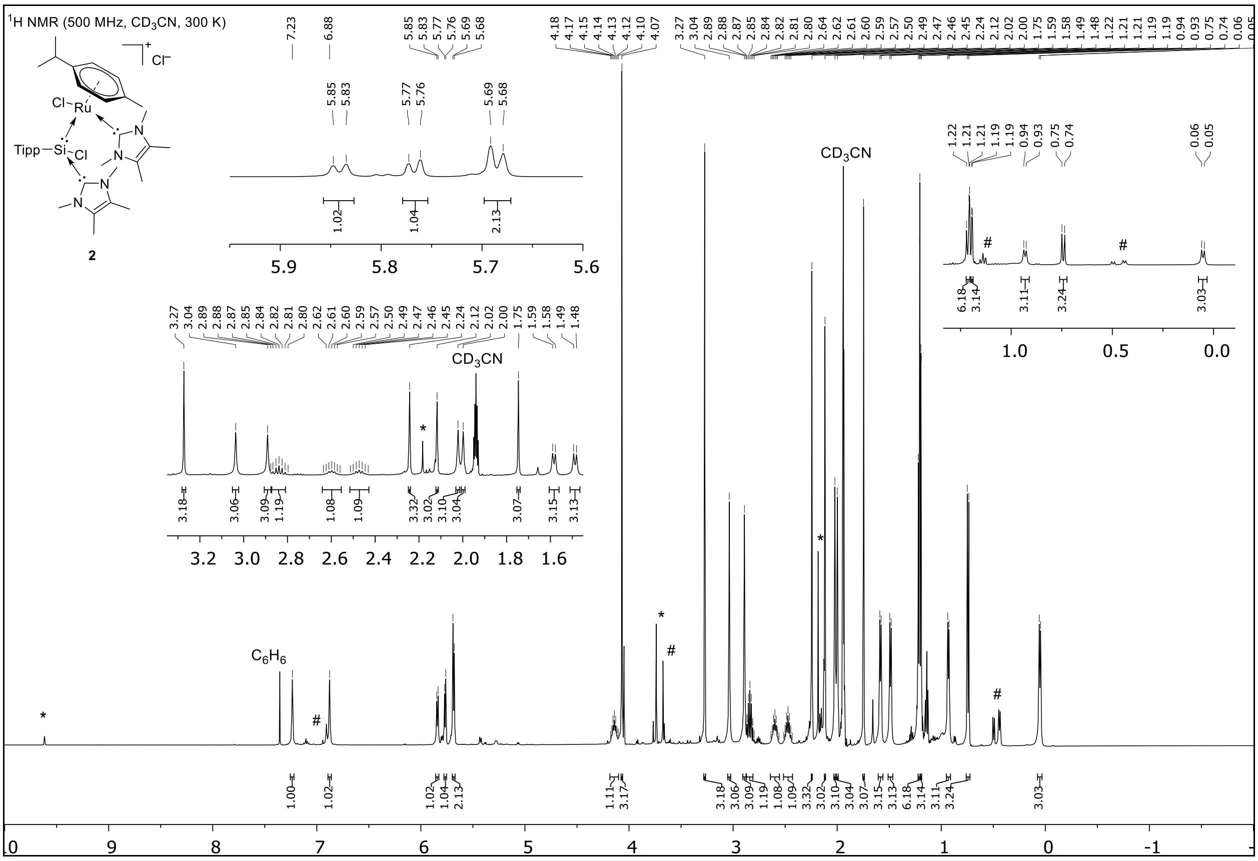

Figure S8 ^{1}H NMR spectrum of [Tipp–Si(IMe$_4$)Cl→RuCl(IMe$_4$)(*p*-cymene)]Cl (**2**) in CD_3CN at 300 K. Residual imidazolium chloride [IMe$_4$·HCl] from the synthesis of **1a** and from the decomposition of **2** is marked with *. Beginning decomposition is marked with #.

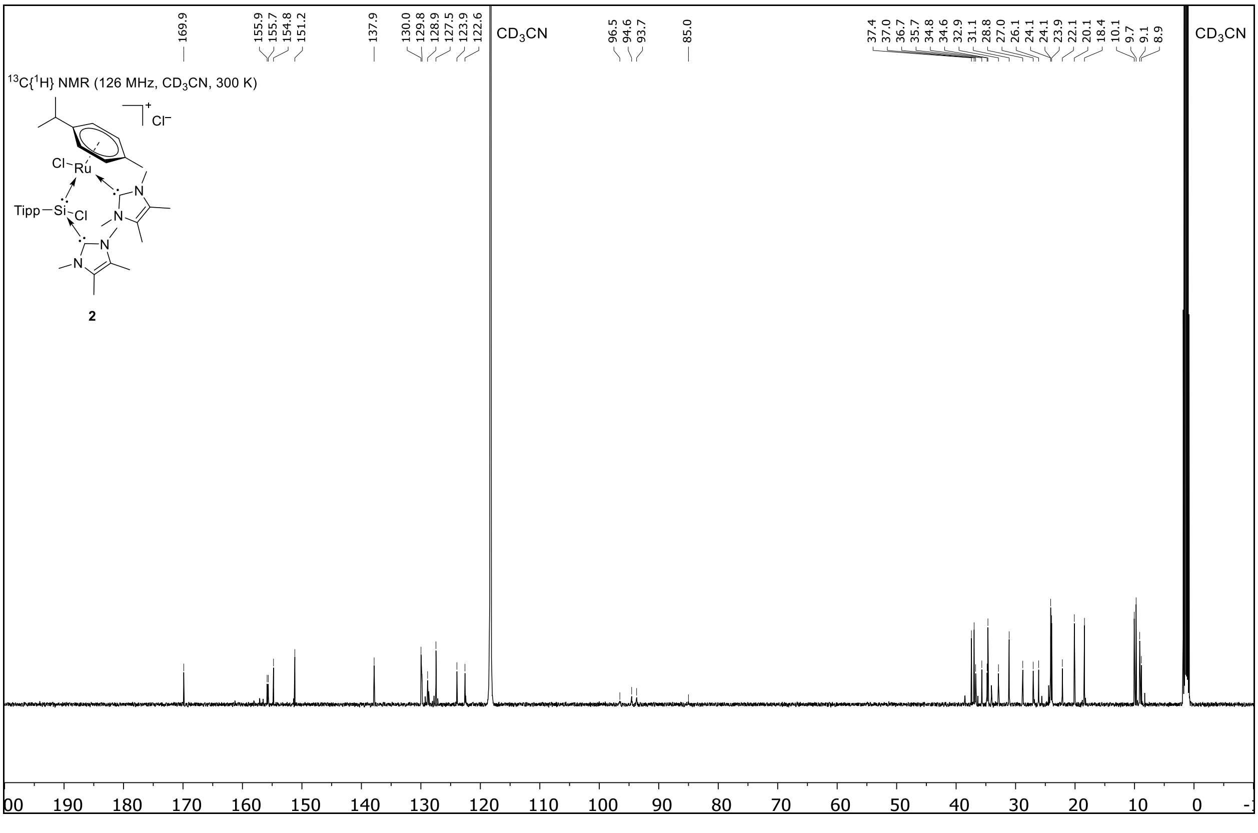

Figure S9 $^{13}C\{^{1}H\}$ NMR spectrum of [Tipp–Si(IMe$_4$)Cl→RuCl(IMe$_4$)(*p*-cymene)]Cl (**2**) in CD_3CN at 300 K.

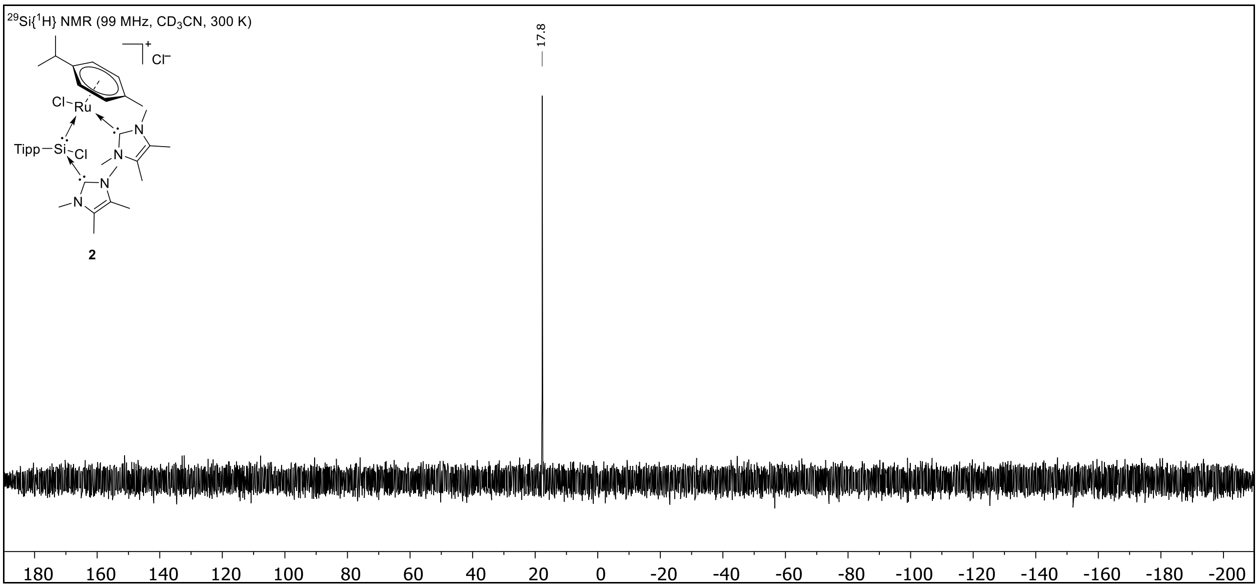

Figure S10 ^{29}Si{^{1}H} NMR spectrum of [Tipp–Si(IMe$_4$)Cl→RuCl(IMe$_4$)(*p*-cymene)]Cl (**2**) in CD_3CN at 300 K.

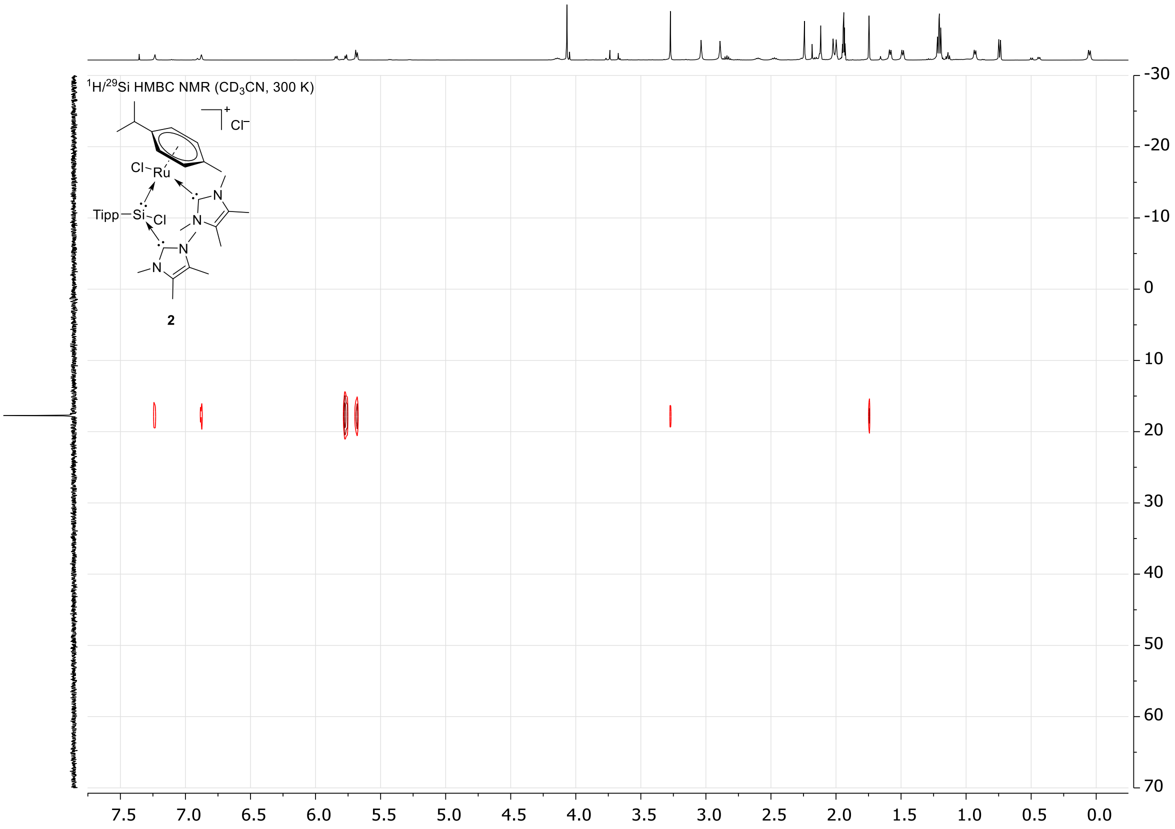

Figure S11 ^{1}H/^{29}Si HMBC NMR spectrum of [Tipp–Si(IMe$_4$)Cl→RuCl(IMe$_4$)(*p*-cymene)]Cl (**2**) in CD_3CN at 300 K.

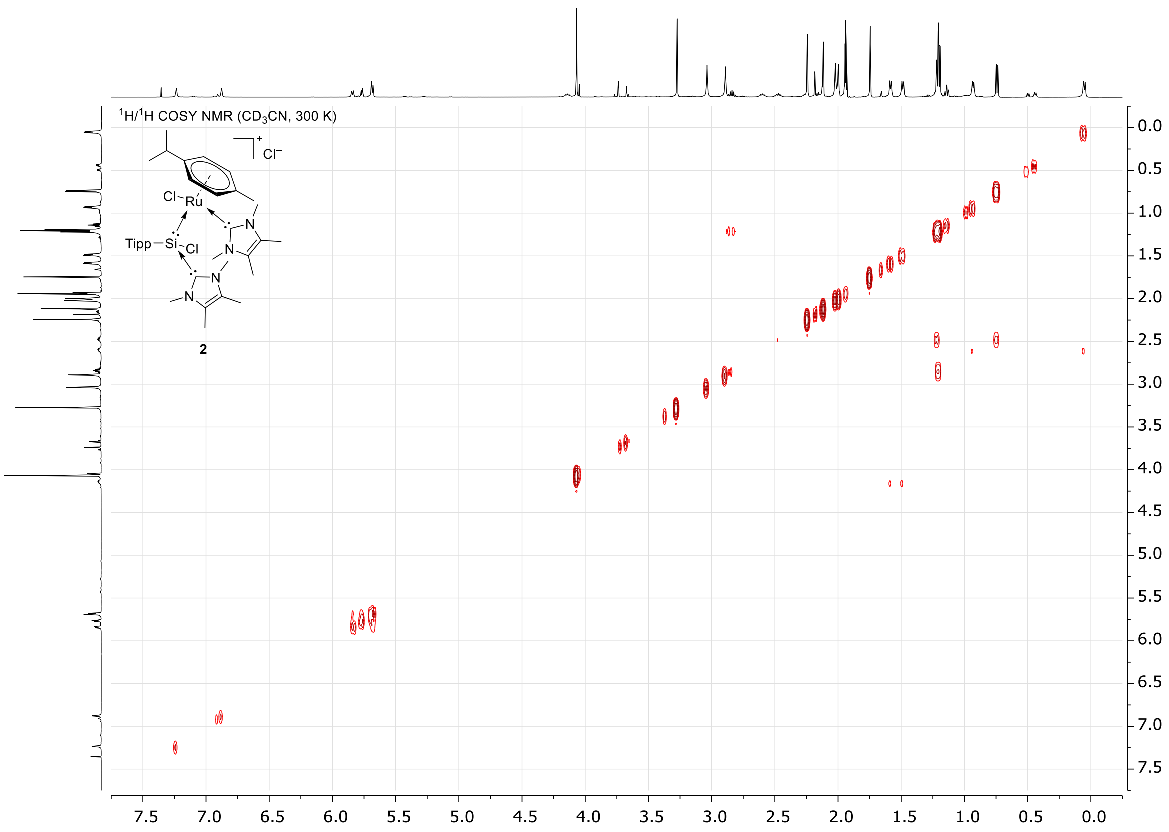

Figure S12 ^{1}H/^{1}H COSY NMR spectrum of [Tipp–Si(IMe$_4$)Cl→RuCl(IMe$_4$)(*p*-cymene)]Cl (**2**) in CD_3CN at 300 K.

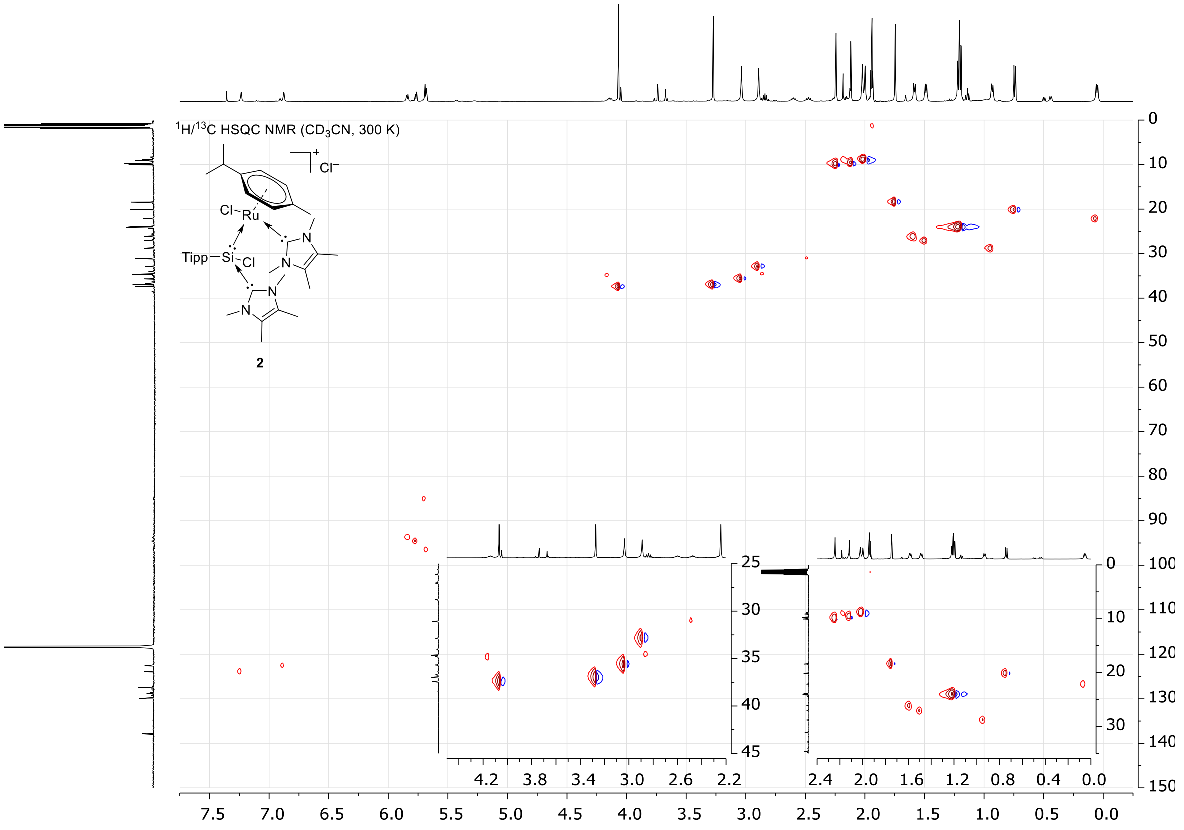

Figure S13 ^{1}H/^{13}C HSQC NMR spectrum of [Tipp–Si(IMe$_4$)Cl→RuCl(IMe$_4$)(*p*-cymene)]Cl (**2**) in CD_3CN at 300 K.

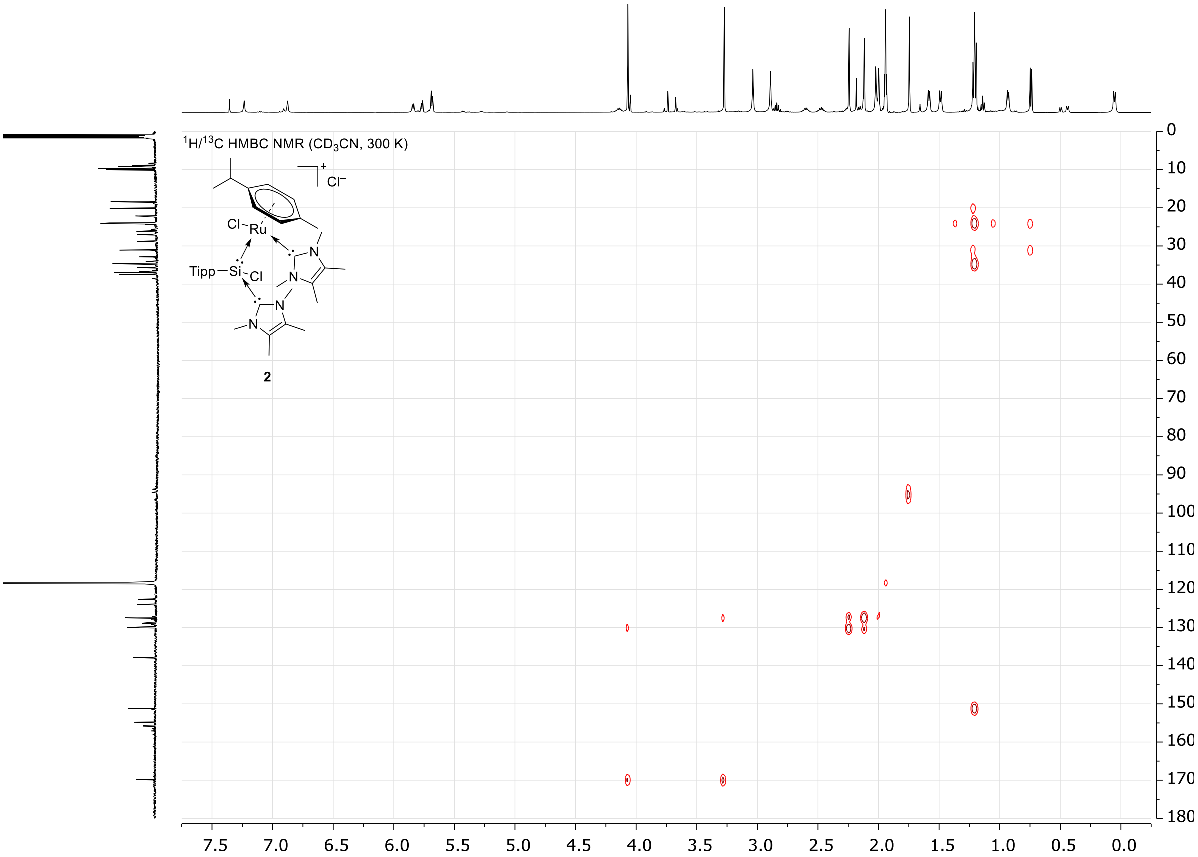

Figure S14 ^{1}H/^{13}C HMBC NMR spectrum of [Tipp–Si(IMe$_4$)Cl→RuCl(IMe$_4$)(*p*-cymene)]Cl (**2**) in CD$_3$CN at 300 K.

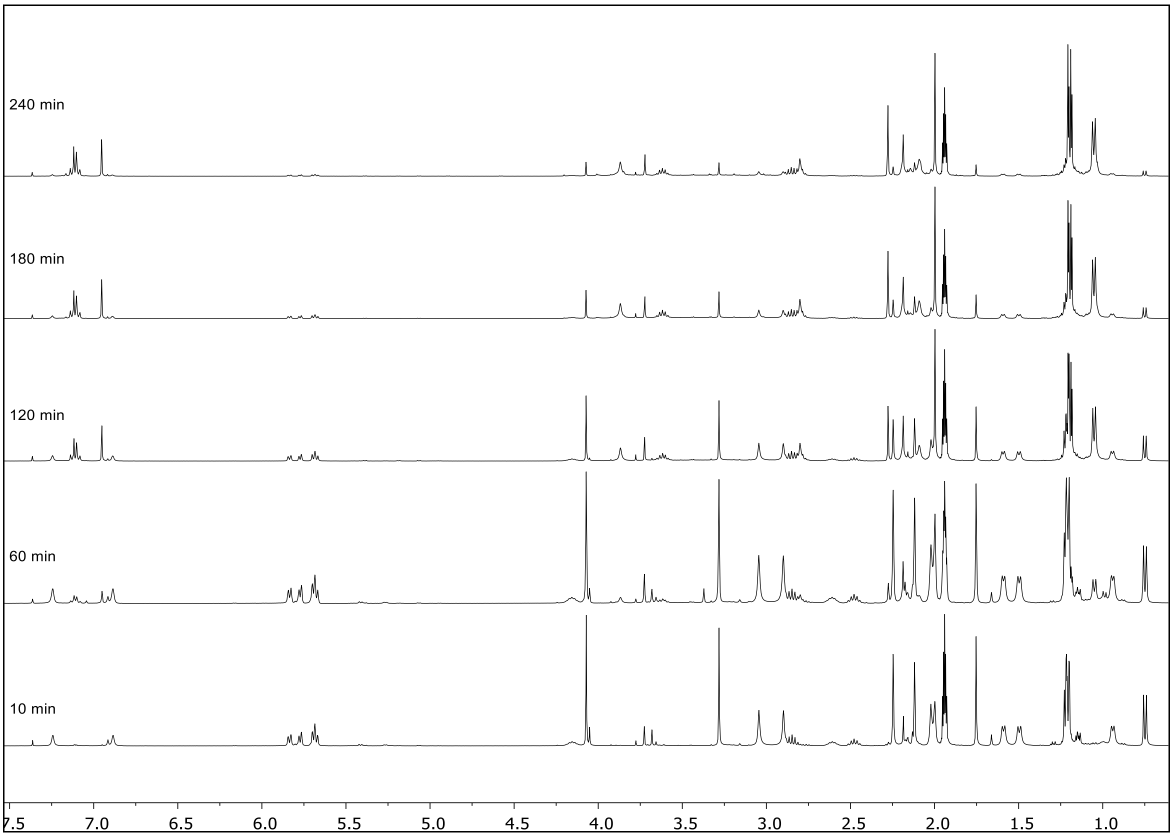

Figure S15 Time resolved ^{1}H NMR spectra of [Tipp–Si(IMe$_4$)Cl→RuCl(IMe$_4$)(*p*-cymene)]Cl (**2**) in CD$_3$CN showing the decomposition in solution at room temperature.

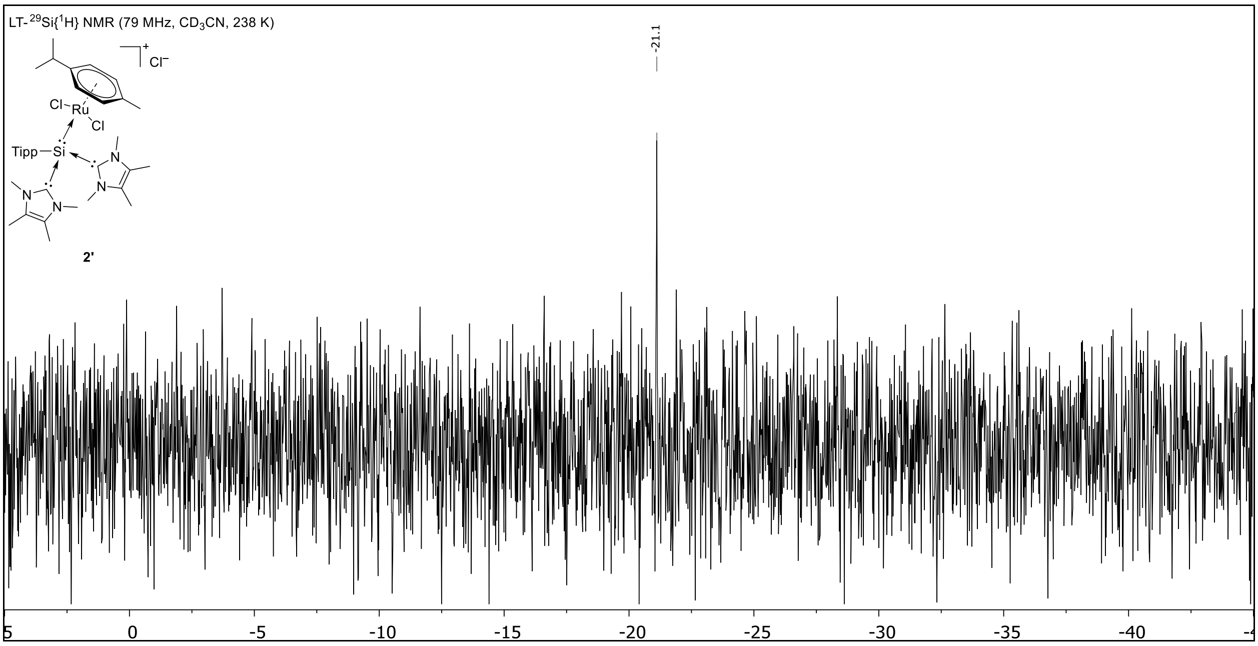

Figure S16 Low temperature $^{29}Si\{^1H\}$ NMR of the deep red species observed during the synthesis of **2** at –35 °C / 238 K in CD_3CN, indicating the presence of the complex [Tipp–Si(IMe$_4$)$_2$→RuCl$_2$(*p*-cymene)]Cl (**2’**).

1.3.2 [^{t}Bu$_3$Si–Si(IMe$_4$)Cl→RuCl(IMe$_4$)(*p*-cymene)]Cl (6a)

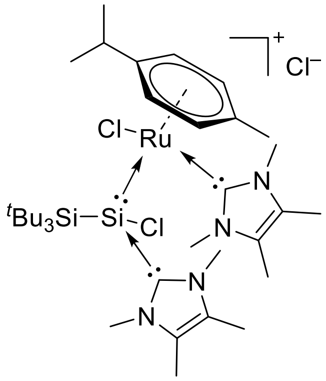

6a
$C_{36}H_{65}Cl_3N_4RuSi_2$
817.53 g/mol

Note: *The complex decomposes in solution at room temperature to an unidentified mixture of products.*

Batch size: **5a**: 100.0 mg, 195.6 µmol, 1.0 eq.
[RuCl$_2$(*p*-cymene)]$_2$: 59.9 mg, 97.8 µmol, 0.5 eq.

Yield: 102.6 mg (125.5 µmol, 64%) as an orange solid.

SC-XRD: Suitable crystals were obtained by slow diffusion of Et$_2$O into a concentrated solution of **6a** in MeCN at –35 °C.

^{1}H NMR (500 MHz, CD$_3$CN, 300 K): δ [ppm] = 5.91 (dd, $^3J_{H–H}$ = 6.2, $^4J_{H–H}$ = 0.9 Hz, 1H, $C_{p\text{-cym}}H_{ar}$), 5.56 (dd, $^3J_{H–H}$ = 5.9, $^4J_{H–H}$ = 1.2 Hz, 1H, $C_{p\text{-cym}}H_{ar}$), 5.42 (dd, $^3J_{H–H}$ = 6.2, $^4J_{H–H}$ = 1.2 Hz, 1H, $C_{p\text{-cym}}H_{ar}$), 5.18 (dd, $^3J_{H–H}$ = 5.9, $^4J_{H–H}$ = 0.9 Hz, 1H, $C_{p\text{-cym}}H_{ar}$), 4.15 (s, 3H, $N_{NHC→Si}CH_3$), 3.86 (s, 3H, $N_{NHC→Ru}CH_3$), 2.99 (s, 3H, $N_{NHC→Ru}CH_3$), 2.90 (s, 3H, $N_{NHC→Si}CH_3$), 2.61 (sept, $^3J_{H–H}$ = 6.9 Hz, 1H, C*H*(CH$_3$)$_2$), 2.20 (s, 3H, $C_{NHC→Si}CH_3$), 2.15 (s, 3H, $C_{NHC→Ru}CH_3$), 1.99 (s, 3H, $C_{p\text{-cym}}CH_3$), 1.86 (s, 3H, $C_{NHC→Ru}CH_3$), 1.83 (s, 3H, $C_{NHC→Si}CH_3$), 1.25 (s, 27H, Si((C(C*H*$_3$)$_3$)$_3$), 1.22 (d, $^3J_{H–H}$ = 6.9 Hz, 3H, CH(C*H*$_3$)$_2$), 1.17 (d, $^3J_{H–H}$ = 6.9 Hz, 3H, CH(C*H*$_3$)$_2$).

^{13}C{^{1}H} NMR (126 MHz, CD$_3$CN, 300 K): δ [ppm] = 172.1 ($NC_{NHC→Ru}N$), 155.2 ($NC_{NHC→Si}N$), 131.2 ($C_{NHC→Si}CH_3$), 128.9 ($C_{NHC→Ru}CH_3$), 128.7 ($C_{NHC→Si}CH_3$), 127.1 ($C_{NHC→Ru}CH_3$), 124.6 ($C_{p\text{-cym}}$–^{i}Pr), 101.4 ($C_{p\text{-cym}}H_{ar}$), 94.2 ($C_{p\text{-cym}}CH_3$), 91.1 ($C_{p\text{-cym}}H_{ar}$), 85.0 ($C_{p\text{-cym}}H_{ar}$), 83.9 ($C_{p\text{-cym}}H_{ar}$), 37.7 ($N_{NHC→Ru}CH_3$), 37.6 ($N_{NHC→Ru}CH_3$), 36.8 ($N_{NHC→Si}CH_3$), 36.4 ($N_{NHC→Si}CH_3$), 33.1 (Si((C(*C*H$_3$)$_3$)$_3$), 31.5 (*C*H(CH$_3$)$_2$), 26.4 (Si((*C*(CH$_3$)$_3$)$_3$), 22.4 (CH(*C*H$_3$)$_2$), 21.8 (CH(*C*H$_3$)$_2$), 17.7 ($C_{p\text{-cym}}CH_3$), 9.8 ($C_{NHC→Ru}CH_3$), 9.8 ($C_{NHC→Ru}CH_3$), 9.7 ($C_{NHC→Si}CH_3$), 8.5 ($C_{NHC→Si}CH_3$).

^{29}Si{^{1}H} NMR (99 MHz, CD_3CN, 300 K): δ [ppm] = 29.4 (*Si*Ru), 23.3 (*Si*^{t}Bu$_3$).

EA: $C_{36}H_{65}Cl_3N_4RuSi_2$ calculated [%]: C (52.89), H (8.01), N (6.85).
measured [%]: C (53.12), H (8.23), N (7.11).

M.P.: 122-123 °C (decomposition, color change to black).

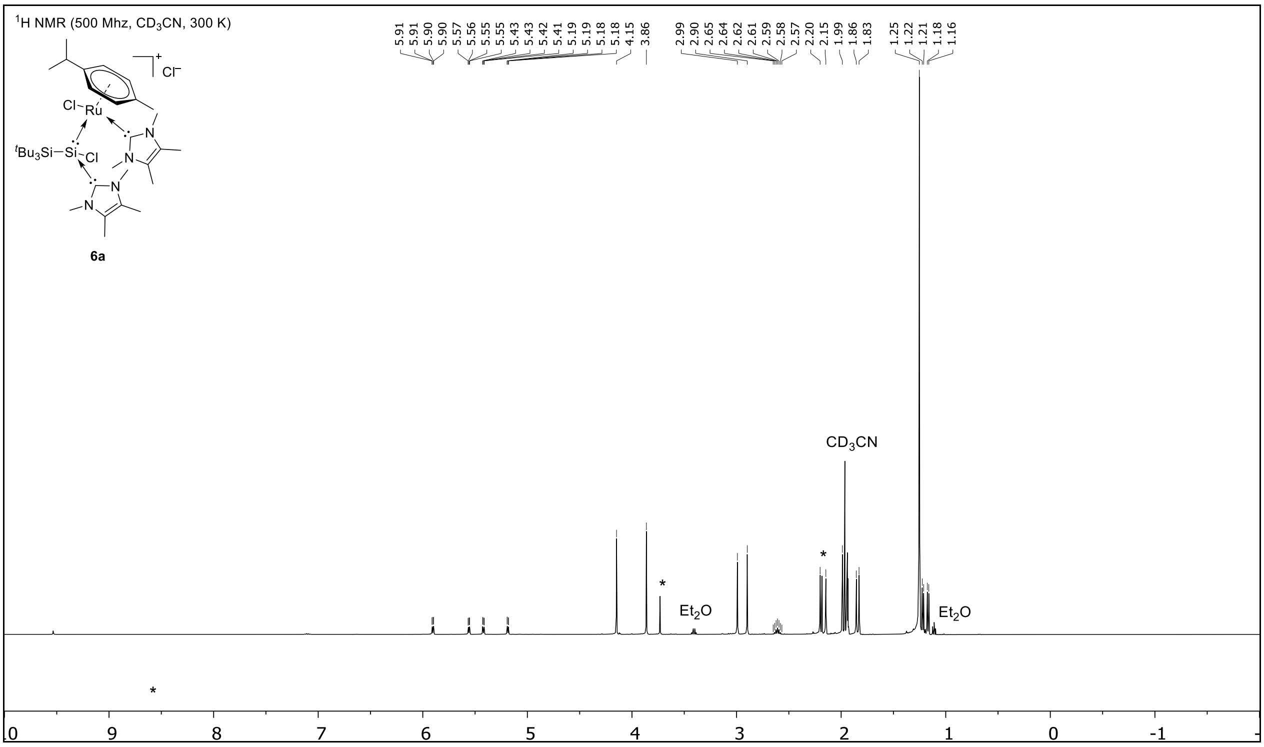

Figure S17 ^{1}H NMR spectrum of [^{t}Bu$_3$Si–Si(IMe$_4$)Cl→RuCl(IMe$_4$)(*p*-cymene)]Cl (**6a**) in CD_3CN at 300 K. Residual imidazolium chloride [IMe$_4$·HCl] from the synthesis of **5a** and from the decomposition of **6a** is marked with *.

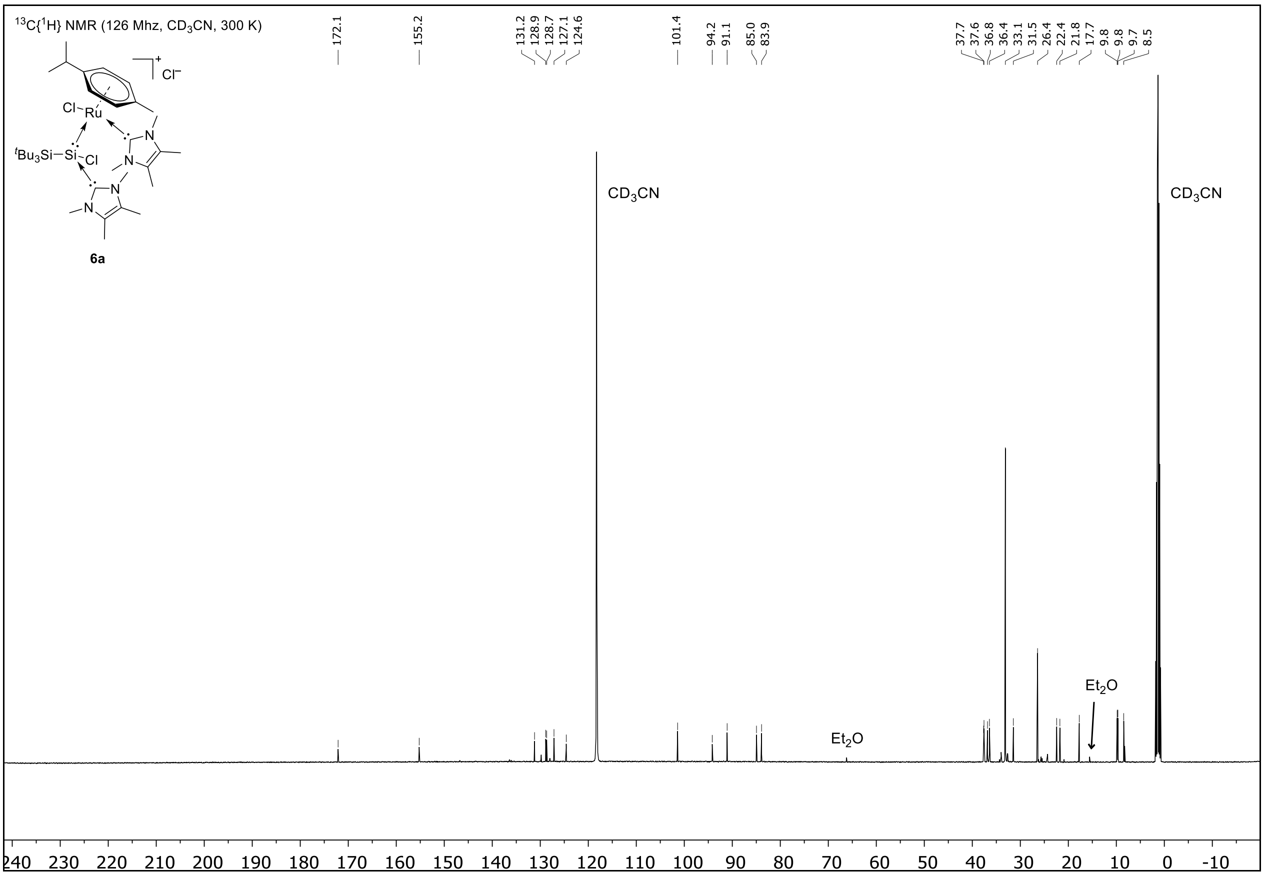

Figure S18 $^{13}C\{^{1}H\}$ NMR spectrum of [$^{t}Bu_3Si$–Si(IMe_4)Cl→RuCl(IMe_4)(*p*-cymene)]Cl (**6a**) in CD_3CN at 300 K.

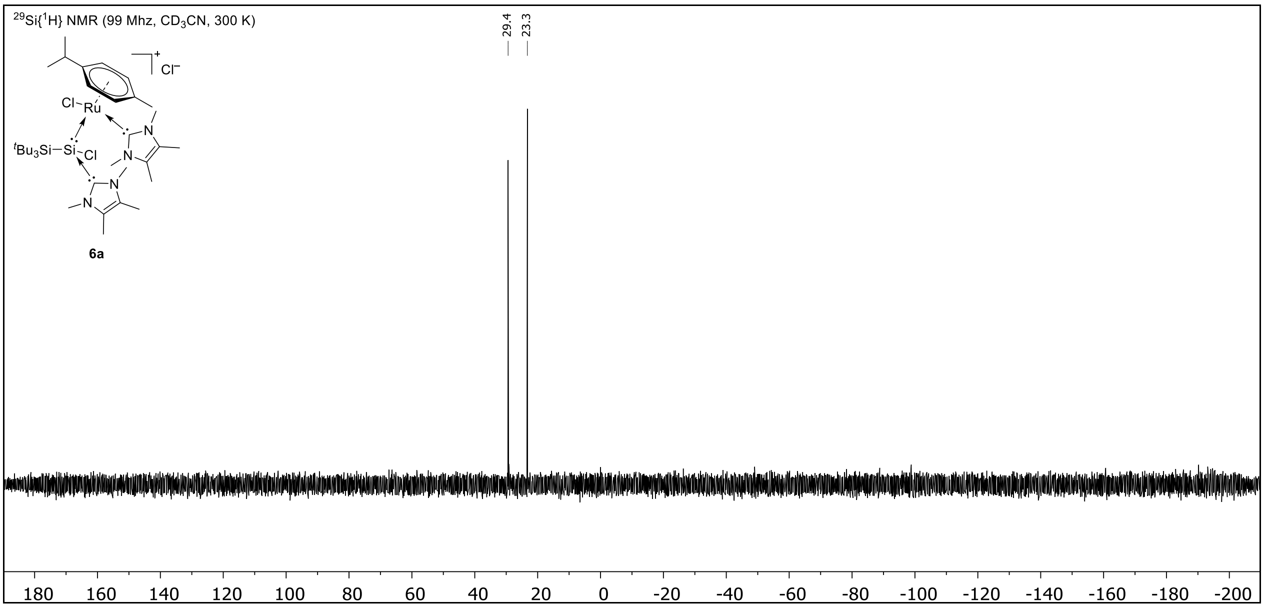

Figure S19 $^{29}Si\{^{1}H\}$ NMR spectrum of [$^{t}Bu_3Si$–Si(IMe_4)Cl→RuCl(IMe_4)(*p*-cymene)]Cl (**6a**) in CD_3CN at 300 K.

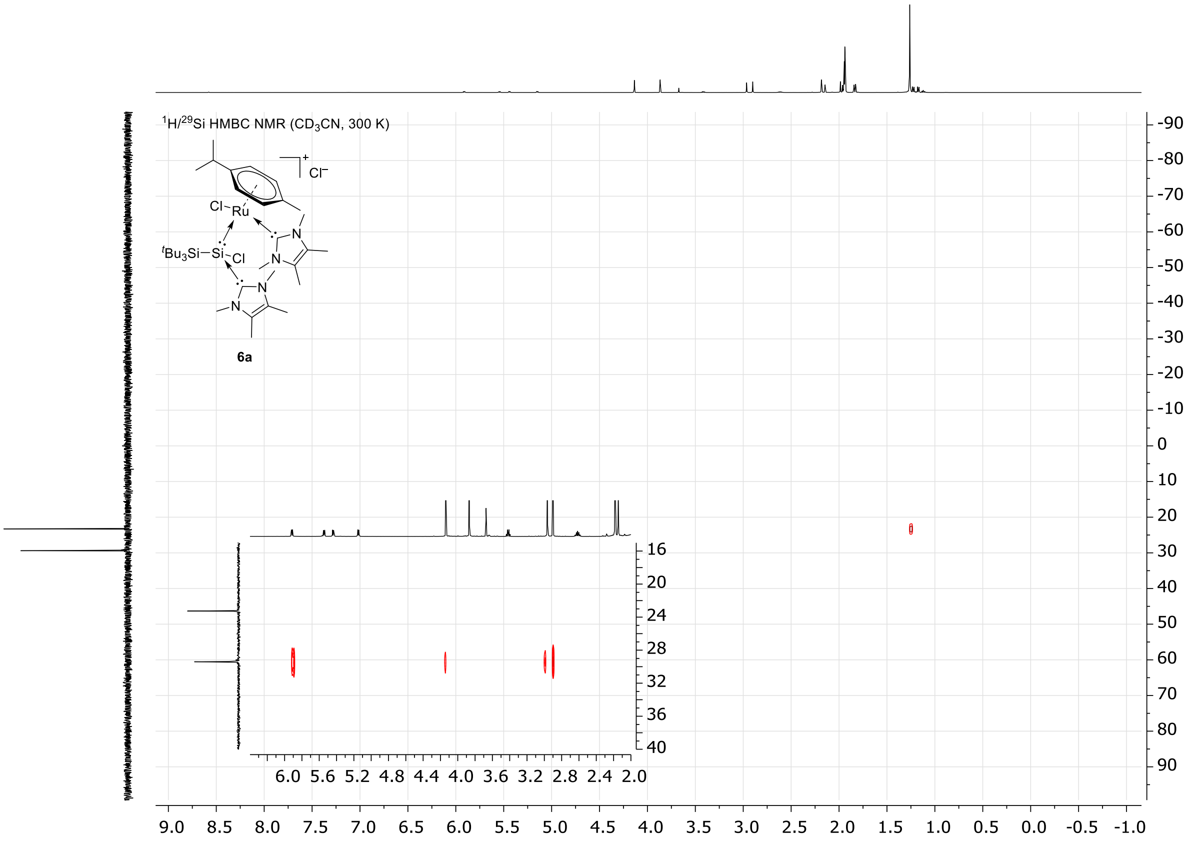

Figure S20 $^{1}H/^{29}Si$ HMBC NMR spectrum of [$^{t}Bu_3Si$–Si(IMe$_4$)Cl→RuCl(IMe$_4$)(*p*-cymene)]Cl (**6a**) in CD_3CN at 300 K.

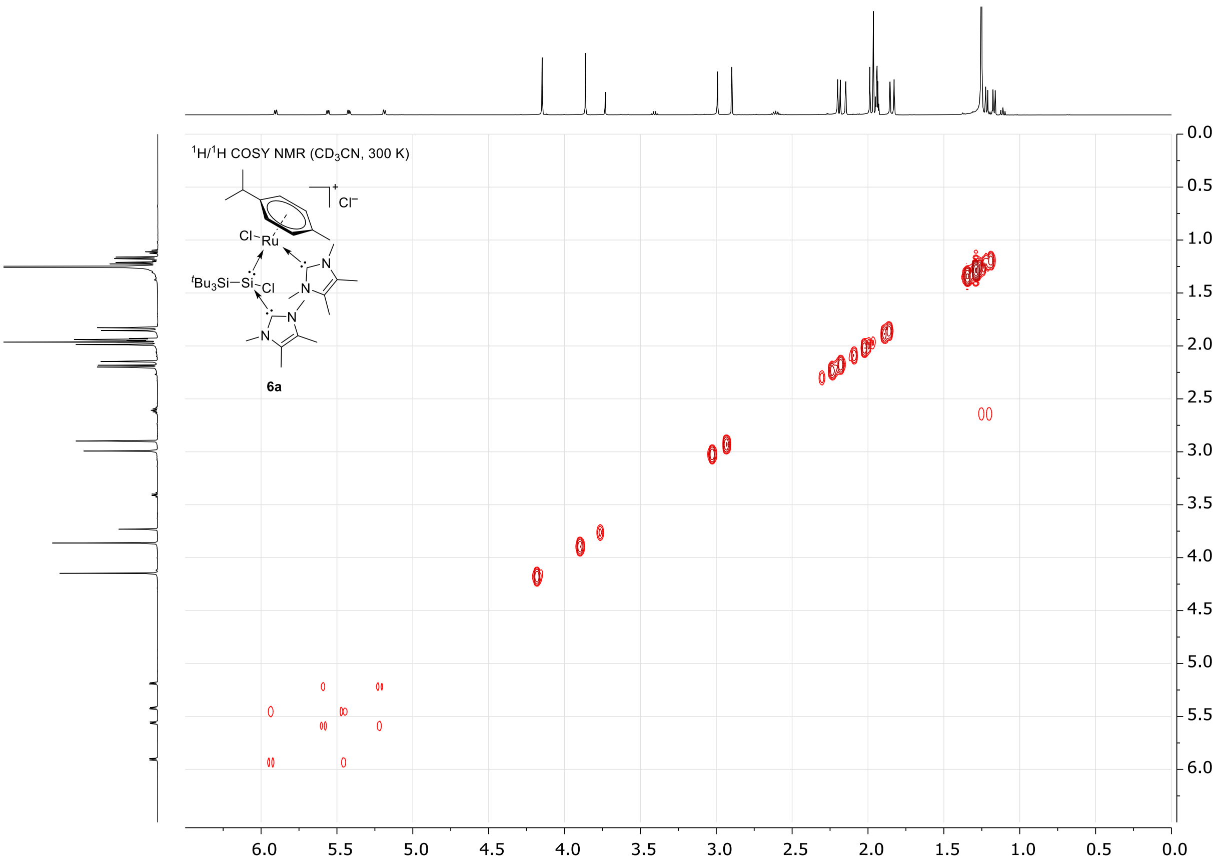

Figure S21 $^{1}H/^{1}H$ COSY NMR spectrum of [$^{t}Bu_3Si$–Si(IMe$_4$)Cl→RuCl(IMe$_4$)(*p*-cymene)]Cl (**6a**) in CD_3CN at 300 K.

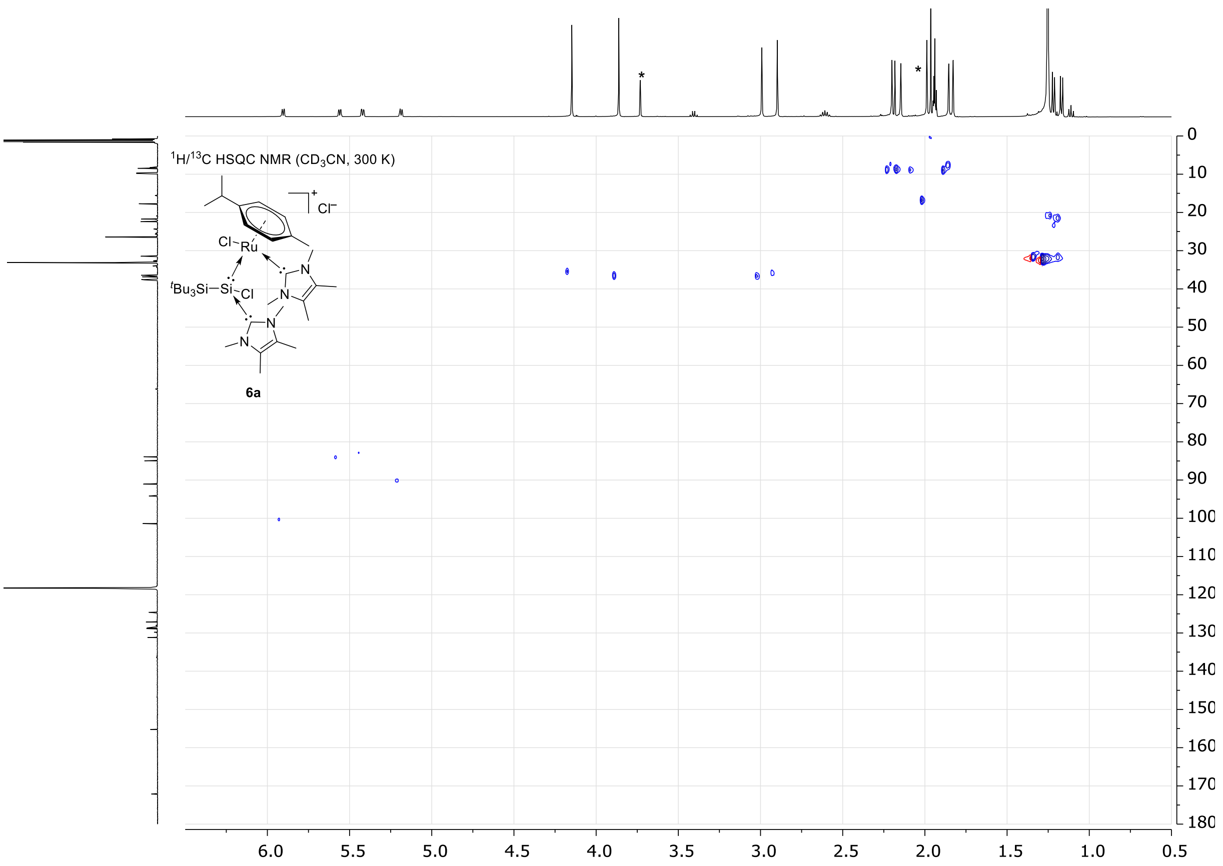

Figure S22 $^{1}H/^{13}C$ HSQC NMR spectrum of [$^{t}Bu_3Si$–Si(IMe$_4$)Cl→RuCl(IMe$_4$)(*p*-cymene)]Cl (**6a**) in CD_3CN at 300 K.

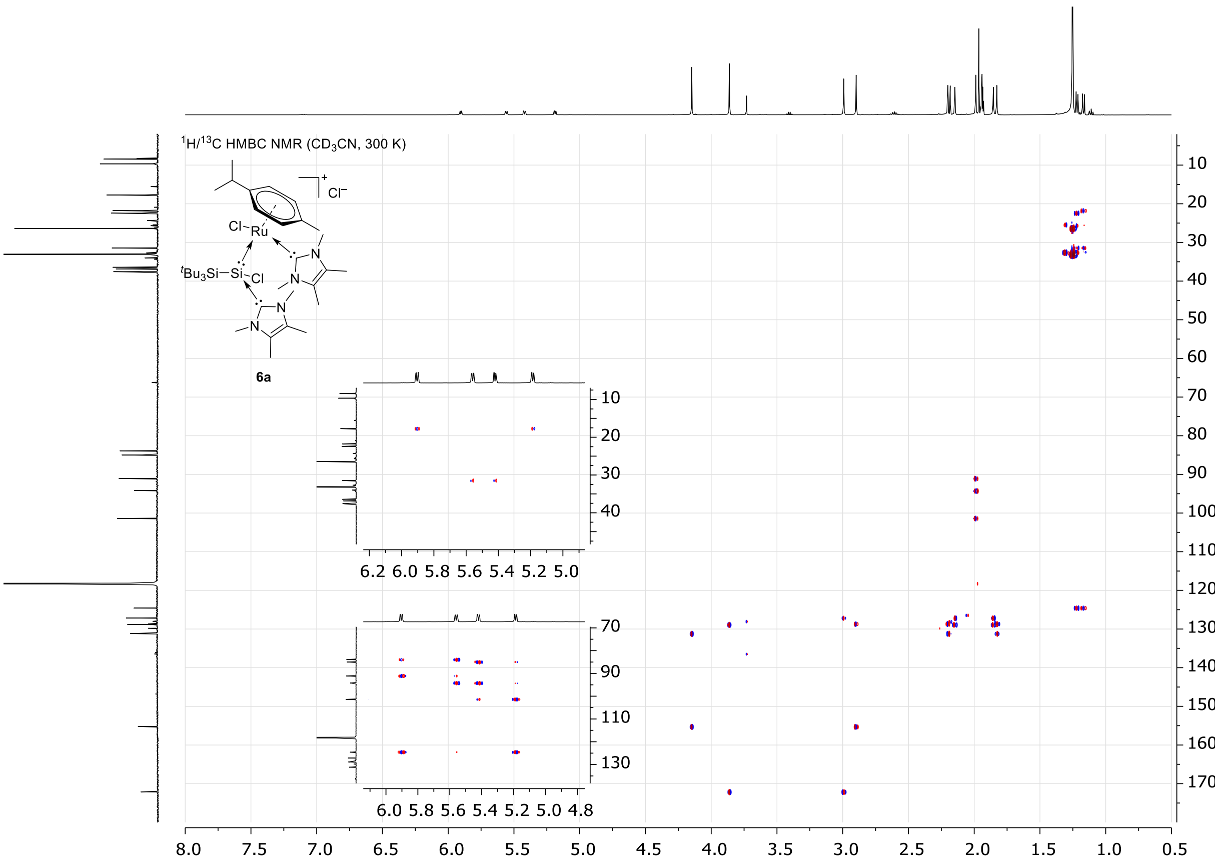

Figure S23 $^{1}H/^{13}C$ HMBC NMR spectrum of [$^{t}Bu_3Si$–Si(IMe$_4$)Cl→RuCl(IMe$_4$)(*p*-cymene)]Cl (**6a**) in CD_3CN at 300 K.

1.3.3 [^{t}Bu$_2$MeSi–Si(IMe$_4$)Cl→RuCl(IMe$_4$)(*p*-cymene)]Cl (6b)

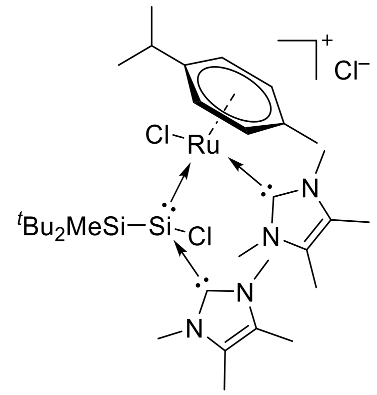

6b

$C_{33}H_{59}Cl_3N_4RuSi_2$

775.45 g/mol

Note: *The complex decomposes in solution at room temperature to an unidentified mixture of products.*

Batch size: **5b**: 50.0 mg, 106.6 µmol, 1.0 eq.

[RuCl$_2$(*p*-cymene)]$_2$: 32.6 mg, 53.3 µmol, 0.5 eq.

Yield: 46.4 mg (59.8 µmol, 58%) as an orange solid.

^{1}H NMR (500 MHz, CD$_3$CN, 300 K): δ [ppm] = 5.94 (dd, $^3J_{H–H}$ = 6.0 Hz, $^4J_{H–H}$ = 1.2 Hz, 1H, $C_{p\text{-cym}}H_{ar}$), 5.65 (dd, $^3J_{H–H}$ = 6.1 Hz, $^4J_{H–H}$ = 1.1 Hz, 1H, $C_{p\text{-cym}}H_{ar}$), 5.16 (dd, $^3J_{H–H}$ = 6.0 Hz, $^4J_{H–H}$ = 1.1 Hz, 1H, $C_{p\text{-cym}}H_{ar}$), 4.87 (dd, $^3J_{H–H}$ = 6.1 Hz, $^4J_{H–H}$ = 1.2 Hz, 1H, $C_{p\text{-cym}}H_{ar}$), 4.09 (s, 3H, $N_{NHC\to Si}CH_3$), 3.84 (s, 3H, $N_{NHC\to Ru}CH_3$), 2.96 (s, 3H, $N_{NHC\to Ru}CH_3$), 2.89 (s, 3H, $N_{NHC\to Si}CH_3$), 2.66 (sept, $^3J_{H–H}$ = 7.0 Hz, 1H, $CH(CH_3)_2$), 2.21 (s, 3H, $C_{NHC\to Si}CH_3$), 2.14 (s, 3H, $C_{NHC\to Ru}CH_3$), 2.11 (s, 3H, $C_{p\text{-cym}}CH_3$), 1.85 (s, 3H, $C_{NHC\to Ru}CH_3$), 1.83 (s, 3H, $C_{NHC\to Si}CH_3$), 1.26 (s, 9H, $Si(C(CH_3)_3)_2CH_3$), 1.24 (d, $^3J_{H–H}$ = 7.0 Hz, 3H, $CH(CH_3)_2$), 1.19 (d, $^3J_{H–H}$ = 7.0 Hz, 3H, $CH(CH_3)_2$), 0.72 (s, 9H, $Si(C(CH_3)_3)_2CH_3$), 0.35 (s, 3H, $Si(C(CH_3)_3)_2CH_3$).

^{13}C{^{1}H} NMR (101 MHz, CD$_3$CN, 300 K): δ [ppm] = 172.7 ($NC_{NHC\to Ru}N$), 155.3 ($NC_{NHC\to Si}N$), 130.6 ($C_{NHC\to Si}CH_3$), 128.8 ($C_{NHC\to Ru}CH_3$), 128.8 ($C_{NHC\to Si}CH_3$), 127.1 ($C_{NHC\to Ru}CH_3$), 124.7 ($C_{p\text{-cym}}$–^{i}Pr), 99.8 ($C_{p\text{-cym}}H_{ar}$), 94.4 ($C_{p\text{-cym}}CH_3$), 87.7 ($C_{p\text{-cym}}H_{ar}$), 87.5 ($C_{p\text{-cym}}H_{ar}$), 87.2 ($C_{p\text{-cym}}H_{ar}$), 38.0 ($N_{NHC\to Ru}CH_3$), 37.4 ($N_{NHC\to Ru}CH_3$), 35.9 ($N_{NHC\to Si}CH_3$), 35.8 ($N_{NHC\to Si}CH_3$), 31.2 ($Si(C(CH_3)_3)_2CH_3$), 30.9 ($CH(CH_3)_2$), 29.8 ($Si(C(CH_3)_3)_2CH_3$), 24.4 ($Si(C(CH_3)_3)_2CH_3$), 23.9 ($Si(C(CH_3)_3)_2CH_3$), 23.2 ($CH(CH_3)_2$), 20.9 ($CH(CH_3)_2$), 17.6 ($C_{p\text{-cym}}CH_3$), 9.9 ($C_{NHC\to Ru}CH_3$), 9.6 ($C_{NHC\to Ru}CH_3$), 9.5 ($C_{NHC\to Si}CH_3$), 8.3 ($C_{NHC\to Si}CH_3$), 0.8 ($Si(C(CH_3)_3)_2CH_3$).

Note: *The resonance corresponding to the CH_3-substituent of the $^{t}Bu_2MeSi$ moiety (0.8 ppm) overlaps with the resonance of CD_3CN (1.3 ppm, sept) and was determined from $^{1}H/^{13}C$ HSQC NMR.*

$^{29}Si\{^{1}H\}$ NMR (99 MHz, CD_3CN, 300 K): δ [ppm] = 29.4 (*Si*Ru), 8.7 (*Si*$^{t}Bu_2Me$).

EA: $C_{33}H_{59}Cl_3N_4RuSi_2$ calculated [%]: C (51.11), H (7.67), N (7.23).

measured [%]: C (51.25), H (7.79), N (7.54).

M.P.: 119-120 °C (decomposition, color change to black).

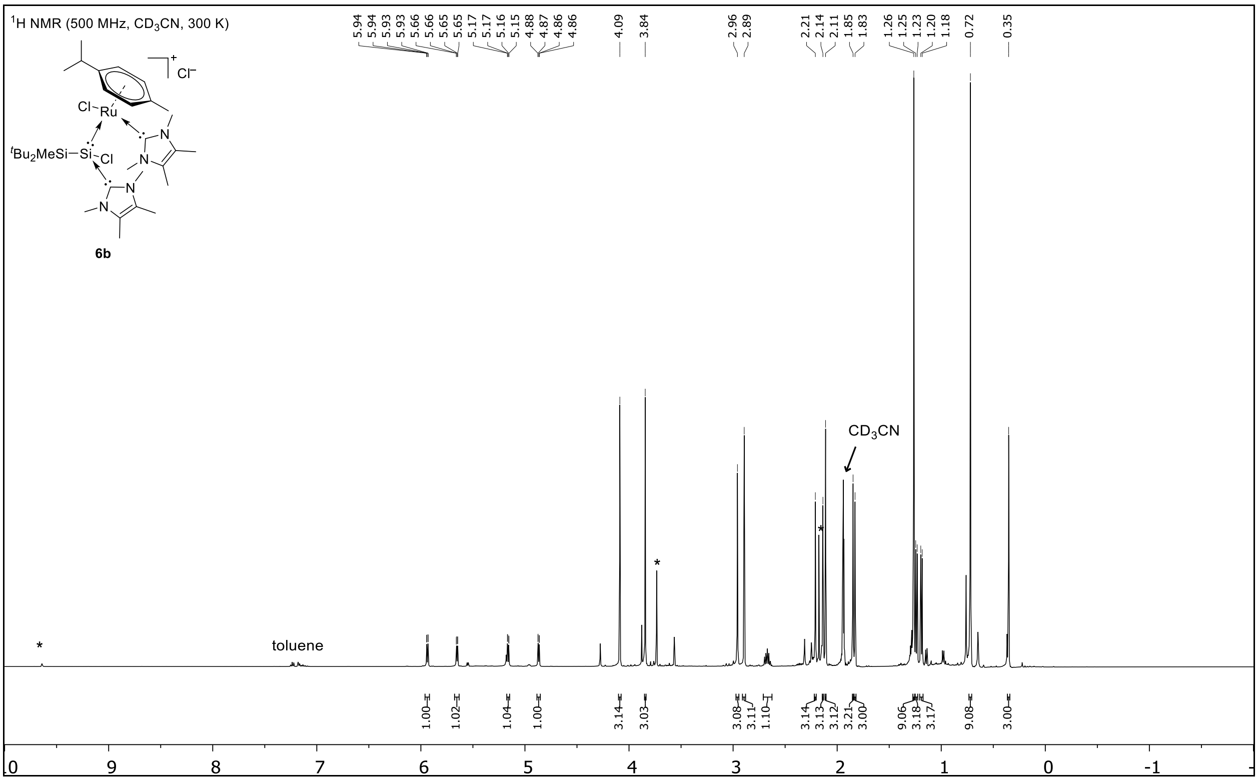

Figure S24 ^{1}H NMR spectrum of [$^{t}Bu_2MeSi$–Si(IMe_4)Cl→RuCl(IMe_4)(*p*-cymene)]Cl (**6b**) in CD_3CN at 300 K. Small amounts of residual imidazolium chloride [IMe_4·HCl] from the synthesis of **2b** and the decomposition of **6b** are marked with *.

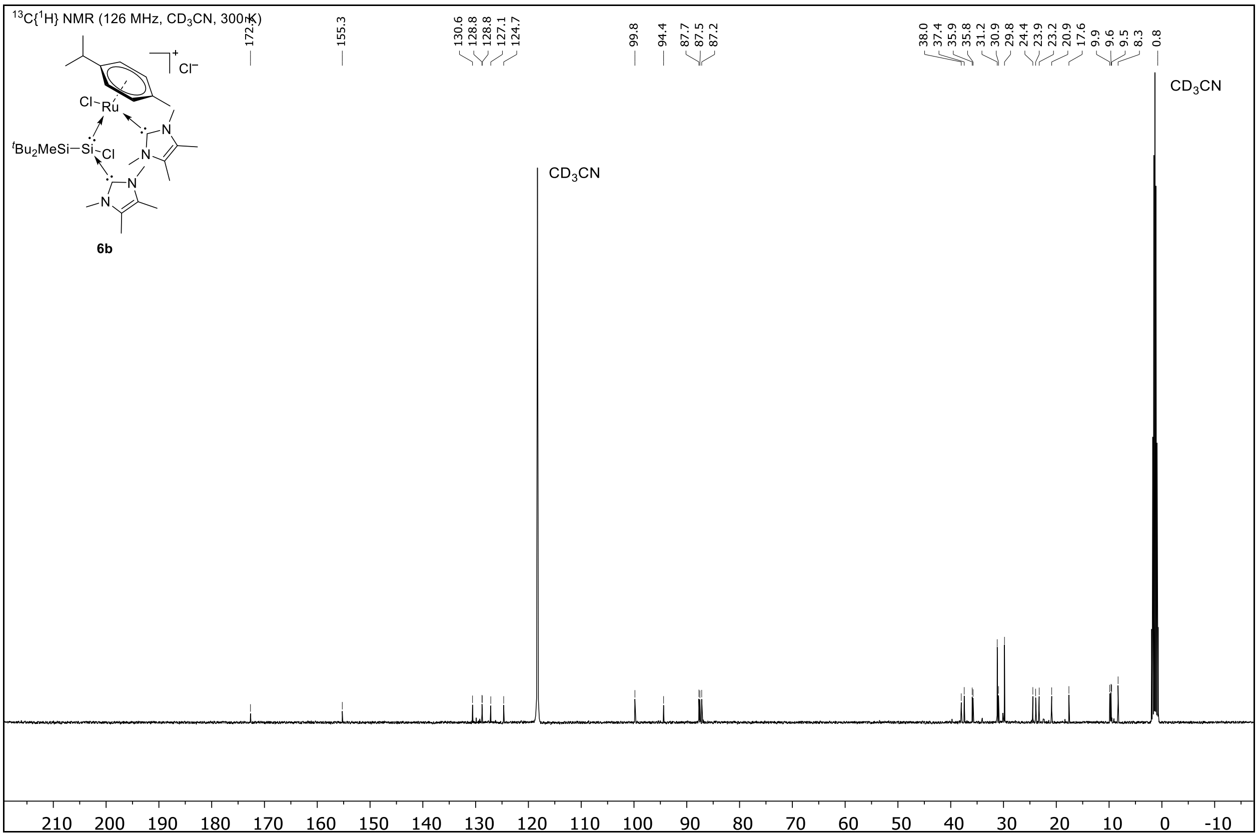

Figure S25 $^{13}C\{^1H\}$ NMR spectrum of [$^tBu_2MeSi$–Si(IMe$_4$)Cl→RuCl(IMe$_4$)(*p*-cymene)]Cl (**6b**) in CD_3CN at 300 K.

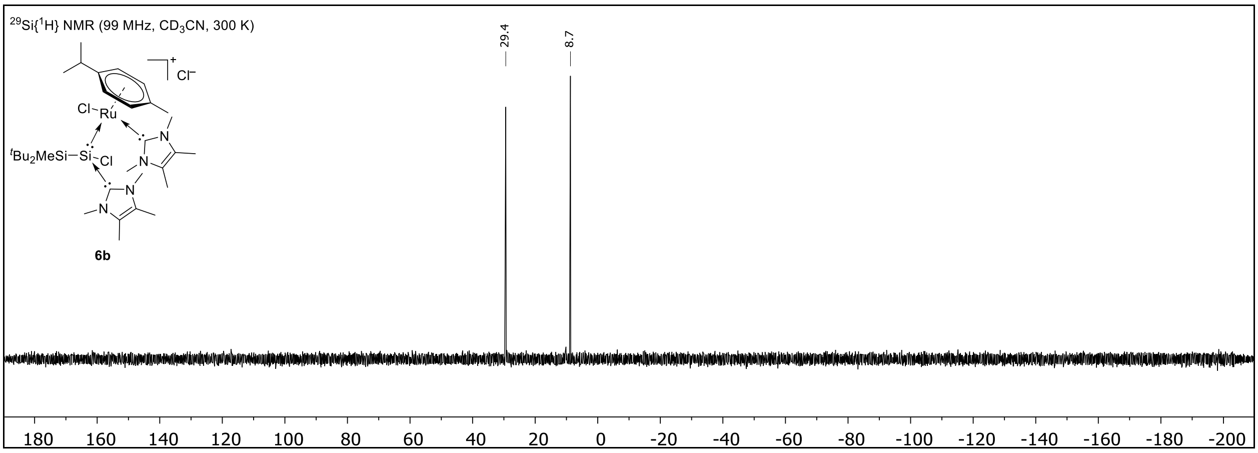

Figure S26 $^{29}Si\{^1H\}$ NMR spectrum of [$^tBu_2MeSi$–Si(IMe$_4$)Cl→RuCl(IMe$_4$)(*p*-cymene)]Cl (**6b**) in CD_3CN at 300 K.

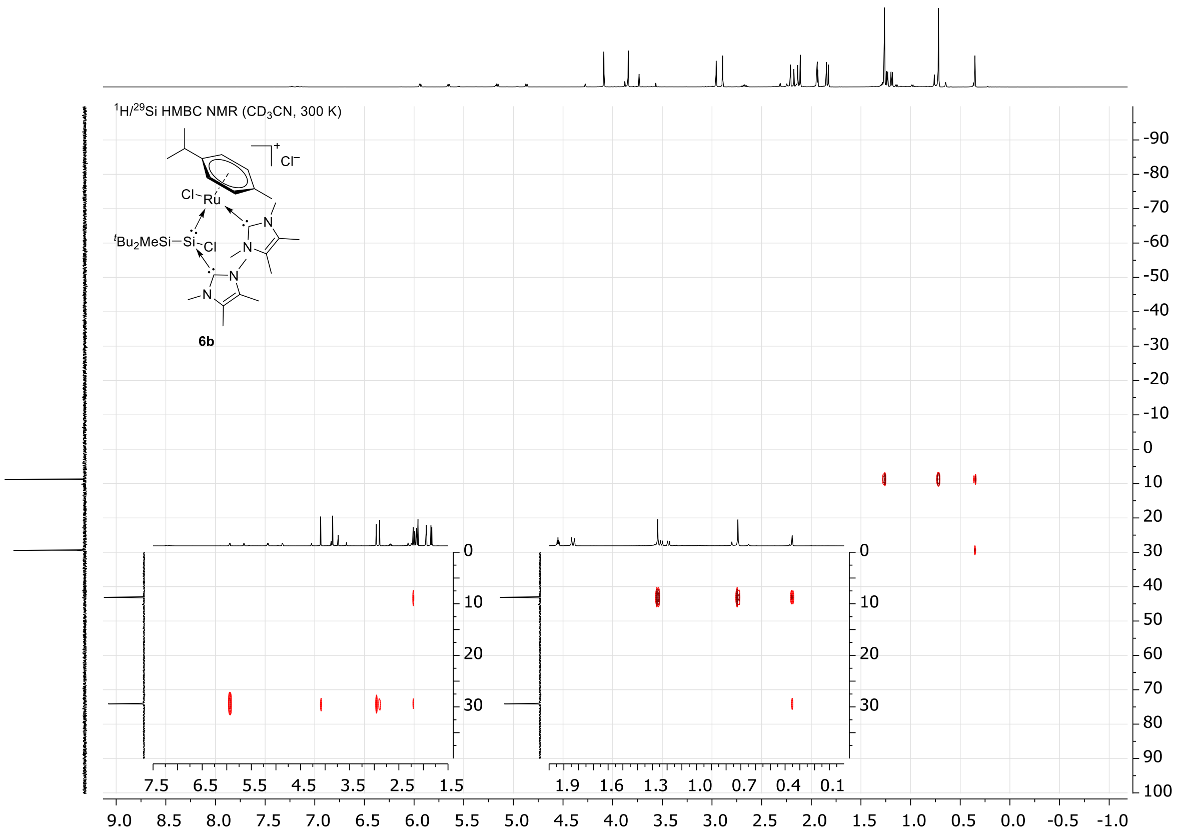

Figure S27 $^1H/^{29}Si$ HMBC NMR spectrum of [$^tBu_2MeSi$–Si(IMe_4)Cl→RuCl(IMe_4)(*p*-cymene)]Cl (**6b**) in CD_3CN at 300 K.

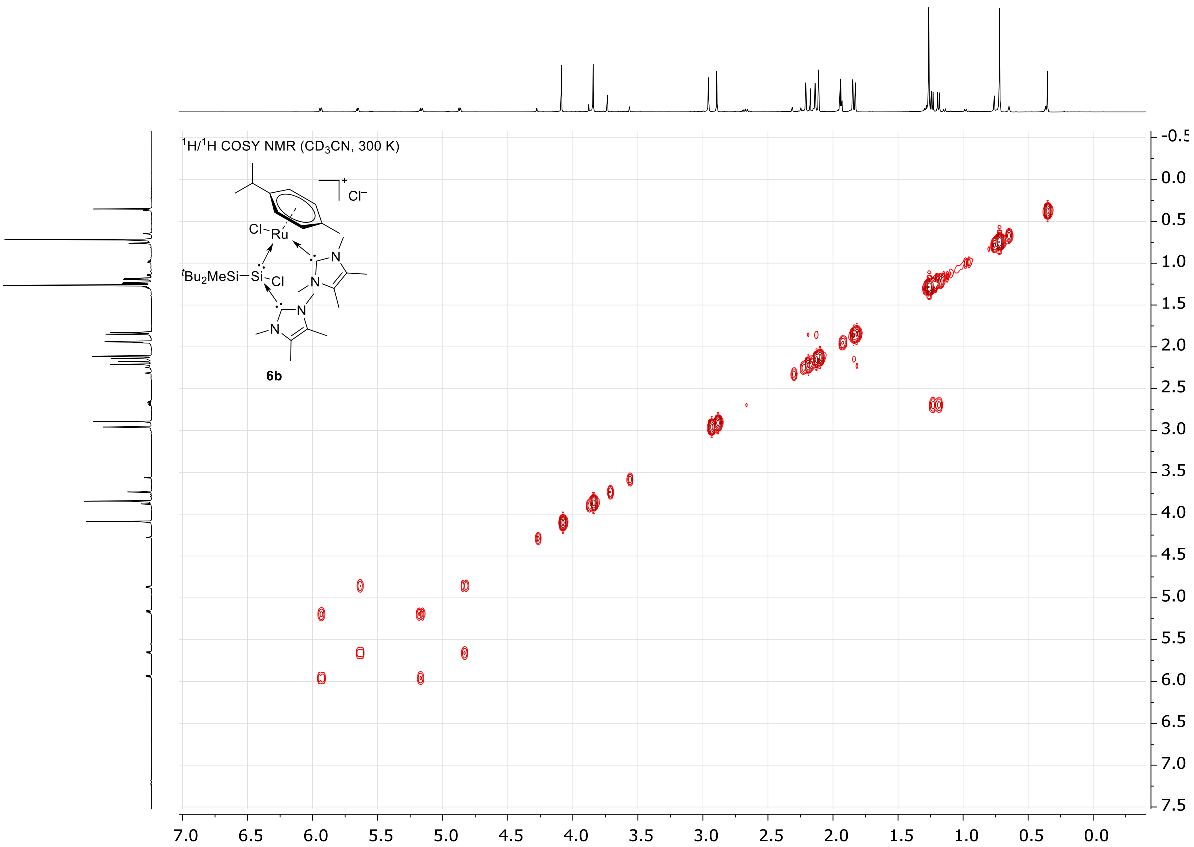

Figure S28 $^1H/^1H$ COSY NMR spectrum of [$^tBu_2MeSi$–Si(IMe_4)Cl→RuCl(IMe_4)(*p*-cymene)]Cl (**6b**) in CD_3CN at 300 K.

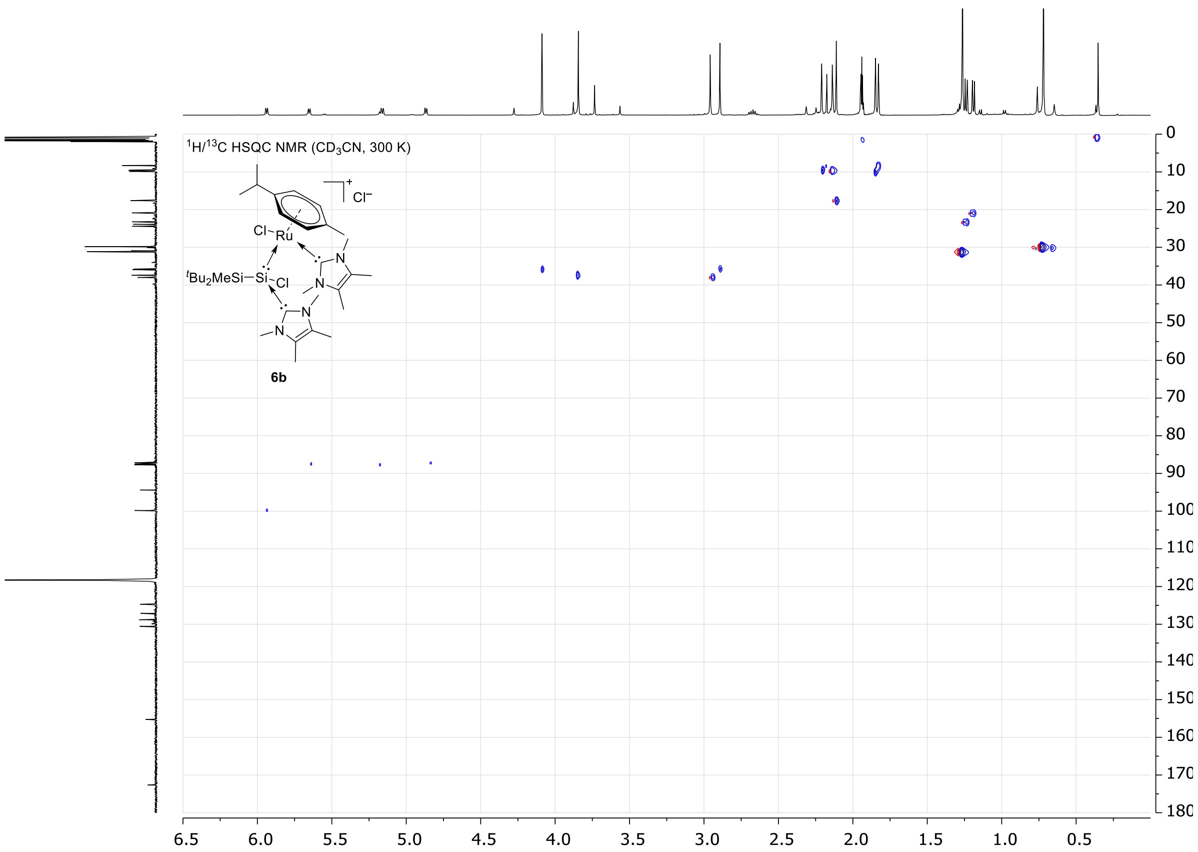

Figure S29 $^1H/^{13}C$ HSQC NMR spectrum of [$^tBu_2MeSi$–Si(IMe$_4$)Cl→RuCl(IMe$_4$)(*p*-cymene)]Cl (**6b**) in CD_3CN at 300 K.

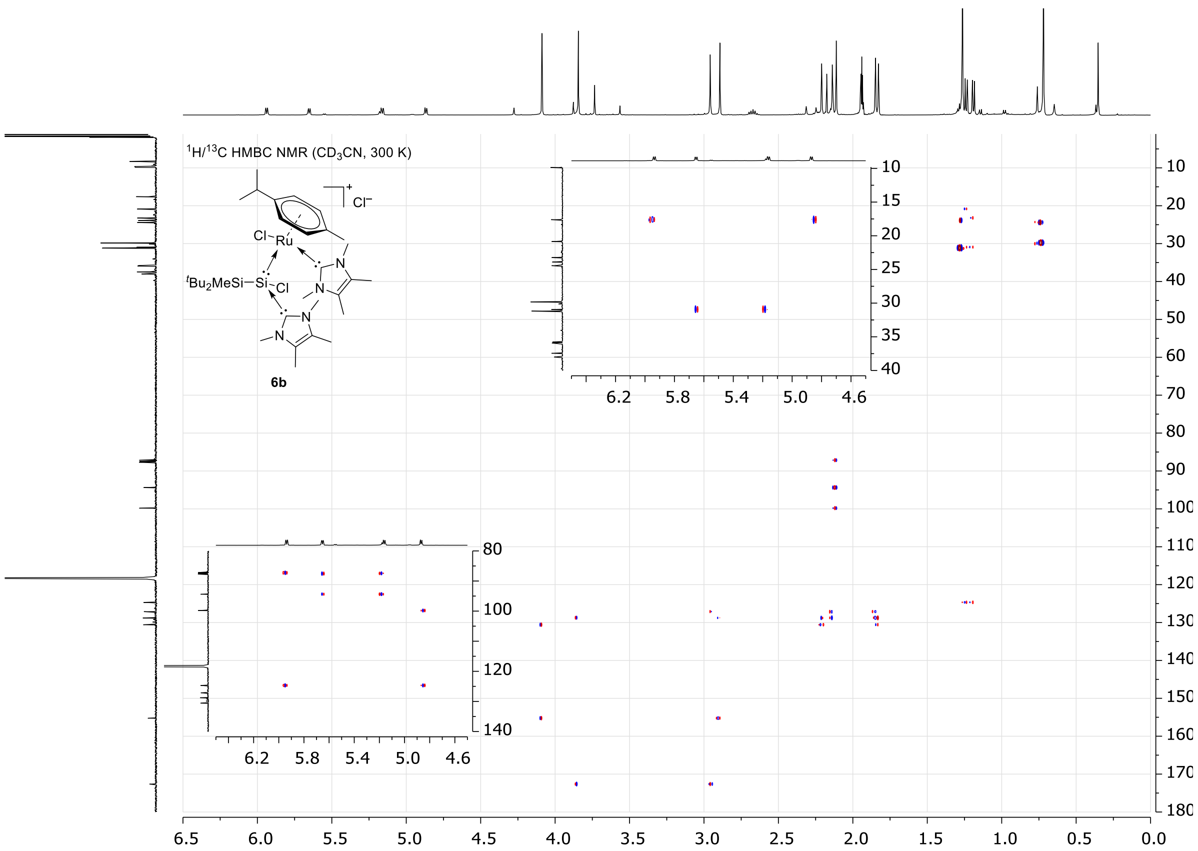

Figure S30 $^1H/^{13}C$ HMBC NMR spectrum of [$^tBu_2MeSi$–Si(IMe$_4$)Cl→RuCl(IMe$_4$)(*p*-cymene)]Cl (**6b**) in CD_3CN at 300 K.

1.3.4 [^{t}Bu$_2$MeSi–Si(IEt$_2$Me$_2$)Cl→RuCl(IEt$_2$Me$_2$)(*p*-cymene)]Cl (6c)

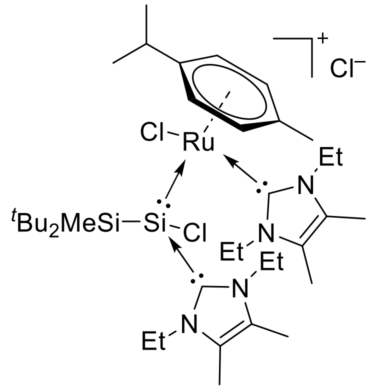

6c

$C_{37}H_{67}Cl_3N_4RuSi_2$

831.56 g/mol

Note: ***(a)*** *The complex decomposes in solution at room temperature to an unidentified mixture of products;* ***(b)*** *The reaction takes longer to complete than the reactions with the IMe$_4$-stabilized silyliumylidene ions (~30 minutes vs. 1-3 minutes) due to the increased steric hindrance of the NHCs. Attempts to isolate the silyliumylidene complex (analogous to* ***2'****) failed. The rate determining step appears to be the coordination of the silyliumylidene* ***5c*** *to the transition metal precursor with the insertion reaction occurring significantly faster. At low temperature (to slow down the insertion step) no coordinating reaction was observed.*

Batch size: **5c**: 50.0 mg, 95.2 µmol, 1.0 eq.
[RuCl$_2$(*p*-cymene)]$_2$: 29.1 mg, 47.6 µmol, 0.5 eq.

Yield: 35.3 mg (42.5 µmol, 45%) as an orange solid.

^{1}H NMR (500 MHz, CD$_3$CN, 300 K): δ [ppm] = 6.13 (dd, $^3J_{H–H}$ = 6.4 Hz, $^4J_{H–H}$ = 1.4 Hz, 1H, C$_{p\text{-cym}}H_{ar}$), 5.73 (dd, $^3J_{H–H}$ = 5.9 Hz, $^4J_{H–H}$ = 1.6 Hz, 1H, C$_{p\text{-cym}}H_{ar}$), 5.38 (dd, $^3J_{H–H}$ = 6.4 Hz, $^4J_{H–H}$ = 1.4 Hz, 1H, C$_{p\text{-cym}}H_{ar}$), 5.00 (dd, $^3J_{H–H}$ = 5.9 Hz, $^4J_{H–H}$ = 1.6 Hz, 1H, C$_{p\text{-cym}}H_{ar}$), 4.89 (dq, $^2J_{H–H}$ = 14.3 Hz, $^3J_{H–H}$ = 7.1 Hz, 1H, N$_{NHC→Si}$C*H*$_2$CH$_3$), 4.56 – 4.46 (m, 2H, N$_{NHC→Si}$C*H*$_2$CH$_3$, N$_{NHC→Ru}$C*H*$_2$CH$_3$), 4.35 (dq, $^2J_{H–H}$ = 14.2 Hz, $^3J_{H–H}$ = 7.1 Hz, 1H, N$_{NHC→Ru}$C*H*$_2$CH$_3$), 3.88 (dq, $^2J_{H–H}$ = 14.2 Hz, $^3J_{H–H}$ = 7.1 Hz, 1H, N$_{NHC→Si}$C*H*$_2$CH$_3$), 3.75 (dq, $^2J_{H–H}$ = 14.3 Hz, $^3J_{H–H}$ = 7.1 Hz, 1H, N$_{NHC→Ru}$C*H*$_2$CH$_3$), 2.68 (sept, $^3J_{H–H}$ = 6.9 Hz, 1H, C*H*(CH$_3$)$_2$), 2.51 (dq, J = 13.9, 7.0 Hz, 1H, N$_{NHC→Ru}$C*H*$_2$CH$_3$), 2.37 – 2.31 (m, 1H, N$_{NHC→Si}$C*H*$_2$CH$_3$), 2.26 (s, 3H, C$_{NHC→Si}$C*H*$_3$), 2.20 (s, 3H, C$_{NHC→Ru}$C*H*$_3$), 2.12 (s, 3H, C$_{p\text{-cym}}$C*H*$_3$), 1.99 (s, 3H, C$_{NHC→Si}$C*H*$_3$), 1.96 (s, 3H, C$_{NHC→Ru}$C*H*$_3$), 1.43 (t, $^3J_{H–H}$ = 7.1 Hz, 3H, N$_{NHC→Si}$CH$_2$C*H*$_3$), 1.31 (d, $^3J_{H–H}$ = 6.9 Hz, 3H, CH(C*H*$_3$)$_2$), 1.30 – 1.26 (m, 12H, Si(C(C*H*$_3$)$_3$)$_2$CH$_3$, N$_{NHC→Ru}$CH$_2$C*H*$_3$), 1.25 (d, $^3J_{H–H}$ = 6.9 Hz, 3H, CH(C*H*$_3$)$_2$), 1.11 (t, $^3J_{H–H}$ = 7.1 Hz, 3H, N$_{NHC→Ru}$CH$_2$C*H*$_3$), 0.86 – 0.81 (m, 12H, Si(C(C*H*$_3$)$_3$)$_2$CH$_3$, N$_{NHC→Si}$CH$_2$C*H*$_3$), 0.37 (s, 3H, Si(C(CH$_3$)$_3$)$_2$C*H*$_3$).

***Note:** 3 of the 4 signals corresponding to the CH_3 groups in the NHC-wingtip substituents (triplets at 1.43, 1.28 and 0.84 ppm) overlap with (**I**) the triplet corresponding to the imidazolium chloride [IEt_2Me_2·HCl] impurity (1.41 ppm), (**II**) the singlet corresponding to one ^{t}Bu moiety in $^tBu_2$MeSi (1.28 ppm) and (**III**) the singlet corresponding to the other ^{t}Bu moiety in $^tBu_2$MeSi (0.82 ppm). They were determined using $^1H/^1H$ COSY, $^1H/^{13}C$ HSQC and $^1H/^{13}C$ HMBC measurements. The 4th signal does not overlap (triplet at 1.11 ppm).*

$^{13}C\{^1H\}$ NMR (126 MHz, CD_3CN, 300 K): δ [ppm] = 172.9 (N*C*$_{NHC\to Ru}$N), 154.8 (N*C*$_{NHC\to Si}$N), 131.9 (*C*$_{NHC\to Si}$CH$_3$), 130.0 (*C*$_{NHC\to Ru}$CH$_3$), 128.8 (*C*$_{NHC\to Si}$CH$_3$), 127.1 (*C*$_{NHC\to Ru}$CH$_3$), 125.7 (*C*$_{p\text{-cym}}$–^{i}Pr), 101.6 (*C*$_{p\text{-cym}}$H$_{ar}$), 90.7 (*C*$_{p\text{-cym}}$CH$_3$), 87.4 (*C*$_{p\text{-cym}}$H$_{ar}$), 87.2 (*C*$_{p\text{-cym}}$H$_{ar}$), 84.6 (*C*$_{p\text{-cym}}$H$_{ar}$), 45.4 (N$_{NHC\to Ru}$*C*H$_2$CH$_3$), 45.0 (N$_{NHC\to Ru}$*C*H$_2$CH$_3$), 44.1 (N$_{NHC\to Si}$*C*H$_2$CH$_3$), 43.4 (N$_{NHC\to Si}$*C*H$_2$CH$_3$), 31.6 (Si(C(*C*H$_3$)$_3$)$_2$CH$_3$), 31.1 (*C*H(CH$_3$)$_2$), 30.4 (Si(C(*C*H$_3$)$_3$)$_2$CH$_3$), 24.8 (Si(*C*(CH$_3$)$_3$)$_2$CH$_3$), 24.1 (Si(*C*(CH$_3$)$_3$)$_2$CH$_3$), 22.7 (CH(*C*H$_3$)$_2$), 21.4 (CH(*C*H$_3$)$_2$), 17.5 (N$_{NHC\to Ru}$CH$_2$*C*H$_3$), 17.3 (N$_{NHC\to Ru}$CH$_2$*C*H$_3$), 16.5 (C$_{p\text{-cym}}$*C*H$_3$), 15.9 (N$_{NHC\to Si}$CH$_2$*C*H$_3$), 14.7 (N$_{NHC\to Si}$CH$_2$*C*H$_3$), 10.2 (C$_{NHC\to Ru}$*C*H$_3$), 10.0 (C$_{NHC\to Si}$*C*H$_3$), 9.9 (C$_{NHC\to Ru}$*C*H$_3$), 9.4 (C$_{NHC\to Si}$*C*H$_3$), 0.4 (Si(C(CH$_3$)$_3$)$_2$*C*H$_3$).

$^{29}Si\{^1H\}$ NMR (99 MHz, CD_3CN, 300 K): δ [ppm] = 23.5 (*Si*Ru), 9.1 (*Si*^{t}Bu$_2$Me).

EA: $C_{37}H_{67}Cl_3N_4RuSi_2$ calculated [%]: C (53.44), H (8.12), N (6.74).
measured [%]: C (53.56), H (8.17), N (7.01).

M.P.: 128-129 °C (decomposition, color change to dark brown).

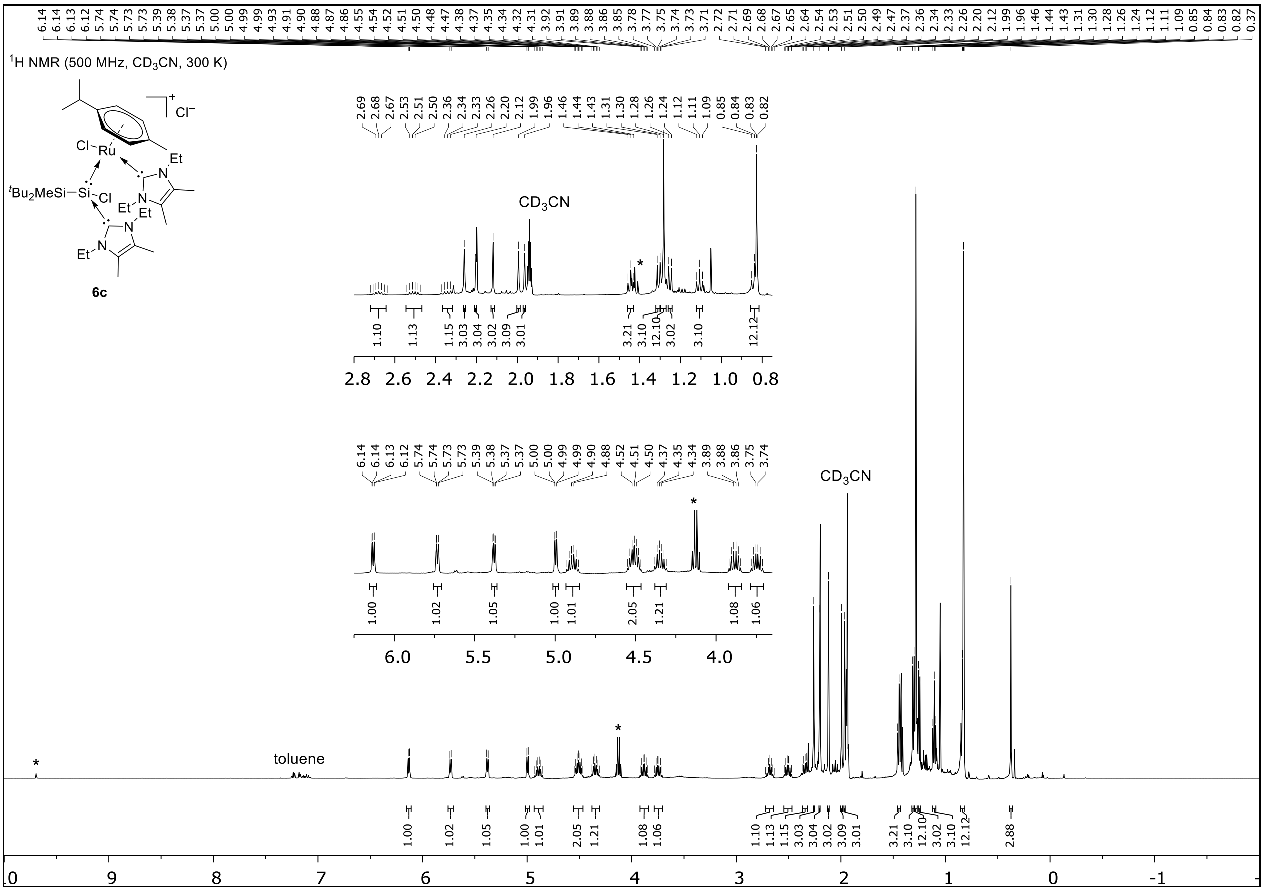

Figure S31 ^{1}H NMR spectrum of [$^{t}Bu_2MeSi$–Si(IEt_2Me_2)Cl→Ru(*p*-cymene)Cl(IEt_2Me_2)]Cl (**6c**) in CD_3CN at 300 K. Residual imidazolium chloride [IEt_2Me_2·HCl] from the silyliumylidene precursor is marked with *.

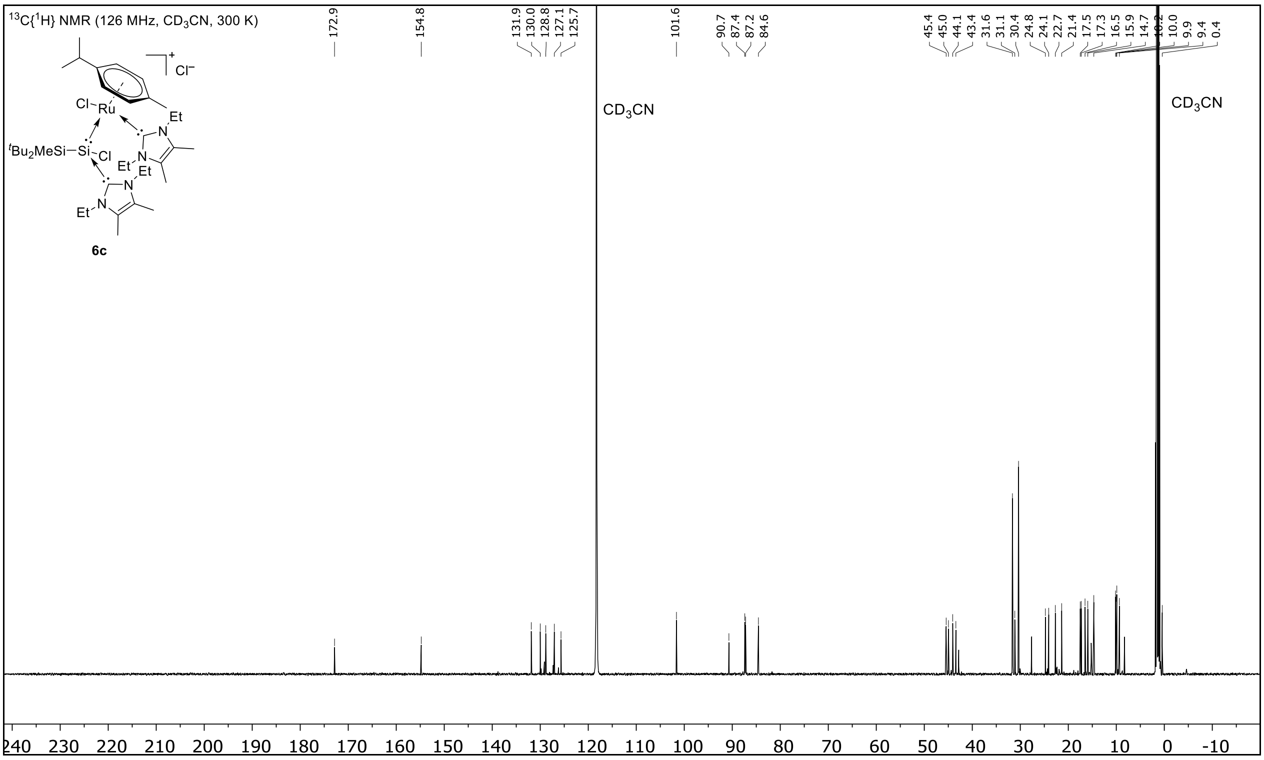

Figure S32 $^{13}C\{^{1}H\}$ NMR spectrum of [$^{t}Bu_2MeSi$–Si(IEt_2Me_2)Cl→Ru(*p*-cymene)Cl(IEt_2Me_2)]Cl (**6c**) in CD_3CN at 300 K.

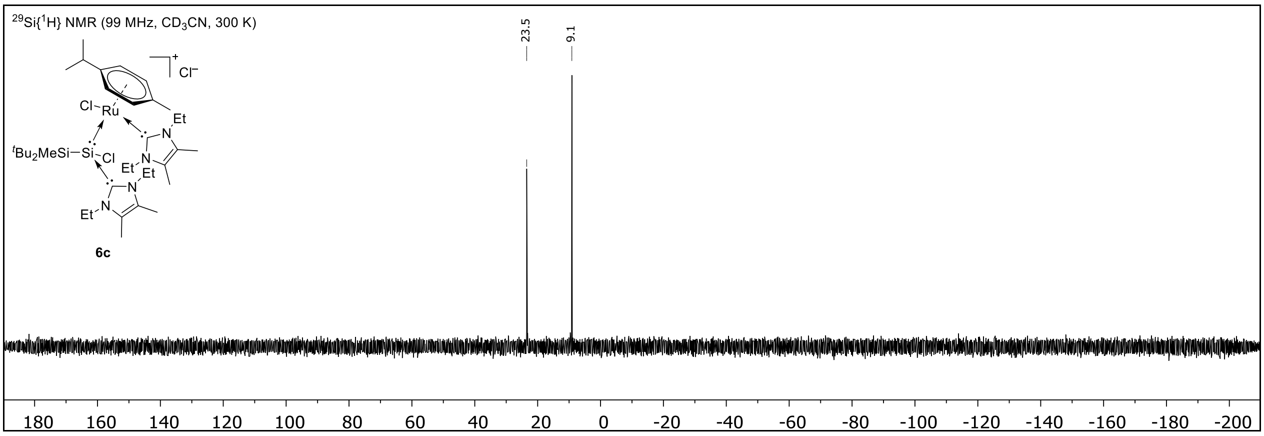

Figure S33 $^{29}Si\{^1H\}$ NMR spectrum of [$^tBu_2MeSi–Si(IEt_2Me_2)Cl \rightarrow Ru(p\text{-cymene})Cl(IEt_2Me_2)$]Cl (**6c**) in CD_3CN at 300 K.

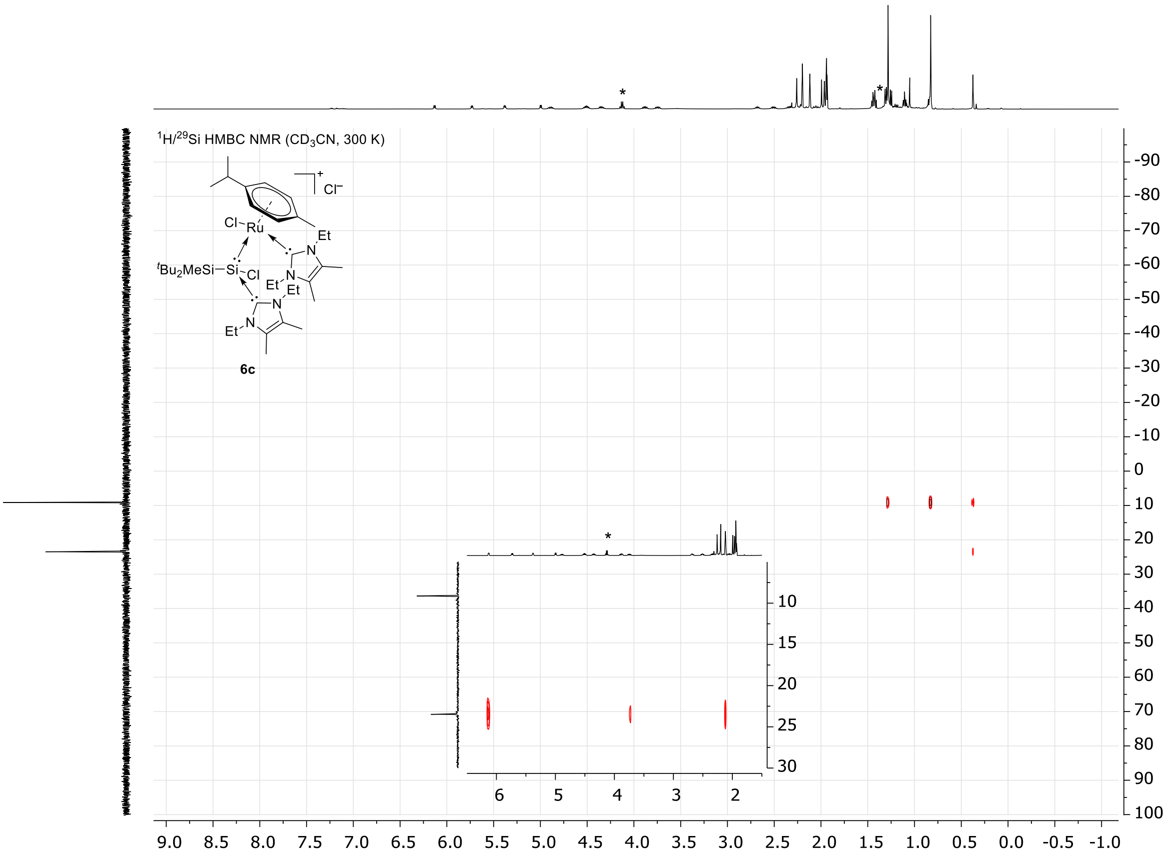

Figure S34 $^1H/^{29}Si$ HMBC NMR spectrum of [$^tBu_2MeSi–Si(IEt_2Me_2)Cl \rightarrow Ru(p\text{-cymene})Cl(IEt_2Me_2)$]Cl (**6c**) in CD_3CN at 300 K. Imidazolium chloride [$IEt_2Me_2 \cdot HCl$] from the silyliumylidene precursor is marked with *.

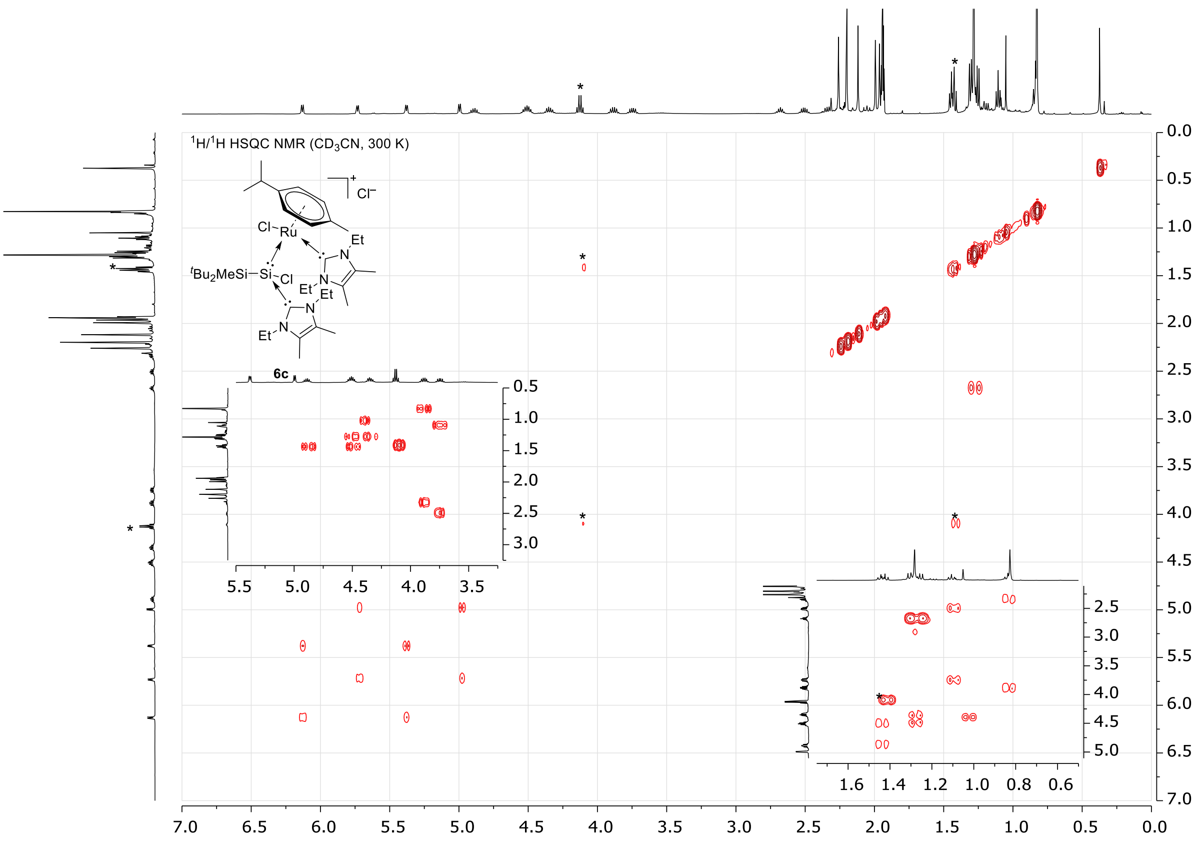

Figure S35 $^{1}H/^{1}H$ COSY NMR spectrum of [$^{t}Bu_2MeSi$–Si(IEt_2Me_2)Cl→Ru(*p*-cymene)Cl(IEt_2Me_2)]Cl (**6c**) in CD_3CN at 300 K. Imidazolium chloride [IEt_2Me_2·HCl] from the silyliumylidene precursor is marked with *.

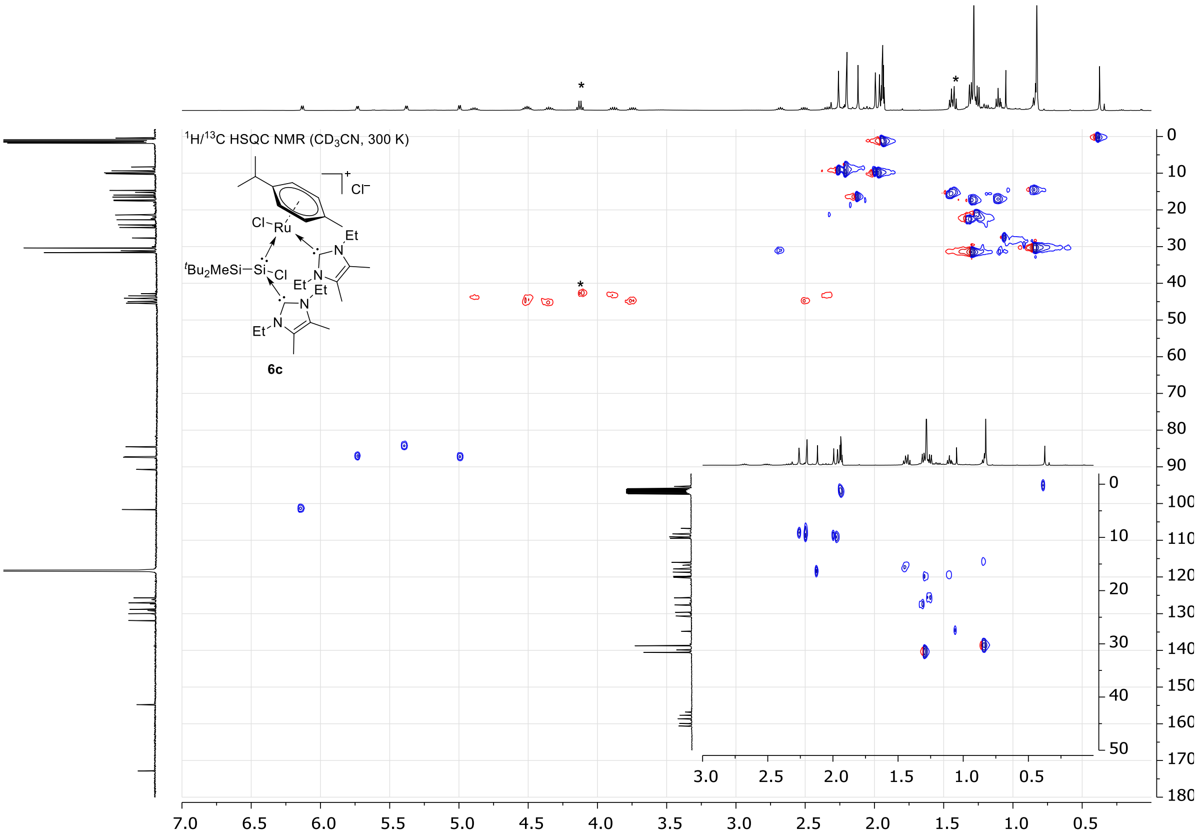

Figure S36 $^{1}H/^{13}C$ HSQC NMR spectrum of [$^{t}Bu_2MeSi$–Si(IEt_2Me_2)Cl→Ru(*p*-cymene)Cl(IEt_2Me_2)]Cl (**6c**) in CD_3CN at 300 K. Imidazolium chloride [IEt_2Me_2·HCl] from the silyliumylidene precursor is marked with *.

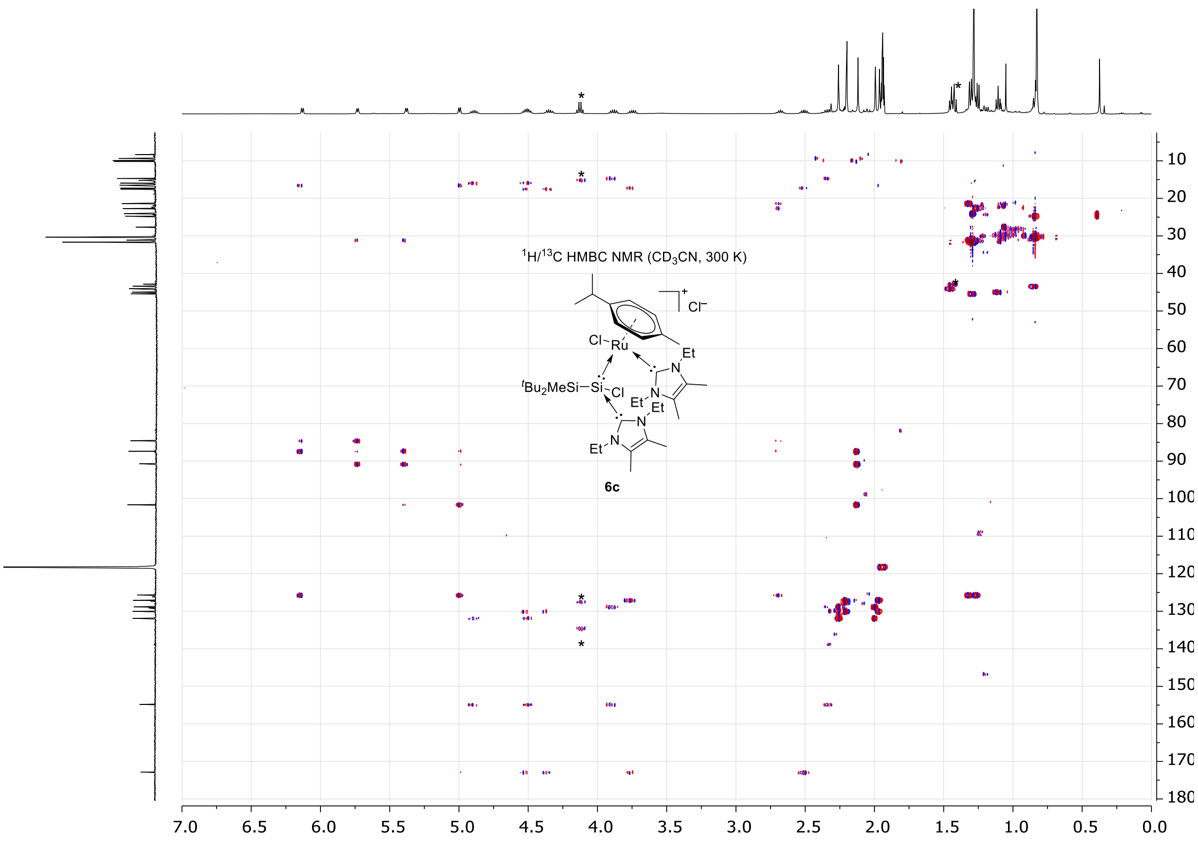

Figure S37 ^{1}H/^{13}C HMBC NMR spectrum of [^{t}Bu$_2$MeSi–Si(IEt$_2$Me$_2$)Cl→Ru(*p*-cymene)Cl(IEt$_2$Me$_2$)]Cl (**6c**) in CD_3CN at 300 K. Imidazolium chloride [IEt$_2$Me$_2$·HCl] from the silyliumylidene precursor is marked with *.

1.4 General Synthetic Procedure for [R–Si(NHC)Cl→RhCl(NHC)(Cp*)]Cl

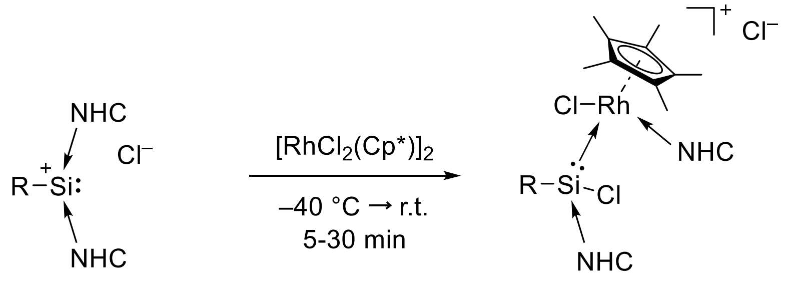

	R	NHC
**5a**	^{t}Bu$_3$Si	IMe$_4$
**5b**	^{t}Bu$_2$MeSi	IMe$_4$
**5c**	^{t}Bu$_2$MeSi	IEt$_2$Me$_2$

	R	NHC
**7a**	^{t}Bu$_3$Si	IMe$_4$
**7b**	^{t}Bu$_2$MeSi	IMe$_4$
**7c**	^{t}Bu$_2$MeSi	IEt$_2$Me$_2$

Silyliumylidene chloride [R$_3$Si–Si(NHC)$_2$]Cl (**5a-c**) (1.0 eq) and [RhCl$_2$(Cp*)]$_2$ (0.5 eq) were mixed, cooled to –40 °C and pre-cooled (–40 °C) MeCN (3-10 mL) was added. The reaction mixtures were stirred at –40 °C until all starting material had dissolved (5-30 minutes) and then warmed to room temperature. The solutions were quickly concentrated under reduced pressure to about 1-3 mL. A mixture of toluene and Et$_2$O (1:1, 5-15 mL) was added and the orange-red solutions were stored at –35 °C for 3-10 days. The formed crystals/precipitate was collected by filtration, washed with benzene or toluene (2 mL) and Et$_2$O (2×2 mL) and after drying under vacuum the complexes **7a-b** were isolated as orange to orange-red air- and moisture-sensitive solids/crystals. In the case of **7c** (R = Si^{t}Bu$_2$Me, NHC = IEt$_2$Me$_2$) the product could only be detected and assigned *via* ^{29}Si NMR spectroscopy (*cf.* Figure S56) due to competing side reactions.

1.4.1 [^{t}Bu$_3$Si–Si(IMe$_4$)Cl→RhCl(IMe$_4$)(Cp*)]X (7a, X = Cl, OTf)

(a) [^{t}Bu$_3$Si–Si(IMe$_4$)Cl→RhCl(IMe$_4$)(Cp*)]Cl (7a)

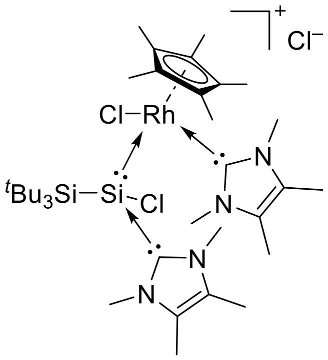

7a
$C_{36}H_{66}Cl_3N_4RhSi_2$
820.38 g/mol

Batch size: **5a**: 100.0 mg, 195.6 µmol, 1.0 eq.
[RhCl$_2$(Cp*)]$_2$: 60.4 mg, 97.8 µmol, 0.5 eq.

Yield: 112.2 mg, (136.8 µmol, 70%) as an orange solid.

SC-XRD: Suitable single crystals were obtained by slow diffusion of Et$_2$O into a concentrated solution of **7a** in MeCN at –35 °C.

^{1}H NMR (500 MHz, CD$_3$CN, 300 K): δ [ppm] = 4.18 (s, 3H, N$_{NHC→Si}$C*H*$_3$), 3.78 (s, 3H, N$_{NHC→Rh}$C*H*$_3$), 3.14 (s, 3H, N$_{NHC→Rh}$C*H*$_3$), 3.06 (s, 3H, N$_{NHC→Si}$C*H*$_3$), 2.20 (s, 3H, C$_{NHC→Si}$C*H*$_3$), 2.14 (s, 3H, C$_{NHC→Rh}$C*H*$_3$), 1.87 (s, 3H, C$_{NHC→Rh}$C*H*$_3$), 1.82 (s, 3H, C$_{NHC→Si}$C*H*$_3$), 1.43 (s, 15H, C$_5$(C*H*$_3$)$_5$), 1.25 (s, 27H, Si(C(C*H*$_3$)$_3$)$_3$).

^{13}C{^{1}H} NMR (126 MHz, CD$_3$CN, 300 K): δ [ppm] = 165.7 (d, $^1J_{Rh–C}$ = 60.2 Hz, N*C*$_{NHC→Rh}$N), 155.2 (d, $^2J_{Rh–C}$ = 2.7 Hz, N*C*$_{NHC→Si}$N), 131.2 (*C*$_{NHC→Si}$CH$_3$), 130.2 (*C*$_{NHC→Rh}$CH$_3$), 129.1 (*C*$_{NHC→Si}$CH$_3$), 128.6 (*C*$_{NHC→Rh}$CH$_3$), 102.8 (d, $^1J_{Rh–C}$ = 4.7 Hz *C*$_5$(CH$_3$)$_5$), 38.1 (N$_{NHC→Si}$*C*H$_3$), 37.7 (N$_{NHC→Rh}$*C*H$_3$), 36.6 (N$_{NHC→Rh}$*C*H$_3$), 36.5 (N$_{NHC→Si}$*C*H$_3$), 33.2 (Si(C(*C*H$_3$)$_3$)$_3$), 26.3 (Si(*C*(CH$_3$)$_3$)$_3$), 9.8 (C$_5$(*C*H$_3$)$_5$), 9.8 (C$_{NHC→Rh}$*C*H$_3$), 9.7 (C$_{NHC→Si}$*C*H$_3$), 9.6 (C$_{NHC→Rh}$*C*H$_3$), 8.5 (C$_{NHC→Si}$*C*H$_3$).

^{29}Si{^{1}H} NMR (99 MHz, CD$_3$CN, 300 K): δ [ppm] = 28.6 (d, $^2J_{Si–Rh}$ = 2.6 Hz, *Si*^{t}Bu$_3$), 23.5 (d, $^1J_{Si–Rh}$ = 63.9 Hz, *Si*Rh).

EA: $C_{36}H_{66}Cl_3N_4RhSi_2$ calculated [%]: C (52.71), H (8.11), N (6.83).
measured [%]: C (53.02), H (8.21), N (7.16).

M.P.: 140-141 °C (decomposition, color change to black).

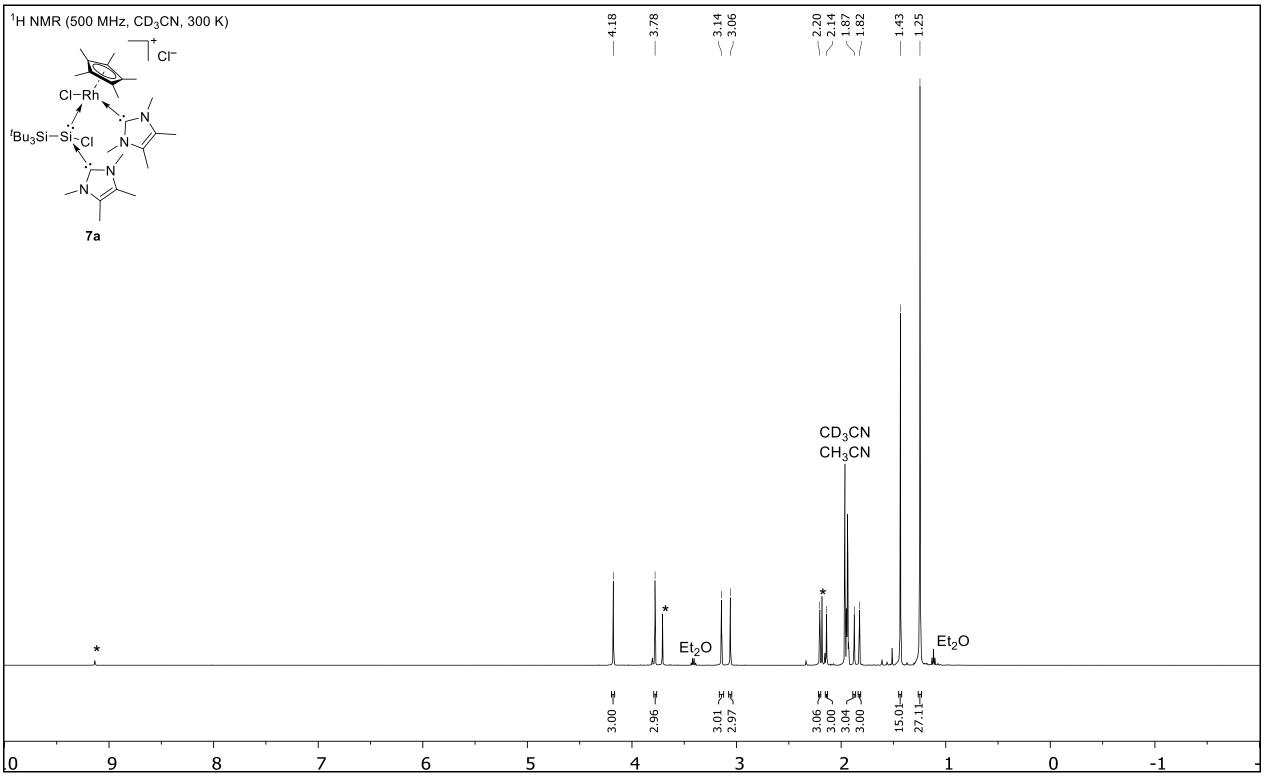

Figure S38 ^{1}H NMR spectrum of [$^{t}Bu_3Si$–Si(IMe$_4$)Cl→RhCl(IMe$_4$)(Cp*)]Cl (**7a**) in CD_3CN at 300 K. Small amounts of residual imidazolium chloride from the precursor [IMe$_4$·HCl] are marked with *.

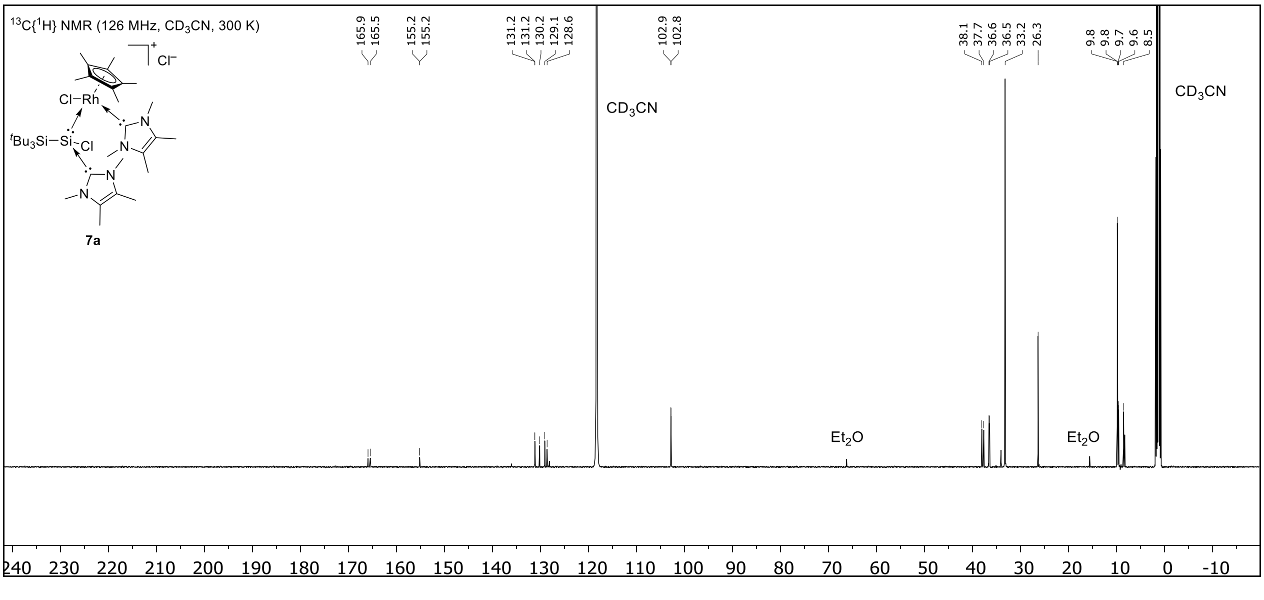

Figure S39 $^{13}C\{^{1}H\}$ NMR spectrum of [$^{t}Bu_3Si$–Si(IMe$_4$)Cl→RhCl(IMe$_4$)(Cp*)]Cl (**7a**) in CD_3CN at 300 K.

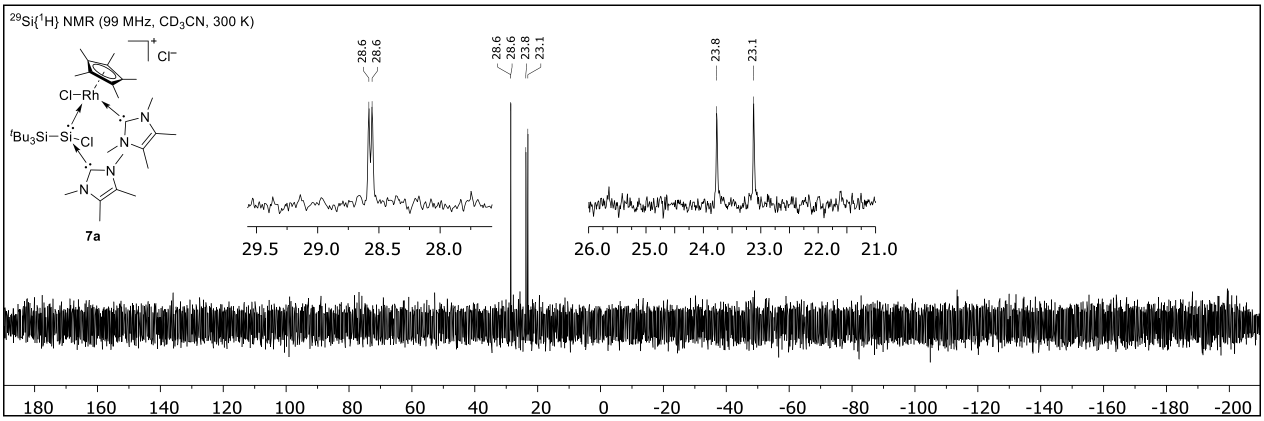

Figure S40 ^{29}Si{^{1}H} NMR spectrum of [^{t}Bu$_3$Si–Si(IMe$_4$)Cl→RhCl(IMe$_4$)(Cp*)]Cl (**7a**) in CD$_3$CN at 300 K.

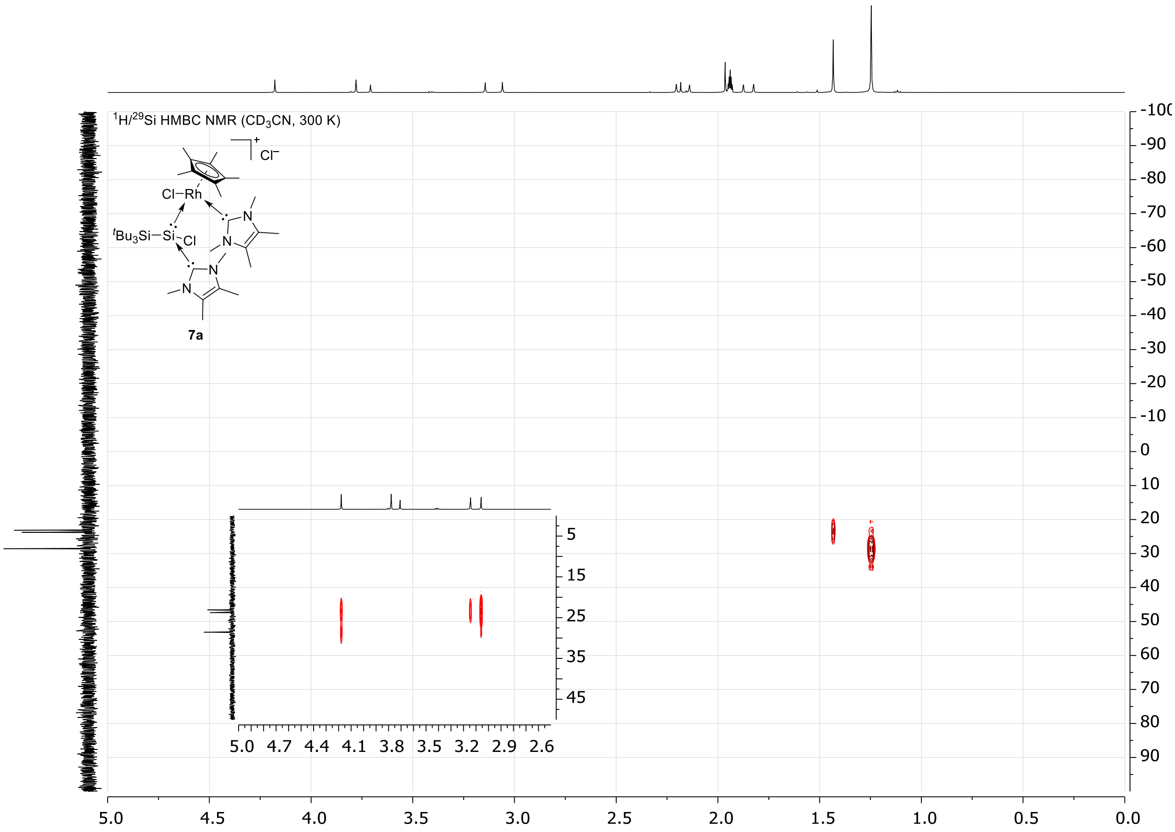

Figure S41 ^{1}H/^{29}Si HMBC NMR spectrum of [^{t}Bu$_3$Si–Si(IMe$_4$)Cl→RhCl(IMe$_4$)(Cp*)]Cl (**7a**) in CD$_3$CN at 300 K.

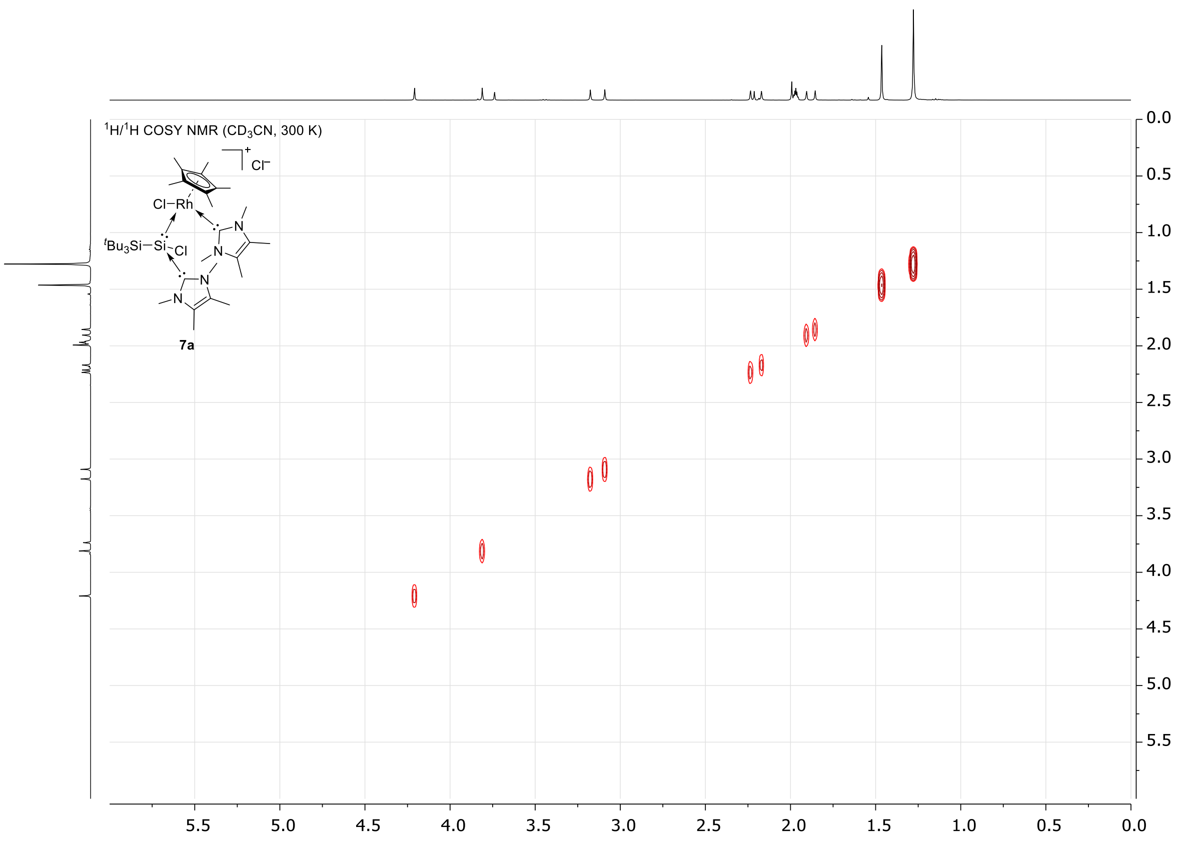

Figure S42 ^{1}H/^{1}H COSY NMR spectrum of [^{t}Bu$_3$Si–Si(IMe$_4$)Cl→RhCl(IMe$_4$)(Cp*)]Cl (**7a**) in CD$_3$CN at 300 K.

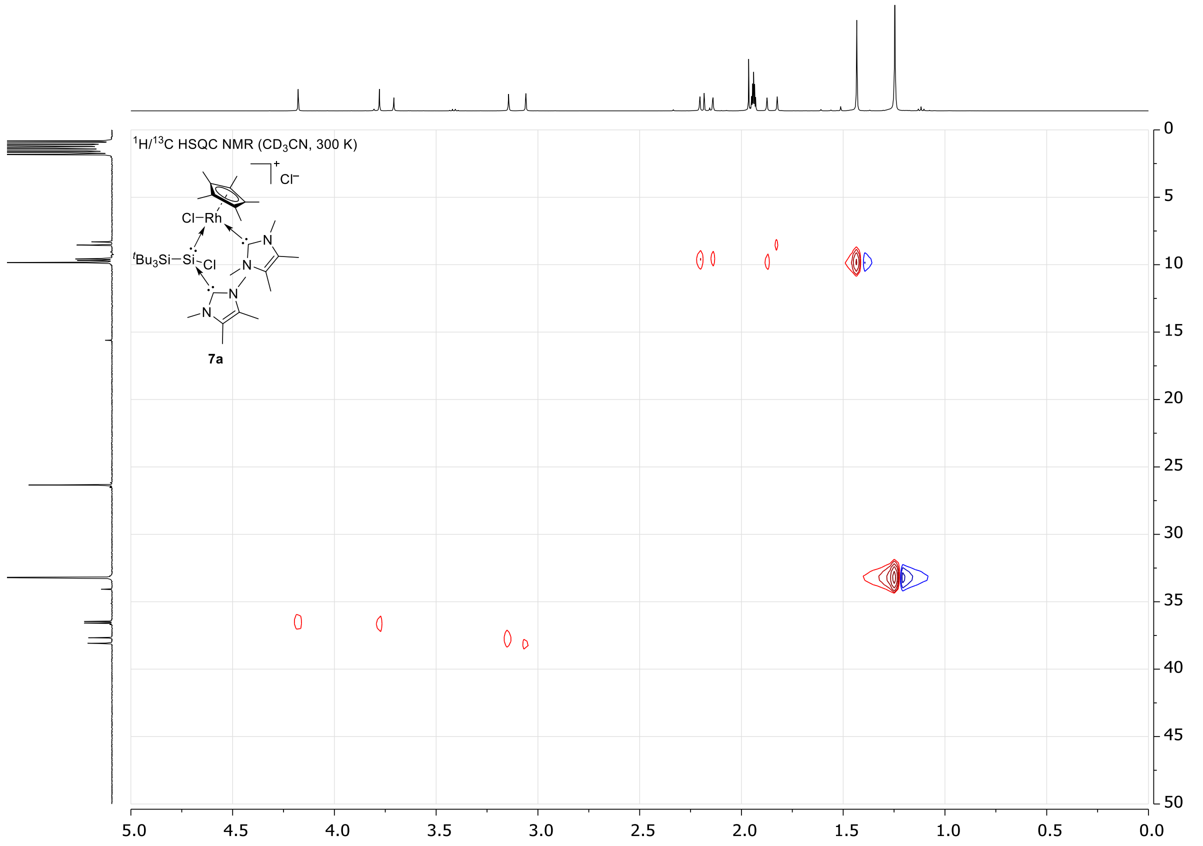

Figure S43 ^{1}H/^{13}C HSQC NMR spectrum of [^{t}Bu$_3$Si–Si(IMe$_4$)Cl→RhCl(IMe$_4$)(Cp*)]Cl (**7a**) in CD$_3$CN at 300 K.

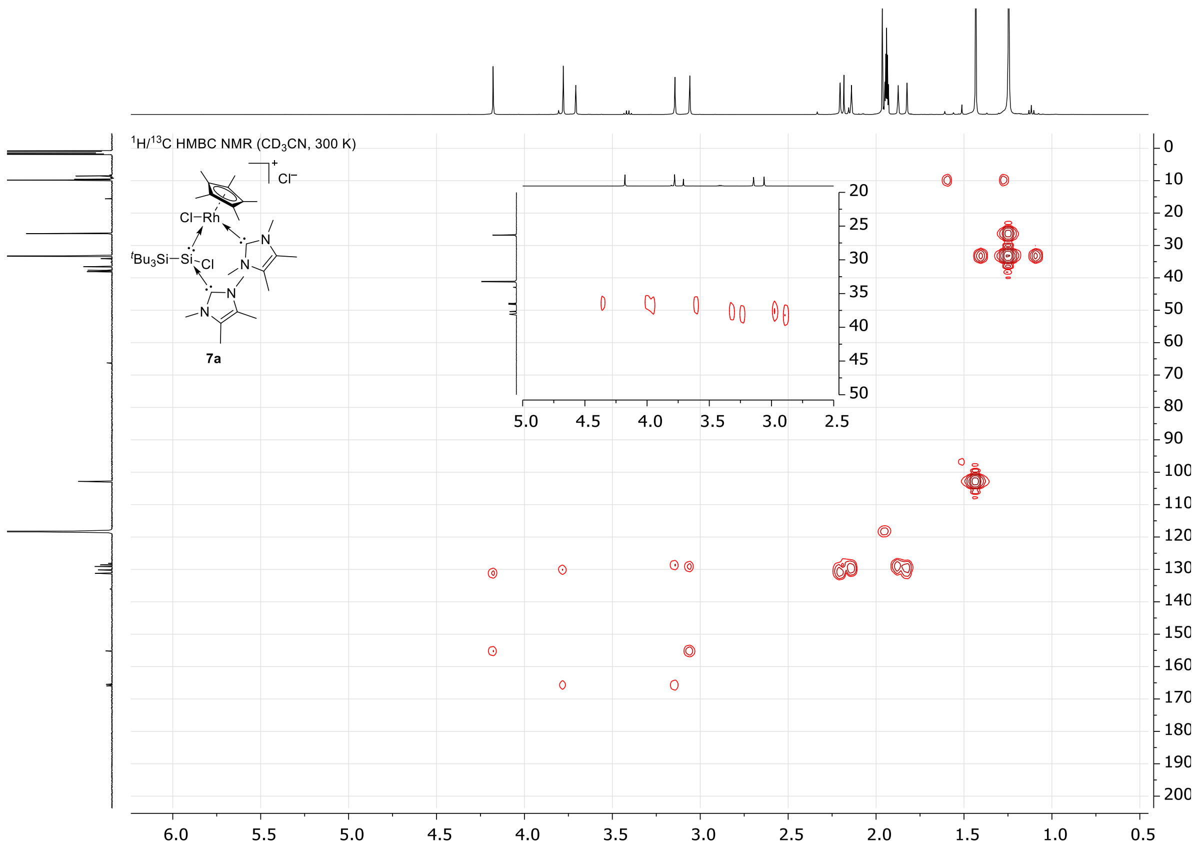

Figure S44 ^{1}H/^{13}C HMBC NMR spectrum of [^{t}Bu$_3$Si–Si(IMe$_4$)Cl→RhCl(IMe$_4$)(Cp*)]Cl (**7a**) in CD$_3$CN at 300 K.

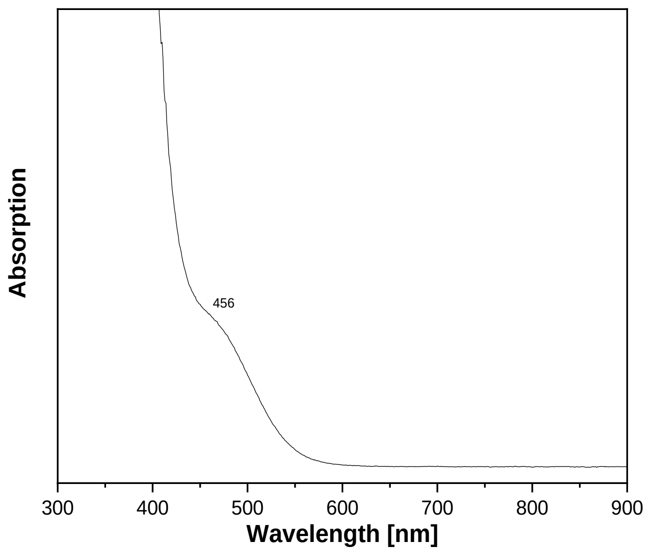

Figure S45 UV-Vis spectrum (300-900 nm) of [^{t}Bu$_3$Si–Si(IMe$_4$)Cl→RhCl(IMe$_4$)(Cp*)]Cl (**7a**) in toluene at room temperature (c = 5.0×10^{-4} mol/L). λ_{max} = 456 nm.

(b) [^{t}Bu$_3$Si–Si(IMe$_4$)Cl→RhCl(IMe$_4$)(Cp*)]OTf (7a-OTf)

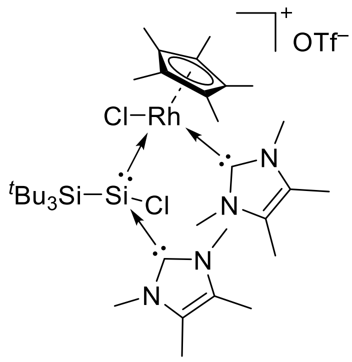

7a-OTf
$C_{37}H_{66}Cl_2F_3N_4O_3RhSSi_2$
933.99 g/mol

To determine whether insertion into the Rh–Cl bond takes place or if the reaction occurs from an NHC-stabilized chlorosilylene, we synthesized **5a-OTf** (*vide supra*) and utilized it in a reaction similar to that described in section 1.4 for the synthesis of **7a**.

[^{t}Bu$_3$Si–Si(IMe$_4$)$_2$]OTf (**5a-OTf**) (30.0 mg, 48.0 µmol, 1.0 eq) was mixed with [RhCl$_2$(Cp*)]$_2$ (14.8 mg, 24.0 µmol, 0.5 eq) and cooled to –35 °C. Pre-cooled (–35 °C) CD$_3$CN (0.5 mL) was added and the suspension was stirred for 5 minutes and then transferred to a *J*-Young NMR tube.

^{1}H NMR (500 MHz, CD$_3$CN, 300 K): δ [ppm] = 4.17 (s, 3H, N$_{NHC→Si}$C*H*$_3$), 3.78 (s, 3H, N$_{NHC→Rh}$C*H*$_3$), 3.13 (s, 3H, N$_{NHC→Rh}$C*H*$_3$), 3.06 (s, 3H, N$_{NHC→Si}$C*H*$_3$), 2.19 (s, 3H, C$_{NHC→Si}$C*H*$_3$), 2.14 (s, 3H, C$_{NHC→Rh}$C*H*$_3$), 1.87 (s, 3H, C$_{NHC→Rh}$C*H*$_3$), 1.82 (s, 3H, C$_{NHC→Si}$C*H*$_3$), 1.44 (s, 15H, C$_5$(C*H*$_3$)$_5$), 1.25 (s, 27H, Si(C(C*H*$_3$)$_3$)$_3$).

***Note:** ^{1}H NMR data are the same as **7a**.*

^{13}C{^{1}H} NMR (126 MHz, CD$_3$CN, 300 K): δ [ppm] = 165.8 (d, $^1J_{Rh-C}$ = 60.0 Hz, N*C*$_{NHC→Rh}$N), 155.2 (d, $^2J_{Rh-C}$ = 2.7 Hz, N*C*$_{NHC→Si}$N), 131.1 (*C*$_{NHC→Si}$CH$_3$), 130.2 (*C*$_{NHC→Rh}$CH$_3$), 129.1 (*C*$_{NHC→Si}$CH$_3$), 128.6 (*C*$_{NHC→Rh}$CH$_3$), 122.1 (q, $^1J_{C-F}$ = 320.9 Hz, SO$_3$*C*F$_3$) 102.8 (d, $^1J_{Rh-C}$ = 4.8 Hz, *C*$_5$(CH$_3$)$_5$), 38.0 (N$_{NHC→Si}$*C*H$_3$), 37.6 (N$_{NHC→Rh}$*C*H$_3$), 36.6 (N$_{NHC→Rh}$*C*H$_3$), 36.4 (N$_{NHC→Si}$*C*H$_3$), 33.2 (Si(C(*C*H$_3$)$_3$)$_3$), 26.3 (Si(*C*(CH$_3$)$_3$)$_3$), 9.8 (C$_5$(*C*H$_3$)$_5$), 9.8 (C$_{NHC→Rh}$*C*H$_3$), 9.6 (C$_{NHC→Si}$*C*H$_3$), 9.5 (C$_{NHC→Rh}$*C*H$_3$), 8.5 (C$_{NHC→Si}$*C*H$_3$).

***Note: (a)** ^{13}C NMR data (for comparison, cf. Figure S47) are the same as **7a** except for the additional resonance corresponding to the triflate anion (122.1 ppm (q, $^1J_{C-F}$ = 320.9 Hz). **(b)** The resonance for the SO$_3$CF$_3$ group (quartet) partially overlaps with the resonance for CD$_3$CN.*

^{29}Si{^{1}H} NMR (99 MHz, CD_3CN, 300 K): δ [ppm] = 28.7 (d, $^{2}J_{Si–Rh}$ = 2.8 Hz, *Si*^{t}Bu$_3$), 23.4 (d, $^{1}J_{Si–Rh}$ = 63.8 Hz, *Si*Rh).

Note: *^{29}Si NMR (for comparison, cf. Figure S48) data are the same as **7a**.*

^{19}F{^{1}H} NMR (471 MHz, CD_3CN, 300 K): δ [ppm] = –79.3 (SO_3CF_3).

Note: *Only the resonance corresponding to a free triflate anion could be observed.*

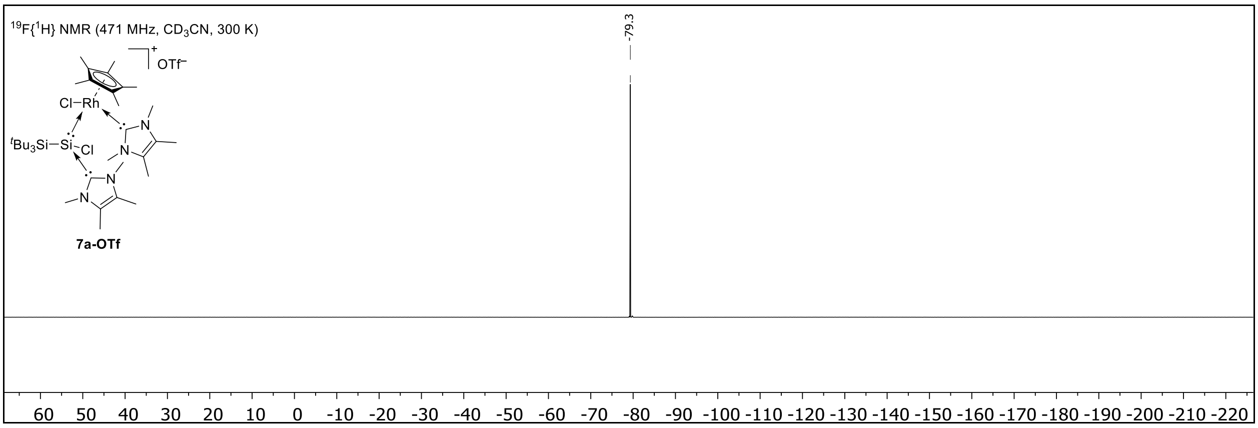

Figure S46 ^{19}F{^{1}H} NMR spectrum of [^{t}Bu$_3$Si–Si(IMe$_4$)Cl→RhCl(IMe$_4$)(Cp*)]OTf (**7a-OTf**) in CD_3CN at 300 K.

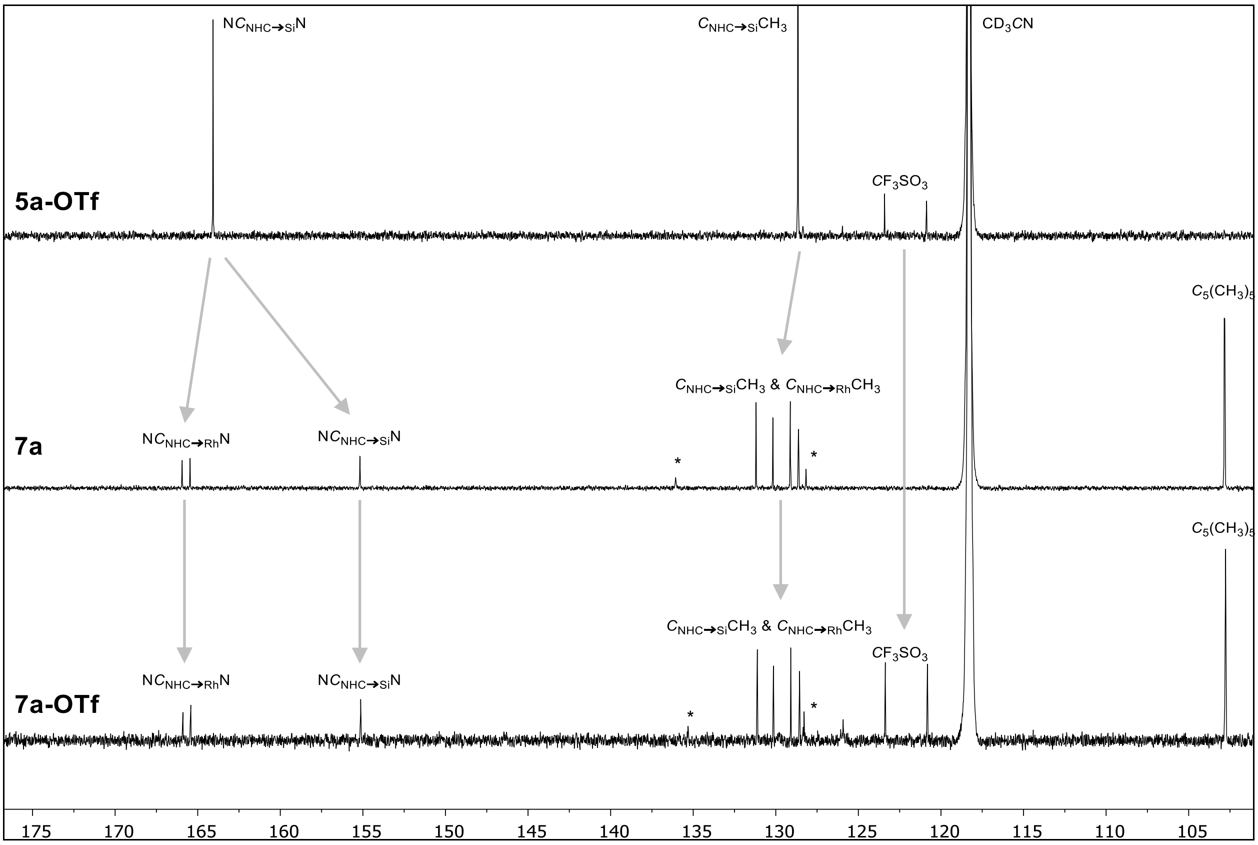

Figure S47 Section (180-100 ppm) of the ^{13}C{^{1}H} NMR spectra of **5a-OTf** (top), **7a** (middle) and **7a-OTf** (bottom) in CD_3CN at 300 K. Small amounts of residual imidazolium chloride [IMe$_4$·HCl] from the synthesis of **5a**/**5a-OTf** are marked with *.

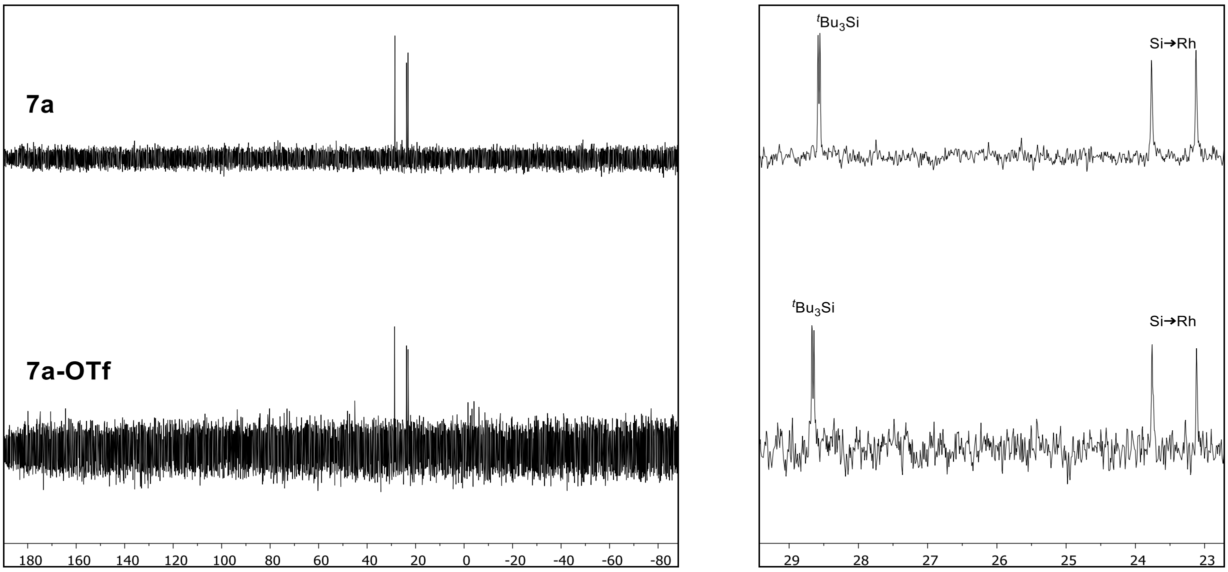

Figure S48 $^{29}Si\{^{1}H\}$ NMR spectrum of **7a** (top) and **7a-OTf** (bottom) in CD_3CN at 300 K.

1.4.2 [^{t}Bu$_2$MeSi–Si(IMe$_4$)Cl→RhCl(IMe$_4$)(Cp*)]Cl (7b)

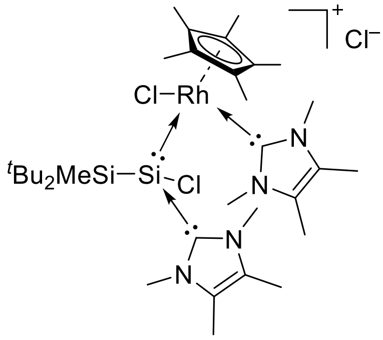

7b

$C_{33}H_{60}Cl_3N_4RhSi_2$

778.30 g/mol

Batch size: **5b**: 50.0 mg, 106.6 µmol, 1.0 eq.
[RhCl$_2$(Cp*)]$_2$: 32.9 mg, 53.3 µmol, 0.5 eq.

Yield: 51.5 mg (66.2 µmol, 62%) as an orange-red solid.

SC-XRD: Suitable single crystals were obtained by slow diffusion of Et$_2$O into a concentrated solution of **7b** in MeCN at –35 °C.

^{1}H NMR (500 MHz, CD$_3$CN, 300 K): δ [ppm] = 4.15 (s, 3H, N$_{NHC\to Si}$CH_3), 3.79 (s, 3H, N$_{NHC\to Rh}$CH_3), 3.15 (s, 3H, N$_{NHC\to Rh}$CH_3), 3.11 (s, 3H, N$_{NHC\to Si}$CH_3), 2.22 (s, 3H, C$_{NHC\to Si}$CH_3), 2.13 (s, 3H, C$_{NHC\to Rh}$CH_3), 1.86 (s, 3H, C$_{NHC\to Rh}$CH_3), 1.84 (s, 3H, C$_{NHC\to Si}$CH_3), 1.47 (s, 15H, C$_5$(CH_3)$_5$), 1.21 (s, 9H, Si(C(CH_3)$_3$)$_2$CH$_3$), 0.74 (s, 9H, Si(C(CH_3)$_3$)$_2$CH$_3$), 0.41 (s, 3H, Si(C(CH$_3$)$_3$)$_2$CH_3).

^{13}C{^{1}H} NMR (126 MHz, CD$_3$CN, 300 K): δ [ppm] = 165.9 (d, $^1J_{Rh–C}$ = 59.2 Hz, N$C_{NHC\to Rh}$N) 154.9 (d, $^2J_{Rh–C}$ = 2.5 Hz, N$C_{NHC\to Si}$N), 130.8 ($C_{NHC\to Si}$CH$_3$), 130.0 ($C_{NHC\to Rh}$CH$_3$), 129.2 ($C_{NHC\to Si}$CH$_3$), 128.4 ($C_{NHC\to Rh}$CH$_3$), 102.3 (d, $^1J_{Rh–C}$ = 4.8 Hz, C_5(CH$_3$)$_5$), 38.4 (N$_{NHC\to Rh}$CH$_3$), 36.6 (N$_{NHC\to Si}$CH$_3$), 36.6 (N$_{NHC\to Rh}$CH$_3$), 35.8 (N$_{NHC\to Si}$CH$_3$), 31.3 (Si(C(CH$_3$)$_3$)$_2$CH$_3$), 29.9 (Si(C(CH$_3$)$_3$)$_2$CH$_3$), 24.7 (Si(C(CH$_3$)$_3$)$_2$CH$_3$), 24.1 (Si(C(CH$_3$)$_3$)$_2$CH$_3$), 9.9 (C$_{NHC\to Rh}$$CH_3$), 9.7 (C$_5$($CH_3$)$_5$), 9.6 (C$_{NHC\to Rh}$$CH_3$), 9.5 (C$_{NHC\to Si}$$CH_3$), 8.3 (C$_{NHC\to Si}$$CH_3$), –0.8 (Si(C(CH$_3$)$_3$)$_2$$CH_3$).

^{29}Si{^{1}H} NMR (99 MHz, CD$_3$CN, 300 K): δ [ppm] = 23.9 (d, $^1J_{Si–Rh}$ = 62.1 Hz, *Si*Rh), 11.9 (d, $^2J_{Si–Rh}$ = 1.3 Hz, *Si*^{t}Bu$_2$Me).

EA: $C_{33}H_{60}Cl_3N_4RhSi_2$ calculated [%]: C (50.93), H (7.77), N (7.20).
measured [%]: C (51.19), H (7.92), N (7.56).

M.P.: 138-139 °C (decomposition, change to red-brown oil).

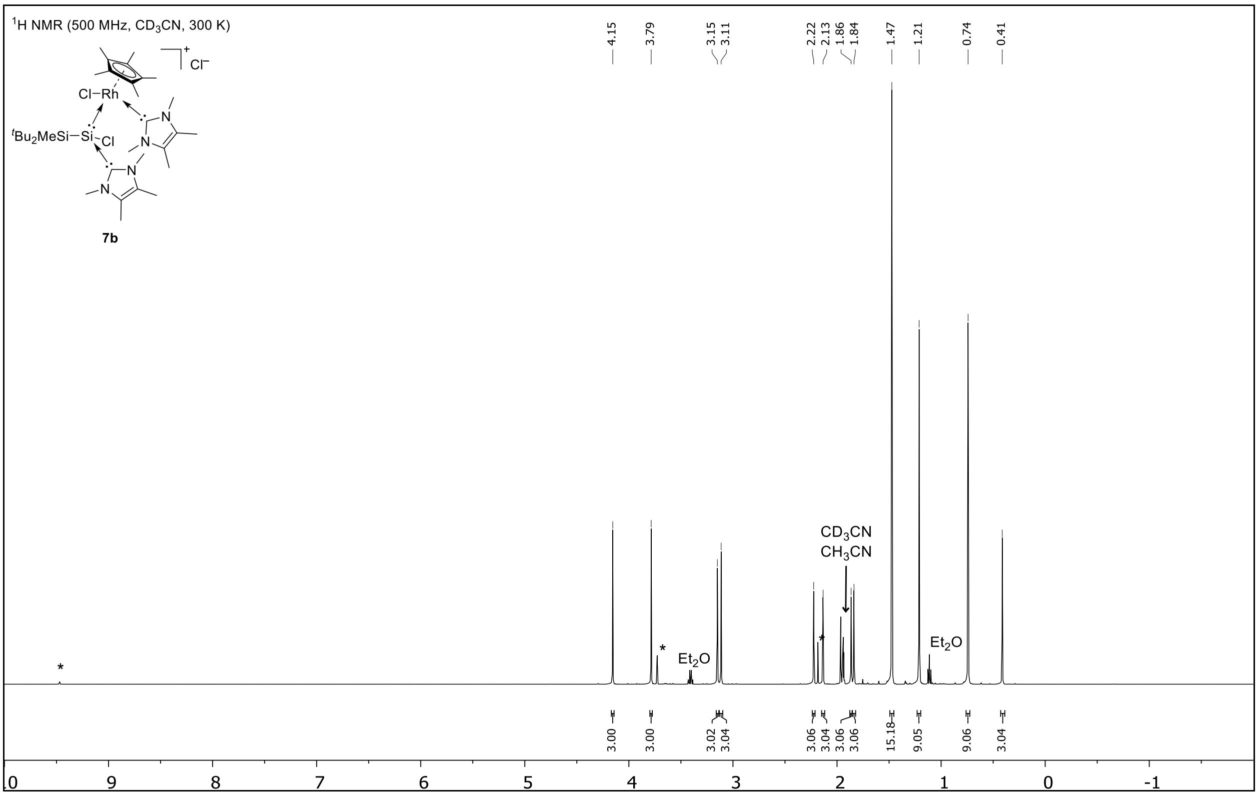

Figure S49 ^{1}H NMR spectrum of [^{t}Bu$_2$MeSi–Si(IMe$_4$)Cl→RhCl(IMe$_4$)(Cp*)]Cl (**7b**) in CD_3CN at 300 K. Small amounts of residual imidazolium chloride from the precursor [IMe$_4$·HCl] are marked with *.

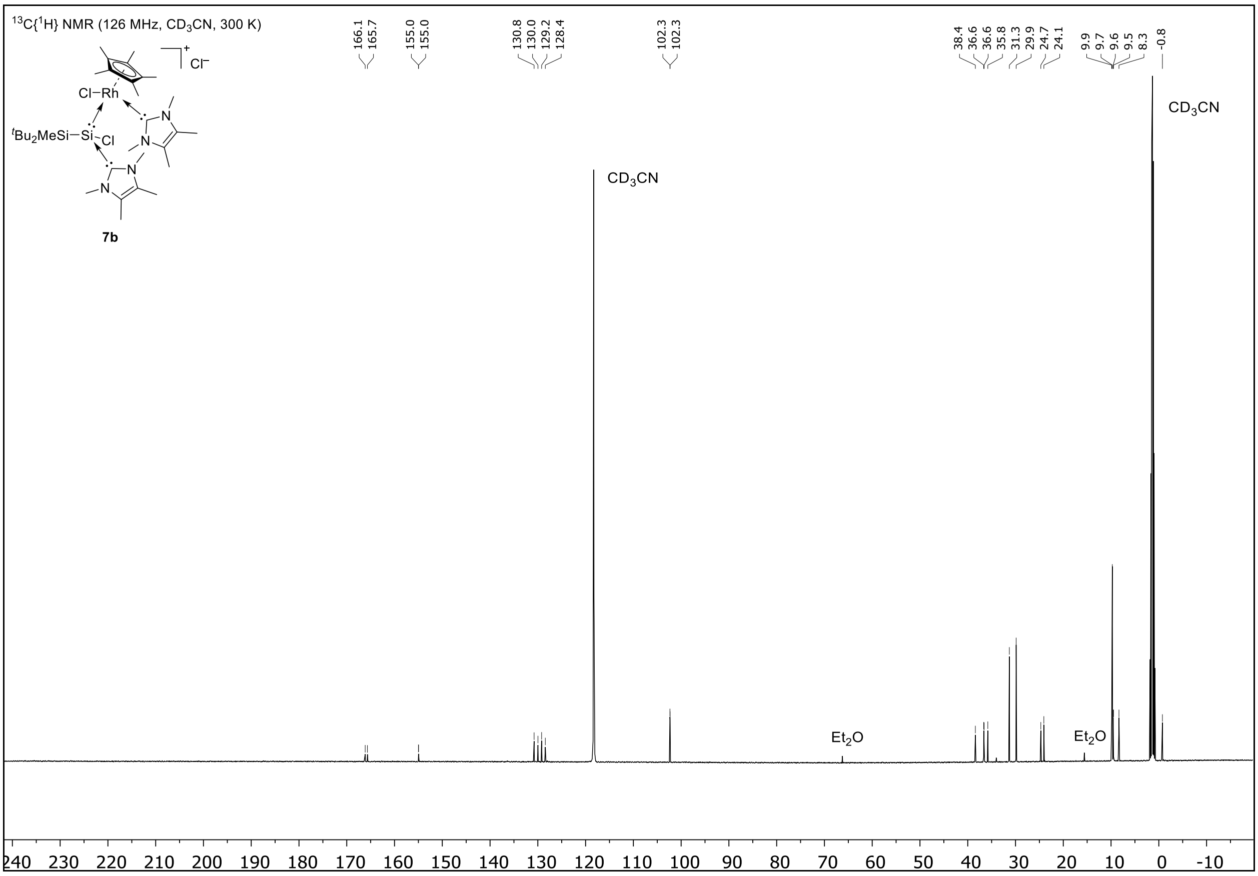

Figure S50 ^{13}C{^{1}H} NMR spectrum of [^{t}Bu$_2$MeSi–Si(IMe$_4$)Cl→RhCl(IMe$_4$)(Cp*)]Cl (**7b**) in CD_3CN at 300 K.

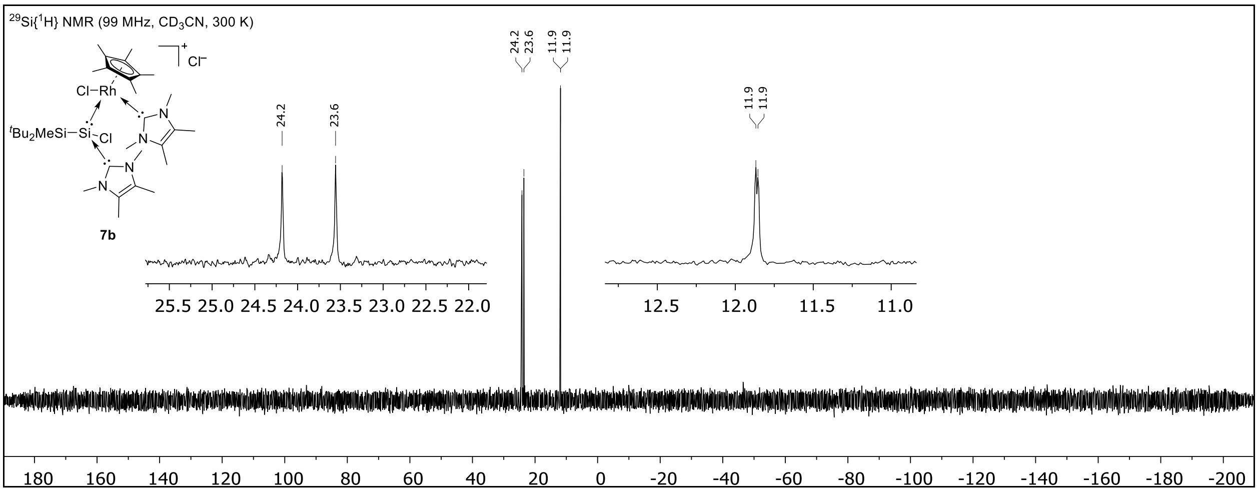

Figure S51 $^{29}Si\{^1H\}$ NMR spectrum of [^{t}Bu$_2$MeSi–Si(IMe$_4$)Cl→RhCl(IMe$_4$)(Cp*)]Cl (**7b**) in CD_3CN at 300 K.

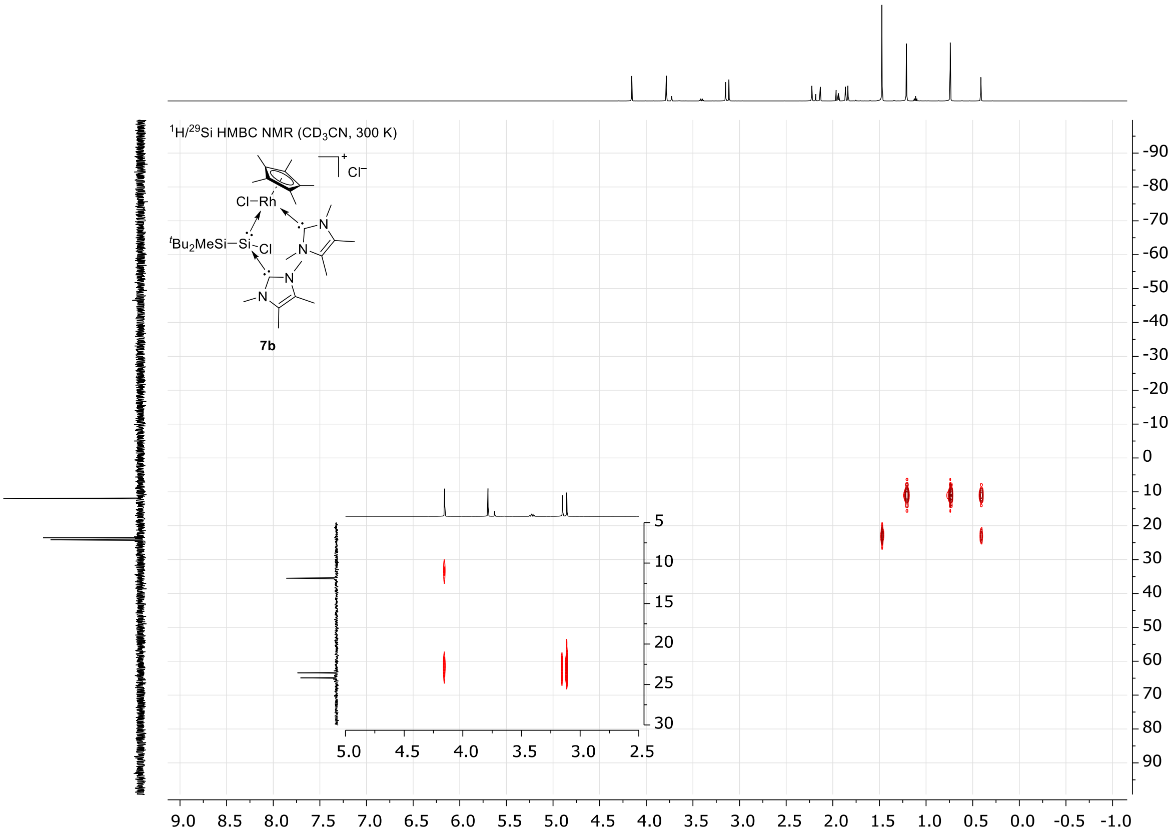

Figure S52 $^1H/^{29}Si$ HMBC NMR spectrum of [^{t}Bu$_2$MeSi–Si(IMe$_4$)Cl→RhCl(IMe$_4$)(Cp*)]Cl (**7b**) in CD_3CN at 300 K.

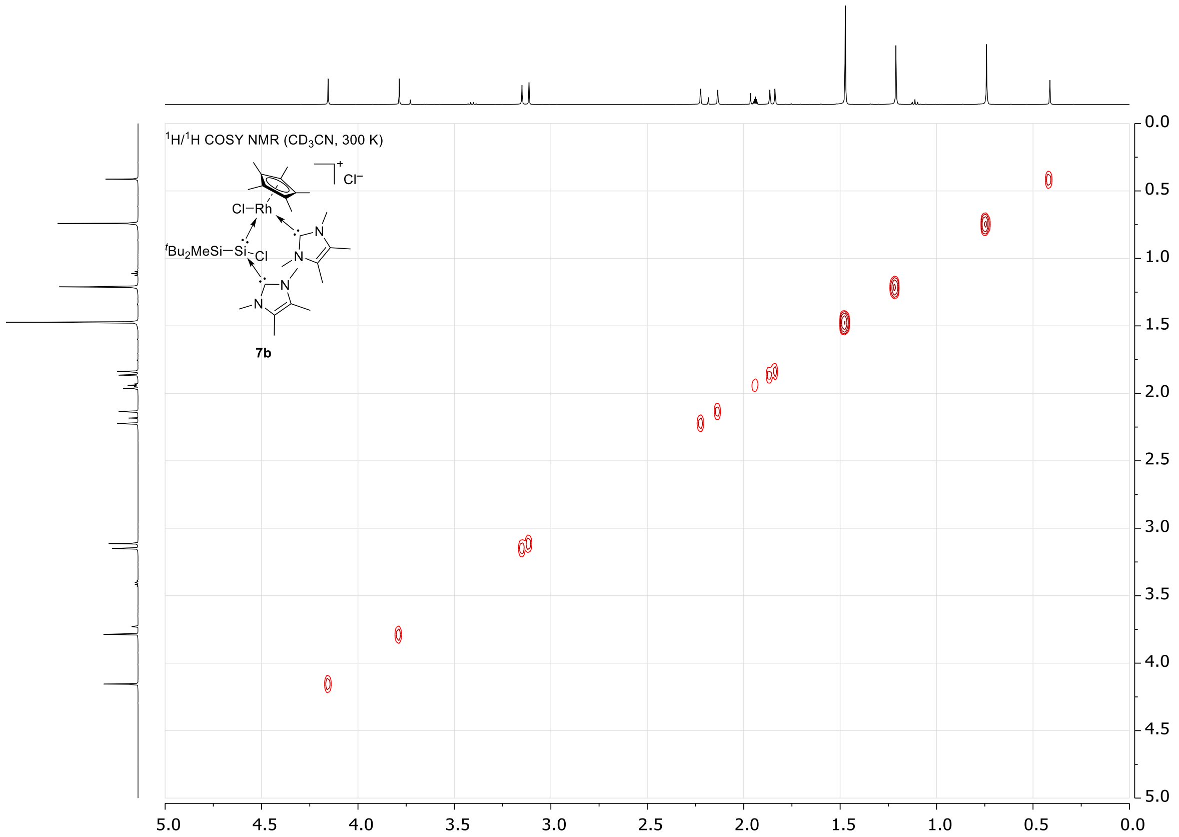

Figure S53 $^{1}H/^{1}H$ COSY NMR spectrum of [$^{t}Bu_2MeSi–Si(IMe_4)Cl \rightarrow RhCl(IMe_4)(Cp^*)$]Cl (**7b**) in CD_3CN at 300 K.

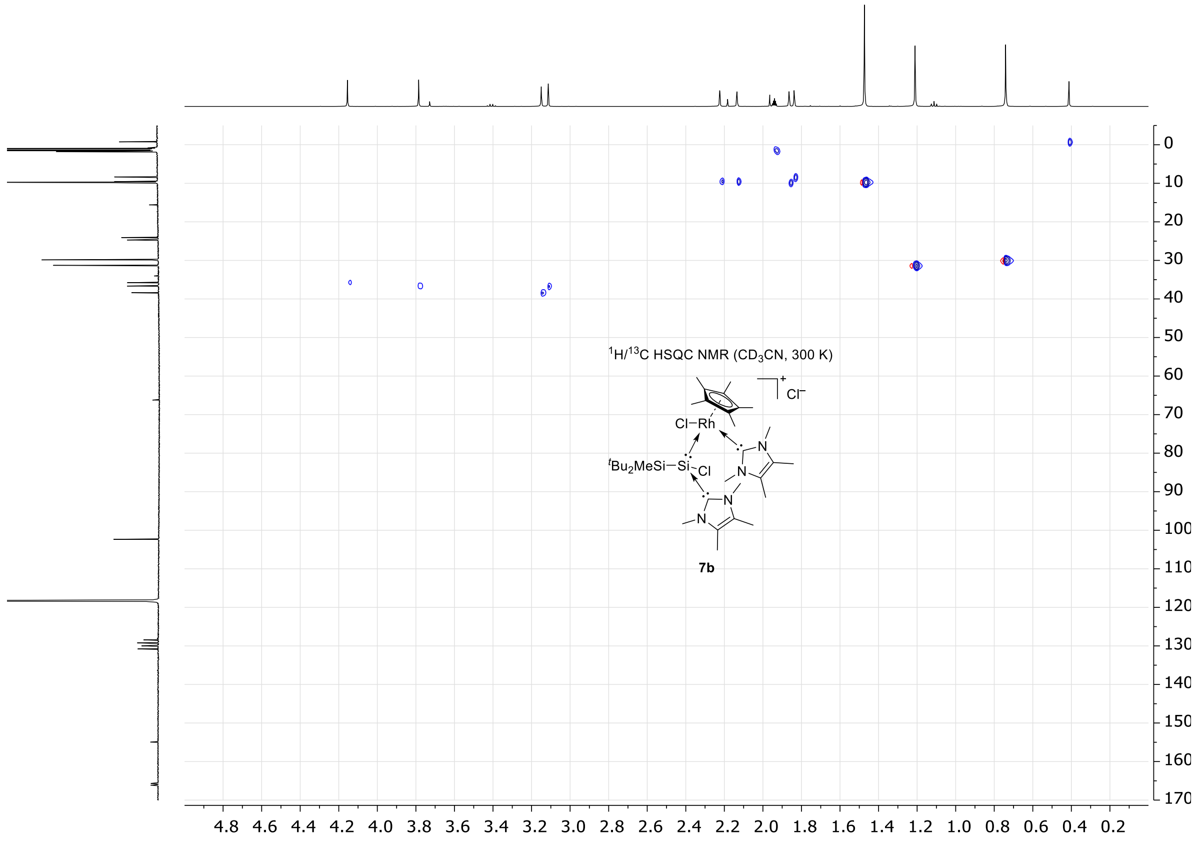

Figure S54 $^{1}H/^{13}C$ HSQC NMR spectrum of [$^{t}Bu_2MeSi–Si(IMe_4)Cl \rightarrow RhCl(IMe_4)(Cp^*)$]Cl (**7b**) in CD_3CN at 300 K.

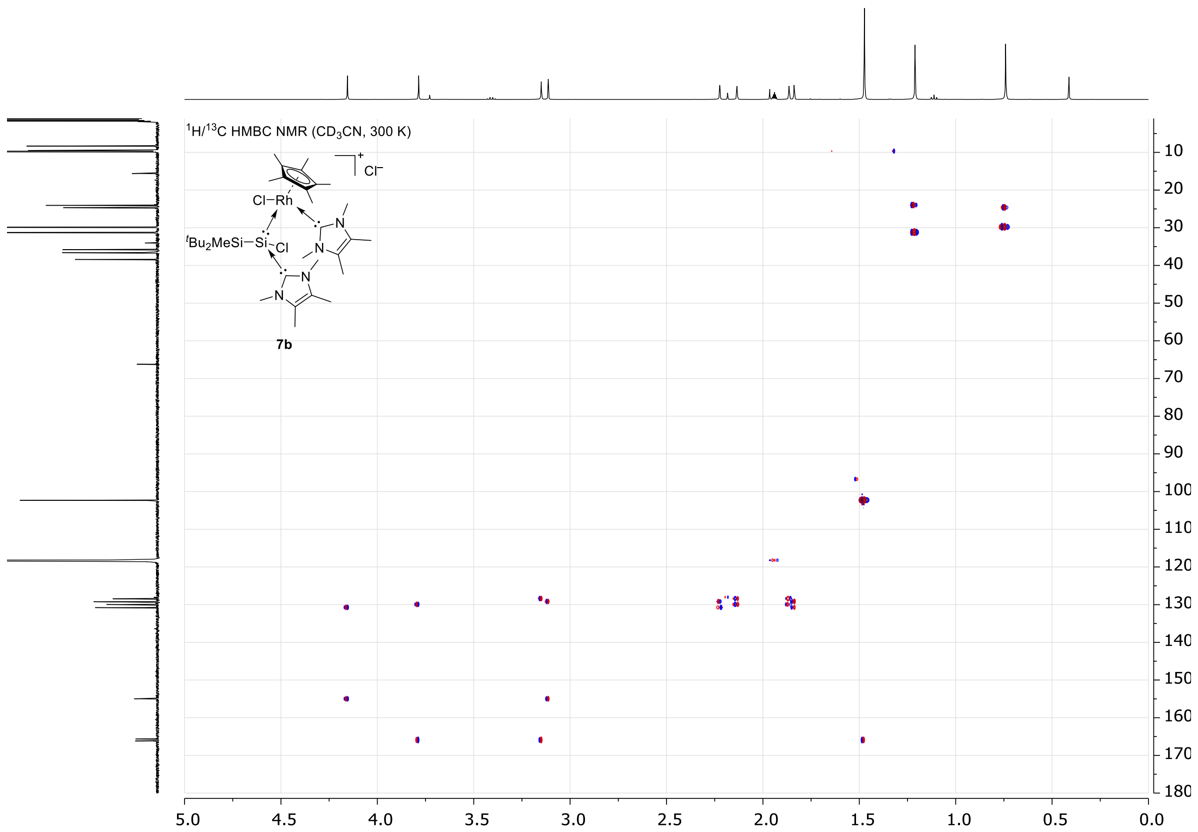

Figure S55 $^1H/^{13}C$ HMBC NMR spectrum of [$^tBu_2MeSi$–Si(IMe_4)Cl→RhCl(IMe_4)(Cp*)]Cl (**7b**) in CD_3CN at 300 K.

1.4.3 [^{t}Bu$_2$MeSi–Si(IEt$_2$Me$_2$)Cl→RhCl(IEt$_2$Me$_2$)(Cp*)]Cl (7c)

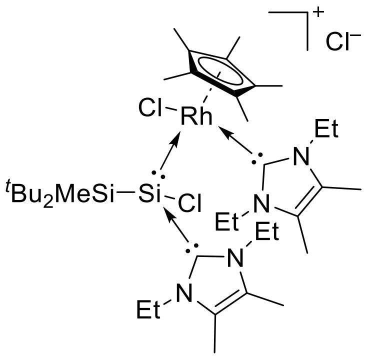

7c

$C_{37}H_{72}Cl_3N_4RhSi_2$

838.44

Synthesis of complex **7c** was attempted in the same fashion as the other complexes. However, multiple reaction products were observed upon NMR analysis. The desired complex is only present with 35-40% in the reaction mixture and purification attempts were unsuccessful. Temperature and solvent variation during the synthesis did not change the obtained results significantly except for more decomposition products at higher temperatures.

Interestingly, only the signals at 18.6 and 12.8 ppm (assigned to **7c**) in the ^{29}Si NMR exhibit coupling to a rhodium atom (s = ½). The other signals do not exhibit any coupling and hence formation of other coordination complexes with rhodium can be excluded. Chemical shifts and coupling constants are in line with the other complexes.

^{29}Si{^{1}H} NMR (99 MHz, CD_3CN, 300 K): δ [ppm] = 18.6 (d, $^{1}J_{Si–Rh}$ = 65.8 Hz), 12.8 (d, $^{2}J_{Si–Rh}$ = 1.9 Hz).

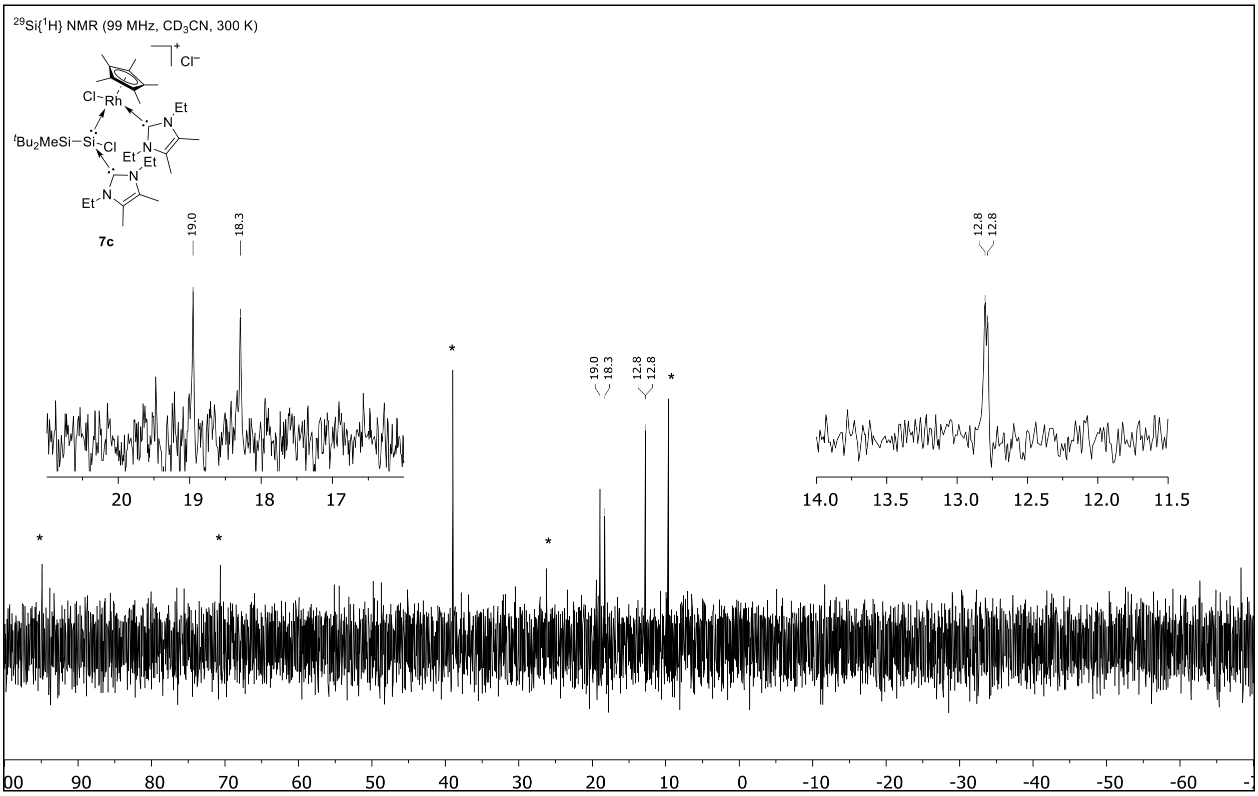

Figure S56 $^{29}Si\{^{1}H\}$ NMR spectrum of [$^{t}Bu_2MeSi$–Si(IEt$_2$Me$_2$)Cl→RhCl(IEt$_2$Me$_2$)(Cp*)]Cl (**7c**) in CD_3CN at 300 K. Unknown side products are marked with *.

1.5 General Synthetic Procedure for [R–Si(NHC)$_2$→MCl$_2$(Ar)]Cl

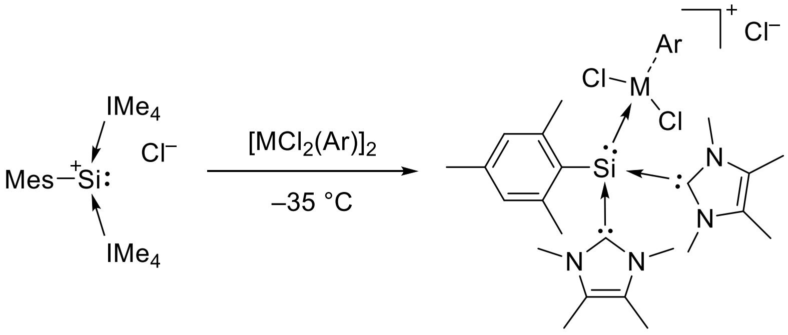

1b

3 M = Ru, Ar = *p*-cymene
4 M = Rh, Ar = Cp*

Note: *Since [Mes–Si(IMe$_4$)$_2$]Cl is not stable for a prolonged time in MeCN solution[2], care must be taken that these reactions are carried out quickly once the starting material is dissolved;* ***(b)*** *Due to the very fast decomposition of complexes* ***3*** *and* ***4*** *in solution, we were not able to isolate the complexes in a clean fashion. Decomposition even occurs slowly at –35 °C. Hence, NMR measurements were carried out immediately after mixing the starting materials.*

[Mes–Si(IMe$_4$)$_2$]Cl (**1b**) (1.0 eq) and [MCl$_2$(Ar)]$_2$ (0.5 eq) were mixed, cooled to –35 °C and pre-cooled (–35 °C) CD_3CN (0.5 mL) was added. The deep red reaction mixtures were transferred to a *J*-Young NMR tube and immediately frozen in liquid nitrogen to stop decomposition. After allowing the NMR samples to thaw the measurements were carried out immediately.

1.5.1 [Mes–Si(IMe$_4$)$_2$→RuCl$_2$(*p*-cymene)]Cl (3)

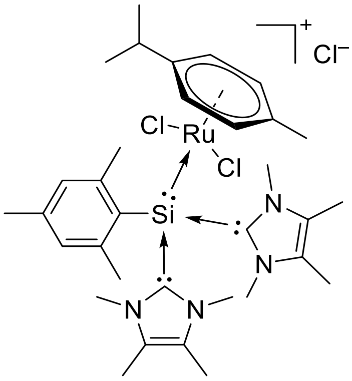

3

$C_{33}H_{49}Cl_3N_4RuSi$
737.29 g/mol

Note: *Complex **3** decomposes rapidly in solution at room temperature to an unidentified mixture of products in less than 2 hours. Decomposition is faster than in the case of **2** (~4 hours). During decomposition, large amounts of precipitate are formed that are no longer soluble in organic solvents (presumably the Si-containing part polymerizes and precipitates, as no silicon species can be observed in the NMR after complete decomposition). The major discernable products after decomposition are imidazolium chloride [IMe$_4$·HCl] and free p-cymene. The complex was characterized based on the similarity of the ^{29}Si NMR resonance to the crystallographically characterized complex **4**.*

Batch size: **1b**: 30.0 mg, 69.6 µmol, 1.0 eq.
[RuCl$_2$(*p*-cym)]$_2$: 21.3 mg, 34.8 µmol, 0.5 eq.

^{29}Si{^{1}H} NMR (99 MHz, CD$_3$CN, 300 K): δ [ppm] = –20.5 (Mes*Si*).

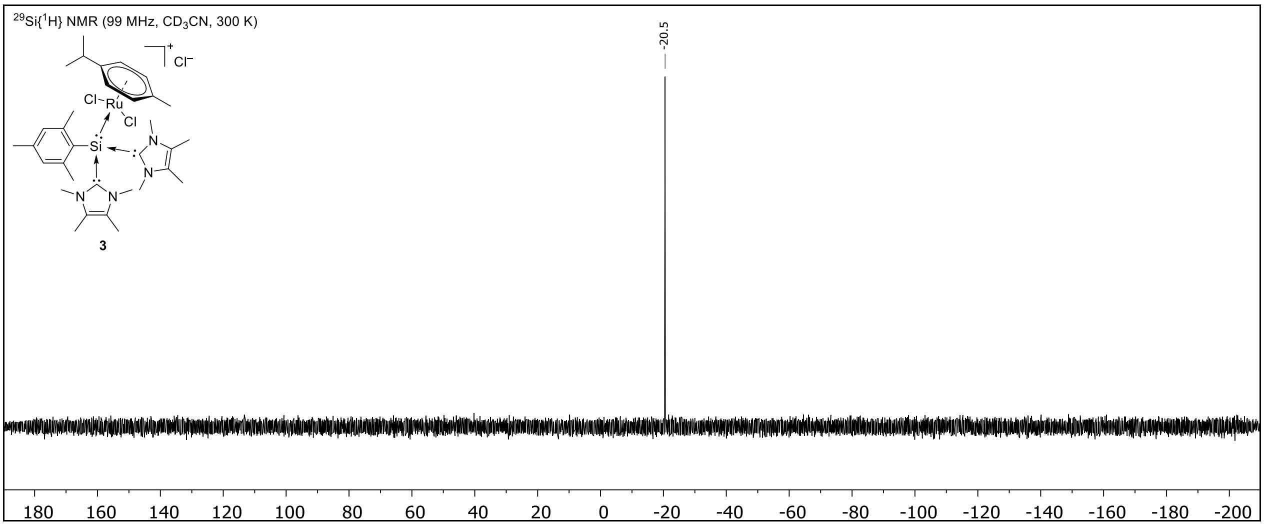

Figure S57 ^{29}Si{^{1}H} NMR spectrum of [Mes–Si(IMe$_4$)$_2$→RuCl$_2$(p-cymene)]Cl (**3**) in CD$_3$CN at 300 K.

1.5.2 [Mes–Si(IMe$_4$)$_2$→RhCl$_2$(Cp*)]X (4, X = Cl, [RhCl$_3$Cp*])

(a) [Mes–Si(IMe$_4$)$_2$→RhCl$_2$(Cp*)]Cl (4)

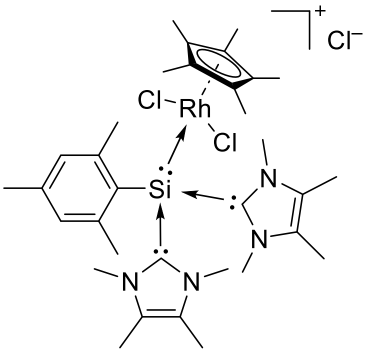

4

$C_{33}H_{50}Cl_3N_4RhSi$

740.13 g/mol

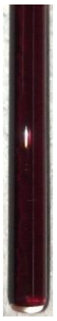

Note:* *(a) *Complex **4** decomposes rapidly in solution at room temperature to an unidentified mixture of products in less than 3 hours. Decomposition is somewhat slower than for complex **3**, presumably due to the increased stability of the Cp* ligand in acetonitrile solution;* ***(b)*** *During decomposition, large amounts of precipitate are formed that are no longer soluble in organic solvents (most likely the Si-containing part polymerizes and precipitates, as no silicon species can be observed in the NMR after complete decomposition);* ***(c)*** *Furthermore, during decomposition, the formation of a new anion [RhCl$_3$Cp*]$^-$ can be observed (Figure S58 and Figure S61) and the amount of imidazolium chloride [IMe$_4$·HCl] increases progressively during measurement. After full decomposition, the major discernable products are imidazolium chloride and several Cp* containing species; (**d**) Due to the decomposition of **4** occurring even at low temperatures, it was not possible to obtain single crystals of **4**. However, single crystals of **4-RhCl$_3$Cp*** could be obtained from the same reaction mixture. **4-RhCl$_3$Cp*** can also be selectively synthesized by using one equivalent of [RhCl$_2$(Cp*)]$_2$ instead of 0.5 eq (vide supra). **4-RhCl$_3$Cp*** does not appear to be more stable than **4**.*

Batch size: **1b**: 50.0 mg, 116.0 µmol, 1.0 eq.
[RhCl$_2$(Cp*)]$_2$: 35.8 mg, 58 µmol, 0.5 eq.

^{1}H NMR (500 MHz, CD$_3$CN, 300 K): δ [ppm] = 6.99 (s, 1H, C$_{mes}$*H*), 6.94 (s, 1H, C$_{mes}$*H*), 4.23 (s, 3H, N$_{NHC\rightarrow Si}$C*H*$_3$), 3.50 (s, 3H, N$_{NHC\rightarrow Rh}$C*H*$_3$), 3.35 (s, 3H, N$_{NHC\rightarrow Rh}$C*H*$_3$), 2.80 (s, 3H, N$_{NHC\rightarrow Si}$C*H*$_3$), 2.47 (s, 3H, C$_{NHC\rightarrow Si}$C*H*$_3$), 2.29 (s, 3H, C$_{mes,para}$C*H*$_3$), 2.21 (s, 3H, C$_{NHC\rightarrow Rh}$C*H*$_3$), 2.15 (bs, 6H, C$_{mes,ortho}$C*H*$_3$), 2.10 (s, 3H, C$_{NHC\rightarrow Rh}$C*H*$_3$), 1.69 (s, 3H, C$_{NHC\rightarrow Si}$C*H*$_3$), 1.18 (s, 15H, C$_5$(C*H*$_3$)$_5$).

Note: *The resonance of the ortho methyl (2.15 ppm) groups of the silyliumylidene ion ligand overlap with a resonance for imidazolium chloride (2.17 ppm)*

^{29}Si{^{1}H} NMR (99 MHz, CD$_3$CN, 300 K): δ [ppm] = –24.2 (d, $^1J_{Si\text{–}Rh}$ = 66.9 Hz, Mes*Si*).

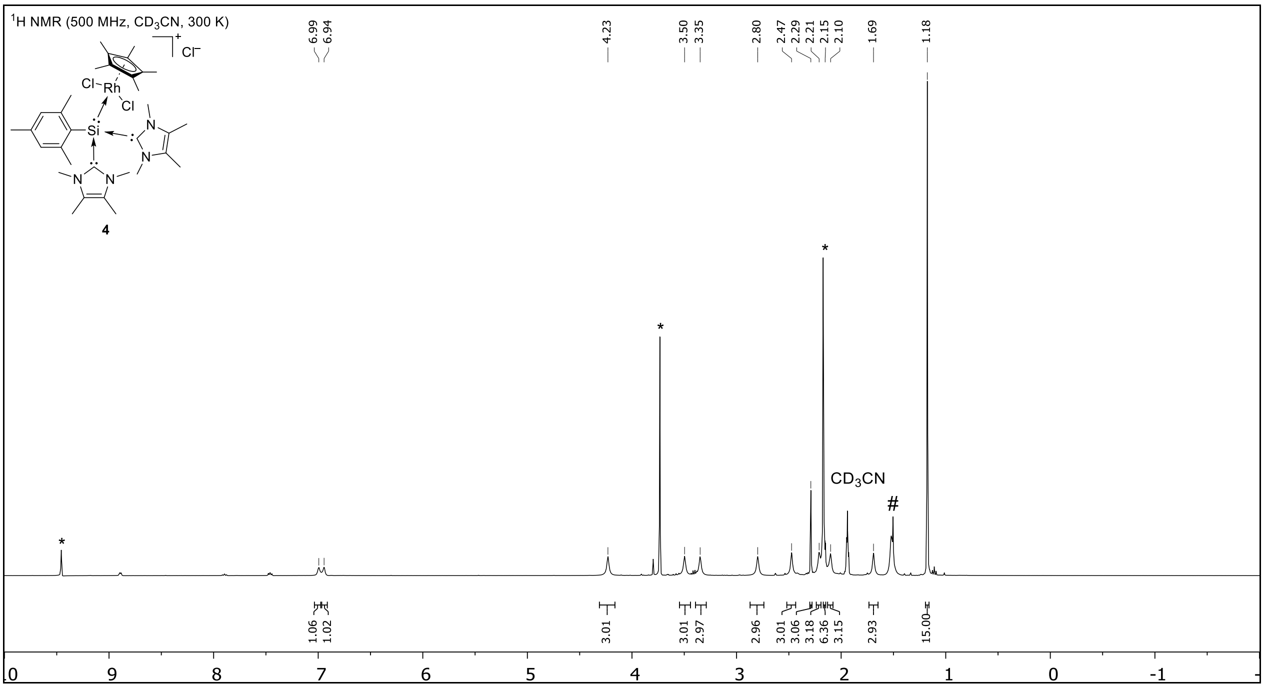

Figure S58 ^{1}H NMR spectrum of [Mes–Si(IMe$_4$)$_2$→RhCl$_2$(Cp*)]Cl (**4**) in CD_3CN at 300 K. The resonances for imidazolium chloride [IMe$_4$·HCl] (marked with *) increase significantly during decomposition and therefore also increase during measurement. The resonance corresponding to the [RhCl$_3$Cp*]$^-$ anion also increases during measurement (marked with #).

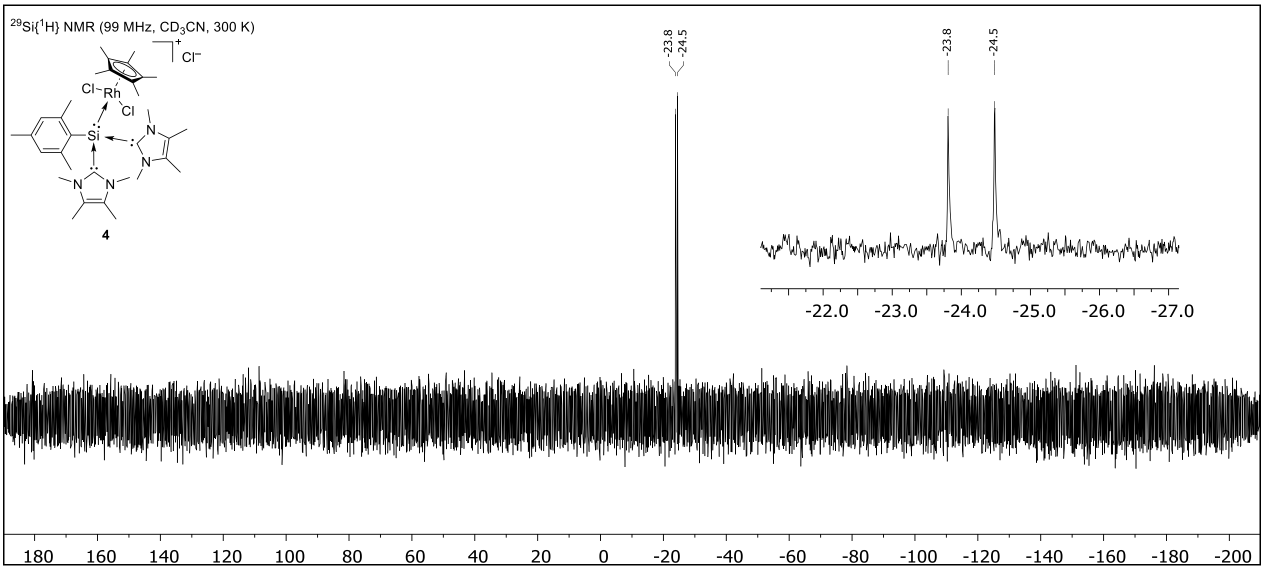

Figure S59 ^{29}Si{^{1}H} NMR spectrum of [Mes–Si(IMe$_4$)$_2$→RhCl$_2$(Cp*)]Cl (**4**) in CD_3CN at 300 K. No silicon-containing decomposition products can be observed, even after full decomposition.

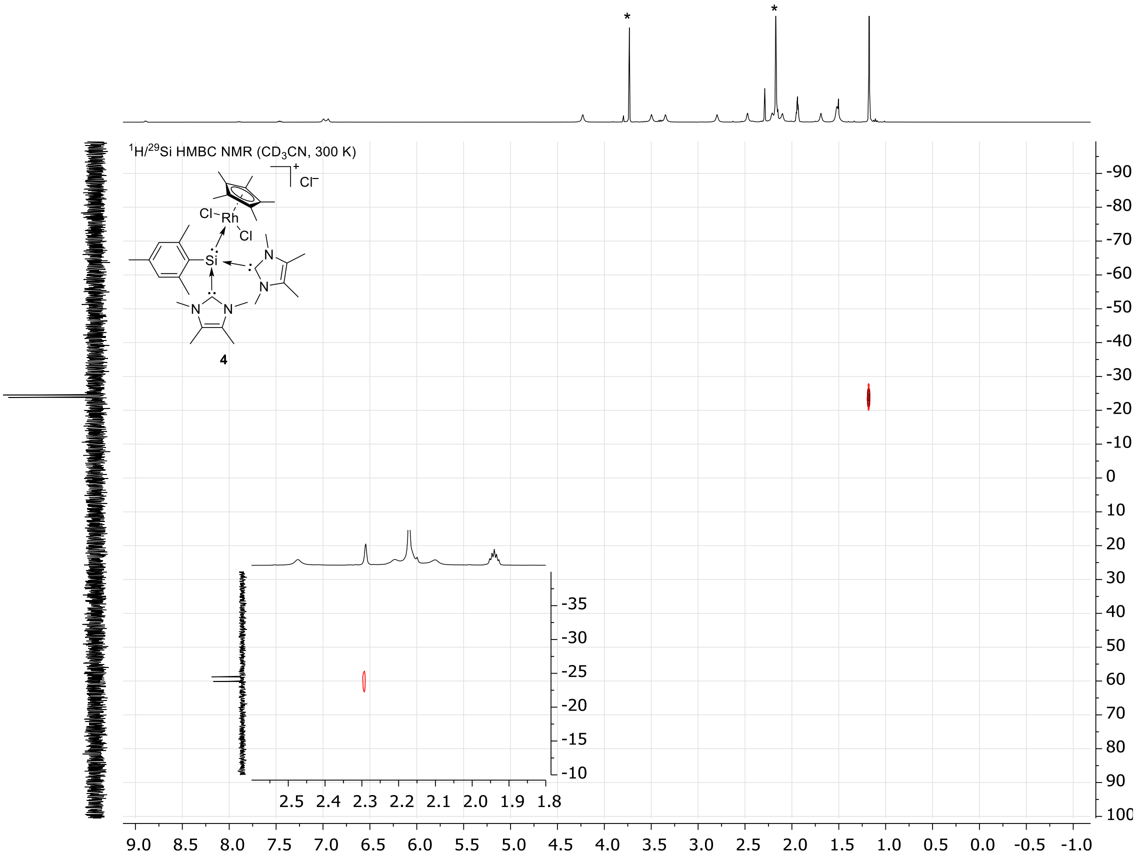

Figure S60 $^{1}H/^{29}Si$ HMBC NMR spectrum of [Mes–Si(IMe$_4$)$_2$→RhCl$_2$(Cp*)]Cl (**4**) in CD$_3$CN at 300 K. Imidazolium chloride [IMe$_4$·HCl] is marked with *.

(b) $[Mes–Si(IMe_4)_2{\rightarrow}RhCl_2(Cp^*)][RhCl_3(Cp^*)]$ (4-RhCl$_3$Cp*)

Note: ***(a)*** ***4-RhCl$_3$Cp**** *was analyzed with the same procedure as described for* ***4*** *(cf. section 1.5) except 1.0 eq of $[RhCl_2(Cp^*)]_2$ were used instead of 0.5 eq.;* ***(b)*** *The cationic part of the complex remains the same regardless of the stoichiometry of the reaction; only the anionic part changes from Cl^- to $[RhCl_3Cp^*]^-$ if an equimolar (or excess) amount of transition metal precursor is utilized. The new anion is also formed during decomposition of* ***4****, albeit not quantitatively;* ***(c)*** *^{29}Si NMR data of* ***4*** *(Figure S59) and* ***4-RhCl$_3$Cp**** *(Figure S62) is identical. ^{1}H NMR data (Figure S58 & Figure S61) for the cationic parts are the same. The ^{1}H NMR spectrum of* ***4-RhCl$_3$Cp**** *shows an additional resonance for the second Cp* ring (1.56 ppm ($C_5(CH_3)_5$).*

^{1}H NMR (500 MHz, CD$_3$CN, 300 K): δ [ppm] = 7.00 (s, 1H, $C_{mes}H$), 6.94 (s, 1H, $C_{mes}H$), 4.23 (s, 3H, $N_{NHC\rightarrow Si}CH_3$), 3.50 (s, 3H, $N_{NHC\rightarrow Rh}CH_3$), 3.34 (s, 3H, $N_{NHC\rightarrow Rh}CH_3$), 2.78 (s, 3H, $N_{NHC\rightarrow Si}CH_3$), 2.48 (s, 3H, $C_{NHC\rightarrow Si}CH_3$), 2.29 (s, 3H, $C_{mes,para}CH_3$), 2.21 (s, 3H, $C_{NHC\rightarrow Rh}CH_3$), 2.16 (bs, 6H, $C_{mes,ortho}CH_3$), 2.10 (s, 3H, $C_{NHC\rightarrow Rh}CH_3$), 1.69 (s, 3H, $C_{NHC\rightarrow Si}CH_3$), 1.56 (s, 15H, $[RhCl_3(C_5(CH_3)_5)]$), 1.18 (s, 15H, $C_5(CH_3)_5$).

^{29}Si{^{1}H} NMR (99 MHz, CD$_3$CN, 300 K): δ [ppm] = –24.1 (d, $^{1}J_{Si–Rh}$ = 67.0 Hz, Mes*Si*).

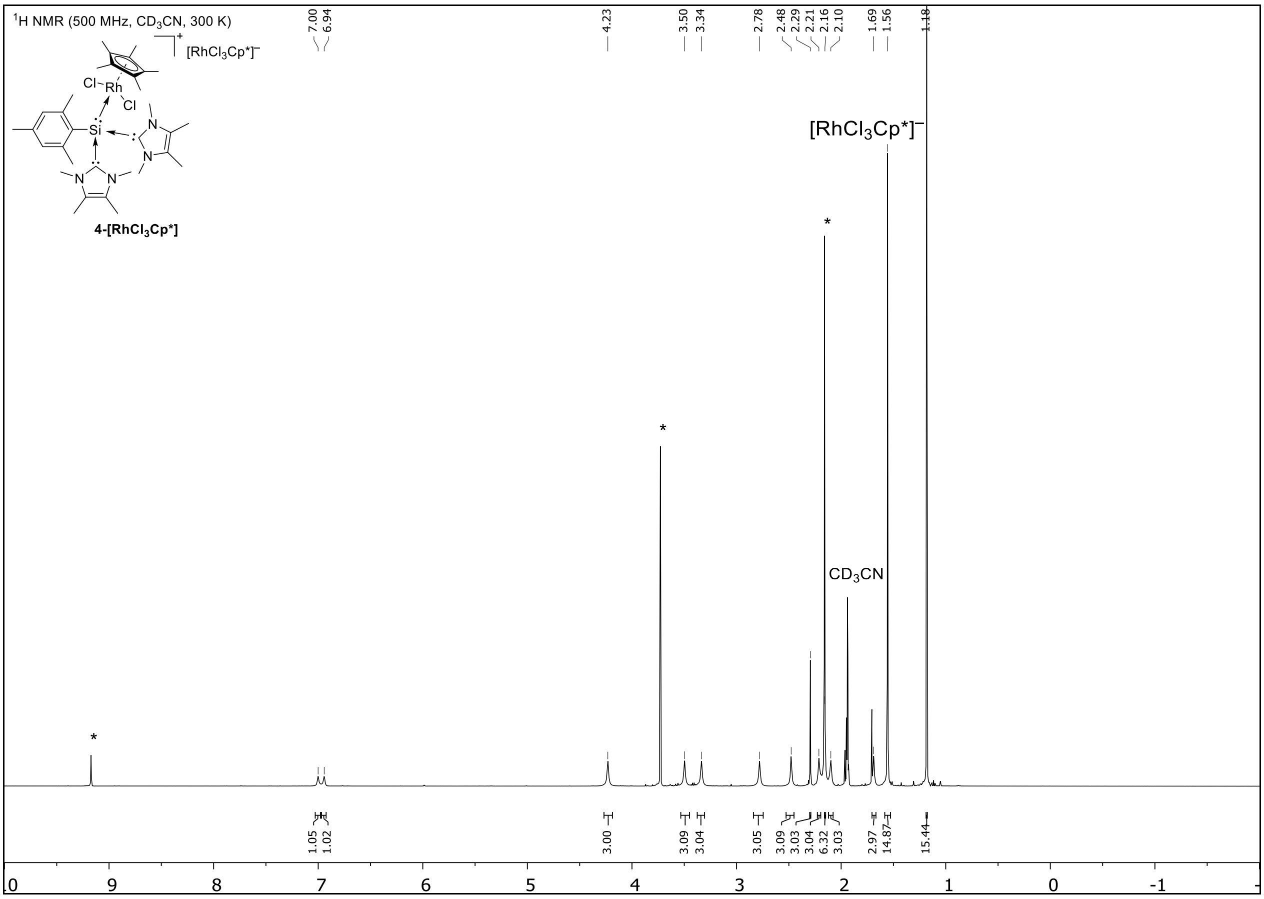

Figure S61 ^{1}H NMR spectrum of $[Mes–Si(IMe_4)_2{\rightarrow}RhCl_2(Cp^*)][RhCl_3(Cp^*)]$ (**4-RhCl$_3$Cp***) in CD_3CN at 300 K. The resonances for imidazolium chloride [IMe_4·HCl] (marked with *) increase significantly during decomposition and therefore also increase during measurement. No increase of the signal assigned to $[RhCl_3Cp^*]^-$ can be observed.

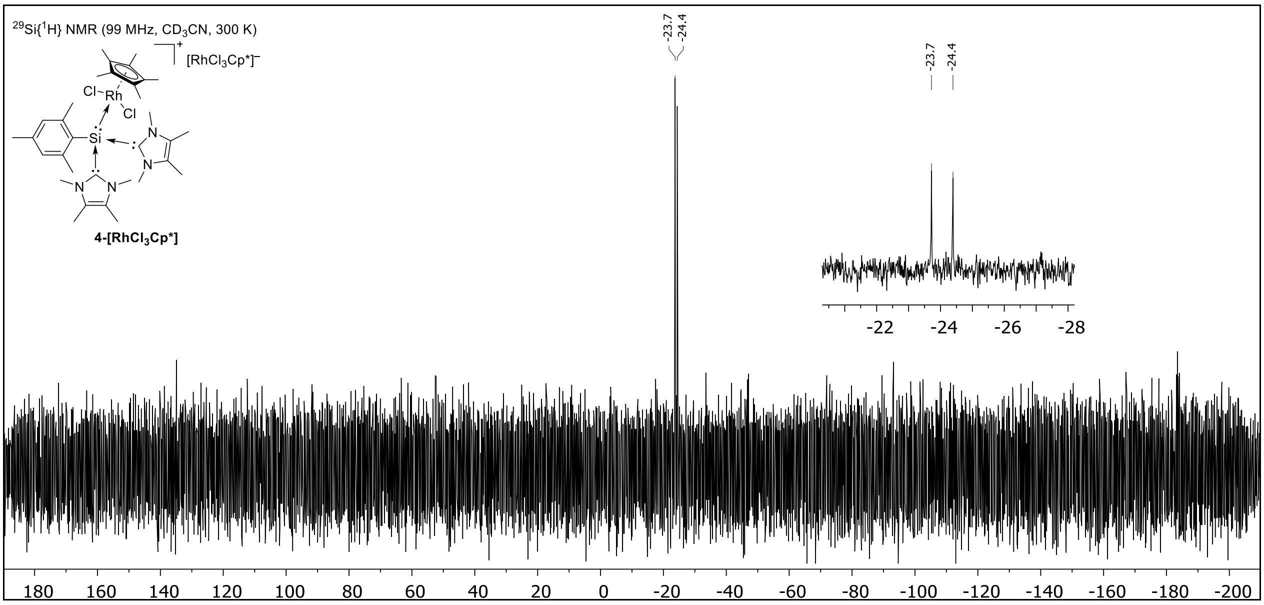

Figure S62 $^{29}Si\{^1H\}$ NMR spectrum of [Mes–Si(IMe$_4$)$_2$→RhCl$_2$(Cp*)][RhCl$_3$(Cp*)] (**4-RhCl$_3$Cp***) in CD_3CN at 300 K.

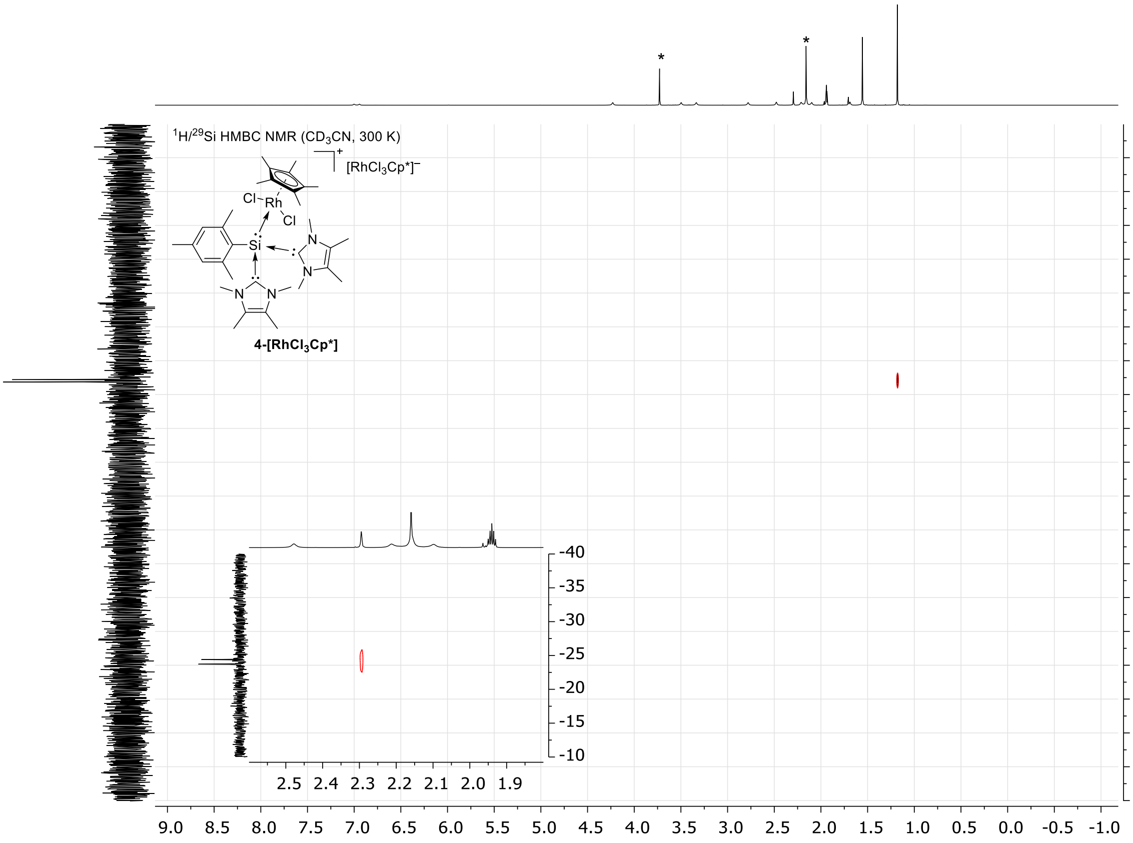

Figure S63 $^1H/^{29}Si$ HMBC NMR spectrum of [Mes–Si(IMe$_4$)$_2$→RhCl$_2$(Cp*)][RhCl$_3$(Cp*)] (**4-RhCl$_3$Cp***) in CD_3CN at 300 K.

1.6 General Synthetic Procedure for [^{t}Bu$_3$Si–SiCl$_2$–M(IMe$_4$)(Ar)]

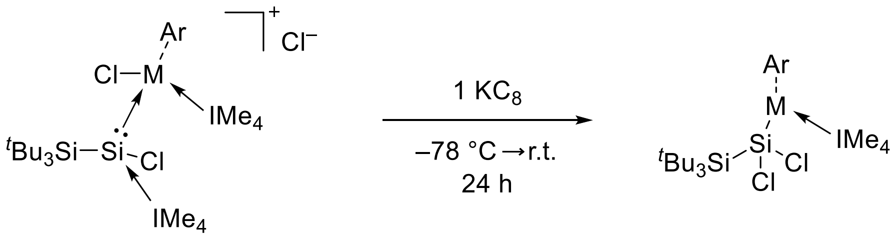

6a M = Ru, Ar = *p*-cymene
7a M = Rh, Ar = Cp*

8 M = Ru, Ar = *p*-cymene
9 M = Rh, Ar = Cp*

[^{t}Bu$_3$Si–Si(IMe$_4$)Cl→MCl(IMe$_4$)(Ar)]Cl (**6a** (M = Ru, Ar = *p*-cymene), **7a** (M = Rh, Ar = Cp*)) (1.0 eq) and KC$_8$ (1.0 eq) were mixed, cooled to –78 °C and pre-cooled (–78 °C) THF (20 mL) was added rapidly while stirring. The mixtures were stirred at –78 °C for 4 hours, then slowly warmed to room temperature overnight (~16 hours) and then stirred at room temperature for an additional 4 hours. The solvent was removed under reduced pressure and the residue was extracted with toluene (3×7 mL). The solvent was removed under reduced pressure and the residue was dried for 5 hours (60 °C, ~5×10^{-3} mbar) to remove free IMe$_4$ *via* sublimation. The residues were dissolved in a minimal amount of toluene and after crystallization at –35 °C, filtration and drying under vacuum, complexes **8** and **9** were obtained as air- and moisture sensitive solids.

Note: *Complexes **8** and **9** are stable in solution at room temperature for at least four weeks.*

1.6.1 [^{t}Bu$_3$Si–SiCl$_2$–Ru(IMe$_4$)(*p*-cymene)] (8)

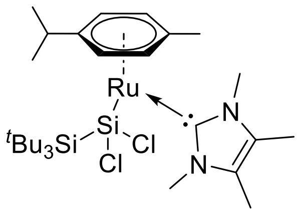

8

$C_{29}H_{53}Cl_2N_2RuSi_2$

657.90 g/mol

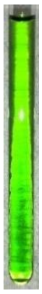

Batch size: **6a**: 100.0 mg, 122.3 µmol, 1.0 eq.
KC_8: 16.5 mg, 122.3 µmol, 1.0 eq.

Yield: 45.5 mg (69.2 µmol, 57%) as a bright green solid.

SC-XRD: Suitable single crystals were obtained by storing a concentrated solution of **8** in toluene at –35 °C.

Note: *No NMR data could be recorded due to* ***8*** *being paramagnetic.*

EPR (toluene, c = 5×10^{-5} ^{mol}/$_L$, 286 K): *g* = 2.1062.

LIFDI-MS: calculated: 657.2168 ($C_{29}H_{53}Cl_2N_2RuSi_2$).
measured: 657.1815 (**8**).

EA: $C_{29}H_{53}Cl_2N_2RuSi_2$ calculated [%]: C (52.94), H (8.12), N (4.26).
measured [%]: C (52.45), H (8.12), N (4.05).

M.P.: 169–170 °C (decomposition, color change to black).

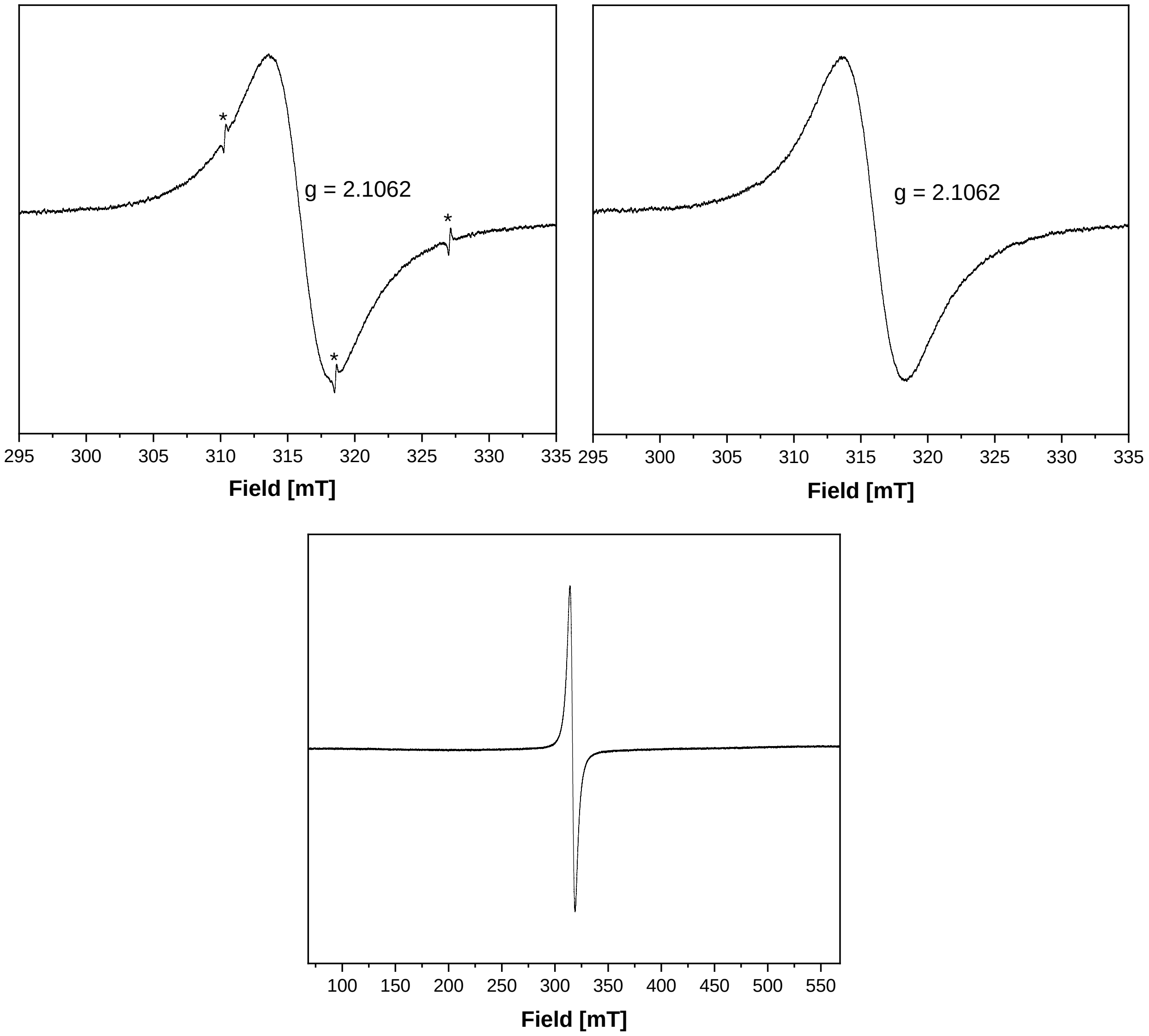

Figure S64 X-band EPR-spectra of [^{t}Bu$_3$Si–SiCl$_2$–Ru(IMe$_4$)(*p*-cymene)] (**8**) in toluene (c = 5.0×10^{-5} $^{mol}/_{L}$, 286 K) (top left: including standard (marked with *), top right: excluding standard, bottom: from 70 to 570 mT).

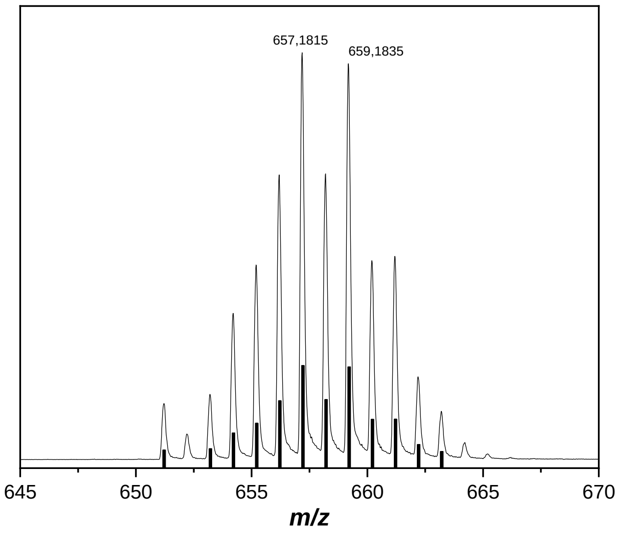

Figure S65 LIFDI-MS spectrum (detailed view) of [^{t}Bu$_3$Si–SiCl$_2$–Ru(IMe$_4$)(*p*-cymene)] (**8**) in toluene (line: measured spectrum; bars: simulated spectrum).

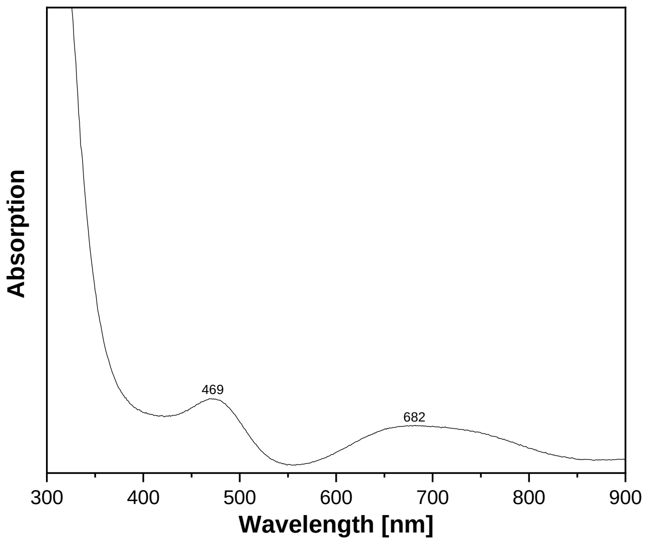

Figure S66 UV-Vis spectrum (300-900 nm) of [$^{t}Bu_3Si–SiCl_2–Ru(IMe_4)$(*p*-cymene)] (**8**) in toluene at room temperature (c = 5.0×10^{-4} $^{mol}/_{L}$). λ_{max} = 469 nm & 682 nm.

1.6.2 [^{t}Bu$_3$Si–SiCl$_2$–Rh(IMe$_4$)(Cp*)] (9)

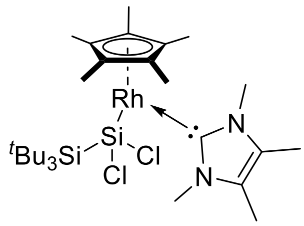

9

$C_{29}H_{54}Cl_2N_2RhSi_2$

660.74 g/mol

Batch size: **7a**: 100.0 mg, 121.9 µmol, 1.0 eq.

KC_8: 16.5 mg, 121.9 µmol, 1.0 eq.

Yield: 52.3 mg (79.2 µmol, 65%) as a dark grey-black solid.

SC-XRD: Suitable single crystals were obtained by storing a concentrated solution of **9** in toluene at –35 °C.

Note: *No NMR data could be recorded due to* ***9*** *being paramagnetic.*

EPR (toluene, c = 5×10^{-5} $^{mol}/_{L}$, 286 K): *g* = 2.1003.

LIFDI-MS: calculated: 659.2258 ($C_{29}H_{54}Cl_2N_2RhSi_2$).

measured: 659.1942 (**9**).

EA: $C_{29}H_{54}Cl_2N_2RhSi_2 \cdot 0.5(C_7H_8)$ calculated [%]: C (55.23), H (8.27), N (3.96).

measured [%]: C (55.29), H (8.39), N (3.75).

M.P.: 191-192 °C (decomposition, change to black oil).

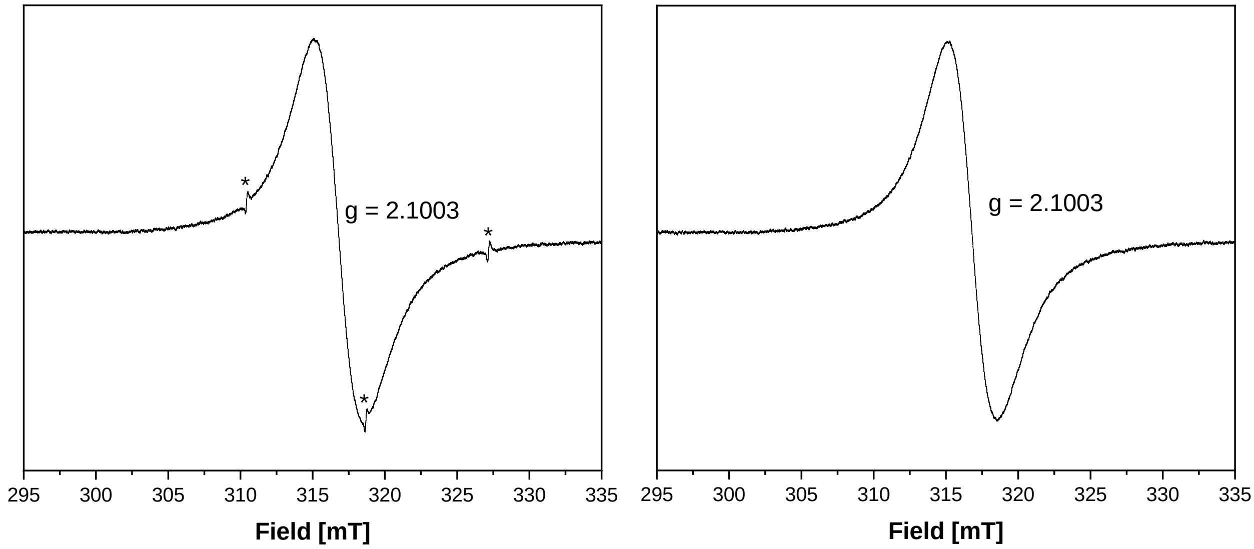

Figure S67 X-band EPR-spectra of [^{t}Bu$_3$Si–SiCl$_2$–Rh(Cp*)(IMe$_4$)] (**9**) in toluene (c = 5.0×10^{-5} $^{mol}/_{L}$, 286 K) (left: including standard (marked with *), right: excluding standard).

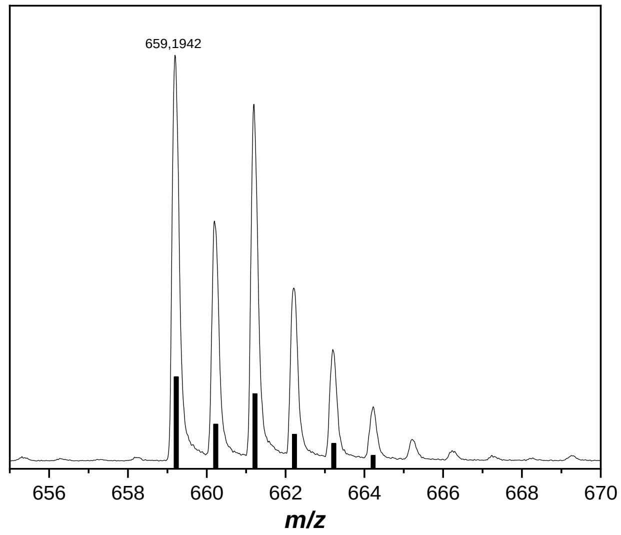

Figure S68 LIFDI-MS spectrum (detailed view) of [${}^{t}Bu_3Si–SiCl_2–Rh(Cp^*)(IMe_4)$] (**9**) in toluene; (line: measured spectrum; bars: simulated spectrum).

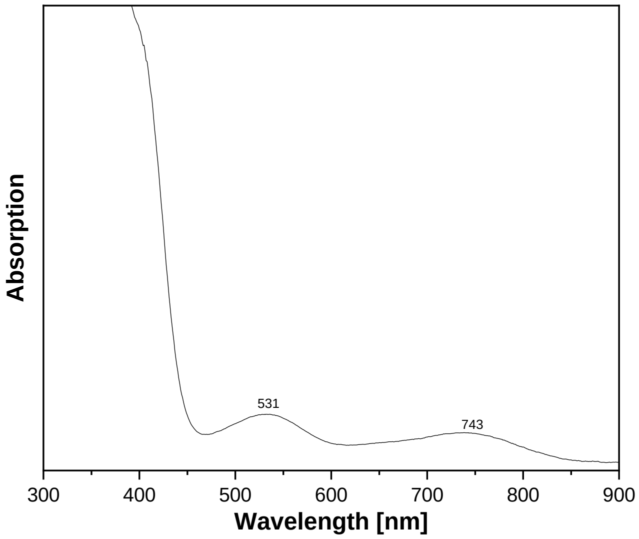

Figure S69 UV-Vis spectrum (300-900 nm) of [${}^{t}Bu_3Si–SiCl_2–Rh(Cp^*)(IMe_4)$] (**9**) in toluene at room temperature (c = $1.5×10^{-3}$ $^{mol}/_{L}$). $λ_{max}$ = 531 nm & 743 nm.

1.7 Synthesis of [^{t}Bu$_3$Si–Si(Cl)=Ru(IMe$_4$)(*p*-cymene)] (10)

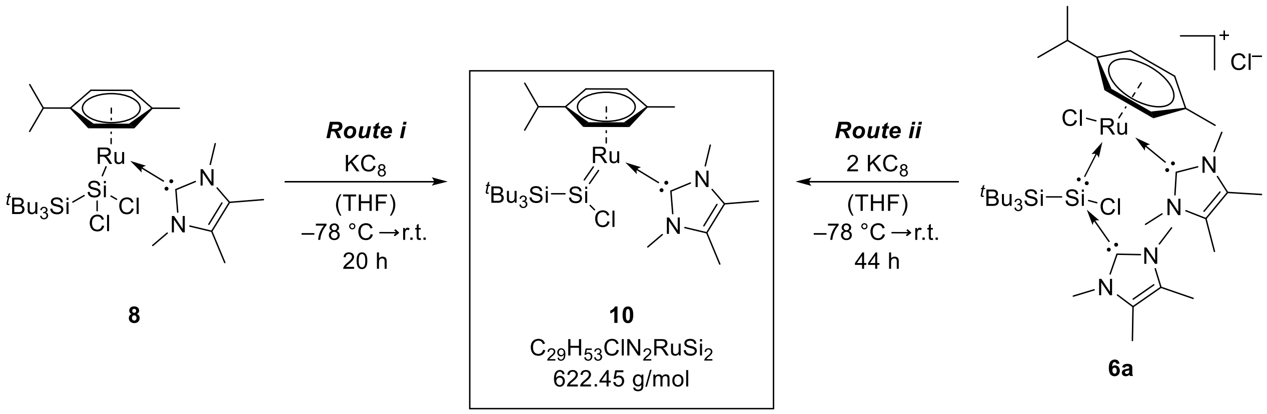

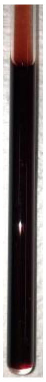

Complex **10** can be synthesized either from **8** through one electron reduction and abstraction of one chloride (***Route i***) or through two-electron reduction and abstraction of two chlorides directly from **6a** (***Route ii***). Isolation of **8** prior to further reduction gives a cleaner reaction and higher yields.

Route i: [^{t}Bu$_3$Si–SiCl$_2$–Ru(IMe$_4$)(*p*-cymene)] (**8**) (50.0 mg, 76.0 µmol, 1.0 eq) and KC_8 (10.3 mg, 76.0 µmol, 1.0 eq) were mixed, cooled to –78 °C and pre-cooled (–78 °C) THF (10 mL) was added rapidly while stirring. The mixture was slowly warmed to room temperature over a period of 4 hours (color change from green→brown→red) and then stirred overnight at room temperature (~16 hours). The solvent was removed under reduced pressure and the residue was extracted with hexane (3×5 mL). The solution was concentrated under reduced pressure and then stored at –35 °C for 1 week. The microcrystalline precipitate was collected by filtration, washed with cold hexane (–35 °C, 1×0.5 mL) and after drying under vacuum **10** (29.9 mg, 48.0 µmol, 63%) was obtained as a dark red air- and moisture sensitive solid.

Route ii: [^{t}Bu$_3$Si–Si(IMe$_4$)Cl→RuCl(IMe$_4$)(*p*-cymene)]Cl (**6a**) (100.0 mg, 122.3 µmol, 1.0 eq) and KC_8 (33.1 mg, 244.6 µmol, 2.0 eq) were mixed, cooled to –78 °C and pre-cooled (–78 °C) THF (20 mL) was added rapidly while stirring. The mixture was stirred at –78 °C for 4 hours, then slowly warmed to room temperature overnight (~16 hours) and then stirred at room temperature for an additional 24 hours. The solvent was removed under reduced pressure and the residue was extracted with hexane (3×8 mL). The solution was concentrated under reduced pressure and then stored at –35 °C for 10 days. The microcrystalline precipitate was collected by filtration, washed with cold hexane (–35 °C, 1×0.5 mL) and after drying under vacuum **10** (31.4 mg, 50.5 µmol, 41%) was obtained as a dark red air- and moisture sensitive solid.

SC-XRD: Suitable single crystals were obtained by storing a concentrated solution of **10** in *n*-hexane at –35 °C.

^{1}H NMR (500 MHz, C_6D_6, 300 K): δ [ppm] = 4.78 (m, 4H, $C_{p\text{-cym}}H_{ar}$), 3.42 (s, 6H, $N_{NHC}CH_3$), 2.44 (sept, $^3J_{H–H}$ = 6.9 Hz, 1H, $CH(CH_3)_2$), 2.26 (s, 3H, $C_{p\text{-cym}}CH_3$), 1.67 (s, 6H, $C_{NHC}CH_3$), 1.56 (s, 27H, $Si((C(CH_3)_3)_3$), 1.39 (d, $^3J_{H–H}$ = 6.9 Hz, 6H, $CH(CH_3)_2$).

$^{13}C\{^1H\}$ NMR (126 MHz, C_6D_6, 300 K): δ [ppm] = 188.5 ($NC_{NHC}N$), 123.8 ($C_{NHC}CH_3$), 108.5 ($C_{p\text{-cym}}$–^{i}Pr), 94.0 ($C_{p\text{-cym}}CH_3$), 81.7 ($C_{p\text{-cym}}H_{ar}$), 78.7 ($C_{p\text{-cym}}H_{ar}$), 36.6 ($N_{NHC}CH_3$), 32.8 ($CH(CH_3)_2$), 32.6 ($Si((C(CH_3)_3)_3$), 25.5 ($CH(CH_3)_2$), 24.9 ($Si((C(CH_3)_3)_3$), 21.8 ($C_{p\text{-cym}}CH_3$), 9.4 ($C_{NHC}CH_3$).

$^{29}Si\{^1H\}$ NMR (99 MHz, C_6D_6, 300 K): δ [ppm] = 240.6 (*Si*=Ru), 4.7 (*Si*^{t}Bu$_3$).

LIFDI-MS: calculated: 622.2479 ($C_{29}H_{53}ClN_2RuSi_2$).
measured: 622.2941 (**10**).

EA: $C_{29}H_{53}ClN_2RuSi_2$ calculated [%]: C (55.96), H (8.58), N (4.50).
measured [%]: C (56.23), H (8.84), N (4.59).

M.P.: 149-150 °C (decomposition, color change to black).

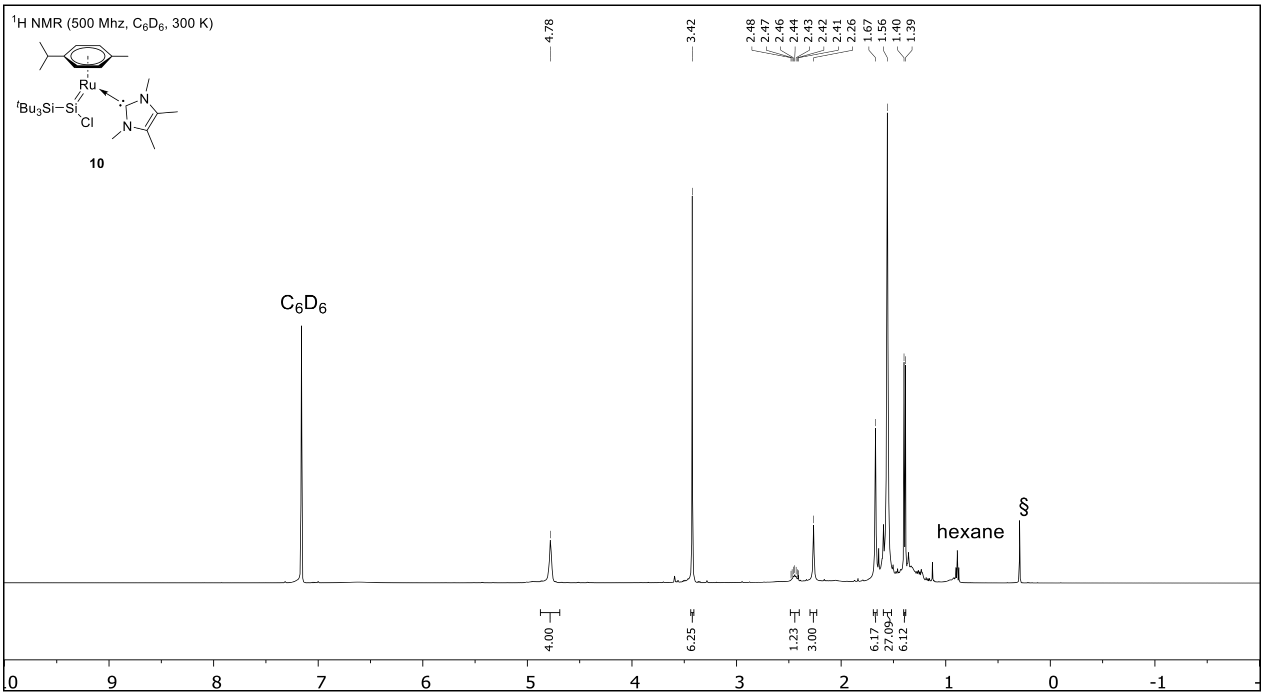

Figure S70 ^{1}H NMR spectrum of [^{t}Bu$_3$Si–Si(Cl)=Ru(IMe$_4$)(*p*-cymene)] (**10**) in C_6D_6 at 300 K. Silicone grease is marked with §.

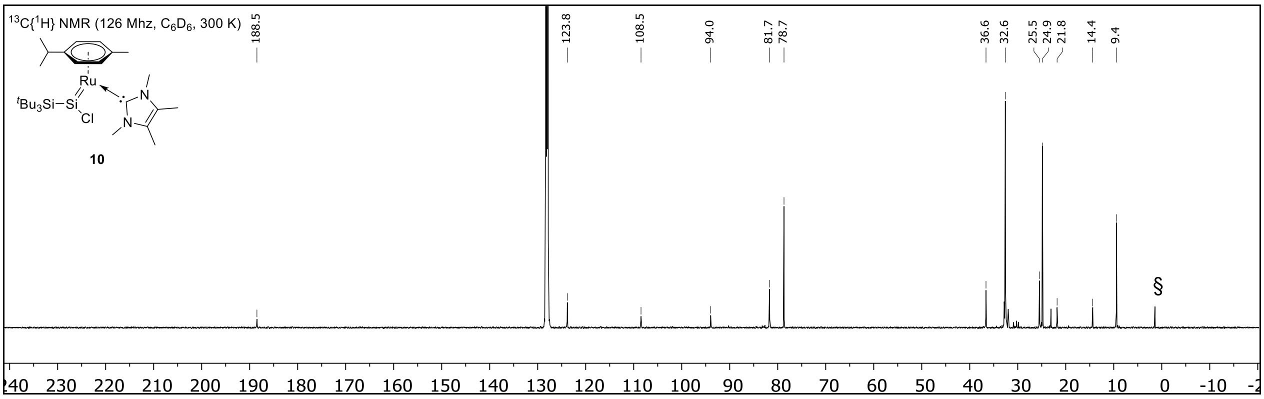

Figure S71 $^{13}C\{^{1}H\}$ NMR spectrum of [^{t}Bu$_3$Si–Si(Cl)=Ru(IMe$_4$)(*p*-cymene)] (**10**) in C_6D_6 at 300 K. Silicone grease is marked with §.

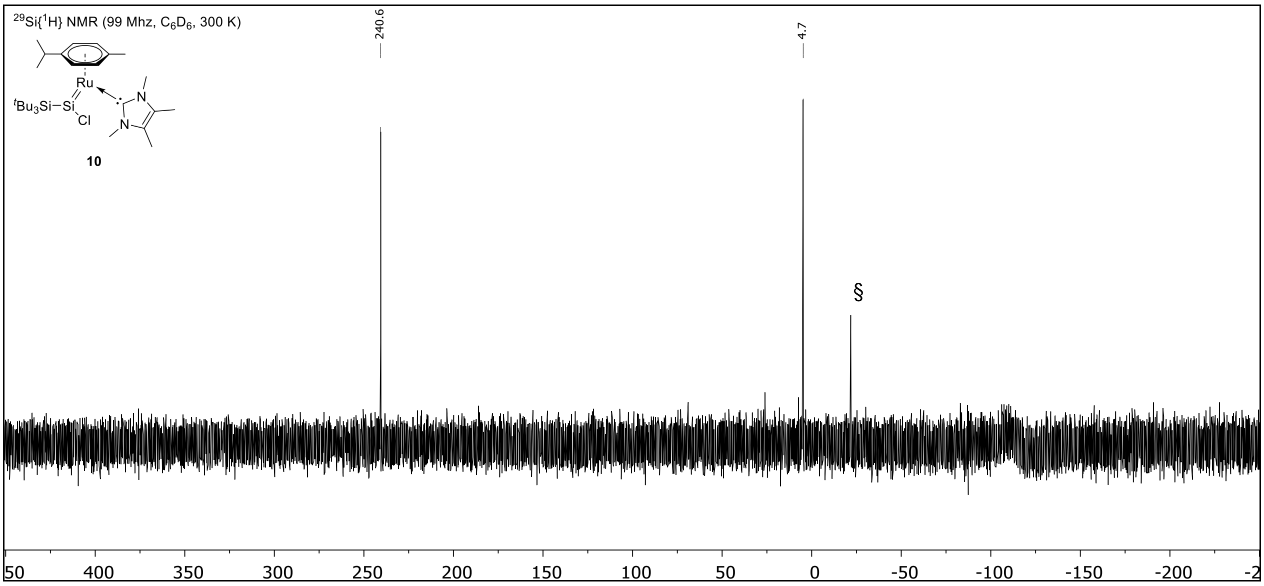

Figure S72 $^{29}Si\{^{1}H\}$ NMR spectrum of [^{t}Bu$_3$Si–Si(Cl)=Ru(IMe$_4$)(*p*-cymene)] (**10**) in C_6D_6 at 300 K. Silicone grease is marked with §.

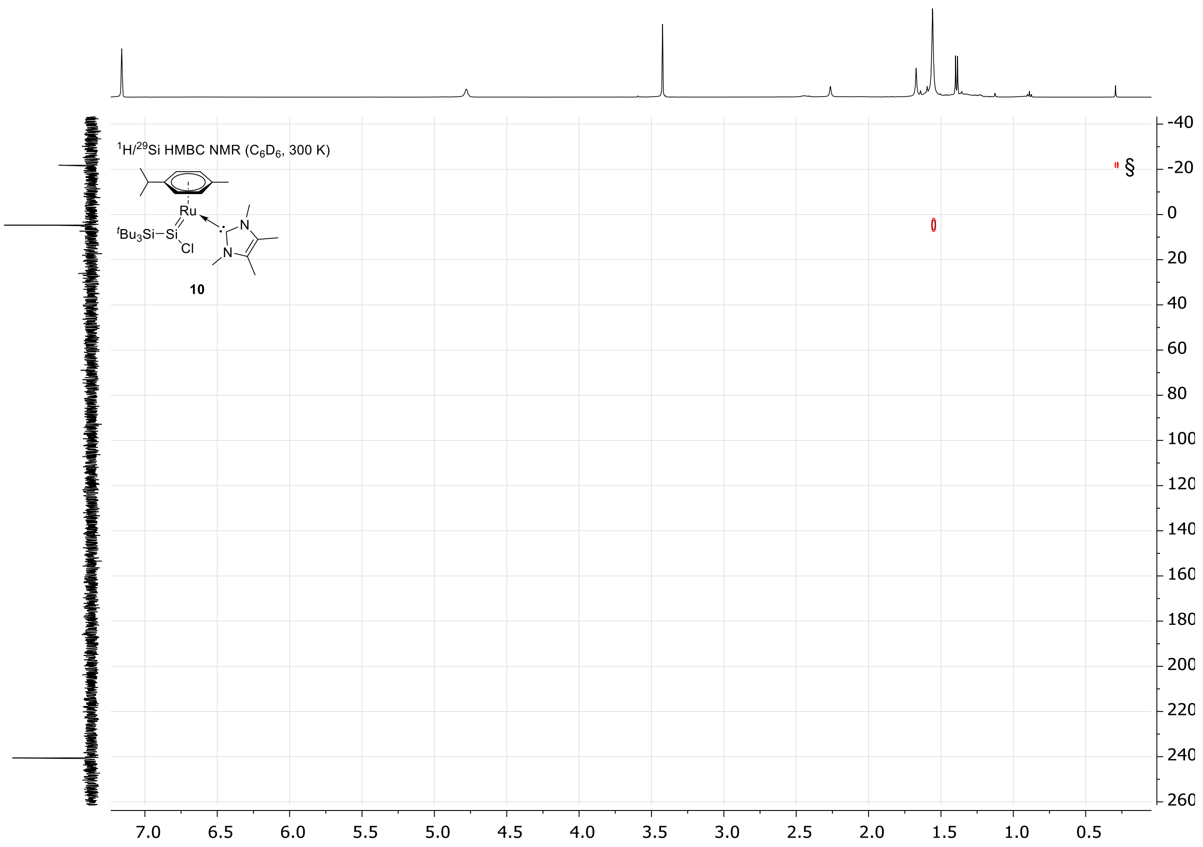

Figure S73 $^1H/^{29}Si$ HMBC NMR spectrum of [$^tBu_3Si$–Si(Cl)=Ru(IMe$_4$)(*p*-cymene)] (**10**) in C_6D_6 at 300 K. Silicone grease is marked with §.

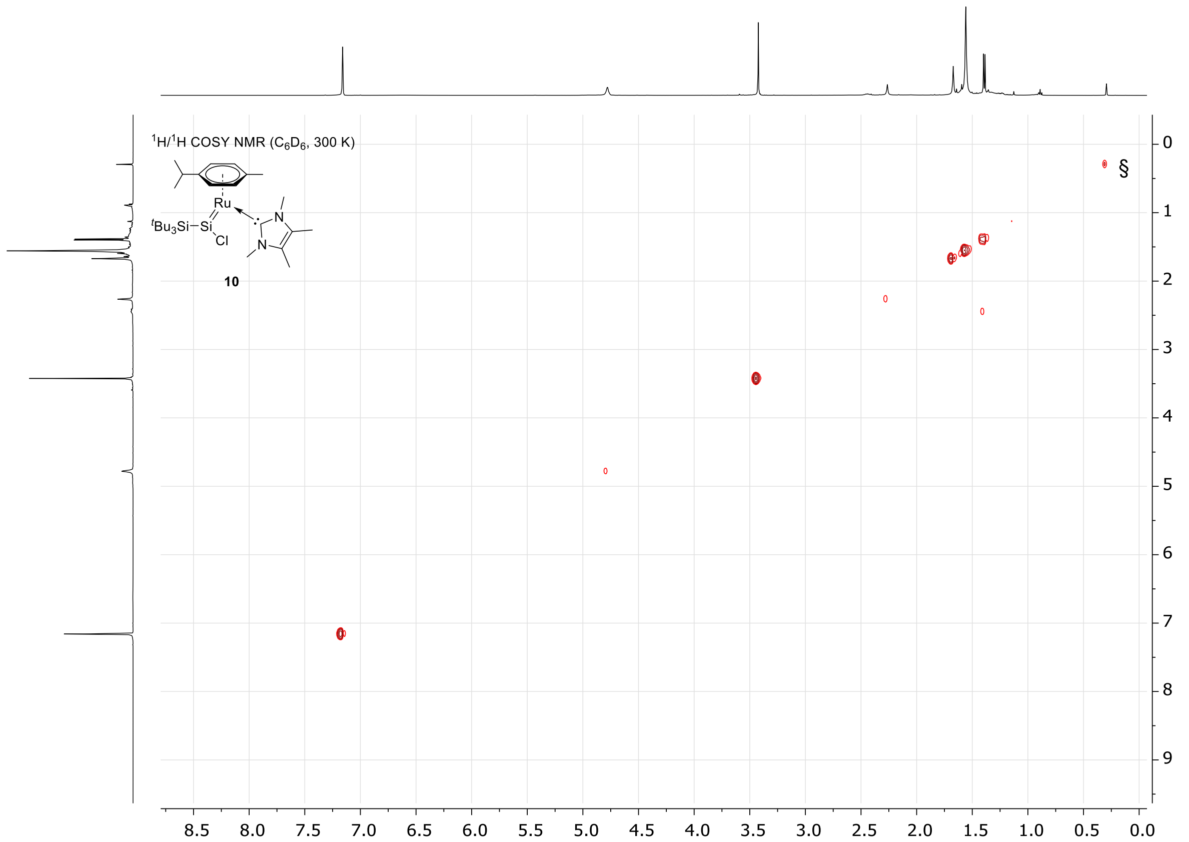

Figure S74 $^1H/^1H$ COSY NMR spectrum of [$^tBu_3Si$–Si(Cl)=Ru(IMe$_4$)(*p*-cymene)] (**10**) in C_6D_6 at 300 K. Silicone grease is marked with §.

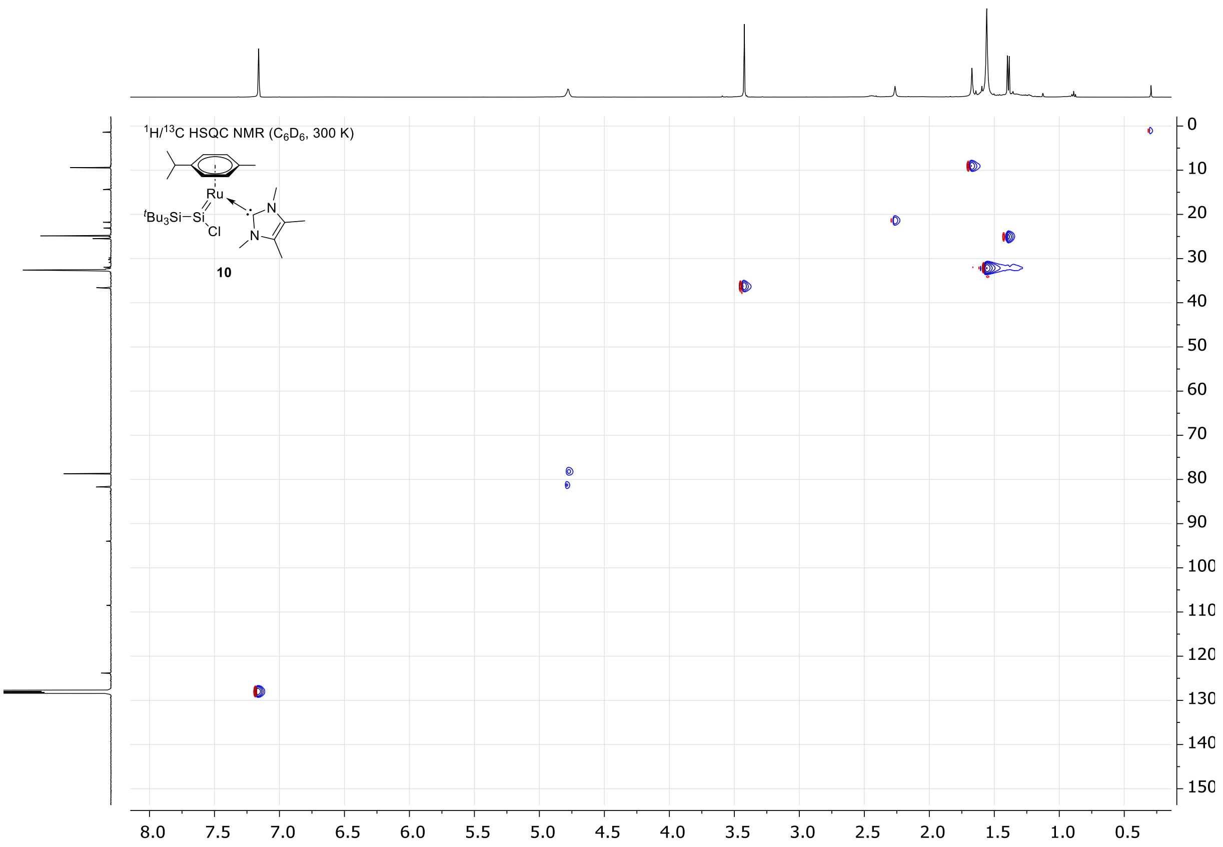

Figure S75 $^{1}H/^{13}C$ HSQC NMR spectrum of [$^{t}Bu_3Si$–Si(Cl)=Ru(IMe$_4$)(*p*-cymene)] (**10**) in C_6D_6 at 300 K.

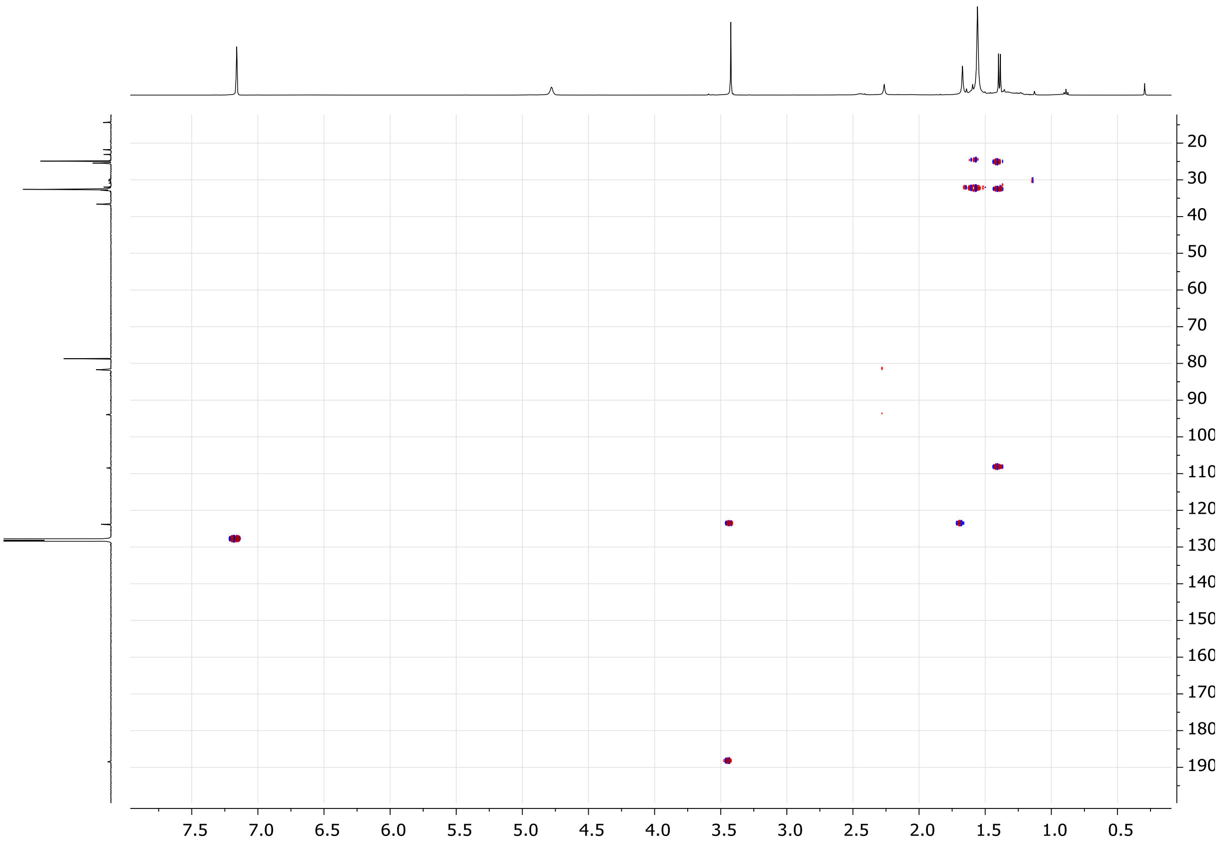

Figure S76 $^{1}H/^{13}C$ HMBC NMR spectrum of [$^{t}Bu_3Si$–Si(Cl)=Ru(IMe$_4$)(*p*-cymene)] (**10**) in C_6D_6 at 300 K.

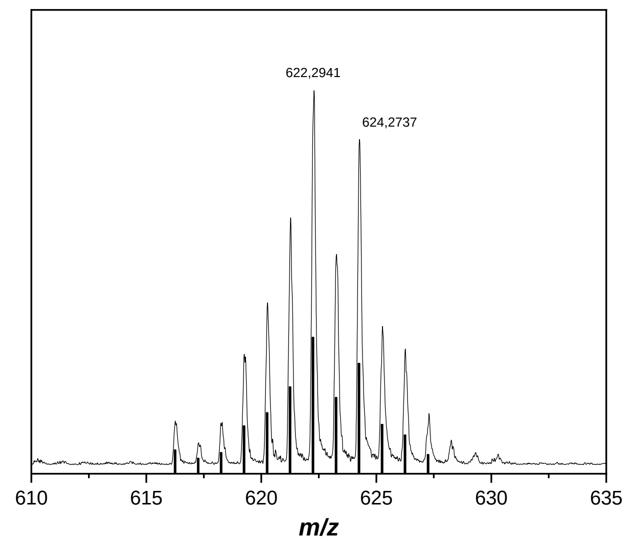

Figure S77 LIFDI-MS spectrum (detailed view) of [$^{t}Bu_3Si–Si(Cl)=Ru(IMe_4)$(*p*-cymene)] (**10**) in toluene; (line: measured spectrum; bars: simulated spectrum).

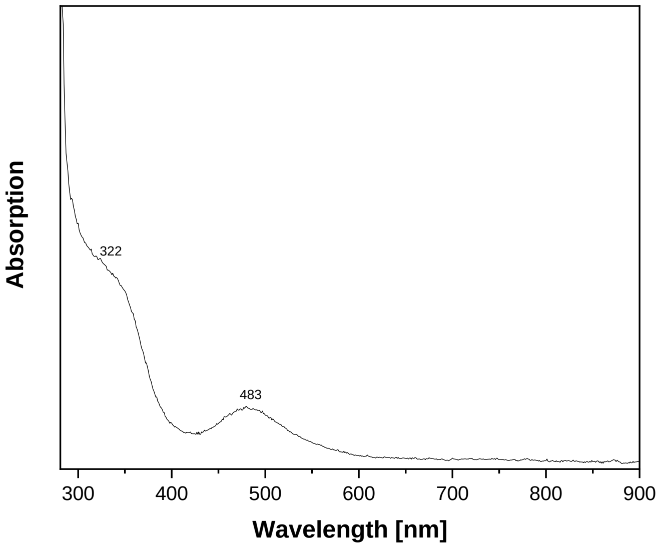

Figure S78 UV-Vis spectrum (280-900 nm) of [$^{t}Bu_3Si–Si(Cl)=Ru(IMe_4)$(*p*-cymene)] (**10**) in toluene at room temperature (c = $5.0×10^{-5}$ $^{mol}/_{L}$). λ_{max} = 322 nm & 483 nm.

Note: *We also attempted reduction of Rh complex* ***7a*** *with two eq. of* KC_8 *as well as reduction of* ***9*** *with one equivalent of* KC_8*. In both cases a deep purple compound can be obtained. Based on the* ^{29}Si *NMR shifts (δ(*^{29}Si*) [ppm] = 260.7 (d,* $^{1}J_{Si–Rh}$ *= 130.3 Hz, SiRh), 1.6 (d,* $^{2}J_{Si–Rh}$ *= 7.1 Hz,* $^{t}Bu_3Si$*)) and the LIFDI-MS data (Figure S79), we presume this purple complex is the to* ***10*** *analogous Rh=Si complex* $^{t}Bu_3Si$*–Si(Cl)=Rh(*IMe_4*)(Cp*). However, we have so far been unable to obtain satisfactory analytical data and therefore this is only included as additional information for the sake of completeness.*

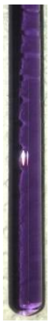

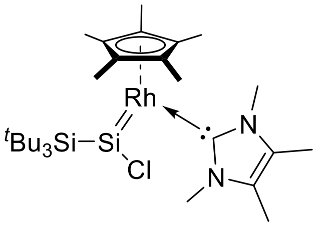

$C_{29}H_{54}ClN_2RhSi_2$
625.29 g/mol

LIFDI-MS: calculated: 624.2569 ($C_{29}H_{54}ClN_2RhSi_2$).
measured: 624.3738 ($^{t}Bu_3Si$–Si(Cl)=Rh(IMe_4)(Cp*)).

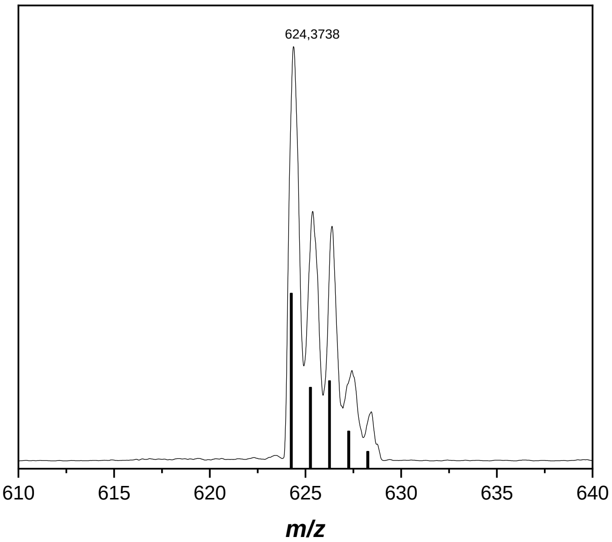

Figure S79 LIFDI-MS spectrum (detailed view) of [$^{t}Bu_3Si$–Si(Cl)=Rh(IMe_4)(Cp*)] in toluene; (line: measured spectrum; bars: simulated spectrum).

2. X-ray Crystallographic Data

2.1 General Information

The X-ray intensity data of **2**, **4-RhCl$_3$Cp***, **6a**, **7a**, **7b**, and **8** were collected on an X-ray single crystal diffractometer equipped with a CMOS detector (Bruker Photon-100), an IMS microsource with MoKα radiation (λ = 0.71073 Å) and a Helios mirror optic by using the APEX III software package.[7] The X-ray intensity data of **9** and **10** were collected on an X-ray single crystal diffractometer equipped with a CMOS detector (Bruker Photon-100), a rotating anode (Bruker TXS) with MoKα radiation (λ = 0.71073 Å) and a Helios mirror optic by using the APEX III software package.[7] The measurements were performed on single crystals coated with the perfluorinated ether Fomblin® Y. The crystals were fixed on the top of a micro sampler, transferred to the diffractometer and frozen under a stream of cold nitrogen. A matrix scan was used to determine the initial lattice parameters. Reflections were merged and corrected for Lorenz and polarization effects, scan speed, and background using SAINT.[8] Absorption corrections, including odd and even ordered spherical harmonics were performed using SADABS.[8] Space group assignments were based upon systematic absences, E statistics, and successful refinement of the structures. Structures were solved by direct methods with the aid of successive difference Fourier maps, and were refined against all data using the APEX III software in conjunction with SHELXL-2014[9] and SHELXLE.[10] All H atoms were placed in calculated positions and refined using a riding model, with methylene and aromatic C–H distances of 0.99 and 0.95 Å, respectively, and Uiso(H) = 1.2·Ueq(C). Full-matrix least-squares refinements were carried out by minimizing $\Delta w(F_o^2–F_c^2)^2$ with SHELXL-97 weighting scheme.[11] Neutral atom scattering factors for all atoms and anomalous dispersion corrections for the non-hydrogen atoms were taken from International Tables for Crystallography.[12] The images of the crystal structures were generated by Mercury.[13] Standard uncertainties of bond distances and (dihedral) angles that include the centroids of aryl ligands were calculated using Diamond 4.6.2.[14] The CCDC numbers CCDC-1976774 (**2**), CCDC-1976772 (**4-RhCl$_3$Cp***), CCDC-1976773 (**6a**), CCDC-1976776 (**7a**), CCDC-1976775 (**7b**), CCDC-1976777 (**8**), CCDC-1976778 (**9**) and CCDC-1976779 (**10**) contain the supplementary crystallographic data for the structures. These data can be obtained free of charge from the Cambridge Crystallographic Data Centre *via* https://www.ccdc.cam.ac.uk/structures/.

2.2 SC-XRD structure of 2 (CCDC-1976774)

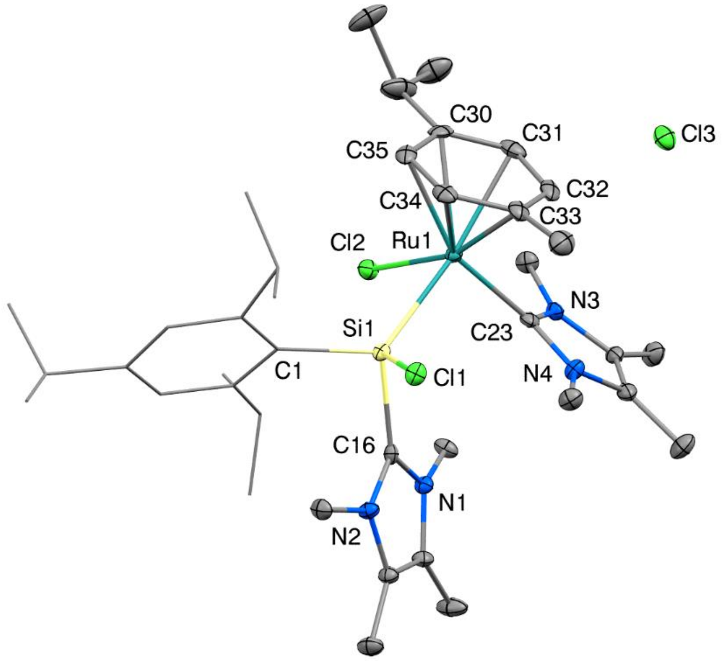

Figure S80 Ellipsoid plot (50% probability level) of the molecular structure of complex **2**. Hydrogen atoms and solvent molecules are omitted and the Tipp substituent is simplified as a wireframe for clarity. Selected bond lengths [Å] and angles [°]: Si1–Ru1 2.409(1), Si1–Cl1 2.167(1), Si1–C1 1.941(4), Si1–C16 1.970(4), Ru1–Cl2 2.404(1), Ru1–C23 2.077(4), Ru1–*p*-cym$_{\perp}$ 1.770(1), C1–Si1–Ru1 123.3(1), Si1–Ru1–*p*-cym$_{\perp}$ 131.1(1), Cl1–Si1–Ru1–Cl2 –173.1(1), C16–Si1–Ru1–C23 –11.1(2).

2.3 SC-XRD structure of 4-$RhCl_3Cp^*$ (CCDC-1976772)

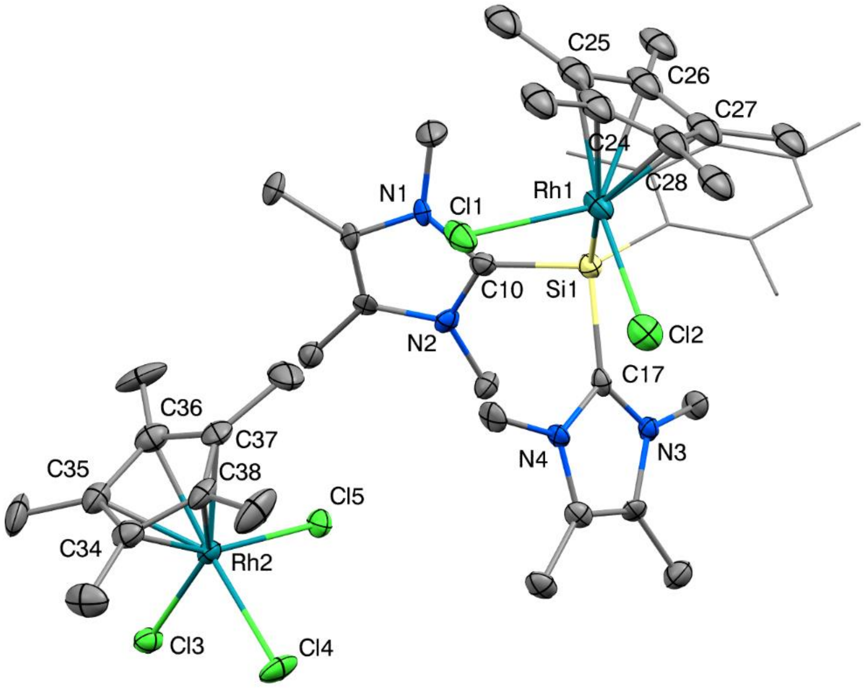

Figure S81 Ellipsoid plot (50% probability level) of the molecular structure of complex **4-$RhCl_3Cp^*$**. Hydrogen atoms and solvent molecules are omitted and the mesityl substituent is simplified as a wireframe for clarity. Selected bond lengths [Å] and angles [°]: Si1–Rh1 2.426(2), Si1–C1 1.899(7), Si1–C10 1.958(7), Si1–C17 1.944(7), Rh1–Cl1 2.420(2), Rh1–Cl2 2.404(2), Rh1–$Cp^*_{\perp}$ 1.857(1), Si1–Rh1–$Cp^*_{\perp}$ 132.3(1), Si1–Rh1–Cl1 94.6(1), Si1–Rh1–Cl2 88.9(1), C10–Si1–C17 93.9(3), C1–Si1–C10 110.3(3), C1–Si1–C17 104.4(3), C1–Si1–Rh1 112.7(2), C10–Si1–Rh1 115.3(2), C17–Si1–Rh1 118.5(2), Cl1–Rh1–Cl2 91.6(1), C1–Si1–Rh1–$Cp^*_{\perp}$ 24.2(2), C1–Si1–Rh1–Cl1 157.0(2), C1–Si1–Rh1–Cl2 –111.5(2), C10–Si1–Rh1–Cl1 29.1(2), C10–Si1–Rh1–Cl2 120.6(2), C17–Si1–Rh1–Cl1 –80.8(2), C17–Si1–Rh1–Cl2 10.7(2).

2.4 SC-XRD structure of 6a (CCDC-1976773)

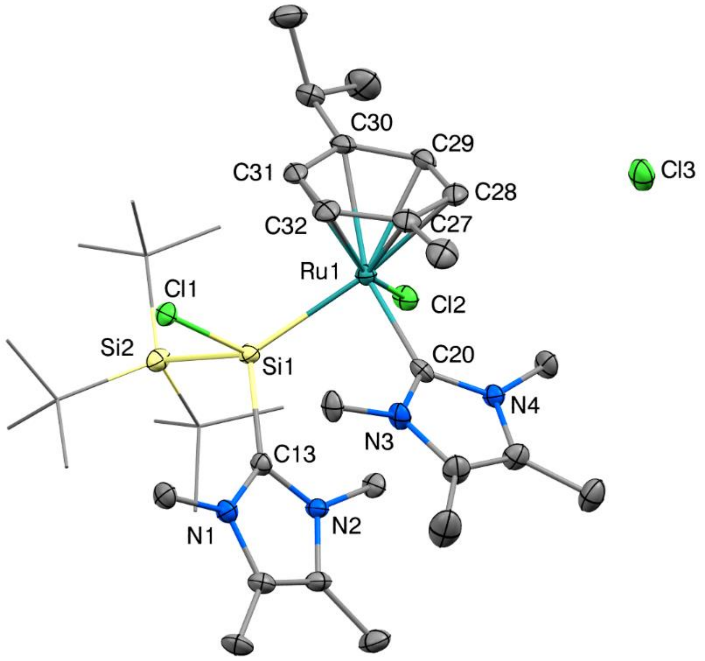

Figure S82 Ellipsoid plot (50% probability level) of the molecular structure of complex **6a**. Hydrogen atoms and solvent molecules are omitted and the ${}^{t}Bu_3Si$ substituent is simplified as a wireframe for clarity. Selected bond lengths [Å] and angles [°]: Si1–Ru1 2.499(1), Si1–Cl1 2.166(2), Si1–Si2 2.551(2), Si1–C13 2.002(4), Ru1–Cl2 2.427(1), Ru1–C20 2.084(3), Ru1–*p*-cym$_{\perp}$ 1.767(1), Si2–Si1–Ru1 132.6(1), Si1–Ru1–*p*-cym$_{\perp}$ 130.4(1), Cl1–Si1–Ru1–Cl2 –156.1(1), C13–Si1–Ru1–C20 –0.2(2).

2.5 SC-XRD structure of 7a (CCDC-1976776)

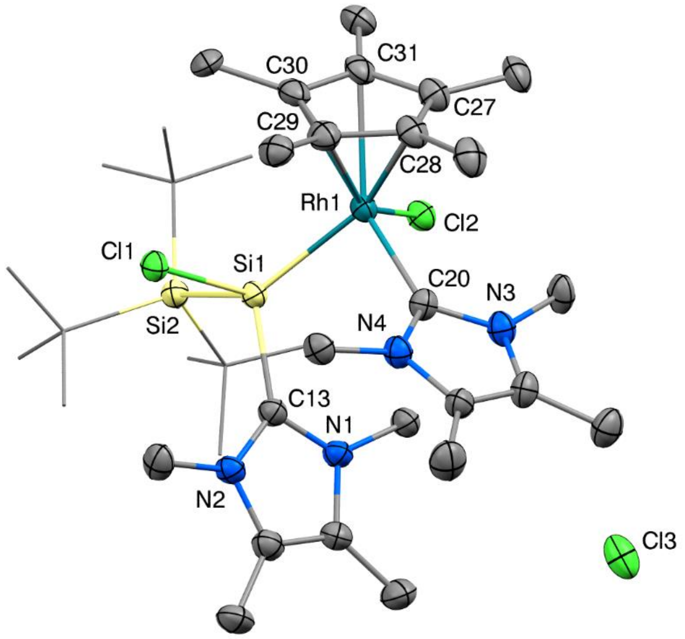

Figure S83 Ellipsoid plot (50% probability level) of the molecular structure of complex **7a**. Hydrogen atoms and solvent molecules are omitted and the $^{t}Bu_3Si$ substituent is simplified as a wireframe for clarity. Selected bond lengths [Å] and angles [°]: Si1–Rh1 2.423(2), Si1–Cl1 2.159(3), Si1–Si2 2.560(3), Si1–C13 1.999(7), Rh1–Cl2 2.424(2), Rh1–C20 2.061(6), Rh1–Cp*$_{\perp}$ 1.895(1), Si2–Si1–Rh1 130.4(1), Si1–Rh1–Cp*$_{\perp}$ 132.4(1), Cl1–Si1–Rh1–Cl2 –154.8(1), C13–Si1–Rh1–C20 –4.2(3).

2.6 SC-XRD structure of 7b (CCDC-1976775)

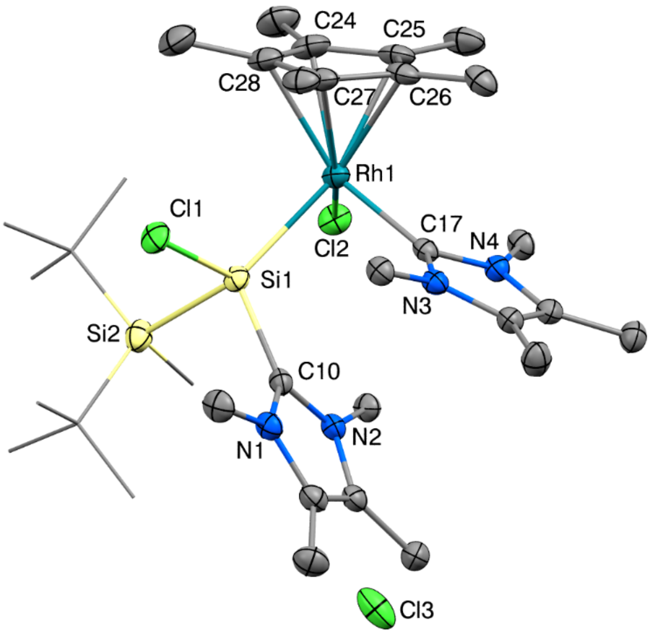

Figure S84 Ellipsoid plot (50% probability level) of the molecular structure of complex **7b**. Hydrogen atoms are omitted and the $^{t}Bu_2MeSi$ substituent is simplified as a wireframe for clarity. Selected bond lengths [Å] and angles [°]: Si1–Rh1 2.384(1), Si1–Cl1 2.144(1), Rh1–Cl2 2.415(1), Si1–Si2 2.438(1), Si1–C10 1.978(2), Rh1–C17 2.037(2), Rh1–Cp*$_{\perp}$ 1.890(1), Si2–Si1–Rh1 128.2(1), Si1–Rh1–Cp*$_{\perp}$ 133.6(1), Cl1–Si1–Rh1–Cl2 –146.8(1), Cl1–Si1–Rh1–Cp*$_{\perp}$ –20.5(1), Cl1–Si1–Rh1–C17 114.3(1), C10–Si1–Rh1–C17 –1.0(1).

2.7 SC-XRD structure of 8 (CCDC-1976777)

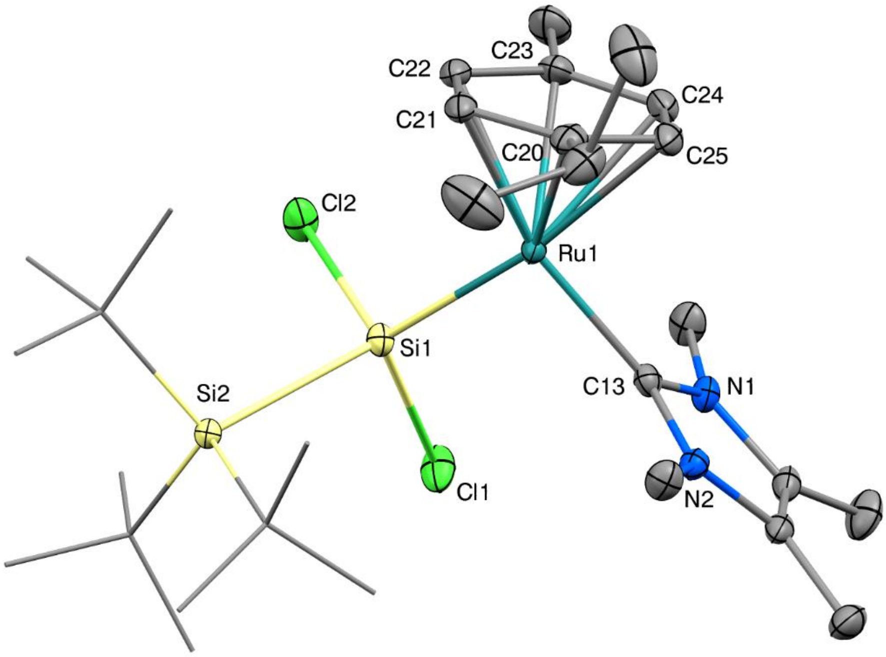

Figure S85 Ellipsoid plot (50% probability level) of the molecular structure of complex **8**. Hydrogen atoms are omitted and the $^{t}Bu_3Si$ substituent is simplified as a wireframe for clarity. Selected bond lengths [Å] and angles [°]: Si1–Ru1 2.374(1), Si1–Cl1 2.161(1), Si1–Cl2 2.160(1), Si1–Si2 2.424(1), Ru1–C13 2.064(2), Ru1–*p*-cym$_{\perp}$ 1.756(1), Si2–Si1–Ru1 128.0(1), Si1–Ru1–*p*-cym$_{\perp}$ 134.0(1), Cl1–Si1–Ru1–*p*-cym$_{\perp}$ 139.1(1), Cl1–Si1–Ru1–C13 –16.9(1), Cl2–Si1–Ru1–*p*-cym$_{\perp}$ 29.1(1), Cl2–Si1–Ru1–C13 –126.9(1).

2.8 SC-XRD structure of 9 (CCDC-1976778)

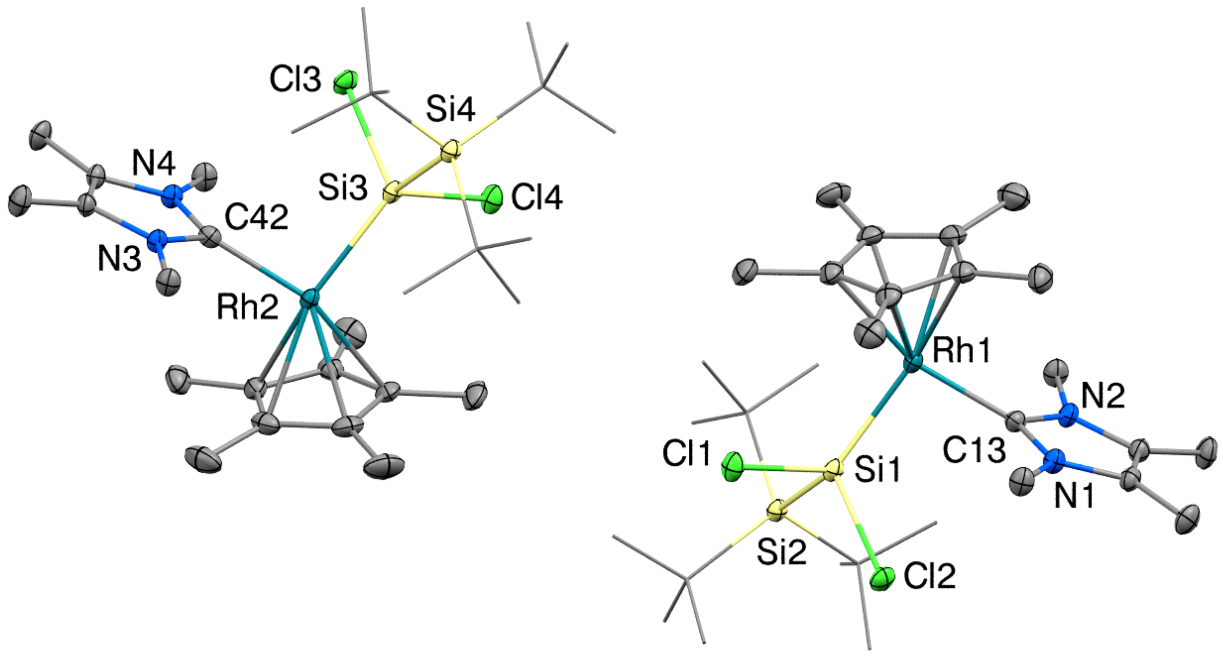

Figure S86 Ellipsoid plot (50% probability level) of the molecular structure of complex **9**. Hydrogen atoms and solvent molecules are omitted and the $^{t}Bu_3Si$ substituents are simplified as a wireframe for clarity. Selected bond lengths [Å] and angles [°]: Si1–Rh1 2.328(1), Si1–Cl1 2.145(1), Si1–Cl2 2.170(1), Si1–Si2 2.430(1), Rh1–C13 2.033(3), Rh1–Cp*$_\perp$ 1.911(1), Si2–Si1–Rh1 126.6(1), Si1–Rh1–Cp*$_\perp$ 135.7(1), Cl1–Si1–Rh1–Cp*$_\perp$ –16.1(1), Cl1–Si1–Rh1–C13 141.0(1), Cl2–Si1–Rh1–Cp*$_\perp$ –124.8(1), Cl2–Si1–Rh1–C13 32.3(1), Si3–Rh2 2.331(1), Si3–Cl3 2.146(1), Si3–Cl4 2.171(1), Si3–Si4 2.429(1), Rh2–C42 2.018(3), Rh2–Cp*$_\perp$ 1.909(1), Si4–Si3–Rh2 126.0(4), Si3–Rh2–Cp*$_\perp$ 136.1(1), Cl3–Si3–Rh2–Cp*$_\perp$ 16.1(1), Cl3–Si3–Rh2–C42 –140.8(1), Cl4–Si3–Rh2–Cp*$_\perp$ 125.4(1), Cl4–Si3–Rh2–C42 –31.6(1).

2.9 SC-XRD structure of 10 (CCDC-1976779)

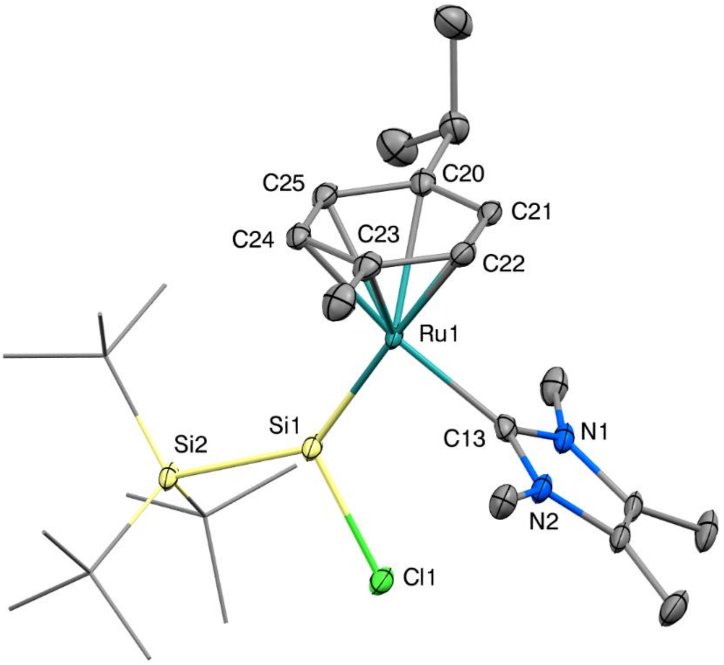

Figure S87 Ellipsoid plot (50% probability level) of the molecular structure of complex **10**. Hydrogen atoms are omitted and the $^{t}Bu_3Si$ substituent is simplified as a wireframe for clarity. Selected bond lengths [Å] and angles [°]: Si1–Ru1 2.236(1), Si1–Cl1 2.169(1), Si1–Si2 2.416(1), Ru1–C13 2.055(4), Ru1–*p*-cym$_{\perp}$ 1.751(1), Si2–Si1–Ru1 143.8(1), Si1–Ru1–*p*-cym$_{\perp}$ 147.7(1), Cl1–Si1–Ru1–*p*-cym$_{\perp}$ –175.0(1), Cl1–Si1–Ru1–C13 6.8(1).

2.10 Crystal data and structural refinement parameters

Table S1 Crystal data and structural refinement parameters for compounds **2**, **6a**, **7a** and **7b**.

Compound #	2	6a	7a	7b
**CCDC #**	1976774	1976773	1976776	1976775
**Chemical formula**	$C_{45}H_{70}Cl_3N_7RuSi$	$C_{38}H_{68}Cl_3N_5RuSi_2$	$C_{38}H_{69}Cl_3N_5RhSi_2$	$C_{33}H_{60}Cl_3N_4RhSi_2$
**Formula weight**	944.59	858.57	861.40	778.29
**Temperature**	100(2) K	100(2) K	100(2) K	100(2) K
**Wavelength**	0.71073 Å	0.71073 Å	0.71073 Å	0.71073 Å
**Crystal size**	0.316 × 0.334 × 0.494 mm	0.154 × 0.176 × 0.255 mm	0.101 × 0.116 × 0.168 mm	0.212 × 0.298 × 0.324 mm
**Crystal habit**	clear intense orange fragment	clear intense orange fragment	clear intense red fragment	clear orange-red fragment
**Crystal system**	orthorhombic	triclinic	tetragonal	monoclinic
**Space group**	P 21 21 21	P -1	I -4	P 21/c
**Unit cell dimensions**	a = 12.1974(10) Å, α = 90°<br>b = 12.7513(11) Å, α = 90°<br>c = 31.058(3) Å, α = 90°	a = 8.5873(19) Å, α = 100.272(8)°<br>b = 10.536(3) Å, β = 94.576(8)°<br>c = 25.732(6) Å, γ = 103.458(7)°	a = 30.924(7) Å, α = 90°<br>b = 30. 924(7) Å, β = 90°<br>c = 9.760(4) Å, γ = 90°	a = 12.2917(8) Å, α = 90°<br>b = 11.7135(8) Å, β = 95.774(2)°<br>c = 26.4973(18) Å, α = 90°
**Volume**	4830.5(7) $Å^3$	2209.9(10) $Å^3$	9333(6) $Å^3$	3795.7(4) $Å^3$
**Z**	4	1	8	4
**Density (calculated)**	1.299 g/cm^3	1.290 g/cm^3	1.226 g/cm^3	1.362 g/cm^3
**Absorption coefficient**	0.553 mm^{-1}	0.622 mm^{-1}	0.619 mm^{-1}	0.752 mm^{-1}
**F(000)**	1992	908	3648	1640
**Diffractometer**	Bruker D8 Venture Duo IMS	Bruker D8 Venture Duo IMS	Bruker D8 Venture Duo IMS	Bruker D8 Venture Duo IMS
**Radiation source**	IMS microsource, Mo	IMS microsource, Mo	IMS microsource, Mo	IMS microsource, Mo
**Theta range for data collection**	2.31 to 25.03°	2.03 to 25.35°	2.08 to 31.60°	2.48 to 25.35°
**Index ranges**	-14<=h<=14, -15<=k<=15, -36<=l<=36	-10<=h<=10, -12<=k<=12, -30<=l<=30	-44<=h<=45, -45<=k<=45, -13<=l<=14	-14<=h<=14, -14<=k<=14, -31<=l<=31
**Reflections collected**	175495	102323	246899	99017
**Independent reflections**	8526 [R(int) = 0.0955]	8108 [R(int) = 0.0556]	14537 [R(int) = 0.0769]	6937 [R(int) = 0.0365]
**Coverage of independent reflections**	99.9%	99.9%	95.7%	99.9%
**Absorption correction**	Multi-Scan	Multi-Scan	Multi-Scan	Multi-Scan
**Refinement method**	Full-matrix least-squares on F^2	Full-matrix least-squares on F^2	Full-matrix least-squares on F^2	Full-matrix least-squares on F^2
**Refinement program**	SHELXL-2016/6 (Sheldrick, 2016)	SHELXL-2016/6 (Sheldrick, 2016)	SHELXL-2017/1 (Sheldrick, 2017)	SHELXL-2016/6 (Sheldrick, 2016)
**Function minimized**	Σ w(F_o^2 - F_c^2)^2	Σ w(F_o^2 - F_c^2)^2	Σ w(F_o^2 - F_c^2)^2	Σ w(F_o^2 - F_c^2)^2
**Data / restraints / parameters**	8526 / 66 / 593	8108 / 0 / 463	14537 / 0 / 468	6937 / 0 / 408
**Goodness-of-fit on F^2**	1.046	1.167	1.117	1.062
**Final R indices**	7841 data; I>2σ(I): R1 = 0.0292, wR2 = 0.0497<br>all data: R1 = 0.0366, wR2 = 0.0517	7467 data; I>2σ(I): R1 = 0.0469, wR2 = 0.1131<br>all data: R1 = 0.0517, wR2 = 0.1165	10905 data; I>2σ(I): R1 = 0.0467, wR2 = 0.0978<br>all data: R1 = 0.1096, wR2 = 0.1492	6315 data; I>2σ(I): R1 = 0.0294, wR2 = 0.0713<br>all data: R1 = 0.0342, wR2 = 0.0760
**Weighting scheme**	w=1/[σ^2(F_o^2)+(0.0159P)^2+3.1813P]<br>where P=(F_o^2+2F_c^2)/3	w=1/[σ^2(F_o^2)+(0.0202P)^2+11.3934P]<br>where P=(F_o^2+2F_c^2)/3	w=1/[σ^2(F_o^2)+(0.0218P)^2+94.0608P]<br>where P=(F_o^2+2F_c^2)/3	w=1/[σ^2(F_o^2)+(0.0305P)^2+6.5835P]<br>where P=(F_o^2+2F_c^2)/3
**Largest diff. peak and hole**	0.335 and -0.344 e$Å^{-3}$	2.224 and -0.744 e$Å^{-3}$	0.1.785 and -1.871 e$Å^{-3}$	1.777 and -0.701 e$Å^{-3}$
**R.M.S. deviation from mean**	0.057 e$Å^{-3}$	0.104 e$Å^{-3}$	0.169 e$Å^{-3}$	0.066 e$Å^{-3}$

Table S2 Crystal data and structural refinement parameters for compounds **4-RhCl$_3$Cp***, **8**, **9**, **10**.

Compound #	4-RhCl$_3$Cp*	8	9	10
**CCDC #**	1976772	1976777	1976778	1976779
**Chemical formula**	$C_{45}H_{68}Cl_5N_5Rh_2Si$	$C_{29}H_{53}Cl_2N_2RuSi_2$	$C_{65}H_{116}Cl_4N_4Rh_2Si_4$	$C_{29}H_{53}ClN_2RuSi_2$
**Formula weight**	1090.20	657.88	1413.60	622.43
**Temperature**	100(2) K	100(2) K	100(2) K	103(2) K
**Wavelength**	0.71073 Å	0.71073 Å	0.71073 Å	0.71073 Å
**Crystal size**	0.098 × 0.160 × 0.258 mm	0.100 × 0.204 × 0.256 mm	0.039 × 0.072 × 0.189 mm	0.083 × 0.139 × 0.239 mm
**Crystal habit**	clear intense red fragment	clear intense green fragment	grey-black fragment	clear intense red plate
**Crystal system**	orthorhombic	triclinic	triclinic	monoclinic
**Space group**	P 21 21 21	P -1	P -1	P 21/n
**Unit cell dimensions**	a = 9.0670(11) Å, α = 90°	a = 8.7307(6) Å, α = 104.544(4)°	a = 8.4991(8) Å, α = 85.097(3)°	a = 13.072(3) Å, α = 90°
	b = 17.650(3) Å, α = 90°	b = 11.6729(10) Å, β = 93.919(3)°	b = 19.5364(19) Å, β = 80.082(3)°	b = 14.981(3) Å, β = 107.024(11)°
	c = 32.302(5) Å, α = 90°	c = 18.1360(15) Å, γ = 111.578(2)°	c = 23.877(2) Å, γ = 79.898(3)°	c = 17.516(4) Å, γ = 90°
**Volume**	5169.4(13) Å^3	1637.0(2) Å^3	3838.5(6) Å^3	3279.9(13) Å^3
**Z**	4	2	2	4
**Density (calculated)**	1.401 g/cm^3	1.335 g/cm^3	1.223 g/cm^3	1.260 g/cm^3
**Absorption coefficient**	0.955 mm^{-1}	0.736 mm^{-1}	0.669 mm^{-1}	0.652 mm^{-1}
**F(000)**	2248	694	1496	1320
**Diffractometer**	Bruker D8 Venture Duo IMS	Bruker D8 Venture Duo IMS	Bruker D8 Venture	Bruker D8 Venture
**Radiation source**	IMS microsource, Mo	IMS microsource, Mo	TXS rotating anode, Mo	TXS rotating anode, Mo
**Theta range for data collection**	2.22 to 25.35°	2.52 to 25.35°	1.98 to 25.68°	2.20 to 25.35°
**Index ranges**	-10<=h<=10, -21<=k<=21, -38<=l<=38	-10<=h<=10, -14<=k<=14, -21<=l<=21	-10<=h<=10, -23<=k<=23, -29<=l<=29	-15<=h<=15, -18<=k<=18, -21<=l<=21
**Reflections collected**	140074	43724	138631	99553
**Independent reflections**	9464 [R(int) = 0.0865]	5975 [R(int) = 0.0236]	14584 [R(int) = 0.0496]	6001 [R(int) = 0.0985]
**Coverage of independent reflections**	99.9%	99.6%	100.0%	100.0%
**Absorption correction**	Multi-Scan	Multi-Scan	Multi-Scan	Multi-Scan
**Refinement method**	Full-matrix least-squares on F^2	Full-matrix least-squares on F^2	Full-matrix least-squares on F^2	Full-matrix least-squares on F^2
**Refinement program**	SHELXL-2017/1 (Sheldrick, 2017)	SHELXL-2016/6 (Sheldrick, 2016)	SHELXL-2017/1 (Sheldrick, 2017)	SHELXL-2017/1 (Sheldrick, 2017)
**Function minimized**	Σ w(F_o^2 - F_c^2)^2	Σ w(F_o^2 - F_c^2)^2	Σ w(F_o^2 - F_c^2)^2	Σ w(F_o^2 - F_c^2)^2
**Data / restraints / parameters**	9464 / 390 / 738	5975 / 0 / 341	14584 / 0 / 749	6001 / 0 / 332
**Goodness-of-fit on F^2**	1.079	1.040	1.035	1.136
**Final R indices**	8907 data; I>2σ(I): R1 = 0.0413, wR2 = 0.1016	5762 data; I>2σ(I): R1 = 0.0169, wR2 = 0.0415	11939 data; I>2σ(I): R1 = 0.0355, wR2 = 0.0854	5145 data; I>2σ(I): R1 = 0.0407, wR2 = 0.1039
	all data: R1 = 0.0464, wR2 = 0.1059	all data: R1 = 0.0179, wR2 = 0.0423	all data: R1 = 0.0470, wR2 = 0.915	all data: R1 = 0.0511, wR2 = 0.1093
**Weighting scheme**	w=1/[$\sigma^2(F_o^2)+(0.0486P)^2+14.8769P$]	w=1/[$\sigma^2(F_o^2)+(0.0164P)^2+1.1024P$]	w=1/[$\sigma^2(F_o^2)+(0.0412P)^2+6.7176P$]	w=1/[$\sigma^2(F_o^2)+(0.0473P)^2+8.3380P$]
	where P=(F_o^2+2F_c^2)/3	where P=(F_o^2+2F_c^2)/3	where P=(F_o^2+2F_c^2)/3	where P=(F_o^2+2F_c^2)/3
**Largest diff. peak and hole**	1.005 and -0.939 eÅ^{-3}	0.312 and -0.371 eÅ^{-3}	3.115 and -0.694 eÅ^{-3}	2.371 and -1.082 eÅ^{-3}
**R.M.S. deviation from mean**	0.107 eÅ^{-3}	0.042 eÅ^{-3}	0.081 eÅ^{-3}	0.110 eÅ^{-3}

3. DFT Calculations

DFT calculations were carried out at the B97-D/def2-SVP level of theory using Gaussian 09.[15-18] Stationary points on the potential energy surface were characterized by harmonic vibrational frequency calculations. NMR shifts were obtained at the HCTH407/def2-SVP//B97-D/def2-SVP level of theory.[19] Table S3 to Table S9 contain the respective relevant data for the NBO analysis of the cationic parts of complexes **2**, **4**, **6a**, **7a**, **8**, **9** and **10**. Table S10 contains the calculated Si–M and Si–C_{NHC} bond lengths, WBIs/MBOs and NPA charges. Figure S88 to Figure S94 contain the respective HOMOs and LUMOs for complexes **2**, **4**, **6a**, **7a**, **8**, **9** and **10** and the detailed calculated mechanism for the formation of complex **2** *via* **2’** is depicted in Figure S95. Table S11 contains the Cartesian coordinates for the calculated structures.

3.1 NBO Analysis of Complexes 2, 4, 6a, 7a, 8, 9 and 10

Table S3 NBO-Analysis of the central Si–Ru moiety in **2**.

3	Occupation	Atom	Polarization	*s*-character	*p*-character	*d*-character
Bond	1.91	Si	43.71%	45.86%	54.04%	0.10%
		Ru	56.29%	14.46%	6.82%	78.71%
Lone pair	1.51	C_{NHC}	×	36.96%	63.04%	0.00%
Empty orbital	0.53	Si	×	14.79%	84.90%	0.31%

Table S4 NBO-Analysis of the central Si–Rh moiety in **4**.

7	Occupation	Atom	Polarization	*s*-character	*p*-character	*d*-character
Bond	1.81	Si	52.57%	32.80%	67.08%	0.12%
		Rh	47.43%	39.03%	6.92%	54.04%
Bond	1.94	C_{NHC}	75.79%	44.15%	55.85%	0.01%
		Si	24.21%	19.79%	79.39%	0.81%
Bond	1.94	C_{NHC}	27.36%	27.70%	71.63%	0.68%
		Si	72.64%	30.69%	69.29%	0.02%

Table S5 NBO-Analysis of the central Si–Ru moiety in **6a**.

4a	Occupation	Atom	Polarization	*s*-character	*p*-character	*d*-character
Bond	1.81	Si	52.26%	37.80%	62.11%	0.08%
		Ru	47.74%	36.27%	10.96%	52.76%
Bond	1.95	C_{NHC}	78.01%	24.20%	75.53%	0.27%
		Si	21.99%	13.01%	85.98%	1.01%

Table S6 NBO-Analysis of the central Si–Rh moiety in **7a**.

5a	Occupation	Atom	Polarization	*s*-character	*p*-character	*d*-character
Bond	1.79	Si	52.23%	35.60%	64.27%	0.13%
		Rh	47.77%	38.03%	8.80%	53.15%
Bond	1.95	C_{NHC}	76.10%	43.65%	56.35%	0.00%
		Si	23.90%	18.71%	80.44%	0.84%

Table S7 NBO-Analysis of the central Si–Ru moiety in **8**.

8	Occupation	Atom	Polarization	*s*-character	*p*-character	*d*-character
Bond (alpha)	0.96	Si	46.97%	39.33%	60.61%	0.06%
		Ru	53.03%	14.86%	5.83%	79.31%
Bond (beta)	0.92	Si	54.41%	39.26%	60.64%	0.10%
		Ru	45.59%	37.53%	8.72%	53.72%

Table S8 NBO-Analysis of the central Si–Rh moiety in **9**.

9	Occupation	Atom	Polarization	*s*-character	*p*-character	*d*-character
Bond (alpha)	0.97	Si	45.52%	37.69%	62.23%	0.08%
		Rh	54.48%	14.87%	3.69%	81.43%
Bond (beta)	0.93	Si	52.15%	37.30%	62.59%	0.11%
		Rh	47.85%	30.21%	5.17%	64.61%

Table S9 NBO-Analysis of the central Si–Ru moiety in **10**.

10	Occupation	Atom	Polarization	*s*-character	*p*-character	*d*-character
Bond	1.82	Si	57.16%	53.72%	46.18%	0.10%
		Ru	42.84%	44.44%	15.88%	39.66%
Bond	1.91	Si	29.43%	5.19%	94.58%	0.23%
		Ru	70.57%	3.93%	4.82%	91.25%

Table S10 Calculated Si–M and Si–C_{NHC} bond lengths [Å], NPA charges of Si and Metal atoms, Wiberg Bond Index (WBI) and Mayer Bond Order (MBO) in studied transition metal complexes.

#	Bond length [Å]			NPA charge		WBI/MBO		
	Si–M	Si–C_{NHC}	Si–C_{NHC}	Si	Metal	Si–M	Si–C_{NHC}	Si–C_{NHC}
**2**	2.392	1.997	×	+1.31	–0.56	0.73/0.83	0.66/0.81	×
**4**	2.365	1.988	1.986	+1.23	–0.25	0.64/0.81	0.69/0.80	0.69/0.80
**6a**	2.481	2.030	×	+0.81	–0.53	0.73/0.72	0.69/0.82	×
**7a**	2.428	2.029	×	+0.74	–0.21	0.64/0.70	0.69/0.81	×
**8**	2.371	×	×	+0.74	–0.46	0.60/0.86	×	×
**9**	2.324	×	×	+0.68	–0.21	0.68/0.91	×	×
**10**	2.225	×	×	+0.62	–0.73	1.35/1.52	×	×

3.2 HOMOs and LUMOs of Complexes 2, 4, 6a, 7a, 8, 9 and 10

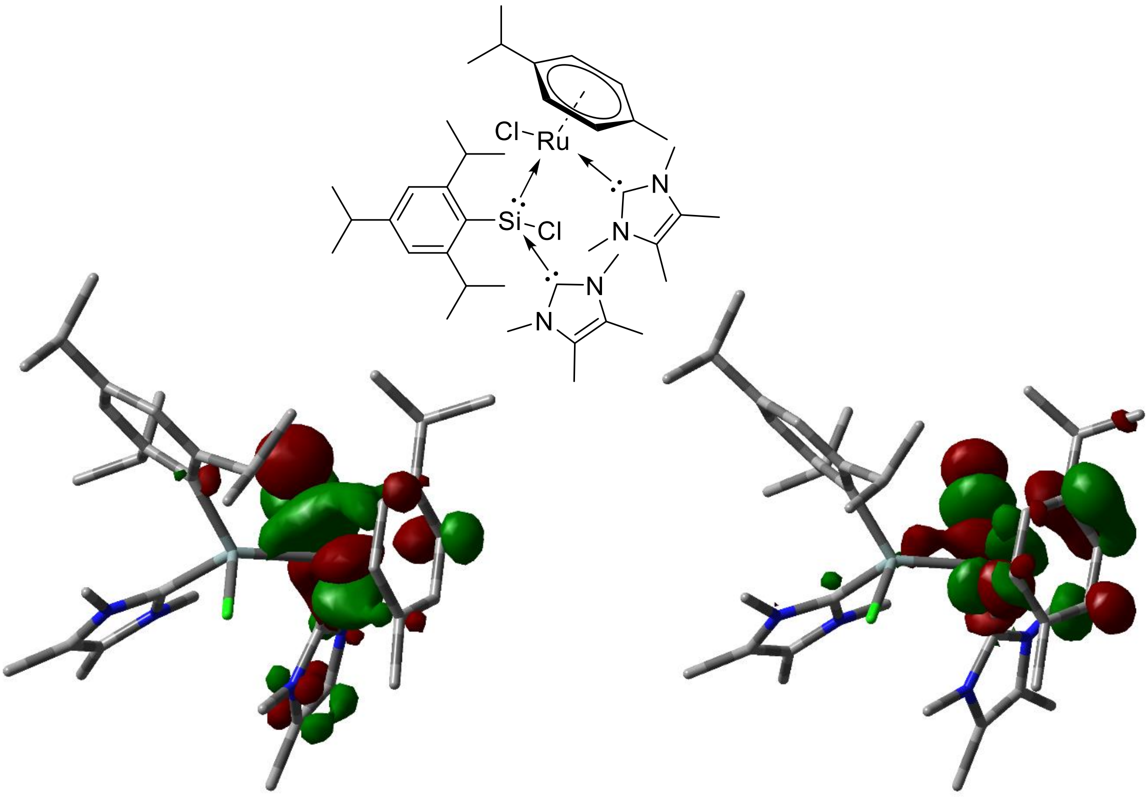

Figure S88 HOMO (left, –7.06 eV) and LUMO (right, –4.46 eV) of **2**.

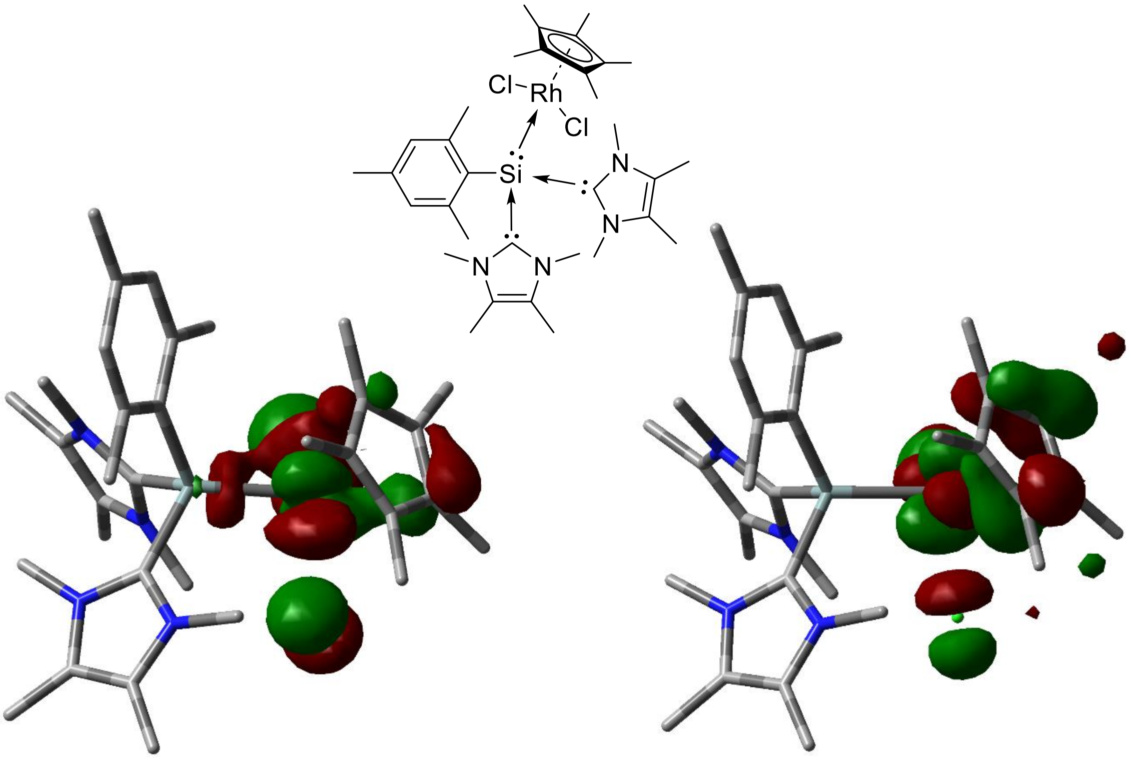

Figure S89 HOMO (left, –6.55 eV) and LUMO (right, –4.71 eV) of **4**.

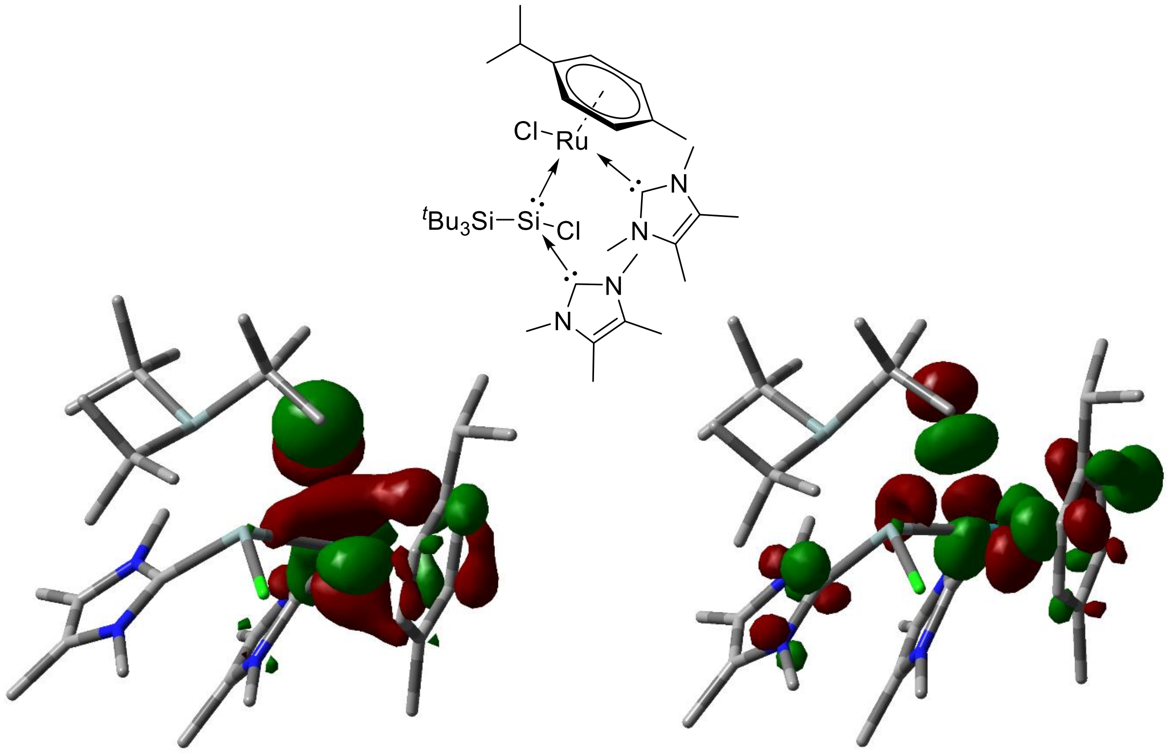

Figure S90 HOMO (left, –6.97 eV) and LUMO (right, –4.62 eV) of **6a**.

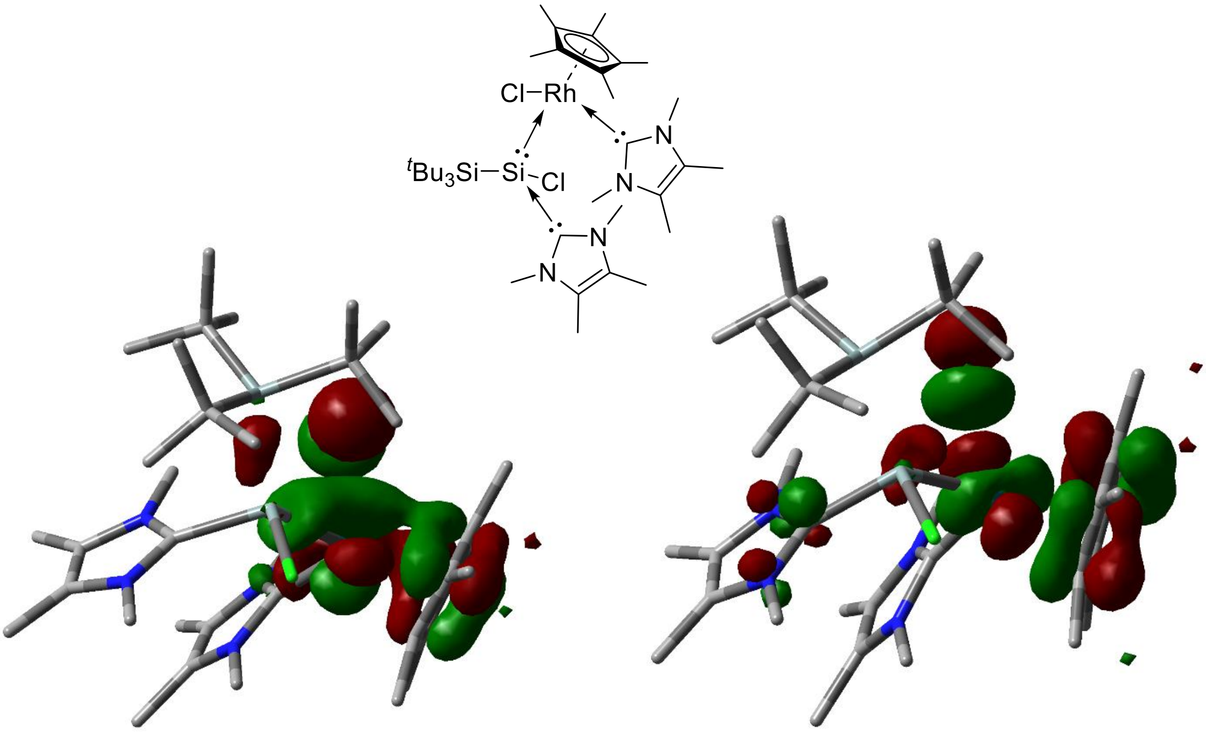

Figure S91 HOMO (left, –7.01 eV) and LUMO (right, –4.82 eV) of **7a**.

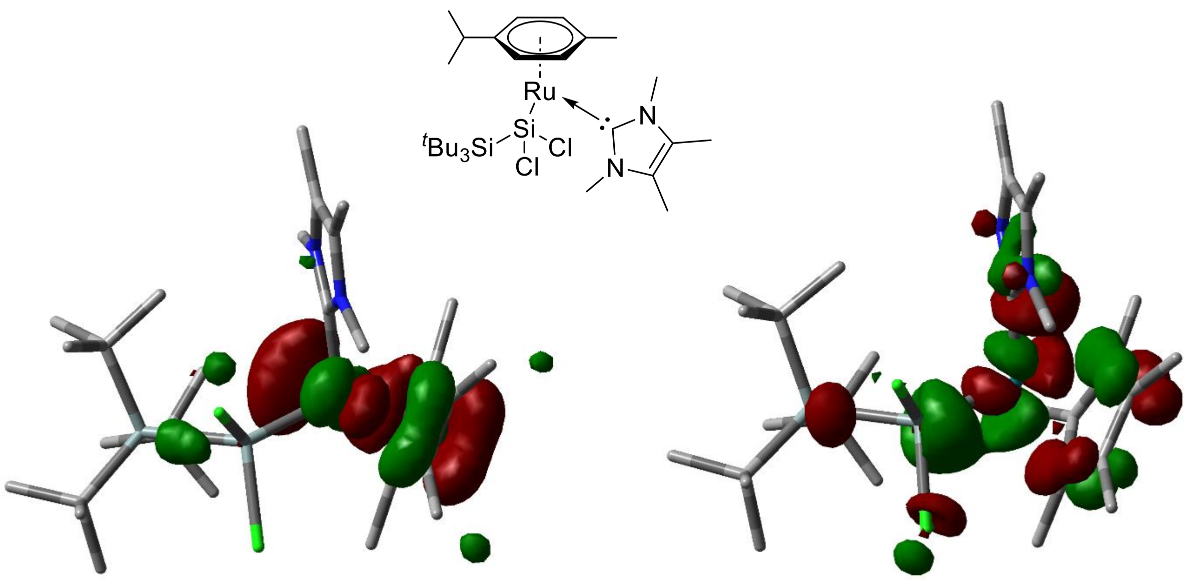

Figure S92 HOMO (left, –3.55 eV) and LUMO (right, –2.58 eV) of **8**.

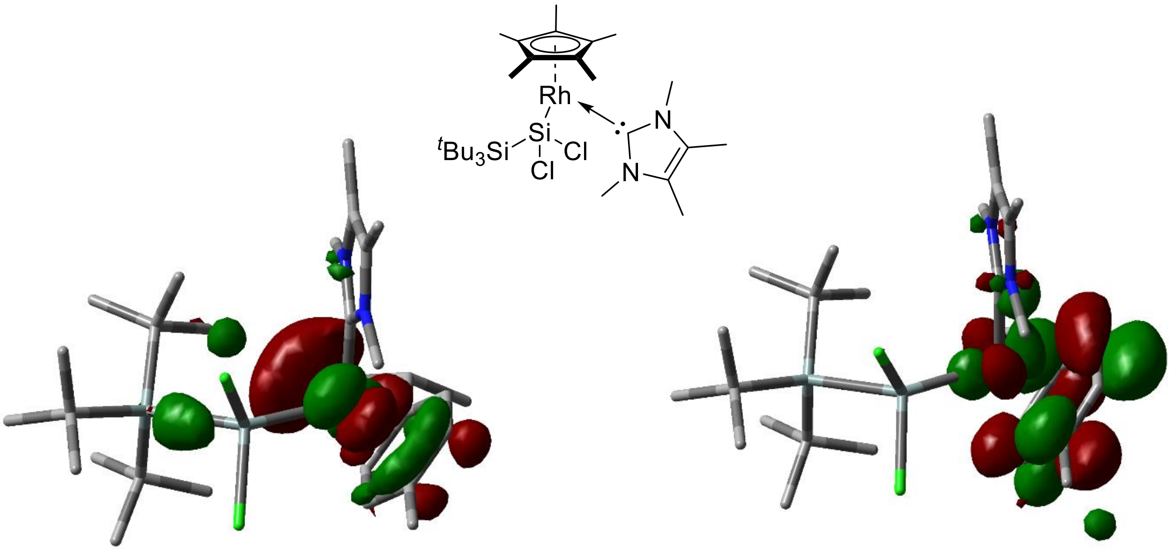

Figure S93 HOMO (left, –3.50 eV) and LUMO (right, –2.80 eV) of **9**.

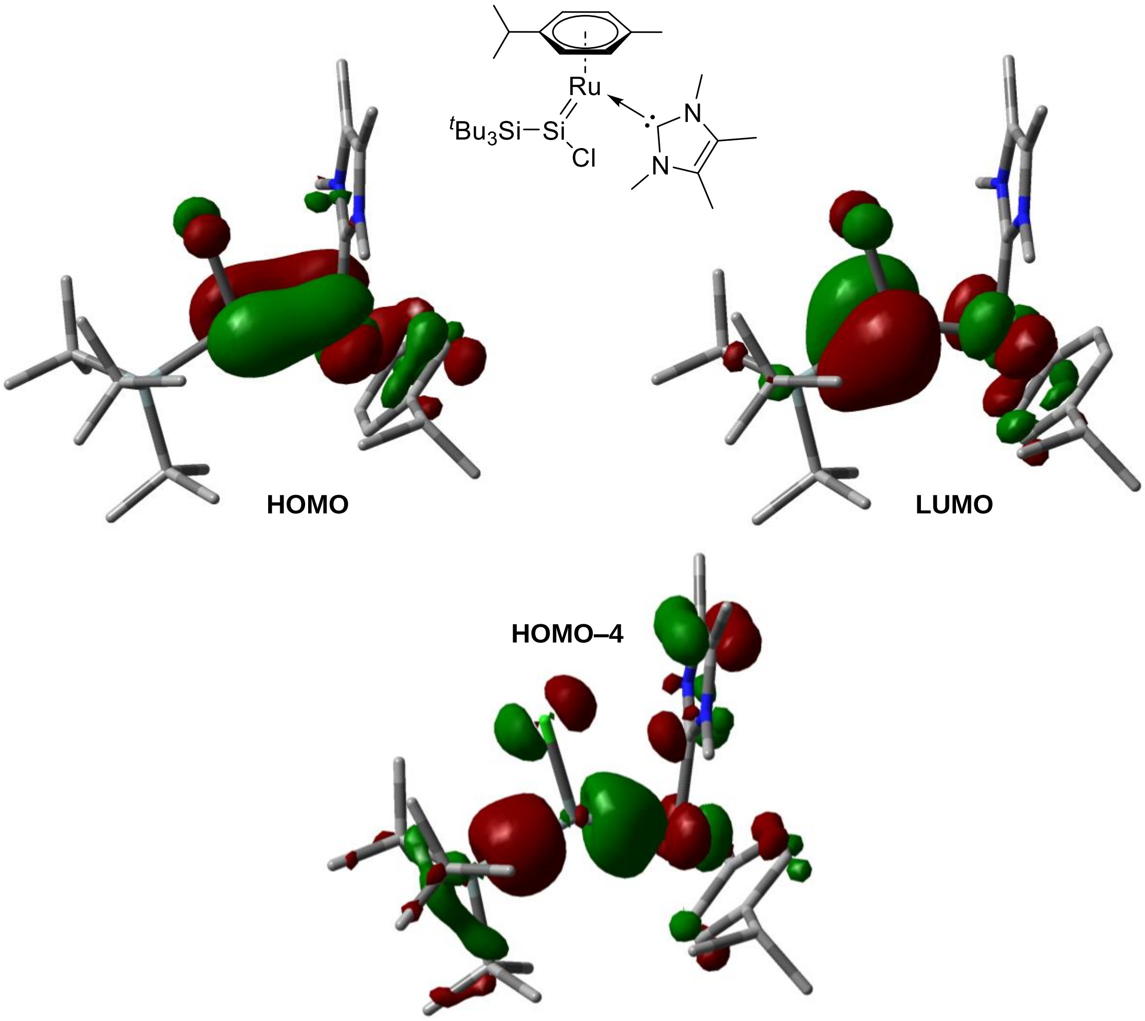

Figure S94 HOMO (top left, –3.52 eV; bonding orbital of Si–Ru π-bond), LUMO (top right, –1.43 eV; anti-bonding orbital of the Si–Ru π-bond) and HOMO–4 (bottom middle, –4.91 eV; σ-orbital of the Si–Ru bond) of **10**.

3.3 Calculated Mechanism for the Formation of 2 *via* 2’

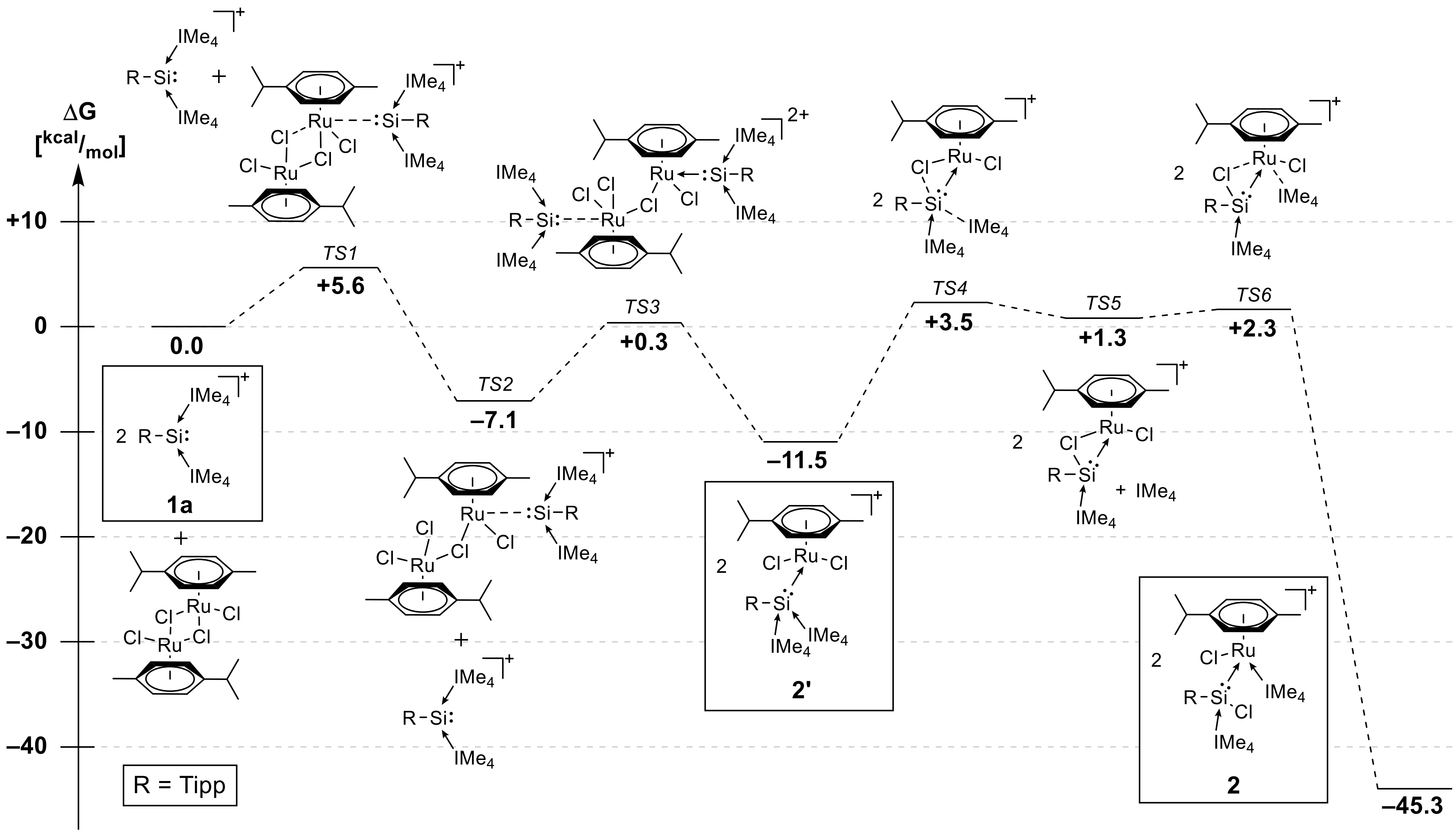

Figure S95 DFT-derived mechanism for the formation of **2** *via* **2’**.

3.4 Cartesian Coordinates of the Calculated Structures

Table S11 Cartesian coordinates of the calculated structures

2				4			
**Atomtype**	**X [Å]**	**Y [Å]**	**Z [Å]**	**Atomtype**	**X [Å]**	**Y Å]**	**Z [Å]**
N	0.931894	3.113872	0.88157	N	2.13126	-0.750307	2.118786
C	0.192414	2.161587	0.239417	C	1.922932	0.188473	1.157718
N	-0.722956	2.856971	-0.492854	N	2.866992	1.1557	1.401378
C	-0.595044	4.225749	-0.280497	C	3.605819	0.846444	2.542351
C	0.453037	4.389669	0.597078	C	3.135439	-0.366335	2.99457
Si	0.358907	0.194594	0.5423	Si	0.454904	0.116457	-0.18101
Ru	-1.437389	-1.327611	0.120783	Rh	-1.723396	-0.372155	0.599311
C	-2.914523	0.088865	-0.039598	Cl	-1.356097	-2.809482	0.694572
N	-3.912177	0.077466	-0.989672	C	3.261156	2.276622	0.544019
C	-4.850985	1.08949	-0.768386	C	4.673815	1.734336	3.092873
C	-4.447173	1.753989	0.361814	C	3.518596	-1.180085	4.185892
N	-3.277862	1.120335	0.793347	C	1.47819	-2.052694	2.228706
C	-4.101644	-0.907481	-2.050823	C	1.296678	-1.314517	-1.271745
C	-6.039309	1.30312	-1.648692	N	2.604238	-1.730167	-1.28564
C	-5.047816	2.920869	1.076536	C	2.738071	-2.933188	-1.979453
C	-2.57939	1.545326	1.993836	C	1.479471	-3.258568	-2.433923
C	-1.575872	2.336035	-1.560373	N	0.631698	-2.250436	-2.000164
C	-1.456523	5.23115	-0.970168	C	3.761462	-0.984915	-0.791541
C	1.060691	5.633646	1.156887	C	-0.760757	-2.185642	-2.417635
C	2.158598	2.909168	1.662411	C	0.99686	-4.429222	-3.224374
C	2.195702	-0.024532	-0.054127	C	4.035408	-3.656176	-2.144619
C	3.163598	-0.751129	0.71455	C	0.611758	1.804732	-1.078887
C	4.536003	-0.584105	0.452852	C	0.883619	1.968711	-2.472251
C	5.01399	0.246296	-0.572027	C	0.947513	3.260996	-3.031715
C	4.059497	0.864081	-1.39116	C	0.749424	4.421853	-2.265165
C	2.674127	0.730895	-1.176785	C	0.459341	4.252646	-0.900359
C	2.810821	-1.793883	1.778002	C	0.382319	2.984125	-0.294845
C	3.417401	-1.481442	3.161131	C	1.08711	0.800322	-3.41978
C	1.77408	1.346179	-2.252011	C	0.852242	5.799084	-2.878817
C	1.984035	0.613459	-3.59631	C	0.067925	2.943732	1.188073
C	6.504802	0.413538	-0.825601	Cl	-0.965306	-0.056003	2.889804
C	7.135194	-0.912452	-1.304541	C	-3.704144	0.619132	1.210325
Cl	0.559315	0.42575	2.744446	C	-3.989849	-0.685953	0.722486
C	1.978456	2.864037	-2.431864	C	-3.534048	-0.748552	-0.662583
C	7.23626	0.954603	0.420272	C	-2.96263	0.525929	-1.013307
C	3.257001	-3.197166	1.306066	C	-3.047687	1.377594	0.155579
Cl	-0.793497	-0.994438	-2.218461	C	-4.030593	1.157104	2.56482
C	-1.128996	-3.603258	-0.26982	C	-4.645962	-1.815077	1.451029
C	-2.547197	-3.343418	-0.208796	C	-3.830545	-1.902225	-1.567998
C	-3.107957	-2.668347	0.894031	C	-2.576491	0.998294	-2.382199
C	-2.296399	-2.208051	1.993144	C	-2.889692	2.864361	0.146981

C	-0.898268	-2.491196	1.922819	H	1.149523	3.361934	-4.106767
C	-0.323143	-3.220745	0.830045	H	0.272294	5.140584	-0.280653
C	-0.572546	-4.297322	-1.497617	H	1.155844	1.152461	-4.464471
C	0.940866	-4.117803	-1.679428	H	0.24824	0.085202	-3.363363
C	-2.908894	-1.543366	3.196938	H	2.014009	0.237285	-3.198459
C	-0.962726	-5.793574	-1.454242	H	1.861853	6.227495	-2.710927
H	4.409519	1.456562	-2.246494	H	0.123827	6.49696	-2.42515
H	5.25748	-1.137215	1.066076	H	0.68228	5.769981	-3.970639
H	0.724869	1.161351	-1.985008	H	0.954834	2.721007	1.808284
H	1.26319	3.261154	-3.178685	H	-0.66586	2.160294	1.438543
H	2.99669	3.084084	-2.80423	H	-0.327592	3.917686	1.524222
H	1.84168	3.421058	-1.488404	H	-0.802743	-1.974387	-3.502821
H	1.274119	0.998212	-4.353808	H	-1.257112	-1.393509	-1.846048
H	1.809701	-0.470076	-3.47771	H	-1.25711	-3.139058	-2.189562
H	3.012442	0.769576	-3.973705	H	1.839897	-5.093552	-3.476722
H	6.619197	1.156556	-1.64007	H	0.514191	-4.109588	-4.168188
H	8.31024	1.108893	0.202044	H	0.252322	-5.013789	-2.649493
H	7.161469	0.237862	1.261041	H	4.768302	-3.065043	-2.729452
H	6.80505	1.918307	0.752517	H	3.864988	-4.60438	-2.681054
H	8.20828	-0.76976	-1.535641	H	4.498378	-3.894273	-1.167247
H	6.627535	-1.290943	-2.211675	H	4.636537	-1.245878	-1.408249
H	7.056268	-1.688787	-0.518715	H	3.980386	-1.225323	0.263747
H	1.716956	-1.82026	1.890752	H	3.569357	0.090867	-0.893085
H	2.898305	-3.972914	2.01046	H	4.363171	2.314149	0.506756
H	4.360021	-3.264811	1.262345	H	2.879396	3.232682	0.940274
H	2.869717	-3.424046	0.296688	H	2.858661	2.136873	-0.46647
H	3.090229	-2.236351	3.902081	H	5.522373	1.850669	2.389531
H	3.108313	-0.484398	3.5202	H	5.067083	1.307528	4.030449
H	4.522879	-1.511591	3.119573	H	4.283424	2.746931	3.312213
H	-2.633433	2.56875	-1.350208	H	3.848572	-2.196518	3.896952
H	-1.433256	1.252082	-1.661389	H	2.658796	-1.295162	4.874004
H	-1.27659	2.821902	-2.505838	H	4.341253	-0.691099	4.733576
H	-1.214344	6.246301	-0.614038	H	2.24397	-2.840985	2.100752
H	-2.531392	5.042676	-0.781712	H	0.686335	-2.159504	1.472869
H	-1.304775	5.206493	-2.067393	H	0.999014	-2.14056	3.216102
H	0.465382	6.511966	0.856182	H	-4.048721	-2.738185	1.333337
H	2.095112	5.780353	0.787673	H	-5.661299	-2.004077	1.046393
H	1.103888	5.603133	2.262394	H	-4.736583	-1.59454	2.52794
H	2.882821	3.686394	1.36583	H	-3.456105	-1.733203	-2.591492
H	2.580782	1.92136	1.434116	H	-4.927973	-2.04289	-1.626502
H	1.945775	2.982477	2.742363	H	-3.393976	-2.832492	-1.161111
H	-4.323067	-0.388327	-2.999244	H	-1.709937	1.679224	-2.336741
H	-4.954087	-1.568113	-1.800921	H	-3.42014	1.556266	-2.837228
H	-3.179551	-1.488474	-2.173001	H	-2.334802	0.157651	-3.054413
H	-6.641392	2.147845	-1.273178	H	-2.088522	3.188939	-0.535373
H	-6.689508	0.406847	-1.680486	H	-2.685332	3.258465	1.155286
H	-5.742241	1.528625	-2.692024	H	-3.837839	3.322023	-0.20615

H	-5.949507	3.267658	0.543852
H	-4.338459	3.769855	1.145238
H	-5.343239	2.660099	2.112314
H	-3.298702	1.644118	2.825351
H	-2.082792	2.520257	1.835041
H	-1.828267	0.792992	2.252685
H	-3.176099	-3.629583	-1.056672
H	-4.176107	-2.420744	0.890785
H	-0.255529	-2.142021	2.735686
H	0.749458	-3.409775	0.81177
H	-3.248631	-2.309624	3.922467
H	-3.787517	-0.938175	2.911496
H	-2.173101	-0.89619	3.704137
H	-1.075942	-3.828117	-2.364784
H	1.248224	-4.521532	-2.660988
H	1.50272	-4.669304	-0.901686
H	1.218751	-3.049681	-1.634595
H	-0.619059	-6.298607	-2.376176
H	-2.057507	-5.930241	-1.371189
H	-0.486769	-6.293295	-0.58844

H	-4.124373	0.345334	3.304513
H	-4.989407	1.713896	2.522976
H	-3.24148	1.843878	2.91398

**6a**			
**Atomtype**	**X [Å]**	**Y [Å]**	**Z [Å]**
C	-0.972557	2.466978	-2.256578
C	-2.224359	2.776713	-1.651467
C	-2.229147	3.572969	-0.448578
C	-1.016534	3.993521	0.125875
C	0.257876	3.72707	-0.497064
C	0.250412	2.957402	-1.686989
Ru	-0.910991	1.710692	-0.160636
C	-2.533299	0.547724	0.260593
N	-3.134851	-0.413663	-0.52201
C	-4.257901	-0.964872	0.099601
C	-4.402867	-0.302107	1.292084
N	-3.362843	0.632414	1.355466
C	-2.807442	-0.721782	-1.904133
C	-3.269444	1.602897	2.442732
C	-5.060017	-2.065678	-0.514293
C	-5.419246	-0.466249	2.375431
C	-3.537197	2.390014	-2.281024
C	1.568339	4.253193	0.074435
C	2.241023	5.202721	-0.9426
Cl	-0.083411	1.606716	2.135277
Si	0.539585	-0.263441	-0.551209
C	-0.482614	-2.013116	-0.427018

**7a**			
**Atomtype**	**X [Å]**	**Y [Å]**	**Z [Å]**
C	0.860033	-3.734224	-0.144799
C	2.221478	-3.404685	0.285234
C	2.832253	-2.645104	-0.750177
C	1.84953	-2.461782	-1.817158
C	0.668817	-3.226508	-1.459412
Rh	1.102159	-1.420057	0.014365
C	2.409885	0.108108	0.257411
N	3.212554	0.362624	1.341875
C	4.028148	1.479957	1.131383
C	3.766106	1.917786	-0.141811
N	2.797217	1.05604	-0.659201
C	3.31144	-0.430939	2.561721
C	2.406813	1.089985	-2.057338
C	4.972903	2.001724	2.164812
C	4.330142	3.062632	-0.917625
C	2.83448	-3.879594	1.564469
C	4.254206	-2.167116	-0.79032
C	2.143008	-1.988801	-3.208712
C	-0.409241	-3.618027	-2.421573
C	-0.084954	-4.585174	0.642185
Cl	0.284008	-1.562558	2.321941
Si	-0.741976	0.07929	-0.479708

N	-0.737731	-3.039798	-1.30301	C	-0.113527	2.006227	-0.390527
C	-1.49119	-4.044466	-0.696997	N	0.431059	2.532414	0.75101
C	-1.728288	-3.632808	0.591451	C	0.877205	3.832833	0.55722
N	-1.112472	-2.395263	0.725908	C	0.606525	4.144018	-0.752095
C	-0.456907	-3.1366	-2.73669	N	0.003992	3.017556	-1.313107
C	-1.20552	-1.658023	1.981153	C	0.559404	1.890502	2.054667
C	-1.907076	-5.291511	-1.4063	C	-0.367975	3.04876	-2.729442
C	-2.485887	-4.284721	1.699779	C	1.533905	4.636059	1.630271
Cl	0.956067	-0.162123	-2.718417	C	0.858935	5.402305	-1.516574
Si	2.861506	-0.906348	0.307194	Cl	-1.01579	-0.172797	-2.6493
C	2.796507	-1.737253	2.108517	Si	-3.144875	0.1171	0.337236
C	4.217952	-1.874917	2.7194	C	-3.33771	0.989457	2.101334
C	3.642682	-2.212122	-0.981138	C	-4.766911	0.77665	2.668892
C	2.580466	-3.244262	-1.417081	C	-3.733924	-1.749219	0.403925
C	3.954073	0.724662	0.322297	C	-5.27856	-1.875334	0.364781
C	3.609084	1.610413	1.54165	C	-4.190334	1.125515	-1.023554
C	4.833896	-2.984175	-0.358936	C	-3.417877	2.387491	-1.467348
C	4.179094	-1.555815	-2.279506	C	-3.250335	-2.467169	1.685162
C	5.471037	0.41484	0.392609	C	-3.125987	-2.504046	-0.797825
C	3.684594	1.536595	-0.965664	C	-4.474857	0.302418	-2.306666
C	2.157273	-3.147124	2.026871	C	-5.565955	1.577246	-0.471781
C	1.996066	-0.916717	3.14619	C	-2.383713	0.427988	3.179577
C	1.431529	4.928818	1.44664	C	-3.069749	2.510614	1.975564
H	4.526961	-3.650312	0.463952	H	-2.162015	-2.373626	1.83537
H	5.627389	-2.312829	0.012397	H	-3.498009	-3.54649	1.601887
H	5.280891	-3.62607	-1.146614	H	-3.764621	-2.087705	2.585637
H	3.412241	-0.966301	-2.804315	H	-2.024238	-2.500666	-0.730981
H	4.515125	-2.362186	-2.964639	H	-3.414295	-2.079254	-1.774077
H	5.051045	-0.907651	-2.095191	H	-3.4592	-3.563502	-0.776368
H	2.05466	-3.719607	-0.571392	H	-5.757204	-1.375402	1.225014
H	3.065468	-4.046699	-2.012524	H	-5.545346	-2.951593	0.415789
H	1.84341	-2.756619	-2.067152	H	-5.726901	-1.470373	-0.556588
H	5.741344	-0.143535	1.304374	H	-5.125836	-0.566612	-2.118578
H	6.025197	1.376467	0.413272	H	-3.554745	-0.055101	-2.791907
H	5.8357	-0.155151	-0.477198	H	-5.006272	0.955688	-3.029948
H	2.614122	1.77941	-1.062771	H	-6.165002	0.733545	-0.087417
H	3.991215	1.001338	-1.878436	H	-6.138359	2.045625	-1.299363
H	4.245499	2.493717	-0.924279	H	-5.473827	2.332836	0.326299
H	2.526725	1.800031	1.638783	H	-4.049016	2.983027	-2.160576
H	4.126991	2.587043	1.438319	H	-2.514614	2.093895	-2.016499
H	3.964652	1.155599	2.483011	H	-3.126638	3.042681	-0.627946
H	2.052127	-3.560693	3.052014	H	-2.493576	1.039434	4.100257
H	2.771906	-3.858085	1.449224	H	-1.332594	0.441959	2.878693
H	1.153006	-3.126386	1.566371	H	-2.617336	-0.613756	3.444489
H	0.967259	-0.70778	2.839001	H	-3.835405	3.025396	1.369946
H	2.462619	0.056887	3.359791	H	-2.084115	2.72454	1.519901
H	1.965472	-1.488261	4.098225	H	-3.084802	2.972011	2.985452

H	4.128888	-2.409027	3.688463	H	-4.841588	1.311887	3.638427
H	4.652259	-0.883726	2.937501	H	-4.954241	-0.292242	2.87143
H	4.929535	-2.435292	2.096146	H	-5.57105	1.151044	2.01929
H	-1.412937	-3.265058	-3.276017	H	1.56627	2.097473	2.448284
H	0.184249	-4.01282	-2.928897	H	0.44066	0.801848	1.966075
H	0.041872	-2.22553	-3.083948	H	-0.199316	2.294904	2.74567
H	-2.455724	-5.951644	-0.713951	H	0.881853	4.74109	2.518414
H	-1.031366	-5.84841	-1.791945	H	1.771073	5.645351	1.254936
H	-2.565181	-5.075912	-2.271118	H	2.477286	4.158832	1.961827
H	-3.34152	-3.658781	2.021088	H	1.331143	6.15089	-0.858867
H	-1.844869	-4.449819	2.587139	H	-0.080888	5.834871	-1.910974
H	-2.876577	-5.261203	1.368553	H	1.529767	5.233157	-2.381978
H	-2.265478	-1.616238	2.279279	H	-1.191506	3.768122	-2.877142
H	-0.834605	-0.630695	1.860618	H	-0.68017	2.053556	-3.061534
H	-0.62155	-2.176838	2.759554	H	0.506474	3.376719	-3.31862
H	-2.736471	-1.810612	-2.040954	H	2.875312	0.112663	3.41954
H	-1.856693	-0.235738	-2.162671	H	4.376972	-0.635814	2.768552
H	-3.605544	-0.344245	-2.567274	H	2.756977	-1.365875	2.42697
H	-5.874163	-2.367555	0.166341	H	4.453507	2.239351	3.114057
H	-4.43029	-2.9553	-0.716946	H	5.463691	2.919908	1.799415
H	-5.517153	-1.758085	-1.475612	H	5.765615	1.265767	2.405178
H	-6.040639	0.442924	2.498714	H	3.536542	3.777475	-1.213414
H	-4.943969	-0.671324	3.354899	H	4.834903	2.724481	-1.843852
H	-6.093562	-1.305966	2.135288	H	5.070829	3.605885	-0.306624
H	-4.279479	1.996857	2.653514	H	2.166492	2.121914	-2.355337
H	-2.599757	2.415143	2.13392	H	1.538165	0.436636	-2.199967
H	-2.85667	1.139296	3.356065	H	3.238669	0.727731	-2.688708
H	-3.179702	3.793896	0.048239	H	2.168767	-3.653495	2.418226
H	-1.037154	4.507426	1.089929	H	3.818516	-3.412973	1.743035
H	1.192943	2.708985	-2.181012	H	2.97856	-4.97851	1.536019
H	-0.94004	1.865315	-3.169039	H	4.647753	-1.986844	0.224733
H	-4.201698	1.890215	-1.553102	H	4.343161	-1.22976	-1.365569
H	-3.391608	1.733499	-3.15372	H	4.900234	-2.92482	-1.277354
H	-4.053063	3.306188	-2.62898	H	1.310037	-1.390253	-3.615937
H	2.225373	3.37992	0.202516	H	2.27667	-2.865549	-3.87583
H	0.823197	5.852505	1.381124	H	3.070994	-1.395279	-3.253742
H	2.433027	5.214359	1.816544	H	-1.207764	-4.193076	-1.927724
H	0.971149	4.246829	2.182707	H	0.042094	-4.26343	-3.203393
H	2.419815	4.70303	-1.912646	H	-0.859527	-2.751876	-2.930878
H	3.21616	5.545882	-0.549083	H	-0.121809	-4.246317	1.692804
H	1.609924	6.094615	-1.123863	H	0.25207	-5.641828	0.633567
				H	-1.105565	-4.53977	0.228551

8			
**Atomtype**	**X [Å]**	**Y [Å]**	**Z [Å]**
Ru	-1.378445	-0.617534	-0.422016
Cl	0.708306	2.478791	-1.400219
Cl	1.379434	-0.393733	-2.999151
Si	0.718565	0.312408	-1.022475
Si	2.719084	0.071859	0.311719
N	-2.465042	2.256172	-0.548842
N	-2.246904	1.618166	1.503279
C	2.091463	0.451139	2.139304
C	1.150348	-0.685003	2.591447
C	1.259394	1.757622	2.163163
C	3.239907	0.587318	3.165845
C	4.171343	1.303317	-0.194625
C	5.521346	0.866208	0.427866
C	4.318513	1.356179	-1.735732
C	3.905579	2.75104	0.287006
C	3.316039	-1.807463	0.143746
C	4.179329	-2.255594	1.347448
C	4.140612	-2.026928	-1.149948
C	2.098513	-2.756033	0.03539
C	-2.035952	1.199646	0.211299
C	-2.894135	3.32379	0.243273
C	-2.757627	2.919526	1.548595
C	-2.498988	2.260924	-2.002265
C	-3.358882	4.619276	-0.336468
C	-3.033734	3.63338	2.831248
C	-2.045067	0.783186	2.674056
C	-2.377601	-2.552105	0.401584
C	-1.273162	-2.889196	-0.452668
C	-1.195246	-2.391237	-1.784334
C	-2.210183	-1.527514	-2.325764
C	-3.294446	-1.179772	-1.462025
C	-3.387423	-1.699954	-0.131274
C	-2.553779	-3.135272	1.795128
C	-1.238897	-3.530435	2.484407
C	-3.517026	-4.3434	1.725902
C	-2.153441	-1.055483	-3.755163
H	1.681296	-1.642324	2.7306
H	0.342089	-0.842093	1.846029
H	0.677569	-0.424298	3.562853
H	0.378215	1.696143	1.501166
H	1.843035	2.640616	1.859116
H	0.895122	1.933137	3.19961
H	2.812422	0.750849	4.179369
H	3.891295	1.450343	2.939309

9			
**Atomtype**	**X [Å]**	**Y [Å]**	**Z [Å]**
C	-1.346717	-3.077038	0.127556
C	-2.242386	-2.589419	-0.898104
C	-3.251911	-1.787968	-0.252676
C	-2.984403	-1.779699	1.167312
C	-1.822275	-2.592682	1.400758
Rh	-1.237722	-0.762141	0.059496
C	-1.921501	1.121932	0.022463
N	-2.125272	1.962017	1.090815
C	-2.733288	3.158235	0.700198
C	-2.944812	3.057722	-0.653043
N	-2.454012	1.807563	-1.038465
C	-1.81081	1.635535	2.470718
C	-2.581132	1.27832	-2.388154
C	-3.021187	4.266372	1.659522
C	-3.544026	4.022015	-1.623805
C	-2.173832	-2.956027	-2.348933
C	-4.436983	-1.137388	-0.903424
C	-3.844126	-1.116775	2.205038
C	-1.216678	-2.904419	2.736443
C	-0.296228	-4.129595	-0.083034
Si	0.742172	-0.241378	-1.039941
Si	2.728065	0.335607	0.216239
C	2.597095	2.247962	0.701729
C	3.065497	3.18844	-0.435134
Cl	0.670313	1.39462	-2.500308
Cl	1.374577	-1.856127	-2.378734
C	4.390399	-0.015991	-0.778177
C	5.607882	0.659726	-0.097688
C	2.611737	-0.802047	1.820564
C	2.270548	-2.248312	1.389363
C	4.268872	0.520193	-2.226736
C	4.697729	-1.530443	-0.867839
C	3.916243	-0.825315	2.649668
C	1.450356	-0.347698	2.73405
C	3.445004	2.570441	1.956508
C	1.122703	2.605442	1.000253
H	3.454119	0.019412	-2.778239
H	5.220765	0.320488	-2.764811
H	4.084691	1.606857	-2.259032
H	3.864918	-2.095637	-1.319782
H	4.923673	-1.964939	0.12246
H	5.595727	-1.676155	-1.505644
H	5.753425	0.308029	0.939777
H	5.52667	1.760235	-0.076589

H	3.873627	-0.315712	3.2103
H	5.47784	0.834261	1.531888
H	6.303413	1.60342	0.144966
H	5.859239	-0.121997	0.072678
H	4.541764	0.370152	-2.175929
H	5.154435	2.041857	-1.994261
H	3.400181	1.741029	-2.212581
H	2.945628	3.143346	-0.087479
H	4.716965	3.405891	-0.097053
H	3.916232	2.832186	1.388991
H	3.610468	-2.249809	2.294696
H	5.071815	-1.618704	1.480428
H	4.529277	-3.29703	1.1785
H	4.390426	-3.106637	-1.235071
H	5.092508	-1.469427	-1.152213
H	3.567079	-1.738256	-2.047865
H	2.458858	-3.804872	-0.04312
H	1.508571	-2.536513	-0.871212
H	1.422811	-2.695495	0.903313
H	-1.970815	1.358786	-2.348754
H	-1.984439	3.154861	-2.393009
H	-3.547215	2.247007	-2.361462
H	-4.222628	4.47905	-1.017188
H	-2.554986	5.109114	-0.922134
H	-3.665973	5.309265	0.468785
H	-3.780957	3.097285	3.451135
H	-3.424736	4.644921	2.624974
H	-2.11525	3.739686	3.443608
H	-3.00106	0.644321	3.215205
H	-1.302394	1.234533	3.355101
H	-1.670595	-0.193675	2.327041
H	-0.462945	-3.514942	-0.073297
H	-0.31518	-2.614042	-2.395093
H	-4.059896	-0.483419	-1.823236
H	-4.212773	-1.38142	0.516459
H	-3.042174	-2.350531	2.408579
H	-1.438824	-3.860747	3.521056
H	-0.528838	-2.687561	2.512641
H	-0.75081	-4.371959	1.956646
H	-3.076924	-5.146491	1.102864
H	-4.487066	-4.057191	1.277749
H	-3.702737	-4.753163	2.738126
H	-1.114789	-0.819659	-4.045281
H	-2.780556	-0.156992	-3.900946
H	-2.531851	-1.844689	-4.437688

H	6.526713	0.40766	-0.669876
H	3.770485	-1.46414	3.547696
H	4.211054	0.181394	2.994389
H	4.758568	-1.249951	2.075216
H	0.497163	-0.329998	2.166462
H	1.617296	0.648958	3.178605
H	1.331791	-1.071162	3.568696
H	2.201344	-2.894134	2.291939
H	3.018871	-2.692321	0.714191
H	1.290704	-2.279279	0.882374
H	3.088509	2.035928	2.854745
H	3.37971	3.659242	2.170851
H	4.511141	2.320372	1.811501
H	0.493742	2.509081	0.098788
H	1.058825	3.660327	1.347752
H	0.697079	1.963194	1.784234
H	2.512726	3.008004	-1.372544
H	4.145123	3.094422	-0.642857
H	2.881652	4.239858	-0.12451
H	-2.73447	1.614086	3.078104
H	-1.113106	2.37771	2.897718
H	-1.343052	0.638899	2.480956
H	-3.503012	5.108446	1.133019
H	-2.092186	4.644238	2.132665
H	-3.696452	3.940309	2.476307
H	-4.426247	3.591034	-2.138634
H	-2.815915	4.310638	-2.408062
H	-3.866613	4.938765	-1.100017
H	-2.070853	0.302808	-2.411198
H	-2.103334	1.957415	-3.113754
H	-3.65059	1.152646	-2.64068
H	-2.567524	-3.981266	-2.518534
H	-1.1297	-2.93055	-2.706782
H	-2.770002	-2.262169	-2.969147
H	-5.362967	-1.718648	-0.709839
H	-4.307163	-1.068266	-1.996689
H	-4.599884	-0.117135	-0.508863
H	-4.223662	-0.141955	1.846029
H	-3.281852	-0.942834	3.139788
H	-4.727476	-1.7402	2.460251
H	-1.625299	-3.850313	3.151777
H	-1.42501	-2.102681	3.468661
H	-0.122037	-3.016448	2.656908
H	-0.776338	-5.107171	-0.29985
H	0.331478	-4.257124	0.815016
H	0.362209	-3.879392	-0.931543

10				1a			
**Atomtype**	**X [Å]**	**Y [Å]**	**Z [Å]**	**Atomtype**	**X [Å]**	**Y [Å]**	**Z [Å]**
Ru	-1.350501	0.747659	-0.509013	C	-1.504431	-0.031915	-0.900279
Cl	0.232663	-2.572259	0.49512	C	-1.057569	-0.485136	0.386825
Si	0.485551	-0.475973	-0.19417	C	-2.022913	-1.081223	1.259295
Si	2.893305	-0.327636	-0.041655	C	-3.374515	-1.155715	0.864219
N	-2.762042	-1.34174	1.239892	C	-3.824327	-0.662792	-0.368293
N	-2.847796	-1.91213	-0.841912	C	-2.865597	-0.114244	-1.23905
C	3.417684	-0.805977	1.806197	Si	0.710473	-0.160711	1.115197
C	2.344743	-0.290774	2.799684	C	1.003475	1.669928	0.54725
C	3.518859	-2.337029	2.012328	N	1.866602	2.321489	-0.295085
C	4.788339	-0.196546	2.187933	C	1.687802	3.705921	-0.218473
C	3.77611	-1.498679	-1.355579	C	0.681179	3.924472	0.693424
C	3.160665	-2.919042	-1.352406	N	0.286871	2.669219	1.153902
C	5.293179	-1.622499	-1.072977	C	2.719323	1.716344	-1.310691
C	3.599755	-0.937245	-2.78577	C	2.477974	4.666864	-1.044613
C	3.303627	1.567377	-0.411462	C	0.061212	5.202903	1.155372
C	4.805083	1.854497	-0.640333	C	-0.794403	2.431444	2.108784
C	2.526232	2.037353	-1.666278	C	-1.662607	-1.695999	2.614332
C	2.80436	2.43986	0.76467	C	-1.855783	-3.226879	2.590138
C	-2.354738	-0.940617	-0.008047	C	-5.291537	-0.737588	-0.765476
C	-3.462398	-2.548874	1.191232	C	-5.879707	0.669769	-1.001052
C	-3.518328	-2.911124	-0.132155	C	-0.555877	0.510421	-1.971321
C	-2.37665	-0.676932	2.471709	C	-0.77176	2.018805	-2.214316
C	-3.986466	-3.228217	2.413632	C	2.047305	-1.184476	0.178399
C	-4.124769	-4.104194	-0.795438	C	-2.444511	-1.05169	3.776493
C	-2.592799	-1.962897	-2.270581	C	-5.491193	-1.63519	-2.005468
C	-1.873012	2.65983	0.551917	C	-0.670929	-0.263234	-3.303394
C	-3.091923	2.116985	0.041907	H	2.423814	2.092174	-2.3065
C	-3.244041	1.777903	-1.341384	H	2.595519	0.625791	-1.288085
C	-2.186605	1.973594	-2.27396	H	3.779121	1.976175	-1.135336
C	-0.976447	2.571972	-1.773492	H	2.197867	5.70291	-0.791433
C	-0.822201	2.920538	-0.401327	H	2.294849	4.519535	-2.127621
C	-1.776999	3.06866	2.011321	H	3.566596	4.554595	-0.873396
C	-0.391383	2.816122	2.624693	H	0.196296	5.355609	2.244322
C	-2.193899	4.5479	2.173193	H	-1.025807	5.224646	0.944649
C	-2.346327	1.64785	-3.737735	H	0.526856	6.055331	0.633244
H	2.659196	-0.530072	3.838683	H	-0.471168	1.65558	2.829576
H	1.371599	-0.786228	2.620338	H	-1.695534	2.06574	1.584059
H	2.192396	0.800165	2.737089	H	-1.019933	3.365846	2.64542
H	3.738995	-2.538152	3.083155	H	-4.10429	-1.616485	1.542878
H	4.335175	-2.789099	1.421643	H	-3.191667	0.247316	-2.22218
H	2.576386	-2.848213	1.754214	H	0.472409	0.35959	-1.605962
H	4.779368	0.907217	2.185036	H	-0.034095	2.411079	-2.942156
H	5.593185	-0.533442	1.509098	H	-1.780639	2.198315	-2.632007
H	5.058119	-0.521763	3.216069	H	-0.687216	2.601987	-1.281023

H	3.228587	-3.411606	-0.368748
H	3.706746	-3.546656	-2.089908
H	2.094286	-2.902178	-1.640887
H	5.805339	-0.644335	-1.094186
H	5.759879	-2.26428	-1.851329
H	5.495907	-2.090463	-0.094049
H	2.534711	-0.749633	-3.02554
H	3.988821	-1.675261	-3.519669
H	4.162769	0.001353	-2.934263
H	4.953071	2.946889	-0.784289
H	5.180876	1.350577	-1.549405
H	5.432166	1.539074	0.212107
H	2.61945	3.140813	-1.766489
H	1.4552	1.79005	-1.58453
H	2.906469	1.587899	-2.597742
H	3.376398	2.272809	1.693773
H	1.739428	2.251308	0.983725
H	2.913287	3.513864	0.498667
H	-1.720431	0.165588	2.194411
H	-1.819181	-1.377728	3.1209
H	-3.266369	-0.306547	3.017224
H	-3.165823	-3.513927	3.102688
H	-4.530385	-4.147522	2.134422
H	-4.681159	-2.57714	2.98218
H	-4.620345	-4.745815	-0.045931
H	-3.357791	-4.714617	-1.313865
H	-4.88114	-3.815384	-1.553482
H	-3.5437	-1.983285	-2.837359
H	-2.003442	-2.866032	-2.519176
H	-2.006918	-1.063781	-2.530506
H	-3.896834	1.876213	0.746007
H	-4.160817	1.275185	-1.671316
H	-0.147641	2.73949	-2.468438
H	0.131945	3.332881	-0.061955
H	-2.51287	2.44656	2.559494
H	0.361759	3.503686	2.196549
H	-0.058582	1.78256	2.419527
H	-0.414645	2.987286	3.717822
H	-2.178422	4.847242	3.239614
H	-3.210715	4.722599	1.773224
H	-1.492748	5.204209	1.620934
H	-2.740608	2.520106	-4.301394
H	-3.052392	0.809106	-3.8824
H	-1.374784	1.368665	-4.185481

H	0.095999	0.090747	-4.019774
H	-0.533768	-1.349647	-3.153328
H	-1.660666	-0.111949	-3.773309
H	-0.587145	-1.50223	2.800668
H	-1.545047	-3.672146	3.554958
H	-2.916835	-3.491536	2.417275
H	-1.256153	-3.690653	1.782826
H	-2.121988	-1.482277	4.743697
H	-2.278841	0.042265	3.811385
H	-3.532852	-1.226466	3.675162
H	-5.838721	-1.198859	0.080376
H	-6.566536	-1.718495	-2.25359
H	-4.971938	-1.208745	-2.885909
H	-5.092244	-2.652446	-1.831501
H	-6.958186	0.603189	-1.240305
H	-5.75812	1.309181	-0.106141
H	-5.37421	1.168488	-1.851108
N	3.388323	-1.08919	0.47957
C	4.103344	-2.15262	-0.073801
C	3.183036	-2.946018	-0.721132
N	1.938125	-2.334193	-0.560455
C	0.701835	-2.924428	-1.067534
C	3.360969	-4.221306	-1.478725
C	5.581876	-2.311997	0.072243
C	3.951099	-0.068194	1.354299
H	0.831013	-3.194457	-2.129851
H	0.455862	-3.836668	-0.492414
H	-0.117763	-2.20164	-0.961037
H	4.906155	-0.427494	1.768974
H	4.125567	0.879586	0.814636
H	3.232555	0.119351	2.178989
H	5.921869	-3.193468	-0.496658
H	6.128285	-1.426595	-0.308267
H	5.877074	-2.458486	1.13037
H	3.034103	-4.119862	-2.532538
H	4.423127	-4.517863	-1.477067
H	2.773737	-5.045233	-1.027981

[RuCl2(p-cym)]2			
Atomtype	X [Å]	Y [Å]	Z [Å]
C	-3.95386	1.008606	0.437922
C	-3.757442	-0.396076	0.283618
C	-2.978141	-0.918723	-0.816773
C	-2.404344	0.007029	-1.738366
C	-2.576191	1.415732	-1.545043
C	-3.365397	1.95655	-0.473496
Ru	-1.847073	0.623022	0.309309
Cl	0.372139	1.596753	-0.32971
C	-2.698745	-2.401162	-1.002163
C	-3.064496	-3.271126	0.208802
C	-3.459396	3.43708	-0.217207
Cl	-0.398155	-1.19326	1.231234
C	-3.433683	-2.892894	-2.271027
H	-1.688835	-0.356569	-2.482162
H	-1.991761	2.107644	-2.162801
H	-4.437131	1.381681	1.347651
H	-4.121329	-1.070537	1.062953
H	-4.43911	3.834174	-0.552224
H	-2.659186	3.974641	-0.756542
H	-3.342485	3.633215	0.864049
H	-1.610269	-2.480963	-1.182089
H	-2.755133	-4.315931	0.01976
H	-4.158572	-3.272701	0.386495
H	-2.55487	-2.920772	1.123363
H	-3.199094	-3.959274	-2.45161
H	-3.128536	-2.315831	-3.16363
H	-4.531488	-2.796359	-2.153726
Cl	-1.599976	1.728721	2.41804
Ru	1.699591	-0.464101	0.107117
Cl	0.835508	-1.37033	-1.955798
C	3.566428	0.60508	-0.144773
C	3.104516	0.773959	1.217796
C	2.755228	-0.337825	2.025197
C	2.850407	-1.682701	1.500931
C	3.345892	-1.861642	0.171608
C	3.700434	-0.727498	-0.640813
H	2.875315	1.784527	1.573876
H	2.264444	-0.171211	2.989921
C	2.366554	-2.854274	2.31267
H	3.326221	-2.859908	-0.278227
H	3.92748	-0.889492	-1.698067
C	3.805145	1.828691	-1.011399
H	2.059525	-3.686013	1.654601
H	3.180423	-3.209978	2.976764

TS1 (+5.6 kcal/mol)			
Atomtype	X [Å]	Y [Å]	Z [Å]
C	3.489871	-1.455813	2.114251
C	4.71448	-0.745743	1.812731
C	5.489175	-1.210014	0.712307
C	5.060524	-2.341439	-0.067362
C	3.82341	-3.006228	0.209665
C	3.047605	-2.553044	1.336204
Ru	3.484053	-0.866153	-0.01259
Cl	1.699628	-1.200206	-1.605786
C	5.075602	0.495143	2.585916
C	3.299712	-4.124182	-0.673537
C	3.819699	-4.075092	-2.116765
Cl	2.103025	1.13577	0.693435
Ru	0.288226	2.21721	-0.678002
Cl	-0.403179	3.373411	1.410794
Si	-1.547972	0.231401	0.227908
C	-2.973711	1.390005	1.030131
N	-3.815771	2.008024	0.152475
C	-4.713322	2.845404	0.81125
C	-4.423261	2.738636	2.149126
N	-3.37101	1.830852	2.255444
C	-3.836631	1.779207	-1.285497
C	-5.750871	3.656738	0.10672
C	-5.039712	3.416301	3.3282
C	-2.777464	1.50116	3.540297
C	-0.718858	-0.975212	1.614408
N	-0.223449	-0.728119	2.868069
C	0.06837	-1.914175	3.533983
C	-0.213708	-2.934159	2.65936
N	-0.652879	-2.338732	1.48069
C	0.187091	0.551581	3.44348
C	-0.865143	-3.151008	0.275462
C	-0.123942	-4.414123	2.846397
C	0.602066	-1.950897	4.927957
C	-2.879467	-1.156176	-0.412361
C	-2.934204	-1.649488	-1.75496
C	-3.662815	-2.814928	-2.063349
C	-4.449916	-3.485993	-1.120715
C	-4.491588	-2.938944	0.167534
C	-3.718878	-1.822555	0.548855
C	-2.274304	-0.96336	-2.940486
C	-3.361424	-0.545068	-3.96418
C	-5.227386	-4.742086	-1.483842
C	-6.230091	-4.480566	-2.627358
C	-3.879244	-1.414801	2.015602

H	1.505002	-2.563425	2.938569
C	3.480101	1.591937	-2.495026
H	3.114004	2.607209	-0.635543
C	5.253336	2.325371	-0.807581
H	3.518771	2.551732	-3.043023
H	4.217401	0.911344	-2.963938
H	2.473962	1.149304	-2.606718
H	5.423933	3.25439	-1.384394
H	5.463881	2.534652	0.258846
H	5.97683	1.563222	-1.158203

C	-3.713479	-2.57694	3.018026
C	0.445322	4.319509	-1.326219
C	1.772739	3.813937	-1.306057
C	1.993709	2.568109	-1.988988
C	0.994713	1.956869	-2.798905
C	-0.285839	2.568925	-2.928771
C	-0.588771	3.685096	-2.089319
C	2.913637	4.414793	-0.502335
C	2.494515	5.601841	0.375911
C	-1.217363	2.166777	-4.032284
Cl	4.577117	0.294664	-1.849724
C	4.074486	4.789518	-1.451961
C	3.603076	-5.481067	0.001933
C	-5.236745	-0.712222	2.232015
C	-4.266376	-5.895763	-1.844
C	-1.152621	-1.779683	-3.652543
H	2.067498	-2.99401	1.506216
H	2.820346	-1.049852	2.879546
H	6.351136	-0.614365	0.396235
H	5.622598	-2.579561	-0.973808
H	5.533168	0.227724	3.55988
H	4.173286	1.104751	2.775529
H	5.795876	1.111257	2.01934
H	2.201599	-3.983018	-0.710055
H	3.299288	-4.83778	-2.725155
H	4.903415	-4.298001	-2.159959
H	3.637785	-3.079122	-2.556902
H	3.160545	-6.307092	-0.5869
H	3.193931	-5.528038	1.03061
H	4.695708	-5.649611	0.062318
H	2.950302	2.045807	-1.873481
H	1.22904	1.017245	-3.306424
H	-1.599128	4.107834	-2.092995
H	0.177835	5.162737	-0.6854
H	-2.279548	2.178503	-3.745827
H	-1.091933	2.914636	-4.841693
H	-0.958916	1.184479	-4.447622
H	3.273265	3.601722	0.158148
H	3.362373	5.957754	0.961346
H	2.139563	6.449688	-0.243486
H	1.690469	5.314947	1.077589
H	4.926107	5.18353	-0.865746
H	4.428419	3.910059	-2.021159
H	3.759053	5.572023	-2.169856
H	-5.133519	-3.423449	0.913975
H	-3.633776	-3.191664	-3.092717
H	1.285252	0.548223	3.544296

H	-0.088137	1.382159	2.776596
H	-0.271914	0.673333	4.439159
H	-0.11122	-3.95458	0.280376
H	-1.87993	-3.581047	0.259418
H	-0.68365	-2.534338	-0.60995
H	0.171008	-4.641074	3.884368
H	-1.096534	-4.90238	2.644984
H	0.622244	-4.878083	2.171911
H	-0.088811	-1.462419	5.642414
H	0.748602	-2.995694	5.248504
H	1.575286	-1.428254	5.004339
H	-3.567722	1.482176	4.308194
H	-2.321235	0.50597	3.489795
H	-2.010561	2.250355	3.798893
H	-4.283399	2.651807	-1.786953
H	-2.802698	1.652214	-1.638991
H	-4.413798	0.868285	-1.518366
H	-6.3494	4.218078	0.843445
H	-5.294593	4.389718	-0.587851
H	-6.440894	3.019703	-0.480331
H	-4.2888	3.995402	3.899931
H	-5.825067	4.113395	2.991527
H	-5.506527	2.689523	4.022482
H	-3.089409	-0.699774	2.257164
H	-5.320754	-0.32782	3.267502
H	-5.372542	0.13179	1.532557
H	-6.065567	-1.426537	2.067264
H	-3.696628	-2.18122	4.0523
H	-4.551238	-3.296003	2.956602
H	-2.774803	-3.12949	2.845041
H	-1.772129	-0.058853	-2.554615
H	-0.65646	-1.028731	-4.312364
H	-0.47024	-2.073777	-2.84046
H	-1.542739	-2.644096	-4.220318
H	-2.913146	-0.081256	-4.862053
H	-3.917955	-1.437563	-4.307733
H	-4.102561	0.158889	-3.540435
H	-5.803977	-5.045685	-0.587103
H	-6.816998	-5.393626	-2.84297
H	-6.932292	-3.666807	-2.364919
H	-5.700914	-4.190953	-3.555939
H	-4.833552	-6.8198	-2.066581
H	-3.667226	-5.639366	-2.739421
H	-3.566024	-6.107361	-1.013035

TS2 (–7.1 $^{kcal}/_{mol}$)			
Atomtype	X [Å]	Y [Å]	Z [Å]
N	-0.039434	-0.482025	2.823525
C	-0.514768	-0.77057	1.570901
N	-0.444191	-2.137465	1.482564
C	0.001075	-2.693033	2.678622
C	0.258135	-1.645413	3.527441
Si	-1.32831	0.398319	0.139058
Ru	0.279008	2.192422	-0.689377
Cl	2.083694	1.143568	0.708046
Ru	3.470787	-0.844833	-0.006428
Cl	4.515872	0.412916	-1.786581
C	-2.650486	-0.999608	-0.474943
C	-2.713039	-1.523516	-1.80388
C	-3.511381	-2.646985	-2.098589
C	-4.331726	-3.268006	-1.151854
C	-4.334492	-2.711614	0.131672
C	-3.514874	-1.624292	0.496281
C	-2.789178	1.556624	0.878711
N	-3.627881	2.125894	-0.03451
C	-4.545868	2.976794	0.579791
C	-4.272465	2.92801	1.923685
N	-3.207611	2.040268	2.080044
Cl	1.62406	-1.29863	-1.512869
C	0.375764	4.286377	-1.372816
C	1.722505	3.833012	-1.318545
C	2.001621	2.592211	-1.976867
C	1.045754	1.926478	-2.795564
C	-0.251608	2.488434	-2.968891
C	-0.61747	3.602459	-2.148705
C	2.822677	4.489365	-0.500953
C	4.004597	4.863529	-1.424625
C	-1.131095	2.032721	-4.090355
C	4.563358	-0.744092	1.910843
C	5.459452	-1.106567	0.866871
C	5.157561	-2.191063	-0.031608
C	3.959297	-2.955439	0.107861
C	3.070714	-2.619659	1.196746
C	3.354943	-1.52903	2.056509
C	4.806189	0.457295	2.783626
C	3.567669	-4.057131	-0.857461
C	3.948402	-5.421871	-0.241052
C	4.154232	-3.877877	-2.265318
C	2.348026	5.69571	0.32089
Cl	-0.428481	3.388396	1.372257
H	2.103509	-3.121722	1.258976

TS3 (+0.3 $^{kcal}/_{mol}$)			
Atomtype	X [Å]	Y [Å]	Z [Å]
N	3.345115	-1.496851	2.630194
C	3.851393	-1.437617	1.359554
N	4.390075	-2.708266	1.186634
C	4.221574	-3.50042	2.309401
C	3.559785	-2.736	3.235905
Si	4.153762	0.200035	0.199852
C	4.846442	-0.775372	-1.4324
N	6.097164	-0.786749	-2.002109
C	6.088218	-1.502818	-3.193342
C	4.810974	-1.992893	-3.356153
N	4.074947	-1.530178	-2.269056
C	2.680293	-1.874182	-2.005343
C	2.692712	-0.37834	3.329173
Ru	1.846154	1.937127	-0.323905
Cl	2.894407	2.36407	1.903641
C	0.583265	2.317888	-2.102957
C	1.843918	1.869218	-2.568287
C	3.000998	2.580884	-2.104134
C	2.860959	3.683294	-1.218245
C	1.581215	4.104235	-0.706947
C	0.444907	3.369045	-1.153285
C	1.921892	0.840052	-3.658512
C	1.477684	5.281842	0.248593
C	0.023523	5.515019	0.718791
Cl	0.019749	1.303427	1.272927
C	2.033021	6.578828	-0.368245
C	5.957654	0.978388	0.805721
C	7.063988	0.214108	1.379238
C	8.339162	0.77257	1.624308
C	8.643962	2.08742	1.290638
C	7.62862	2.859613	0.745538
C	6.331019	2.349421	0.547192
Cl	-0.410056	-0.854158	-1.121601
Ru	-2.02125	-2.317063	0.186688
C	-2.691752	-3.933505	1.58747
C	-1.837343	-4.490104	0.584187
C	-0.504592	-4.030299	0.407145
C	-0.14107	-2.8397	1.121762
C	-0.953759	-2.299615	2.163816
C	-2.193452	-2.932849	2.477504
C	0.507019	-4.685182	-0.505553
C	1.540281	-5.430667	0.364851
C	-2.863148	-2.6789	3.794467
Cl	-2.933989	-3.233026	-1.921287

H	2.604608	-1.207284	2.782041
H	6.317245	-0.46215	0.64966
H	5.793431	-2.335705	-0.908209
H	5.245568	0.144499	3.752619
H	3.85917	0.991063	2.980993
H	5.502343	1.161382	2.295677
H	2.466279	-3.998467	-0.946646
H	3.715839	-4.627229	-2.949747
H	5.250835	-4.029578	-2.265903
H	3.929118	-2.867573	-2.650209
H	3.625889	-6.244541	-0.906879
H	3.476322	-5.569604	0.750132
H	5.045302	-5.49702	-0.109881
H	2.972494	2.104356	-1.835667
H	1.323756	0.986026	-3.280104
H	-1.639651	3.994052	-2.180154
H	0.06583	5.131561	-0.753968
H	-2.204125	2.046767	-3.848765
H	-0.975947	2.748204	-4.923853
H	-0.839299	1.036605	-4.451232
H	3.180869	3.707341	0.197319
H	3.188837	6.095687	0.917203
H	1.989494	6.509653	-0.340312
H	1.531834	5.413191	1.009364
H	4.824992	5.301336	-0.825143
H	4.400824	3.975451	-1.950771
H	3.69203	5.612067	-2.178978
C	-2.631728	1.768649	3.386199
C	-4.920946	3.630604	3.069867
C	-5.593217	3.733871	-0.168988
C	-3.629866	1.849984	-1.465852
C	0.355919	0.815527	3.371536
C	0.781949	-1.636078	4.925618
C	0.11029	-4.164541	2.911841
C	-0.675032	-2.999817	0.315287
C	-3.665156	-1.205901	1.962966
H	-4.994226	-3.159177	0.885613
C	-5.169995	-4.48845	-1.500809
H	-3.498793	-3.044488	-3.119426
C	-1.974571	-0.936285	-3.005275
H	1.454003	0.826769	3.473708
H	0.069935	1.629467	2.689377
H	-0.105425	0.950683	4.364481
H	0.088996	-3.793675	0.330602
H	-1.686124	-3.439928	0.348888
H	-0.531326	-2.420949	-0.600686
H	0.415395	-4.355519	3.954091

Si	-3.676505	-0.425894	-0.148873
C	-4.881246	0.804838	0.869655
C	-4.739381	1.124809	2.255853
C	-5.43783	2.210911	2.817172
C	-6.37338	2.961288	2.098005
C	-6.590707	2.58219	0.768162
C	-5.854938	1.563443	0.130287
C	-3.857215	0.350298	3.233315
C	-2.703342	1.201633	3.798769
C	-7.112258	4.129854	2.730849
C	-6.130873	5.265527	3.093677
C	-6.24241	1.335183	-1.335301
C	-7.589838	0.586066	-1.41819
C	-5.203263	-1.53415	-0.821646
N	-5.902625	-2.255082	0.10354
C	-6.871226	-3.050279	-0.505025
C	-6.768178	-2.813397	-1.854969
N	-5.748468	-1.876882	-2.018704
C	-5.735893	-2.161752	1.548559
C	-7.792646	-3.950235	0.251565
C	-7.548046	-3.378993	-2.995629
C	-5.340779	-1.410306	-3.334796
C	-3.100071	0.96535	-1.478191
N	-2.721771	0.851995	-2.788915
C	-2.628534	2.096427	-3.404422
C	-2.909955	3.034843	-2.441746
N	-3.147019	2.333804	-1.266232
C	-2.232846	-0.34021	-3.475785
C	-2.208872	2.265783	-4.826322
C	-2.975174	4.523592	-2.546748
C	-3.289367	3.066871	0.000903
C	-4.725461	-0.184936	4.400926
C	-7.926587	3.686888	3.964534
C	-6.313655	2.624484	-2.184396
C	-0.110514	-5.616879	-1.560847
H	-0.537015	3.566463	-0.730156
H	-0.302085	1.735899	-2.36007
H	3.993225	2.248591	-2.419818
H	3.784638	4.211628	-0.889029
H	1.681344	1.333529	-4.621977
H	1.197174	0.027572	-3.48912
H	2.938995	0.423518	-3.747714
H	2.087939	5.013644	1.1327
H	1.978652	7.391654	0.378409
H	1.431324	6.885625	-1.246214
H	3.085794	6.470059	-0.684418
H	-0.0059	6.340001	1.453584

H	-0.857415	-4.67138	2.733118
H	0.857669	-4.638549	2.246324
H	0.070919	-1.155863	5.625894
H	0.960053	-2.66884	5.268492
H	1.737304	-1.080568	4.997205
H	-3.439505	1.727126	4.135159
H	-2.126024	0.797812	3.369662
H	-1.910043	2.561139	3.647344
H	-4.043428	2.719506	-2.000906
H	-2.595444	1.685447	-1.802865
H	-4.228922	0.948933	-1.680716
H	-6.197812	4.328979	0.535853
H	-5.146993	4.431744	-0.904936
H	-6.274795	3.055287	-0.718426
H	-4.202371	4.28038	3.606309
H	-5.749694	4.260737	2.705804
H	-5.338837	2.913051	3.803686
H	-2.864193	-0.503119	2.204072
C	-5.017072	-0.493052	2.184892
C	-3.51147	-2.36698	2.970441
H	-5.083313	-0.086372	3.213016
H	-5.167327	0.333973	1.469507
H	-5.848977	-1.209988	2.049919
H	-3.477529	-1.965939	4.002232
H	-4.361785	-3.07191	2.920561
H	-2.584507	-2.937321	2.792199
H	-1.379663	-0.075226	-2.653655
C	-1.00345	-1.944702	-3.657986
C	-3.004664	-0.483053	-4.072224
H	-0.383656	-1.439045	-4.422589
H	-0.321403	-2.39878	-2.920976
H	-1.564429	-2.753064	-4.164425
H	-2.505071	-0.125174	-4.989207
H	-3.644502	-1.333857	-4.367682
H	-3.671106	0.317768	-3.704401
C	-4.265634	-5.703781	-1.803025
H	-5.784278	-4.733319	-0.611236
C	-6.127678	-4.207991	-2.67785
H	-6.768916	-5.088421	-2.873657
H	-6.780045	-3.339858	-2.465566
H	-5.560258	-3.992178	-3.603885
H	-4.878074	-6.600923	-2.015539
H	-3.63159	-5.505443	-2.689262
H	-3.599358	-5.929208	-0.948295

H	-0.399369	4.613285	1.199532
H	-0.620424	5.800011	-0.136002
H	0.794	-2.334621	0.861486
H	-0.625586	-1.420354	2.726377
H	-3.693803	-4.34697	1.739055
H	-2.236755	-5.263175	-0.076239
H	-3.960183	-2.621763	3.737468
H	-2.621017	-3.545382	4.443171
H	-2.472022	-1.773858	4.279748
H	1.024794	-3.867519	-1.03374
H	0.67958	-6.018073	-2.223195
H	-0.608981	-6.481958	-1.082835
H	-0.857421	-5.077127	-2.170236
H	2.340415	-5.864248	-0.266852
H	1.995118	-4.753067	1.108022
H	1.054626	-6.259809	0.914424
H	-7.352151	3.126592	0.197287
H	-5.250651	2.473287	3.864362
H	-1.147833	-0.224263	-3.634178
H	-2.385654	-1.23523	-2.856308
H	-2.741142	-0.440185	-4.449426
H	-2.629808	3.947241	-0.043892
H	-4.331426	3.390524	0.152826
H	-2.948539	2.437775	0.828402
H	-2.854967	4.828313	-3.59953
H	-3.948156	4.906606	-2.184673
H	-2.184177	5.022883	-1.956314
H	-2.893196	1.743774	-5.522925
H	-2.197279	3.335807	-5.091624
H	-1.192371	1.858196	-4.992953
H	-6.229123	-1.341338	-3.982985
H	-4.897254	-0.411016	-3.251245
H	-4.606414	-2.110087	-3.766587
H	-6.076845	-3.100123	2.013135
H	-4.670827	-2.015227	1.773736
H	-6.310706	-1.309448	1.949915
H	-8.486234	-4.446202	-0.447481
H	-7.238866	-4.740766	0.795973
H	-8.397066	-3.390764	0.991792
H	-6.888139	-3.880397	-3.729702
H	-8.272994	-4.121801	-2.623594
H	-8.114799	-2.591952	-3.531653
H	-5.46845	0.723378	-1.807962
H	-7.832866	0.326096	-2.466859
H	-7.589234	-0.338264	-0.816642
H	-8.400315	1.23379	-1.034031
H	-6.432849	2.360586	-3.252985

H	-7.177046	3.256588	-1.907864
H	-5.397766	3.23035	-2.077453
H	-3.412575	-0.495892	2.682122
H	-2.049141	0.579919	4.441163
H	-2.074045	1.636825	3.005119
H	-3.097214	2.023143	4.427205
H	-4.104592	-0.664594	5.177869
H	-5.271254	0.638684	4.892936
H	-5.478171	-0.917497	4.055347
H	-7.822123	4.520908	1.974923
H	-8.493933	4.541778	4.378306
H	-8.642752	2.884563	3.705194
H	-7.257969	3.307569	4.76164
H	-6.678683	6.132441	3.509219
H	-5.403662	4.925354	3.856612
H	-5.563842	5.6049	2.205461
C	7.009616	-1.208998	1.842274
H	9.116807	0.15809	2.090018
H	9.640254	2.502688	1.487519
H	7.827889	3.906422	0.493651
C	5.354433	3.363326	0.081669
C	4.250139	-2.83067	-4.44151
C	7.274735	-1.653682	-4.06179
C	7.255375	-0.037107	-1.527979
C	3.147561	-3.115249	4.607282
C	4.692895	-4.897509	2.417021
C	5.006755	-3.248037	-0.012302
H	2.080756	-0.789144	4.144863
H	3.454392	0.310275	3.730218
H	2.045438	0.173846	2.630235
H	2.107865	-1.840947	-2.94092
H	2.595539	-2.890102	-1.585357
H	2.252398	-1.161228	-1.282377
H	5.363201	-4.263261	0.187353
H	4.272941	-3.297425	-0.819802
H	5.850311	-2.628496	-0.31725
H	4.438805	-5.299648	3.40574
H	4.219884	-5.529322	1.652066
H	5.784174	-4.95103	2.288282
H	2.638504	-2.292066	5.118641
H	2.462942	-3.97553	4.575688
H	4.027472	-3.395333	5.204324
H	8.134018	-0.32242	-2.118393
H	7.077378	1.035894	-1.656338
H	7.425585	-0.203415	-0.439116
H	7.021809	-2.263864	-4.938232
H	7.629802	-0.672026	-4.408867

H	8.09593	-2.143457	-3.517696
H	3.191781	-3.052412	-4.259774
H	4.333114	-2.313852	-5.408854
H	4.794591	-3.78339	-4.514465
H	6.048762	-1.609844	1.574921
C	8.095745	-2.08045	1.252022
C	7.093447	-1.274308	3.34754
H	7.036078	-2.318797	3.689068
H	8.042278	-0.850803	3.705433
H	6.270897	-0.707322	3.807379
H	7.993716	-3.113097	1.615594
H	8.057893	-2.101705	0.154628
H	9.092208	-1.72311	1.543297
H	4.397506	2.858046	0.031649
C	5.292118	4.516285	1.054434
C	5.72779	3.902975	-1.276346
H	4.549513	5.259258	0.736256
H	5.022228	4.16775	2.057262
H	6.262808	5.027099	1.107753
H	5.035615	4.631068	-1.705829
H	6.681095	4.444659	-1.187605
H	5.879777	3.098464	-2.009504

2’			
Atomtype	X [Å]	Y [Å]	Z [Å]
N	-0.03355	2.594325	1.672632
C	0.287966	1.265762	1.592799
N	0.704179	0.941677	2.851803
C	0.640927	2.039803	3.71062
C	0.181636	3.091794	2.953906
Si	0.033909	0.212031	-0.122141
C	1.402989	-1.1937	-0.227442
C	1.573395	-2.223206	0.757319
C	2.822196	-2.84494	0.93041
C	3.917317	-2.556955	0.094942
C	3.656301	-1.768609	-1.033685
C	2.409615	-1.14401	-1.248725
C	0.353478	-2.832818	1.441966
C	0.614188	-3.579506	2.763268
C	5.290198	-3.157794	0.351952
C	5.253915	-4.700673	0.339265
C	2.119009	-0.672703	-2.673557
C	1.940705	-1.928371	-3.562242
C	1.196628	-0.355492	3.295241
C	0.998491	1.972741	5.159943

TS4 (+3.5 kcal/mol)			
Atomtype	X [Å]	Y [Å]	Z [Å]
N	0.721755	2.297087	-2.349828
C	0.995752	1.561861	-1.236333
N	2.147504	2.107171	-0.731572
C	2.570142	3.186974	-1.502681
C	1.660686	3.307011	-2.527117
Si	-0.042581	0.13298	-0.262938
Ru	-2.42122	-0.016372	-0.312446
Cl	-1.091615	-1.003957	-2.153135
C	2.947379	1.603612	0.384962
C	3.81099	3.96434	-1.209992
C	1.624839	4.245188	-3.686974
C	-0.236754	1.989488	-3.416166
C	0.358772	1.387679	1.68632
N	0.1344	2.731715	1.755696
C	0.400094	3.224039	3.033102
C	0.805987	2.148951	3.789948
N	0.776006	1.044857	2.935944
C	-0.341125	3.58379	0.664996
C	1.15941	-0.293739	3.365546
C	1.193319	2.06547	5.230822

C	-0.103824	4.505078	3.343186
C	-0.548074	3.438481	0.594222
Ru	-2.330362	-0.016738	-0.306563
Cl	-2.617527	2.039652	-1.630843
C	-2.970491	-1.777088	0.869712
C	-4.024891	-1.589779	-0.092886
C	-4.619187	-0.306871	-0.188171
C	-4.170849	0.761045	0.650654
C	-3.202015	0.579087	1.681455
C	-2.576067	-0.709601	1.740199
C	-4.487961	-2.682338	-1.042614
C	-3.597528	-3.93081	-1.062772
C	-2.889466	1.680381	2.657199
Cl	-1.555182	-1.198122	-2.297311
C	-5.951711	-3.057607	-0.706734
C	1.077827	1.6197	-1.109481
N	2.179749	2.23774	-0.578202
C	2.573023	3.317711	-1.365145
C	1.714995	3.340466	-2.441362
N	0.826712	2.28486	-2.269098
C	2.962655	1.797565	0.577372
C	3.747057	4.182166	-1.041905
C	1.68759	4.228847	-3.640083
C	-0.043877	1.85776	-3.37031
C	-0.267352	-3.808664	0.411727
C	5.877479	-2.630665	1.679305
C	3.171934	0.275312	-3.2732
H	4.435027	-1.679498	-1.802145
H	2.940566	-3.599474	1.715561
H	1.13316	-0.193777	-2.688979
H	2.840908	0.638384	-4.265001
H	4.13529	-0.24666	-3.4261
H	3.361542	1.150384	-2.627761
H	1.660662	-1.623178	-4.589142
H	1.136668	-2.570597	-3.161194
H	2.876973	-2.516112	-3.614789
H	5.95457	-2.826591	-0.470861
H	6.897147	-3.030057	1.839766
H	5.249453	-2.947185	2.535254
H	5.931016	-1.524694	1.682533
H	6.272	-5.111118	0.479459
H	4.849415	-5.082697	-0.616701
H	4.618422	-5.086698	1.159774
H	-0.378922	-2.027902	1.61772
H	-1.128834	-4.350076	0.847985
H	0.486481	-4.561591	0.113994
H	-0.597312	-3.263362	-0.489121

C	0.224515	4.657606	3.415314
C	1.355734	-1.259564	-0.325378
C	1.448852	-2.3161	0.644138
C	2.669762	-2.969052	0.889681
C	3.832623	-2.677027	0.154355
C	3.685501	-1.819846	-0.943038
C	2.469429	-1.17041	-1.241083
C	0.198647	-2.898593	1.290765
C	-0.365143	-3.947942	0.301939
C	5.16774	-3.319843	0.496397
C	5.661941	-2.847816	1.881103
C	2.352994	-0.656813	-2.675772
C	3.515904	0.243045	-3.127453
C	0.398699	-3.547658	2.672048
C	5.098445	-4.860396	0.431683
C	2.201736	-1.882216	-3.611767
Cl	-2.655491	2.123778	-1.500911
C	-3.088417	-1.807323	0.795986
C	-4.167957	-1.528015	-0.116135
C	-4.718202	-0.223389	-0.110455
C	-4.185874	0.782334	0.757532
C	-3.180098	0.509819	1.731999
C	-2.612179	-0.806389	1.702926
C	-4.704483	-2.548172	-1.107098
C	-6.118473	-2.993982	-0.663848
C	-2.76897	1.5513	2.736482
C	-3.791023	-3.760632	-1.327207
H	4.536297	-1.694286	-1.625817
H	2.713202	-3.754979	1.652299
H	1.405288	-0.120953	-2.784646
H	3.277715	0.718426	-4.098223
H	4.440165	-0.347469	-3.274131
H	3.735156	1.039451	-2.394356
H	2.035722	-1.541542	-4.652466
H	1.336971	-2.497168	-3.305986
H	3.11211	-2.51119	-3.591401
H	5.90142	-2.980691	-0.261753
H	6.653349	-3.283409	2.110094
H	4.957644	-3.167343	2.674207
H	5.747295	-1.744602	1.921402
H	6.093133	-5.300183	0.63615
H	4.760473	-5.203329	-0.564149
H	4.39304	-5.255462	1.188263
H	-0.536236	-2.083467	1.380005
H	-1.232199	-4.477799	0.741094
H	0.411556	-4.703232	0.07764
H	-0.673008	-3.467378	-0.641485

H	-0.347799	-3.839606	3.243458	H	-0.583453	-3.781437	3.124572
H	1.218174	-2.996537	3.482047	H	0.960764	-2.90241	3.371473
H	1.150841	-4.528651	2.577597	H	0.952525	-4.501434	2.585342
H	-0.729492	2.674687	-3.640132	H	-0.864313	2.871495	-3.613897
H	-0.633792	0.981994	-3.069771	H	-0.879698	1.154431	-3.115356
H	0.607693	1.603084	-4.226384	H	0.342184	1.719063	-4.318597
H	2.438834	5.029633	-3.537318	H	2.43153	4.99167	-3.596831
H	0.692796	4.694143	-3.775428	H	0.657095	4.778611	-3.745659
H	1.913867	3.659871	-4.563064	H	1.761291	3.703501	-4.643487
H	3.808411	5.015024	-1.762137	H	3.892064	4.816721	-1.905032
H	4.698138	3.615519	-1.098347	H	4.719901	3.341389	-1.331056
H	3.674227	4.610409	-0.023493	H	3.814058	4.35991	-0.175836
H	4.032512	1.914973	0.336983	H	4.009561	1.628533	0.086676
H	2.766814	0.73243	0.763376	H	2.674184	0.561663	0.595087
H	2.72464	2.400888	1.47213	H	2.805396	2.233033	1.282443
H	-5.337895	-0.098478	-0.985934	H	-5.462435	0.050452	-0.864162
H	-4.562621	1.767645	0.472084	H	-4.538517	1.812879	0.64857
H	-1.79296	-0.880134	2.487756	H	-1.806832	-1.048465	2.40549
H	-2.463796	-2.737271	0.931989	H	-2.617969	-2.788159	0.79084
H	-3.705156	1.769333	3.402609	H	-3.543012	1.643411	3.524736
H	-2.803957	2.651573	2.138707	H	-2.66211	2.539452	2.255284
H	-1.953934	1.481649	3.205973	H	-1.818973	1.286316	3.228224
H	-4.464108	-2.22923	-2.052956	H	-4.792245	-2.01151	-2.072078
H	-3.983829	-4.648008	-1.809654	H	-4.222963	-4.413368	-2.107456
H	-3.599513	-4.439035	-0.078498	H	-3.696144	-4.36292	-0.402709
H	-2.561792	-3.670034	-1.332535	H	-2.787555	-3.440935	-1.652079
H	-6.32106	-3.807675	-1.430701	H	-6.542821	-3.694267	-1.407365
H	-6.621953	-2.179025	-0.744086	H	-6.806673	-2.134498	-0.562026
H	-6.016003	-3.497947	0.307526	H	-6.071168	-3.515344	0.312206
H	1.955708	-0.206513	4.079618	H	1.96633	-0.222742	4.112551
H	0.373983	-0.96552	3.705357	H	0.300913	-0.822708	3.815142
H	1.65721	-0.882874	2.449087	H	1.525344	-0.861073	2.497725
H	0.829201	2.954323	5.633098	H	1.101777	3.058214	5.702286
H	0.383234	1.223584	5.695327	H	0.543248	1.361499	5.786184
H	2.061609	1.700338	5.312601	H	2.239541	1.72366	5.358974
H	0.443054	5.223899	2.703424	H	0.813235	5.329705	2.761876
H	-1.183438	4.73857	3.255447	H	-0.835943	4.970515	3.341906
H	0.19806	4.675275	4.389938	H	0.554264	4.813023	4.456069
H	-1.248823	4.171318	1.024131	H	-0.991321	4.368531	1.082968
H	0.28007	3.979653	0.101549	H	0.513541	4.059104	0.149465
H	-1.103083	2.832019	-0.14227	H	-0.937875	2.991449	-0.050635

TS5 (+1.3 kcal/mol)			
Atomtype	X [Å]	Y [Å]	Z [Å]
N	0.658725	3.256249	-0.652026
C	0.639529	2.111902	0.0797
N	1.362463	2.37528	1.201769
C	1.849741	3.678698	1.180224
C	1.403344	4.236645	0.000616
Si	-0.107779	0.326141	-0.183549
Ru	-2.41879	0.07461	0.114803
Cl	-1.087494	0.338035	-2.180257
C	1.598553	1.415085	2.280281
C	2.683494	4.262086	2.273817
C	1.62997	5.606146	-0.551501
C	-0.030769	3.468174	-1.927428
C	1.501648	-0.707018	-0.209368
C	1.725657	-1.760021	0.734058
C	2.986964	-2.374291	0.811239
C	4.057182	-1.997574	-0.016067
C	3.824056	-0.978644	-0.952358
C	2.582174	-0.327387	-1.07067
C	0.644114	-2.28073	1.676841
C	0.30246	-3.751724	1.353847
C	5.407186	-2.691937	0.080376
C	6.000536	-2.590914	1.501351
C	2.480681	0.751993	-2.149462
C	3.444778	1.925157	-1.876114
C	1.02196	-2.135619	3.165949
C	5.296817	-4.165063	-0.370715
C	2.693445	0.160699	-3.559258
Cl	-2.49784	2.47304	0.334892
C	-2.642607	-2.065975	0.583358
C	-3.437629	-1.759165	-0.561027
C	-4.479235	-0.754256	-0.437453
C	-4.622508	-0.009989	0.741111
C	-3.695482	-0.187829	1.843105
C	-2.746842	-1.264442	1.764475
C	-3.283248	-2.498781	-1.878906
C	-4.460403	-3.490471	-2.037151
C	-3.767097	0.703648	3.052616
C	-1.934249	-3.21655	-2.040464
H	4.643434	-0.683826	-1.620472
H	3.138727	-3.178493	1.540817
H	1.460866	1.161916	-2.149099
H	3.288129	2.733179	-2.616897
H	4.498539	1.598139	-1.955229
H	3.294868	2.344633	-0.864031

TS6 (+2.3 kcal/mol)			
Atomtype	X [Å]	Y [Å]	Z [Å]
C	-0.91829	3.061898	0.828649
C	-0.433755	3.680058	-0.37443
C	0.931646	4.158947	-0.391796
C	1.811523	3.818695	0.649885
C	1.35692	3.082399	1.81617
C	-0.035377	2.801884	1.926176
Ru	0.726374	1.911454	0.052464
Cl	0.8474	1.381646	-2.287088
C	-1.34264	3.918807	-1.56519
C	-1.90718	5.355222	-1.481245
C	2.331781	2.680136	2.889866
Si	-0.306234	-0.18233	0.582495
Cl	-0.056946	-0.457603	2.758787
C	0.439065	-1.994071	0.222032
N	-0.003337	-3.131882	0.835955
C	0.7936	-4.220147	0.494547
C	1.748615	-3.745678	-0.376741
N	1.505224	-2.38485	-0.526734
C	-1.200072	-3.297866	1.666196
C	2.202329	-1.591581	-1.533633
C	2.842461	-4.454613	-1.104968
C	0.544739	-5.601195	1.004493
C	-2.15281	-0.506126	0.120163
C	-3.263958	-0.179664	0.958359
C	-4.551479	-0.648615	0.627492
C	-4.806541	-1.399728	-0.528378
C	-3.723921	-1.636507	-1.391177
C	-2.42049	-1.193278	-1.110965
C	-3.167946	0.682241	2.219486
C	-4.123195	1.896057	2.155891
C	-6.201077	-1.873947	-0.919603
C	-7.170415	-2.031083	0.263909
C	-1.374355	-1.429252	-2.206041
C	-1.135487	-2.927621	-2.486271
C	3.617645	0.437587	0.322398
N	4.488758	0.643408	-0.701854
C	5.484978	-0.339281	-0.751667
C	5.224937	-1.193559	0.297787
N	4.080939	-0.689117	0.930417
C	4.375085	1.73772	-1.66047
C	6.57947	-0.345947	-1.769653
C	5.953787	-2.408371	0.774846
C	3.469044	-1.308711	2.098573
C	-3.438212	-0.139595	3.497674

H	2.570287	0.948146	-4.327564
H	1.962393	-0.643891	-3.760861
H	3.710135	-0.262054	-3.664817
H	6.095856	-2.173398	-0.61564
H	7.003317	-3.057127	1.532
H	5.361026	-3.11964	2.234806
H	6.095487	-1.536528	1.823992
H	6.289968	-4.652292	-0.340525
H	4.900061	-4.239781	-1.400568
H	4.619591	-4.729037	0.3
H	-0.257966	-1.674395	1.501935
H	-0.540256	-4.103991	1.979569
H	1.169259	-4.407829	1.558042
H	0.032979	-3.876341	0.288628
H	0.192082	-2.486234	3.809793
H	1.248162	-1.087724	3.434768
H	1.912415	-2.74467	3.410721
H	0.006132	4.539406	-2.17348
H	-1.079995	3.151705	-1.82132
H	0.461536	2.8928	-2.730521
H	2.263641	6.186862	0.138987
H	0.677642	6.155795	-0.683391
H	2.139554	5.568175	-1.53384
H	2.957739	5.299792	2.022083
H	3.618271	3.688199	2.426112
H	2.136289	4.282249	3.237041
H	1.860417	1.96195	3.199281
H	2.40924	0.71703	2.006911
H	0.674496	0.840243	2.466198
H	-5.086745	-0.50827	-1.31541
H	-5.321193	0.832457	0.771699
H	-2.061334	-1.426284	2.60295
H	-1.872763	-2.833165	0.515075
H	-4.617038	0.399362	3.696069
H	-3.912173	1.753497	2.744237
H	-2.837251	0.634885	3.644572
H	-3.368131	-1.733215	-2.674945
H	-1.847367	-3.619196	-3.065624
H	-1.852754	-4.070994	-1.341328
H	-1.085741	-2.532122	-1.863404
H	-4.400542	-3.994238	-3.019756
H	-5.438958	-2.979608	-1.969106
H	-4.419634	-4.264974	-1.247268

C	-1.760636	-0.693436	-3.507439
C	-6.791395	-0.92242	-1.987881
C	-2.462398	2.873901	-1.689563
H	-3.910171	-2.173883	-2.330245
H	-5.381782	-0.40668	1.298965
H	-0.418253	-0.973865	-1.899608
H	-0.311911	-3.057855	-3.215281
H	-2.039872	-3.391056	-2.924067
H	-0.883527	-3.490917	-1.570058
H	-0.96766	-0.829757	-4.26683
H	-1.874157	0.388597	-3.324938
H	-2.708678	-1.086694	-3.921138
H	-6.076656	-2.869244	-1.394012
H	-8.111305	-2.501587	-0.076666
H	-7.433003	-1.047344	0.699061
H	-6.735685	-2.657262	1.066108
H	-7.777841	-1.287247	-2.333215
H	-6.121502	-0.838595	-2.864105
H	-6.924829	0.09107	-1.561349
H	-2.144838	1.080696	2.293325
H	-3.932759	2.576293	3.007966
H	-5.180242	1.576706	2.210851
H	-4.002745	2.465424	1.214396
H	-3.291438	0.485687	4.399375
H	-2.758241	-1.00693	3.567499
H	-4.481559	-0.509873	3.504182
H	3.288757	-1.747482	-1.43483
H	1.95978	-0.530863	-1.403861
H	1.868121	-1.915974	-2.535554
H	2.886504	-5.509893	-0.787675
H	3.827467	-3.989659	-0.907713
H	2.679005	-4.430518	-2.200244
H	1.310133	-6.291573	0.612494
H	-0.448564	-5.975025	0.687289
H	0.574863	-5.639943	2.110662
H	-1.800497	-4.123416	1.246447
H	-1.800106	-2.380465	1.653103
H	-0.908225	-3.53858	2.703012
H	4.166132	1.343807	-2.671767
H	5.312582	2.323328	-1.684922
H	3.529729	2.373555	-1.356227
H	7.192088	-1.258407	-1.666733
H	7.250774	0.528526	-1.652931
H	6.176851	-0.31456	-2.801085
H	6.773238	-2.65915	0.079135
H	5.282337	-3.286935	0.848413
H	6.396634	-2.253929	1.779395

H	4.195106	-1.359536	2.931223
H	3.132382	-2.335211	1.857816
H	2.598108	-0.709718	2.39965
H	1.317713	4.617281	-1.308512
H	2.881533	4.033942	0.550892
H	-0.397361	2.255542	2.801024
H	-1.954396	2.726287	0.877598
H	2.494536	3.522713	3.592434
H	3.298893	2.398973	2.438703
H	1.940995	1.818623	3.458152
H	-0.701546	3.840191	-2.462755
H	-2.977436	2.992169	-2.65983
H	-3.223275	2.995946	-0.894691
H	-2.052845	1.851743	-1.625144
H	-2.533327	5.567027	-2.368159
H	-1.098679	6.109428	-1.438539
H	-2.537278	5.473488	-0.578104

4. References

[1] S. U. Ahmad, T. Szilvási and S. Inoue, A facile access to a novel NHC-stabilized silyliumylidene ion and C–H activation of phenylacetylene, *Chem. Commun.* **2014**, *50*, 12619-12622.

[2] P. Frisch and S. Inoue, NHC-stabilized silyl-substituted silyliumylidene ions, *Dalton Trans.* **2019**, *48*, 10403-10406.

[3] S. B. Jensen, S. J. Rodger and M. D. Spicer, Facile preparation of η^6-*p*-cymene ruthenium diphosphine complexes. Crystal structure of [(η^6-*p*-cymene)Ru(dppf)Cl]PF_6, *J. Organomet. Chem.* **1998**, *556*, 151-158.

[4] C. White, A. Yates and P. M. Maitlis, (η^5-Pentamethylcyclopentadienyl)Rhodium and -Iridium Compounds in *Inorg. Synth.*, **1992**.

[5] D. Journal of Chemical PhysicsReiter, R. Holzner, A. Porzelt, P. J. Altmann, P. Frisch and S. Inoue, Disilene–Silylene Interconversion: A Synthetically Accessible Acyclic Bis(silyl)silylene, *J. Am. Chem. Soc.* **2019**, *141*, 13536-13546.

[6] P. Frisch, T. Szilvási, A. Porzelt and S. Inoue, Transition Metal Carbonyl Complexes of an *N*-Heterocyclic Carbene Stabilized Silyliumylidene Ion, *Inorg. Chem.* **2019**, *58*, 14931-14937.

[7] APEX suite of crystallographic software, APEX 3 version 2015.5-2; Bruker AXS Inc.: Madison, Wisconsin, USA, **2015**.

[8] SAINT, Version 7.56a and SADABS Version 2008/1; Bruker AXS Inc.: Madison, Wisconsin, USA, **2008**.

[9] G. M. Sheldrick, SHELXL-2014, University of Göttingen, Göttingen, Germany, **2014**.

[10] C. B. Hübschle, G. M. Sheldrick and B. Dittrich, ShelXle: a Qt graphical user interface for SHELXL, *J. Appl. Cryst.* **2011**, *44*, 1281-1284.

[11] G. M. Sheldrick, SHELXL-97, University of Göttingen, Göttingen, Germany, **1998**.

[12] A. J. C. Wilson, International Tables for Crystallography, Vol. C, Tables 6.1.1.4 (pp. 500-502), 4.2.6.8 (pp. 219-222), and 4.2.4.2 (pp. 193-199); Kluwer Academic Publishers: Dordrecht, The Netherlands, **1992**.

[13] C. F. Macrae, I. J. Bruno, J. A. Chisholm, P. R. Edgington, P. McCabe, E. Pidcock, L. Rodriguez-Monge, R. Taylor, J. van de Streek and P. A. Wood, Mercury CSD 2.0 - new features for the visualization and investigation of crystal structures, *J. Appl. Cryst.* **2008**, *41*, 466-470.

[14] Diamond – Crystal and Molecular Structure Visualization, Crystal Impact – Dr. H. Putz & Dr. K. Brandenburg GbR, Kreuzherrenstr. 102, 53227 Bonn, Germany, www.crystalimpact.com/diamond.

[15] A. D. Becke, Density-functional thermochemistry. V. Systematic optimization of exchange-correlation functionals, *J. Chem. Phys.* **1997**, *107*, 8554-8560.

[16] S. Grimme, Semiempirical GGA-type density functional constructed with a long-range dispersion correction, *J. Comput. Chem.* **2006**, *27*, 1787-1799.

[17] F. Weigend and R. Ahlrichs, Balanced basis sets of split valence, triple zeta valence and quadruple zeta valence quality for H to Rn: Design and assessment of accuracy, *Physical Chemistry Chemical Physics* **2005**, *7*, 3297-3305.

[18] M. J. Frisch, G. W. Trucks, H. B. Schlegel, G. E. Scuseria, M. A. Robb, J. R. Cheeseman, G. Scalmani, V. Barone, B. Mennucci, G. A. Petersson, H. Nakatsuji, M. Caricato, X. Li, H. P. Hratchian, A. F. Izmaylov, J. Bloino, G. Zheng, J. L. Sonnenberg, M. Hada, M. Ehara, K. Toyota, R. Fukuda, J. Hasegawa, M. Ishida, T. Nakajima, Y. Honda, O. Kitao, H. Nakai, T. Vreven, J. A. Montgomery, Jr., J. E. Peralta, F. Ogliaro, M. Bearpark, J. J. Heyd, E. Brothers, K. N. Kudin, V. N. Staroverov, R. Kobayashi, J. Normand, K. Raghavachari, A. Rendell, J. C. Burant, S. S. Iyengar, J. Tomasi, M. Cossi, N. Rega, J. M. Millam, M. Klene, J. E. Knox, J. B. Cross, V. Bakken, C. Adamo, J. Jaramillo, R. Gomperts, R. E. Stratmann, O. Yazyev, A. J. Austin, R. Cammi, C. Pomelli, J. W. Ochterski, R. L. Martin, K. Morokuma, V. G. Zakrzewski, G. A. Voth, P. Salvador, J. J. Dannenberg, S. Dapprich, A. D. Daniels, Ö. Farkas, J. B. Foresman, J. V. Ortiz, J. Cioslowski and D. J. Fox, Gaussian 09, Revision E.01, Gaussian, Inc., Wallingford CT, **2009**.

[19] A. D. Boese and N. C. Handy, A new parametrization of exchange–correlation generalized gradient approximation functionals, *J. Chem. Phys.* **2001**, *114*, 5497-5503.
